# Trends in three malnutrition factors in the global burden of disease: iodine deficiency, vitamin A deficiency, and protein-energy malnutrition (1990–2019)

**DOI:** 10.3389/fnut.2024.1426790

**Published:** 2024-07-23

**Authors:** Shaorong Ji, Yinglu Zhou, Qilong Zhao, Runtong Chen, Zhenni Su

**Affiliations:** ^1^Shandong Provincial Third Hospital, Shandong University, Jinan, Shandong, China; ^2^Cao Pu Town Health and Family Planning Clinic, Anning Medical Community, Kunming, Yunnan, China; ^3^School of Public Health, Lanzhou University, Lanzhou, Gansu, China

**Keywords:** vitamin A deficiency, iodine deficiency, protein-energy malnutrition, global burden, age-standardized rate

## Abstract

**Background:**

Vitamin A deficiency, iodine deficiency, and protein-energy malnutrition are prevalent malnutrition issues that disproportionately affect low-income countries and pose significant risks to the health and development of children and adolescents. This study offers a detailed examination of these deficiencies' prevalence trends and gender and regional variations using Global Burden of Disease Study data from 1990 to 2019. It also assesses the specific impact on various age groups, providing essential insights for targeted health interventions and policy-making.

**Methods:**

Data spanning from 1990 to 2019 on Vitamin A deficiency, iodine deficiency, and protein-energy malnutrition were extracted from the 2019 Global Burden of Disease Study. Age-Standardized Incidence Rates (ASR) were computed by gender, region, and etiology, utilizing the estimated annual percentage change (EAPC) to assess temporal trends.

**Results:**

In 2019, Central Sub-Saharan Africa had the highest prevalence of Vitamin A deficiency, particularly among males, and iodine deficiency peaked in the same region for both genders. South Asia had the highest incidence of protein-energy malnutrition for both genders. Regions with a low Socio-Demographic Index (SDI) showed lower ASR for these deficiencies. Notably, Cameroon, Equatorial Guinea, and Maldives recorded the highest ASR for vitamin A deficiency, iodine deficiency, and protein-energy malnutrition, respectively. The declining ASR trend for vitamin A deficiency, especially among males, suggests effective interventions. East Asia saw a significant increase in iodine deficiency ASR from 1990 to 2019, particularly among women, requiring targeted interventions. The rising ASR of protein-energy malnutrition in several regions, especially among men, raises concerns. Vitamin A deficiency primarily affected children and adolescents, iodine deficiency predominantly impacted adolescents and young adults, and protein-energy malnutrition was chiefly observed among children under 5 years old. These findings underscore the necessity for tailored interventions considering age-specific nutritional needs and challenges.

## 1 Introduction

Malnutrition remains a pervasive global health challenge, with far-reaching implications for both individual wellbeing and public health. Iodine, vitamin A, protein, and energy are essential nutrients for the human body, playing a role in numerous physiological functions. Insufficiency or deficiency of these nutrients can result in significant health problems. Iodine deficiency is the leading cause of preventable cognitive impairments and is linked to thyroid dysfunction, miscarriages, premature births, stillbirths, and congenital deformities (1). Vitamin A deficiency is a significant factor in childhood blindness and mortality, as well as its links to infectious diseases, anemia, and reproductive health (2). Insufficient protein and energy intake can weaken the immune system, elevating susceptibility to infections and mortality. The World Health Organization acknowledges iodine deficiency, vitamin A deficiency, and protein-energy malnutrition as global public health issues (3). As per the 2020 Global Nutrition Report, iodine deficiency, vitamin A deficiency, and protein-energy malnutrition represent substantial global risk factors for mortality and disability-adjusted life years (DALYs) (4). Despite substantial public health endeavors in recent decades to tackle this problem, elevated prevalence rates endure in low- and middle-income regions (5). The main reasons include economic constraints leading to a monotonous diet, lack of education and awareness about nutrition, soil and environmental factors affecting the nutritional value of food, heavy disease burdens impacting nutrient absorption, and social and cultural customs restricting the intake of nutrient-rich foods. Moreover, inadequate health systems, absence of targeted nutrition policies, and the adverse effects of climate change on agricultural production have all exacerbated nutritional problems in these countries (6, 7).

In 2015, the United Nations General Assembly set forth the Sustainable Development Goals (SDGs) to eliminate all malnutrition forms by 2030 (8). This encompasses tackling health issues like child growth stunting, wasting, adolescent malnutrition, and the nutritional needs of pregnant, lactating women, and the elderly. To attain this goal, acquiring an understanding of the patterns and temporal trends in iodine deficiency, vitamin A deficiency, and protein-energy malnutrition incidence can facilitate the adoption of more focused preventive strategies, thereby contributing to the attainment of the Sustainable Development Goals.

The Global Burden of Disease (GBD) study has evaluated the prevalence of iodine deficiency, vitamin A deficiency, and protein-energy malnutrition in 204 countries and regions across the globe (9). This provides a distinct opportunity to gain insights into how to address these deficiencies. In this study, we aim to evaluate the temporal trends in the incidence of iodine deficiency, vitamin A deficiency, and protein-energy malnutrition from a global, regional, and national perspective over the period from 1990 to 2019. Our research findings can contribute to the development of targeted prevention and intervention strategies that are tailored to the needs of different nations and populations.

## 2 Materials and methods

### 2.1 Study data

We gathered data on the incidence, age-standardized incidence, and mortality rates of iodine deficiency, vitamin A deficiency, and protein-energy malnutrition, broken down by gender, region, and country. These data span from 1990 to 2019 and were collected through the GBD Results Tool (GHDx) available at: http://ghdx.healthdata.org/gbd-results-tool. The dataset encompassed 204 countries and territories. Subsequently, we categorized these nations and regions into five groups based on the sociodemographic index (SDI), which includes low, low-middle, middle, high-middle, and high categories. Additionally, we partitioned the globe into 21 geographical regions (Supplementary Table 1). A comprehensive account of the indicators, data sources, and statistical models employed in GBD 2019 has been documented in previous publications and adheres to the Guidelines for Accurate and Transparent Health Estimates Reporting (9, 10). According to the International Classification of Diseases, Tenth Revision (ICD-10), protein-energy malnutrition is coded as E40–E46.9; iodine deficiency is coded as E00-E02, and vitamin A deficiency is coded as E50–E50.9.

### 2.2 Statistical analysis

To assess the trends in iodine deficiency, vitamin A deficiency, and protein-energy malnutrition, we use age-standardized incidence rates (ASR) and estimated annual percentage change (EAPC). Direct comparison between populations with different age structures can introduce bias in crude rate comparisons. Hence, standardizing rates is essential. ASR (per 100,000 individuals) is calculated using the direct method and is defined by Equation (1). EAPC is a commonly utilized metric for summarizing rate trends over specific time intervals. It is computed using a linear regression model, as represented in Equations (2, 3). We determine a 95% confidence interval using the EAPC formula mentioned earlier. The standard error is obtained from fitting the regression line. If both the estimated EAPC value and its lower 95% confidence limit are >0, the age-standardized rate is considered to be increasing. Conversely, if both the estimated EAPC value and its upper 95% confidence limit are <0, the age-standardized rate is considered to be decreasing. All statistical analyzes were conducted using the R program (Version 4.2.1, R Core Team). A *P*-value <0.05 was regarded as statistically significant.


(1) $$ASR=\frac{\sum_{i=1}^{A}a_{i}\omega_{i}}{\sum_{i=1}^{A}\omega_{i}}\times 100,000$$

where i denotes the i^*th*^ age class; *a* denotes age-specific rates; ω denotes the number of people (or weight)


(2) $$\begin{array}{l} y=\alpha +\beta x+\epsilon \end{array}$$

(3) $$\begin{array}{l} EAPC=100\times \left(exp\left(\beta \right)-1\right) \end{array}$$

where y represents the natural logarithm of the age-standardized rate, x corresponds to the years in question, and β stands for the estimated slope.

## 3 Results

### 3.1 Global burden of disease of vitamin A deficiency, iodine deficiency, and protein-energy malnutrition in 2019

In 2019, global Age-Standardized Rates (ASRs) for vitamin A deficiency, iodine deficiency, and protein-energy malnutrition were 6,955.6, 108.3, and 2,099.4 per 100,000, respectively. Among females, ASRs were 5,999.1, 139.8, and 1,894.6 per 100,000, and among males, 7,886.2, 78.1, and 2,304 per 100,000 (Table 1). According to the Social Demographic Index (SDI), regions with low SDI had lower ASRs for vitamin A deficiency, iodine deficiency, and protein-energy malnutrition, while high SDI regions exhibited the opposite trend (Supplementary Tables 1, 4, 7). In 2019, among the 21 regions, Central Sub-Saharan Africa had the highest ASR for vitamin A deficiency at 33,739.9 per 100,000 (range: 30,648–37,138.4), followed by Eastern Sub-Saharan Africa and Western Sub-Saharan Africa. For females, Eastern Sub-Saharan Africa had the highest ASR for vitamin A deficiency in 2019 at 20,731.4 per 100,000 (range: 19,414.7–22,180.9), while for males, it was Central Sub-Saharan Africa with an ASR of 33,242.3 per 100,000 (range: 28,347.2–38,656.5; Supplementary Tables 4–6). Central Sub-Saharan Africa had the highest ASR for iodine deficiency in 2019, at 459 per 100,000 (range: 371.5–555.8), followed by South Asia and Eastern Sub-Saharan Africa. For both females and males, the highest ASRs were in Central Sub-Saharan Africa, with 459 per 100,000 (range: 371.5–555.8) and 320.5 per 100,000 (range: 255–391.2), respectively (Supplementary Tables 1–3). In 2019, the region with the highest ASR for protein-energy malnutrition was South Asia at 1,023.5 per 100,000 (range: 860–1,208.2), followed by Southeast Asia and East Asia. For both females and males, the highest ASRs of protein-energy malnutrition were in South Asia, with 3,427.8 per 100,000 (range: 2,828.8–4,111.8) and 3,759.6 per 100,000 (range: 3,101.9–4,516), respectively (Supplementary Tables 7–9).

**Table 1:** The incident cases and age-standardized incidence of vitamin A deficiency, iodine deficiency, and protein-energy malnutrition in 1990 and 2019, and its temporal trends from 1990 to 2019.

**Global**	**Vitamin A deficiency**	**Iodine deficiency**	**Protein-energy malnutrition**
**Both**			
Incident cases (1990) No. × 10^4^[95% UI]	877,376.3 (840,347–914,976.5)	4,466.9 (3,658.2–5,407.2)	111,389.2 (91,268.4–136,380.2)
ASR per 100,000 (1990) No.[95% UI]	17,323.2 (16,526.5–18,138.9)	152.1 (125–183.9)	1,896.6 (1,563.7–2,293.1)
Incident cases (2019) No. × 10^4^[95% UI]	489,662.7 (469,006.4–512,234.3)	5,130.5 (4,124–6,327.5)	154,086 (128,445.2–183,279)
ASR per 100,000 (2019) No.[95% UI]	6,955.6 (6,645.9–7,294.2)	139.8 (112.5–171.8)	2,099.4 (1,752.8–2,487.4)
EAPC (1990-2019) No.[95% CI]	–3.11 (–3.25 to –2.98)	–0.23 (–0.33 to –0.13)	–0.03 (–0.19 to 0.13)
**Female**			
Incident cases (1990) No. × 10^4^[95% UI]	336,074.1 (322,087–351,326.5)	7,711.7 (6,265.2–9,349.8)	59,764 (49,351.5–72,376.6)
ASR per 100,000 (1990) No.[95% UI]	13,456.5 (12,848.5–14,141.4)	129.1 (105.2–156.7)	2,013.2 (1,677.5–2,409.1)
Incident cases (2019) No. × 10^4^[95% UI]	208,933.1 (199,953.4–218,963.7)	8,111.5 (6,500.1–9,966.1)	85,880 (71,398.6–102,291.9)
ASR per 100,000 (2019) No.[95% UI]	5,999.1 (5,719–6,307.3)	108.3 (86.8–133.3)	2,304 (1,918.2–2,735.2)
EAPC (1990-2019) No.[95% CI]	–2.75 (–2.93 to –2.56)	–0.44 (–0.58 to –0.31)	0.13 (–0.01 to 0.28)
**Male**			
Incident cases (1990) No. × 10^4^[95% UI]	541,302.2 (509,180.6–576,528.3)	3,244.8 (2,620.2–3,932.9)	51,625.2 (41,920.1–63,990.1)
ASR per 100,000 (1990) No.[95% UI]	21,073.8 (19,711.5–22,557.3)	106.7 (86.8–129.4)	1,781.6 (1,456.6–2,184.4)
Incident cases (2019) No. × 10^4^[95% UI]	280,729.6 (263,694.4–300,226.4)	2,981 (2,385.9–3,715.7)	68,206 (56,809.7–80,628.8)
ASR per 100,000 (2019) No.[95% UI]	7,886.2 (7,367.7–8,489.8)	78.1 (62.6–97.1)	1,894.6 (1,583.6–2,246.7)
EAPC (1990-2019) No.[95% CI]	–3.36 (–3.46 to –3.26)	–0.77 (–0.95 to –0.58)	–0.23 (–0.42 to –0.04)

ASR, age standardized rate; CI, confidence interval; EAPC, estimated annual percentage change; UI, uncertainty interval.

**Figure 1:**
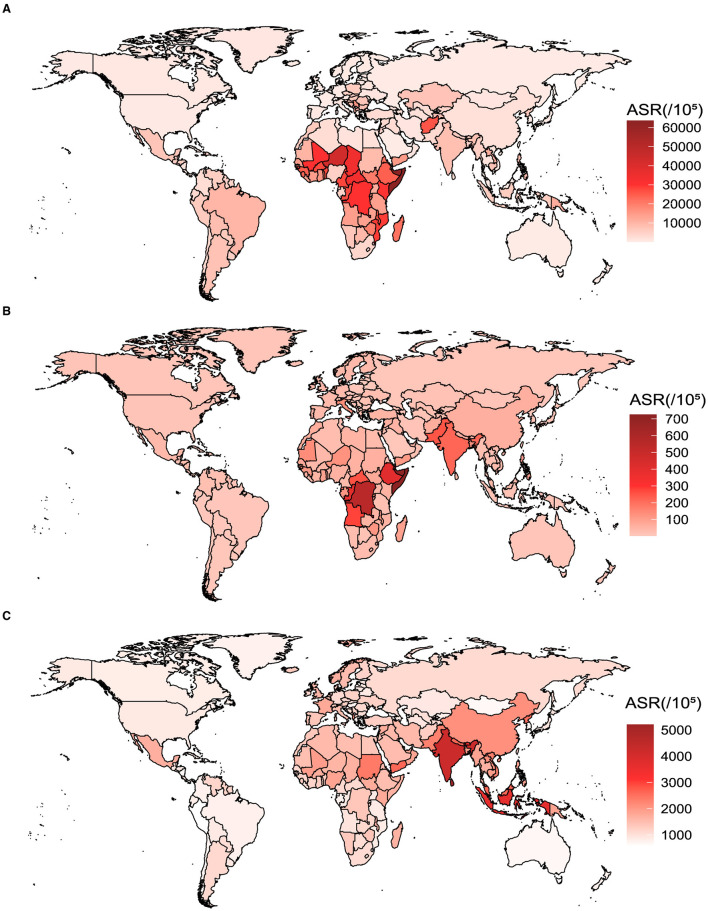
The ASR of vitamin A deficiency, iodine deficiency, and protein-energy malnutrition for both genders in 204 countries and territories in 2019. **(A)** Vitamin A deficiency. **(B)** Iodine deficiency. **(C)** Protein-energy malnutrition.

In 204 countries and territories worldwide, there are significant variations in the ASR of vitamin A deficiency, iodine deficiency, and protein-energy malnutrition (Figure 1). In 2019, Cameroon exhibited the most pronounced ASR for vitamin A deficiency, reaching 69,494.5 per 100,000 (within the range of 66,902.4–72,335.4), surpassing Somalia and Niger. For females, the country with the highest ASR for vitamin A deficiency was Somalia at 58,377.3 per 100,000 (ranging from 53,054.1 to 63,870.7), while for males, it was Cameroon at 92,312.4 per 100,000 (ranging from 89,901.8 to 94,293.4; Supplementary Tables 13–15; Supplementary Figures 1, 2). Equatorial Guinea recorded the highest ASR for iodine deficiency at 1,071.8 per 100,000 (ranging from 902.5 to 1,226.4), followed by the Democratic Republic of the Congo and Somalia. The countries with the highest ASR for iodine deficiency among females and males were Equatorial Guinea, with 1,387.4 per 100,000 (ranging from 1,193.1 to 1,571.3), and 760 per 100,000 (ranging from 613.2 to 899.1), respectively (Supplementary Tables 10–12; Supplementary Figures 1, 2). Maldives recorded the highest ASR for protein-energy malnutrition, with a level of 4,292.4 per 100,000 (ranging from 3,565.3 to 5,068.5), surpassed only by Sri Lanka and Timor-Leste. For females, the country with the highest ASR for protein-energy malnutrition was Maldives at 3,421.6 per 100,000 (ranging from 2,819.4 to 4,204.5), while for males, it was Sri Lanka at 5,876.1 per 100,000 (ranging from 4,963.3 to 6,809.5; Supplementary Tables 16–18; Supplementary Figures 1, 2).

In absolute terms, India exhibited the highest cases of vitamin A deficiency, with 292,439,000 cases in 2019, followed by China and Indonesia (Supplementary Table 5). India also bore the greatest burden of individuals affected by iodine deficiency, reporting 37,856,000 cases in 2019, trailed by China and Bangladesh. Notably, India carried the heaviest load of protein-energy malnutrition, with an astounding count of 376,672,000 cases, surpassed by China and Indonesia.

### 3.2 Changes in the burden of vitamin A deficiency, iodine deficiency, and protein-energy malnutrition over time

**Figure 2:**
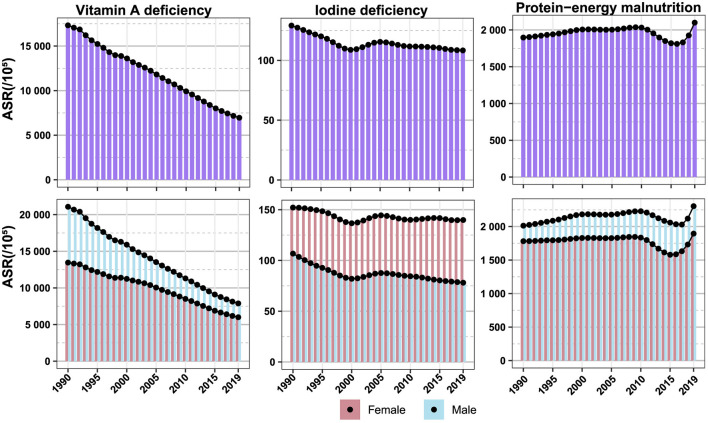
Changes in the burden of vitamin A deficiency, iodine deficiency, and protein-energy malnutrition over time.

Globally, from 1990 to 2019, there has been a declining trend in the ASR of vitamin A deficiency, with a more pronounced decrease in males compared to females. Similar patterns are observed across different SDI regions (Figure 2; Supplementary Figures 3–7). The ASR for iodine deficiency exhibited a global decline from 1990 to 2000, followed by a slow increase from 2000 to 2005, and a subsequent decline. In high SDI, low-middle SDI, and low SDI regions, the ASR for iodine deficiency decreased from 1990 to 2019, while in high-middle SDI and middle SDI regions, there was a pattern of decrease, increase, and then decrease (Figure 2; Supplementary Figures 3–7). From 1990 to 2010, the global ASR for protein-energy malnutrition showed an increasing trend, followed by a decline from 2010 to 2015, and then a subsequent increase. In high SDI, high-middle SDI, and middle SDI regions, the ASR for protein-energy malnutrition exhibited an upward trend from 1990 to 2010, followed by a decline from 2010 to 2015. Conversely, in low-middle SDI and low SDI regions, there was a decline in the ASR from 2010 to 2015. Post-2015, different SDI regions witnessed an upward trend in the ASR for protein-energy malnutrition (Figure 2; Supplementary Figures 3–7). Furthermore, on a global scale and across different SDI regions, the general trend in the ASRs of vitamin A deficiency, iodine deficiency, and protein-energy malnutrition remained consistent from 1990 to 2019 for both males and females. However, the ASR were generally higher in males for vitamin A deficiency and protein-energy malnutrition, while females exhibited higher ASR for iodine deficiency (Figure 2; Supplementary Figures 3–7). In 21 regions worldwide, from 1990 to 2019, the ASR of iodine deficiency in East Asia showed an increasing trend of (EAPC = 0.17; 95% CI: –0.28 to 0.62), mainly among women (EAPC = 0.67; 95% CI: 0.30 to 1.04). The ASR of protein-energy malnutrition in the regions of Australasia, East Asia, Central Europe, Western Europe, and Southern Latin America also showed an upward trend, more pronounced in men than in women (EAPC > 0). No regions were found where the ASR of vitamin A deficiency showed an increasing trend from 1990 to 2019 (EAPC <0; Figure 2; Supplementary Tables 1, 4, 7).

**Figure 3:**
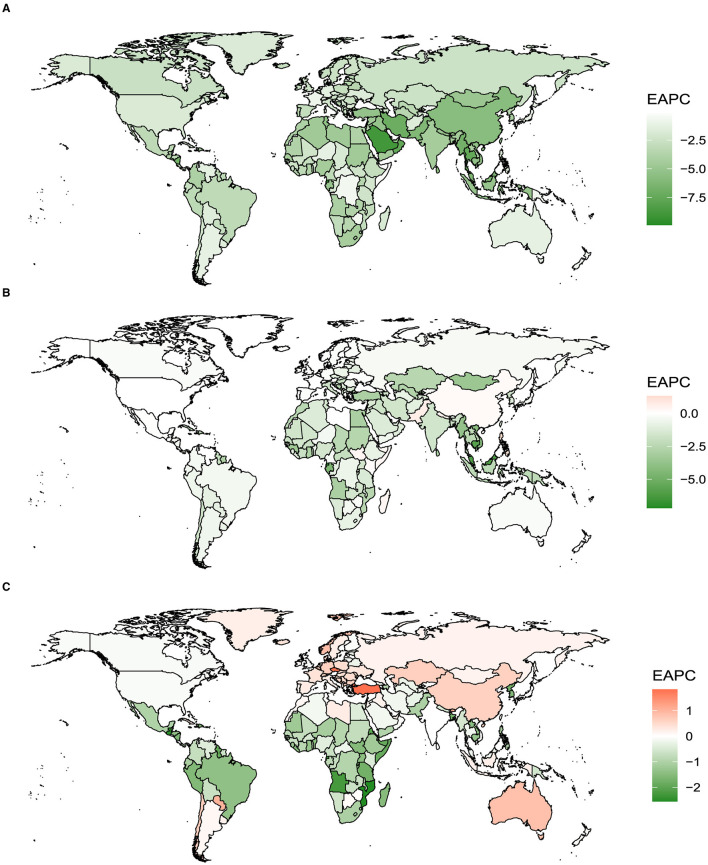
The EAPC of iodine deficiency, vitamin A deficiency, and protein-energy malnutrition for both genders in 204 countries and territories from 1990 to 2019. **(A)** Vitamin A deficiency. **(B)** Iodine deficiency. **(C)** Protein-energy malnutrition.

From 1990 to 2019, the ASR of vitamin A deficiency showed a decreasing trend (EAPC <0) in 204 countries globally. Among them, Equatorial Guinea exhibited the most significant decline (EAPC = –9.90; 95% CI: –10.39 to –9.41), followed by Saudi Arabia and Maldives. In both females (EAPC = –9.88; 95% CI: –10.32 to –9.43) and males (EAPC = –10.09; 95% CI: –10.62 to –9.56), Equatorial Guinea showed the most prominent decrease in the ASR for vitamin A deficiency (Figure 3; Supplementary Figures 8, 9; Supplementary Tables 13–15). Twelve countries and regions experienced an increasing trend in the ASR for iodine deficiency from 1990 to 2019. The sequence of this upward trend included the following: Philippines, Pakistan, Nepal, Republic of Moldova, South Sudan, Madagascar, Somalia, China, Kenya, Monaco, Portugal, and Andorra. Among females, the countries and regions with an increasing trend in the ASR for iodine deficiency included Philippines, Nepal, Pakistan, China, Republic of Moldova, South Sudan, and others, while among males, it was mainly observed in countries like Philippines, Somalia, Romania, and others (Figure 3; Supplementary Figures 8, 9; Supplementary Tables 10–12). Turkey, Czechia, Bhutan, Montenegro, Paraguay, Norway, Australia, and China, among others, witnessed an increasing trend in the ASR for protein-energy malnutrition from 1990 to 2019. Notably, among females, this trend was prominent in Czechia, Turkey, Qatar, Paraguay, Norway, Montenegro, and others, while among males, it was observed in Bhutan, Turkey, Czechia, China, Montenegro, Australia, and others (Figure 3; Supplementary Figures 8, 9; Supplementary Tables 16–18).

### 3.3 Age composition of vitamin A deficiency, iodine deficiency, and protein-energy malnutrition

**Figure 4:**
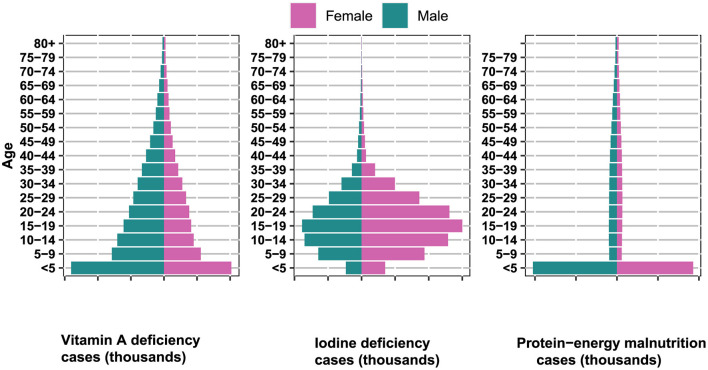
Global age-sex distribution of incident cases of iodine deficiency, vitamin A deficiency, and protein-energy malnutrition in 2019.

In the year 2019, on a worldwide scale (Figure 4), the occurrence of vitamin A deficiency was predominantly centered on the demographic of children and adolescents. Concurrently, iodine deficiency predominantly impacted adolescents and young adults. The focal point of protein-energy malnutrition was observed predominantly among the age group of children under 5 years old.

## 4 Discussion

Nutritional deficiencies have profound implications for societal development. They compromise individual health and productivity, increase healthcare burdens, and impede economic growth. Furthermore, education and learning outcomes are adversely affected, diminishing the overall developmental potential of nations (11–13). In this study, we conducted a comprehensive analysis of the disease burden associated with vitamin A deficiency, iodine deficiency, and protein-energy malnutrition at the global, regional, and national levels to identify areas where these issues persist as significant public health concerns.

Our research indicates that vitamin A deficiency is particularly prevalent in Central Africa, especially in Cameroon, where the ASR of vitamin A deficiency among men is the highest. Zhao et al.'s study reveals that in 2019, the age-standardized DALY rate for vitamin A deficiency was highest in Central Sub-Saharan Africa, followed by Western Sub-Saharan Africa and again Western Sub-Saharan Africa (14).This condition is primarily influenced by poor dietary patterns, environmental pollution of water and soil, infections and diseases, and insufficient iodine content in salt (15–17). The local population predominantly relies on cereals, tubers, and legumes as their main food sources, while the intake of animal products, green or yellow vegetables, and fruits—foods rich in vitamin A or carotenoids—is relatively low, leading to inadequate vitamin A consumption (18). From 1990 to 2019, the annual incidence rate of vitamin A deficiency has declined, with a more pronounced decrease among men, indicating that the interventions and strategies implemented have achieved some success. Since 2000, mortality due to vitamin A deficiency has decreased by over 50%, likely attributable to improved nutritional status, enhanced water and sanitation conditions (such as the control of diarrhea), vaccination (such as measles vaccination), and vitamin A supplementation programs (19–21). However, the high incidence among women persists, suggesting the need for ongoing efforts and gender-specific strategies. our study highlights that vitamin A deficiency has become a major issue among children and adolescents, a particularly vulnerable group for whom vitamin A is crucial for vision, immune function, and overall growth (22). Global research also identifies vitamin A deficiency in children and adolescents as an increasingly significant problem, underscoring the urgency of addressing this issue (23).

In Central Africa, Equatorial Guinea exhibits the highest ASR of iodine deficiency. Research reports that in 2019, Somalia, the Democratic Republic of the Congo, Djibouti, and the Republic of the Congo had the highest age-standardized prevalence rates. Meanwhile, countries like the Philippines and Pakistan have shown an increasing trend. Geographically, the highest prevalence rates are found in Central Sub-Saharan Africa and South Asia (24). The primary cause is the limited consumption of iodine-rich foods such as seafood, milk, and eggs. Additionally, some foods contain goitrogens, such as thiocyanates and soy isoflavones, which inhibit iodine absorption and utilization, exacerbating iodine deficiency (25). According to a report, low iodine levels in soil and water in the region affect both crops and drinking water, contributing to the deficiency. Moreover, soil and water contamination by heavy metals, pesticides, and industrial effluents further destabilize and reduce iodine availability (15, 26, 27). Consuming iodized salt is an effective and economical method to prevent and control iodine deficiency. However, in sub-Saharan Africa, the coverage and quality of iodized salt remain inadequate, perpetuating the deficiency issue. Contributing factors include weak enforcement of iodization policies, insufficient monitoring and evaluation, outdated technology and equipment, cost-benefit disparities, and socio-cultural barriers (15, 28). This study indicates that the global ASR of iodine deficiency decreased from 1990 to 2000, increased from 2000 to 2005, and then declined again, reflecting the impact of global initiatives, particularly the Universal Salt Iodization (USI) policy (29). According to UNICEF's 2020 report, 124 countries have mandated iodized salt, 21 allow voluntary iodization, and 88% of the global population uses iodized salt (30). However, challenges persist in implementing the USI policy, including inaccurate iodine levels in salt, weak quality control and enforcement, low public awareness and education, and a lack of regular surveys and monitoring of iodine status and iodization program impacts (31). These challenges may explain the ASR increase from 2000 to 2005, as some countries experienced a resurgence or persistence of iodine deficiency due to weakened or interrupted USI policies. Research also indicates a unique global pattern of iodine deficiency, predominantly affecting adolescents and young adults. Adequate iodine intake is crucial for thyroid function, and its deficiency can lead to various health issues, particularly during the critical developmental stages of adolescence and early adulthood (32). Therefore, ensuring sufficient iodine intake in this age group is vital for preventing related health problems.

Protein-energy malnutrition is prevalent in South Asia, affecting both genders equally. Xu et al.'s research indicates that South Asia, Southeast Asia, and East Asia rank highest in age-standardized prevalence (33). This phenomenon is likely linked to the region's dense population and limited land resources. Additionally, climate change, natural disasters, and pests impact agricultural production, resulting in unstable grain yields and subsequent food shortages (33–35). Furthermore, pervasive poverty, low per capita income, and high food prices in South Asia make it challenging for many to access sufficient protein and energy-rich foods (36). Social and cultural factors also play a significant role in food distribution and consumption. In some areas, women and children are disadvantaged in food allocation, leading to insufficient protein and energy intake. For example, in regions like India, religious and cultural practices often limit the intake of animal-based foods, with vegetarianism being widespread (7, 37, 38). Studies show a correlation between the Socio-Demographic Index (SDI) and the ASR of nutritional deficiencies. Regions with lower SDI tend to have lower ASR, indicating that socio-economic factors play a crucial role in the prevalence of nutritional deficiencies (39). This finding underscores the need for comprehensive strategies addressing both nutritional needs and broader socio-economic determinants. In absolute terms, India bears the greatest burden of nutritional deficiencies, followed by China and Indonesia. This may be attributed to their large populations, highlighting the need for country-specific strategies to address these issues. However, it is essential to note that while discussing global averages, significant variations can exist within countries in the same region (40). The global ASR of protein-energy malnutrition has experienced fluctuations since 1990, with an initial rise, a subsequent decline, and another increase post-2015, raising concerns and calls for renewed efforts to address the issue. The rising trend in ASR in regions like Australia, East Asia, Central Europe, Western Europe, and Latin America, particularly among males, warrants attention. This indicates the influence of socio-economic factors on nutritional deficiencies in different SDI regions, necessitating region-specific strategies (33). Protein-energy malnutrition primarily affects children under five, highlighting the importance of addressing nutritional needs early in life. During this critical growth and development stage, deficiencies in protein and energy can have long-term impacts on physical and cognitive development (41). Therefore, early nutritional interventions are vital for healthy childhood development.

To address these global nutritional challenges, comprehensive strategies are imperative. First, targeted public health campaigns should elevate awareness among children and adolescents about the critical importance of a vitamin A-rich diet, emphasizing its roles in vision, immune health, and overall growth. Additionally, strengthening iodine supplementation programs and educational initiatives for adolescents and young adults is crucial to ensure optimal thyroid function and prevent associated health issues. To combat protein-energy malnutrition in children under five, expanding community-based nutrition programs to provide access to nutrient-dense foods and offer nutrition education to caregivers is essential. Collaboration among governments, non-governmental organizations, and international agencies is vital for developing and implementing sustainable solutions, promoting a holistic approach to global nutrition across all age groups.

To the best of our knowledge, this study represents the first comprehensive overview and exploration of global disparities in vitamin A deficiency, iodine deficiency, and protein-energy malnutrition, along with their evolving patterns stratified by gender and age. This research has inherent limitations. Despite the adjustments made in GBD 2019 to account for biases and methodological flaws in low-quality sampling, survey methods, and other data sources, the accuracy of the results is significantly contingent upon the quality and quantity of the input data into the model. Moreover, the absence of diagnostic gold standards and the potential interchangeability of these terms might lead to an underestimation of the scale of these diseases.

## 5 Conclusions

Over the past several decades, substantial improvements have been made in addressing global issues related to vitamin A deficiency, iodine deficiency, and protein-energy malnutrition. However, progress remains uneven on a global scale, with nutritional deficiencies posing persistent concerns in countries with lower socioeconomic levels. Urgent policy safeguards and ongoing efforts are imperative to control nutritional deficiencies in these regions. Vitamin A deficiency predominantly impacts children and adolescents, while iodine deficiency primarily affects adolescents and young adults. Meanwhile, protein-energy malnutrition is primarily observed among children under the age of 5. These findings underscore the necessity for age-specific interventions tailored to the distinct nutritional needs and challenges of each age group. Further research is warranted to comprehend and tackle the underlying causes of these age-specific trends.

## Data availability statement

The datasets presented in this study can be found in online repositories. The names of the repository/repositories and accession number(s) can be found in the article/Supplementary material.

## Ethics statement

The studies involving humans were approved by Health Metrics and Evaluation (IHME) at the University of Washington. The studies were conducted in accordance with the local legislation and institutional requirements. The participants provided their written informed consent to participate in this study.

## Author contributions

SJ: Conceptualization, Data curation, Formal analysis, Methodology, Writing — original draft, Writing — review & editing. YZ: Data curation, Formal analysis, Methodology, Writing — original draft, Writing — review & editing. QZ: Data curation, Methodology, Writing — original draft. RC: Data curation, Visualization, Writing — original draft. ZS: Writing — original draft.
