# Supplementary material for: Trends in three malnutrition factors in the global burden of disease: iodine deficiency, vitamin A deficiency, and protein-energy malnutrition (1990–2019)
Source: Front Nutr. 2024 Jul 23;11:1426790. doi: 10.3389/fnut.2024.1426790 (PMC11300326; doi:10.3389/fnut.2024.1426790)
Supplement: Supplementary file 1 [file Data_Sheet_1.PDF]

Trends in three malnutrition factors in the global burden of disease: iodine deficiency, vitamin A deficiency, and protein-energy malnutrition (1990-2019)

July 6, 2024

List of Figures

S1 The ASR of iodine deficiency, vitamin A deficiency, and protein-energy malnutrition for female in 204 countries and territories in 2019. (A) iodine deficiency; (B) Vitamin A deficiency; (C) Protein-energy malnutrition . . . 10
S2 The ASR of iodine deficiency, vitamin A deficiency, and protein-energy malnutrition for mele in 204 countries and territories in 2019. (A) iodine deficiency; (B) Vitamin A deficiency; (C) Protein-energy malnutrition . . . 11
S3 Changes in the burden of iodine deficiency, vitamin A deficiency, and protein-energy malnutrition over time in high SDI regions. . . . 12
S4 Changes in the burden of iodine deficiency, vitamin A deficiency, and protein-energy malnutrition over time in high-middle SDI regions. . . . 13
S5 Changes in the burden of iodine deficiency, vitamin A deficiency, and protein-energy malnutrition over time in middle SDI regions. . . . 14
S6 Changes in the burden of iodine deficiency, vitamin A deficiency, and protein-energy malnutrition over time in low-middle SDI regions. . . . 15
S7 Changes in the burden of iodine deficiency, vitamin A deficiency, and protein-energy malnutrition over time in low SDI regions. . . . 16
S8 The EAPC of iodine deficiency, vitamin A deficiency, and protein-energy malnutrition for female in 204 countries and territories from 1990 to 2019. (A) iodine deficiency; (B) Vitamin A deficiency; (C) Protein-energy malnutrition . . . 17
S9 The EAPC of iodine deficiency, vitamin A deficiency, and protein-energy malnutrition for male in 204 countries and territories from 1990 to 2019. (A) iodine deficiency; (B) Vitamin A deficiency; (C) Protein-energy malnutrition . . . 18

List of Tables

S1 The incident cases and age-standardized incidence of iodine deficiency for both gender in 1990 and 2019, and its temporal trends from 1990 to 2019. . . . 1
S2 The incident cases and age-standardized incidence of iodine deficiency for female in 1990 and 2019, and its temporal trends from 1990 to 2019. . . . 2
S3 The incident cases and age-standardized incidence of iodine deficiency for male in 1990 and 2019, and its temporal trends from 1990 to 2019. . . . 3
S4 The incident cases and age-standardized incidence of Vitamin A deficiency for both gender in 1990 and 2019, and its temporal trends from 1990 to 2019. . . . 4
S5 The incident cases and age-standardized incidence of Vitamin A deficiency for female in 1990 and 2019, and its temporal trends from 1990 to 2019. . . . 5
S6 The incident cases and age-standardized incidence of Vitamin A deficiency for male in 1990 and 2019, and its temporal trends from 1990 to 2019. . . . 6
S7 The incident cases and age-standardized incidence of protein-energy malnutrition for both gender in 1990 and 2019, and its temporal trends from 1990 to 2019. . . . 7
S8 The incident cases and age-standardized incidence of protein-energy malnutrition for female in 1990 and 2019, and its temporal trends from 1990 to 2019. . . . 8
S9 The incident cases and age-standardized incidence of protein-energy malnutrition for male in 1990 and 2019, and its temporal trends from 1990 to 2019. . . . 9
S10 The change of iodine deficiency cases between 1990 and 2019 at national level for both gender. . . 19
S11 The change of iodine deficiency cases between 1990 and 2019 at national level for female. . . . 25
S12 The change of iodine deficiency cases between 1990 and 2019 at national level for male. . . . 31
S13 The change of vitamin A deficiency cases between 1990 and 2019 at national level for both gender. 37

S14 The change of vitamin A deficiency cases between 1990 and 2019 at national level for female. . . 44
S15 The change of vitamin A deficiency cases between 1990 and 2019 at national level for male. . . . 51
S16 The change of protein-energy malnutrition cases between 1990 and 2019 at national level for both gender. 59
S17 The change of protein-energy malnutrition cases between 1990 and 2019 at national level for female. 66
S18 The change of protein-energy malnutrition cases between 1990 and 2019 at national level for male. 73

Table S1: The incident cases and age-standardized incidence of iodine deficiency for both gender in 1990 and 2019, and its temporal trends from 1990 to 2019.

Characteristics	1990		2019		1990–2019
	Incident cases No.$\times 10^4$[95% UI]	ASR per 10,0000 No.[95% UI]	Incident cases No.$\times 10^4$[95% UI]	ASR per 10,0000 No.[95% UI]	EAPC No.[95% CI]
Global	7711.7 (6265.2 to 9349.8)	129.1 (105.2 to 156.7)	8111.5 (6500.1 to 9966.1)	108.3 (86.8 to 133.3)	-0.44 (-0.58 to -0.31)
Socio-demographic index					
High SDI	175.7 (138.8 to 221.2)	22.3 (17.7 to 28.1)	178.8 (142.4 to 222.9)	20.5 (16.4 to 25.8)	-0.29 (-0.30 to -0.27)
High-middle SDI	724.6 (584.5 to 900.8)	61.5 (49.9 to 75.8)	617.6 (488.9 to 761.4)	51.1 (40.7 to 63.4)	-0.68 (-0.83 to -0.54)
Middle SDI	1686.2 (1337.6 to 2096.8)	84.3 (67.4 to 104.3)	1615.8 (1282.3 to 2017)	69.9 (55.4 to 87.2)	-0.61 (-0.70 to -0.52)
Low-middle SDI	3355 (2744.5 to 4037)	240.5 (197.3 to 291.4)	2854.9 (2219.9 to 3600)	146.8 (114.9 to 184.4)	-1.55 (-1.86 to -1.24)
Low SDI	1768.5 (1472.2 to 2093)	268.2 (225 to 320.2)	2842.4 (2307.2 to 3459.7)	205 (167.1 to 248.2)	-0.83 (-0.98 to -0.69)
Region					
Andean Latin America	2.2 (1.7 to 2.9)	5.6 (4.2 to 7.1)	3.1 (2.3 to 4)	4.8 (3.6 to 6.2)	-0.60 (-0.67 to -0.52)
Australasia	2.6 (2 to 3.3)	13.1 (10.2 to 16.6)	3.3 (2.6 to 4.1)	12.4 (9.6 to 15.5)	-0.22 (-0.23 to -0.21)
Caribbean	13.8 (10.7 to 17.3)	35.5 (28 to 44.1)	12.5 (9.7 to 15.6)	27.9 (21.3 to 34.9)	-1.15 (-1.25 to -1.05)
Central Asia	27.2 (20.5 to 34)	33.7 (25.9 to 41.8)	20.6 (15.7 to 26.2)	21.9 (16.7 to 27.9)	-2.16 (-2.57 to -1.75)
Central Europe	18.6 (15 to 22.6)	16 (12.7 to 19.5)	11.1 (8.9 to 13.8)	12.1 (9.4 to 15.2)	-1.11 (-1.18 to -1.05)
Central Latin America	65.9 (49.7 to 84.6)	33.1 (25.4 to 41.4)	79.6 (61.1 to 99.3)	32.4 (24.7 to 40.7)	-0.12 (-0.20 to -0.05)
Central Sub-Saharan Africa	471.6 (422.2 to 523.4)	629.1 (562.3 to 699.6)	822 (653.1 to 1003.8)	459 (371.5 to 555.8)	-1.15 (-1.43 to -0.87)
East Asia	800.7 (612.1 to 1040.7)	58.4 (45.4 to 74.8)	738 (576.7 to 920.2)	60.3 (46.9 to 75.5)	0.17 (-0.28 to 0.62)
Eastern Europe	24.4 (18.7 to 31.1)	12 (9.1 to 15.4)	20.2 (15.7 to 25.2)	12.3 (9.3 to 15.8)	-0.36 (-0.82 to 0.10)
Eastern Sub-Saharan Africa	543.9 (443.2 to 664.8)	234.6 (190.5 to 286)	936 (744.3 to 1155.5)	185.6 (148.2 to 228.8)	-0.64 (-0.96 to -0.32)
High-income Asia Pacific	29.8 (23.7 to 37.2)	17.7 (13.9 to 22.1)	23 (18.1 to 28.9)	14.9 (11.7 to 18.6)	-0.62 (-0.65 to -0.59)
High-income North America	36.8 (29.1 to 46.1)	13.7 (10.7 to 17)	44.9 (35.3 to 56.4)	13.5 (10.6 to 16.9)	-0.03 (-0.04 to -0.01)
North Africa and Middle East	282 (221.6 to 347.9)	65.9 (52.4 to 80)	291.1 (232.4 to 357.3)	46.2 (36.7 to 56.7)	-1.80 (-2.03 to -1.57)
Oceania	0.5 (0.4 to 0.6)	7.1 (5.5 to 9.2)	0.6 (0.4 to 0.8)	4.3 (3.3 to 5.6)	-1.92 (-2.23 to -1.62)
South Asia	4509.3 (3672.4 to 5461.2)	334.6 (272 to 407.9)	4255.5 (3275.6 to 5378.8)	211.5 (164.7 to 266.2)	-1.39 (-1.77 to -1.00)
Southeast Asia	381.6 (303.8 to 473.3)	70.6 (56.8 to 87.1)	280 (214.5 to 351.3)	40.3 (31 to 50.5)	-1.94 (-2.16 to -1.72)
Southern Latin America	5.4 (4.2 to 6.8)	10.7 (8.2 to 13.3)	5.2 (4 to 6.6)	8.3 (6.2 to 10.6)	-0.94 (-0.99 to -0.88)
Southern Sub-Saharan Africa	51.6 (45.6 to 57.8)	81.6 (72.4 to 91.6)	45.4 (35.7 to 57.3)	54.2 (42.8 to 68.3)	-1.06 (-1.33 to -0.79)
Tropical Latin America	11.5 (8.4 to 15.1)	7 (5.3 to 9)	13.9 (10.7 to 17.8)	6.3 (4.8 to 8.2)	-0.44 (-0.47 to -0.41)
Western Europe	217.7 (169.8 to 275.1)	62 (48.5 to 77.8)	167.8 (132.6 to 209.5)	49.5 (38.6 to 62.5)	-0.97 (-1.10 to -0.83)
Western Sub-Saharan Africa	214.7 (171.2 to 261.5)	95.4 (77.8 to 115.9)	337.7 (262.3 to 432.9)	64.3 (50.7 to 80.6)	-1.87 (-2.06 to -1.68)

Abbreviation: ASR, age standardized rate; CI, confidence interval; EAPC, estimated annual percentage change; UI, uncertainty interval.

Table S2: The incident cases and age-standardized incidence of iodine deficiency for female in 1990 and 2019, and its temporal trends from 1990 to 2019.

Characteristics	1990		2019		1990–2019
	Incident cases No.×10^4[95% UI]	ASR per 10,0000 No.[95% UI]	Incident cases No.×10^4[95% UI]	ASR per 10,0000 No.[95% UI]	EAPC No.[95% CI]
Global	4466.9 (3658.2 to 5407.2)	152.1 (125 to 183.9)	5130.5 (4124 to 6327.5)	139.8 (112.5 to 171.8)	-0.23 (-0.33 to -0.13)
Socio-demographic index					
High SDI	103.7 (82.3 to 130.6)	26.7 (21.2 to 33.7)	105 (83.9 to 130.5)	25.1 (20 to 31.5)	-0.21 (-0.23 to -0.19)
High-middle SDI	441.7 (355.1 to 545.1)	76 (61.5 to 93.5)	394.3 (312 to 490)	67.8 (53.6 to 84.7)	-0.45 (-0.57 to -0.34)
Middle SDI	999.1 (790 to 1249.8)	101.1 (80.5 to 125.4)	1021.1 (806.5 to 1277)	90.2 (71.3 to 112)	-0.42 (-0.51 to -0.33)
Low-middle SDI	1872.2 (1541.2 to 2270.4)	276.1 (227 to 335.5)	1814.2 (1408.7 to 2282.1)	188.2 (147.2 to 235.7)	-1.26 (-1.52 to -0.99)
Low SDI	1049.2 (880.9 to 1238.6)	323.5 (271.6 to 385.9)	1794.8 (1466.2 to 2181.4)	258.8 (212.1 to 314.6)	-0.79 (-0.91 to -0.68)
Region					
Andean Latin America	1.2 (0.9 to 1.6)	6 (4.7 to 7.7)	1.7 (1.2 to 2.1)	5.2 (3.9 to 6.7)	-0.59 (-0.67 to -0.51)
Australasia	1.5 (1.1 to 1.8)	14.8 (11.6 to 18.7)	1.8 (1.5 to 2.3)	14 (11 to 17.6)	-0.22 (-0.24 to -0.21)
Caribbean	8 (6.2 to 10.1)	40.7 (31.9 to 50.8)	7.1 (5.5 to 8.8)	31.8 (24.2 to 39.5)	-1.17 (-1.27 to -1.07)
Central Asia	24.8 (18.9 to 31)	61.7 (47.4 to 76.6)	18.5 (14.2 to 23.6)	40.2 (30.6 to 51.4)	-2.16 (-2.57 to -1.75)
Central Europe	11.3 (9.1 to 13.9)	19.6 (15.7 to 24)	6.5 (5.2 to 8.1)	14.1 (10.8 to 17.8)	-1.28 (-1.39 to -1.17)
Central Latin America	45.4 (34.4 to 58)	44.8 (34.4 to 56.6)	53.3 (40.8 to 66.5)	43.8 (33.4 to 55.2)	-0.12 (-0.17 to -0.06)
Central Sub-Saharan Africa	309.8 (280.9 to 338.8)	828.2 (747.9 to 911.4)	535.4 (427.5 to 652.2)	597.6 (485.3 to 722.7)	-1.38 (-1.67 to -1.10)
East Asia	488 (370.6 to 631.7)	72.2 (55.6 to 92.6)	504.7 (389.8 to 634.4)	86.4 (66.5 to 109)	0.67 (0.30 to 1.04)
Eastern Europe	18 (14 to 23)	17.8 (13.6 to 22.8)	14.8 (11.6 to 18.3)	18.4 (14.1 to 23.6)	-0.33 (-0.78 to 0.12)
Eastern Sub-Saharan Africa	315.7 (257.1 to 384.9)	268.9 (218.3 to 328.1)	524.6 (419.2 to 654.6)	205.4 (163.8 to 255.2)	-0.84 (-1.10 to -0.57)
High-income Asia Pacific	16.5 (13.1 to 20.6)	19.8 (15.5 to 24.8)	12.7 (10 to 15.9)	16.8 (13.2 to 21.1)	-0.59 (-0.62 to -0.55)
High-income North America	21.1 (16.6 to 26.3)	15.6 (12.2 to 19.6)	25.6 (20.3 to 32.3)	15.6 (12.2 to 19.4)	-0.02 (-0.04 to -0.01)
North Africa and Middle East	172.9 (134.5 to 215)	81.8 (64.4 to 99.6)	177.1 (141.2 to 216.4)	58.3 (46.3 to 71.2)	-1.70 (-1.91 to -1.50)
Oceania	0.3 (0.2 to 0.4)	8.2 (6.3 to 10.6)	0.4 (0.3 to 0.5)	5.3 (4 to 6.9)	-1.70 (-1.99 to -1.42)
South Asia	2458.2 (2004.3 to 3008.6)	383.1 (312.4 to 468.9)	2702.4 (2092.2 to 3418.7)	275 (214.3 to 345.9)	-1.04 (-1.38 to -0.70)
Southeast Asia	256.7 (204.2 to 317.7)	93.6 (75.1 to 115.4)	180 (137.4 to 227.3)	52.4 (40 to 66.1)	-2.00 (-2.21 to -1.80)
Southern Latin America	2.9 (2.3 to 3.7)	11.5 (8.8 to 14.4)	2.8 (2.2 to 3.6)	9 (6.8 to 11.5)	-0.90 (-0.96 to -0.85)
Southern Sub-Saharan Africa	29.9 (26.4 to 33.6)	92.2 (81.4 to 104.1)	26.6 (21 to 33.7)	63.1 (50 to 79.4)	-1.01 (-1.26 to -0.75)
Tropical Latin America	6.4 (4.7 to 8.3)	7.7 (5.9 to 9.9)	7.9 (6.1 to 10.1)	7.2 (5.5 to 9.2)	-0.34 (-0.37 to -0.31)
Western Europe	136.8 (105.9 to 172)	79.3 (62.2 to 99.2)	103 (81.2 to 128.4)	62.9 (49 to 79.4)	-1.00 (-1.16 to -0.84)
Western Sub-Saharan Africa	141.4 (113.7 to 173.6)	122.7 (99.7 to 150.2)	223.8 (172.8 to 285.2)	82.3 (64.8 to 103.5)	-2.00 (-2.25 to -1.75)

Abbreviation: ASR, age standardized rate; CI, confidence interval; EAPC, estimated annual percentage change; UI, uncertainty interval.

Table S3: The incident cases and age-standardized incidence of iodine deficiency for male in 1990 and 2019, and its temporal trends from 1990 to 2019.

Characteristics	1990		2019		1990–2019
	Incident cases No.×10^4[95% UI]	ASR per 10,0000 No.[95% UI]	Incident cases No.×10^4[95% UI]	ASR per 10,0000 No.[95% UI]	EAPC No.[95% CI]
Global	3244.8 (2620.2 to 3932.9)	106.7 (86.8 to 129.4)	2981 (2385.9 to 3715.7)	78.1 (62.6 to 97.1)	-0.77 (-0.95 to -0.58)
Socio-demographic index					
High SDI	72 (56.9 to 90.9)	18 (14.2 to 22.6)	73.8 (58.4 to 92.6)	16.3 (12.9 to 20.4)	-0.37 (-0.39 to -0.36)
High-middle SDI	282.9 (225.7 to 352.1)	47.6 (38.5 to 58.9)	223.3 (178.3 to 276.8)	35.7 (28.4 to 44.7)	-1.02 (-1.20 to -0.83)
Middle SDI	687.1 (542.3 to 859.1)	68 (54.3 to 84.6)	594.8 (470.9 to 746.1)	50.4 (40 to 63.2)	-0.90 (-1.02 to -0.78)
Low-middle SDI	1482.8 (1198.8 to 1788.8)	205.7 (166.9 to 248.8)	1040.7 (815.6 to 1306.6)	106.3 (84 to 133.5)	-2.00 (-2.36 to -1.64)
Low SDI	719.4 (590.8 to 858.4)	212.9 (175.6 to 254.8)	1047.6 (842.4 to 1279.8)	151.5 (122.6 to 185.8)	-0.90 (-1.12 to -0.69)
Region					
Andean Latin America	1 (0.7 to 1.3)	5.1 (3.8 to 6.5)	1.4 (1 to 1.9)	4.4 (3.3 to 5.8)	-0.59 (-0.66 to -0.52)
Australasia	1.1 (0.9 to 1.5)	11.5 (8.9 to 14.6)	1.4 (1.1 to 1.8)	10.8 (8.3 to 13.8)	-0.22 (-0.23 to -0.22)
Caribbean	5.8 (4.5 to 7.4)	30.3 (23.6 to 38.1)	5.4 (4.2 to 6.9)	24.1 (18.4 to 30.7)	-1.11 (-1.21 to -1.01)
Central Asia	2.3 (1.6 to 3.1)	6.2 (4.4 to 8.1)	2.1 (1.5 to 2.8)	4.4 (3.2 to 5.8)	-1.66 (-2.06 to -1.27)
Central Europe	7.3 (5.8 to 9)	12.5 (9.8 to 15.6)	4.6 (3.6 to 5.8)	10.3 (7.8 to 13)	-0.87 (-0.97 to -0.78)
Central Latin America	20.6 (15.3 to 26.5)	21.4 (16.5 to 26.9)	26.3 (20.1 to 33)	21.3 (16.2 to 26.6)	-0.09 (-0.18 to 0.00)
Central Sub-Saharan Africa	161.8 (138 to 186.4)	430.5 (368.1 to 498.4)	286.6 (223.1 to 350.3)	320.5 (255 to 391.2)	-0.69 (-1.01 to -0.38)
East Asia	312.7 (236.6 to 411)	45.4 (35 to 58.7)	233.3 (183.5 to 294.3)	36.4 (28.7 to 45.9)	-0.70 (-1.29 to -0.11)
Eastern Europe	6.4 (4.8 to 8.3)	6.3 (4.7 to 8.2)	5.4 (4.1 to 7)	6.4 (4.8 to 8.4)	-0.39 (-0.87 to 0.10)
Eastern Sub-Saharan Africa	228.2 (184 to 279.4)	198.6 (160 to 242.1)	411.5 (326.3 to 508.8)	165.4 (131.7 to 204.6)	-0.37 (-0.79 to 0.04)
High-income Asia Pacific	13.4 (10.6 to 16.7)	15.7 (12.4 to 19.6)	10.3 (8.1 to 13)	13.1 (10.2 to 16.4)	-0.65 (-0.68 to -0.62)
High-income North America	15.7 (12.3 to 19.8)	11.7 (9.1 to 14.8)	19.2 (15 to 24.3)	11.5 (9 to 14.7)	-0.03 (-0.05 to -0.01)
North Africa and Middle East	109.1 (85.8 to 133.7)	50.8 (40.8 to 61.7)	114 (90.7 to 140.3)	35 (27.7 to 43)	-1.92 (-2.18 to -1.66)
Oceania	0.2 (0.2 to 0.3)	6.1 (4.6 to 7.9)	0.2 (0.2 to 0.3)	3.4 (2.5 to 4.4)	-2.22 (-2.56 to -1.88)
South Asia	2051 (1646.4 to 2497.8)	289 (232.5 to 351.5)	1553.1 (1200.5 to 1967.7)	151.3 (118.1 to 190.3)	-1.92 (-2.36 to -1.47)
Southeast Asia	124.8 (98.2 to 157)	47.3 (37.8 to 59)	100 (77.2 to 126)	28.5 (22 to 35.9)	-1.76 (-2.01 to -1.50)
Southern Latin America	2.5 (1.9 to 3.2)	9.9 (7.6 to 12.5)	2.4 (1.8 to 3)	7.6 (5.7 to 9.8)	-0.98 (-1.03 to -0.93)
Southern Sub-Saharan Africa	21.7 (19.2 to 24.3)	70.6 (62.8 to 79.3)	18.8 (14.8 to 24)	45.3 (36 to 57.2)	-1.11 (-1.41 to -0.81)
Tropical Latin America	5.1 (3.7 to 6.6)	6.3 (4.8 to 8)	6 (4.5 to 7.7)	5.5 (4.1 to 7.1)	-0.55 (-0.59 to -0.52)
Western Europe	80.9 (63.5 to 102.1)	45.2 (35.6 to 57.3)	64.8 (51.5 to 81.1)	36.7 (28.8 to 46.7)	-0.90 (-1.00 to -0.79)
Western Sub-Saharan Africa	73.2 (58.5 to 89.3)	66.2 (54.1 to 79.6)	113.9 (87.1 to 147.1)	45 (35.6 to 57)	-1.60 (-1.70 to -1.51)

Abbreviation: ASR, age standardized rate; CI, confidence interval; EAPC, estimated annual percentage change; UI, uncertainty interval.

Table S4: The incident cases and age-standardized incidence of Vitamin A deficiency for both gender in 1990 and 2019, and its temporal trends from 1990 to 2019.

Characteristics	1990		2019		1990–2019
	Incident cases No.$\times 10^4$[95% UI]	ASR per 10,0000 No.[95% UI]	Incident cases No.$\times 10^4$[95% UI]	ASR per 10,0000 No.[95% UI]	EAPC No.[95% CI]
Global	877376.3 (840347 to 914976.5)	17323.2 (16526.5 to 18138.9)	489662.7 (469006.4 to 512234.3)	6955.6 (6645.9 to 7294.2)	-3.11 (-3.25 to -2.98)
Socio-demographic index					
High SDI	9794.3 (9326.5 to 10277)	1338.2 (1268.4 to 1413.8)	5241 (4970.4 to 5538)	586.7 (550.4 to 625.8)	-2.69 (-2.78 to -2.61)
High-middle SDI	87803 (81996.4 to 94985.8)	7635 (7130.2 to 8221.9)	34095.8 (32378.4 to 35977.9)	2624.5 (2484.5 to 2776.4)	-3.71 (-3.81 to -3.62)
Middle SDI	241274.2 (226843.9 to 257287.3)	12892.4 (12128.3 to 13703.9)	83001.5 (78166 to 88264.9)	3679 (3464.1 to 3921.2)	-4.16 (-4.25 to -4.07)
Low-middle SDI	324236.7 (304415.3 to 343115.1)	26646.4 (25022.2 to 28112.3)	151479.7 (140880.9 to 163820.3)	8475.9 (7893.2 to 9132.4)	-3.95 (-4.13 to -3.76)
Low SDI	213837 (207854.1 to 219746.7)	37932.6 (36877.7 to 38931.6)	215520.1 (207408.8 to 223699.9)	18004.6 (17394.9 to 18637.1)	-2.58 (-2.80 to -2.36)
Region					
Andean Latin America	4726.9 (4307 to 5191)	12877.7 (11631.9 to 14260.4)	3539.9 (3189.4 to 3925.2)	5904.7 (5259.4 to 6633.2)	-2.75 (-2.92 to -2.58)
Australasia	51.2 (45.8 to 57.2)	237.4 (210.4 to 270)	44.5 (40 to 49.9)	148.8 (131.4 to 168.8)	-1.07 (-1.35 to -0.78)
Caribbean	3768 (3500.2 to 4019.7)	11146.7 (10301.1 to 11991.6)	2775.2 (2525.9 to 3047.6)	6289.7 (5659.2 to 6996.4)	-2.21 (-2.33 to -2.09)
Central Asia	6011.1 (5510.7 to 6558.2)	8840.3 (8009.4 to 9737.2)	3909.5 (3588.2 to 4242.9)	4272 (3883.1 to 4669.6)	-2.45 (-2.67 to -2.24)
Central Europe	17873.5 (17051.2 to 18801.8)	15274 (14477.1 to 16139.9)	8183.3 (7774.9 to 8604.4)	7477.4 (7089.5 to 7916.5)	-2.59 (-2.68 to -2.50)
Central Latin America	23087.7 (21042.4 to 25189)	14629.6 (13257.7 to 16046.8)	13543.1 (12221.5 to 15011.3)	5717.6 (5096.1 to 6419.5)	-3.29 (-3.35 to -3.23)
Central Sub-Saharan Africa	23744 (22119.9 to 25331.8)	43280.4 (39834.2 to 46536.7)	33739.9 (30648 to 37138.4)	25905.2 (23288.4 to 28883)	-1.37 (-1.85 to -0.89)
East Asia	126330.1 (107266.1 to 149469.4)	11210.8 (9382.3 to 13316.5)	26963.6 (23063.4 to 31672)	2183.6 (1847.1 to 2605)	-5.61 (-5.81 to -5.42)
Eastern Europe	2402.5 (2215.4 to 2613)	1071.3 (979.5 to 1179.1)	1125.5 (1028 to 1232.6)	530.9 (481.6 to 587)	-2.45 (-2.62 to -2.29)
Eastern Sub-Saharan Africa	90132.6 (87687.6 to 92711.8)	46770.6 (45268.6 to 48255.9)	98037.3 (93560.5 to 103044)	23500 (22337.2 to 24765.1)	-2.46 (-2.66 to -2.25)
High-income Asia Pacific	2079.6 (1834.6 to 2362.1)	1376.9 (1194.4 to 1589.9)	1051.2 (937.5 to 1169.9)	683.9 (599 to 780)	-2.18 (-2.29 to -2.08)
High-income North America	2002.8 (1760.9 to 2250.8)	811 (703.8 to 924.9)	1518.5 (1307.5 to 1751.1)	485.6 (408.1 to 574)	-1.97 (-2.13 to -1.81)
North Africa and Middle East	53157.2 (51155.5 to 55198.6)	15427.7 (14749.2 to 16089.9)	30635.7 (28841.8 to 32427.9)	5249.9 (4905.9 to 5602.5)	-3.70 (-3.82 to -3.59)
Oceania	1247.9 (1137.4 to 1361.3)	19889.7 (18034.3 to 21935.5)	1639.1 (1449.2 to 1859.5)	13011.6 (11381.7 to 14879.8)	-1.01 (-1.31 to -0.70)
South Asia	299295.4 (270965.1 to 327585.2)	27177.4 (24364.6 to 30028.7)	127933.1 (111031.9 to 147729)	7189.3 (6181.3 to 8389)	-4.53 (-4.81 to -4.26)
Southeast Asia	95994.3 (89960.3 to 102323.5)	21792.8 (20300.6 to 23360.3)	30586.3 (27796.3 to 33573.1)	5175.3 (4668 to 5733.8)	-4.64 (-4.77 to -4.51)
Southern Latin America	5004.6 (4495.5 to 5594.3)	10805.2 (9611.4 to 12213.7)	4164.4 (3690.5 to 4705.3)	6672.8 (5811.8 to 7650.3)	-1.38 (-1.60 to -1.17)
Southern Sub-Saharan Africa	8877.2 (8154.3 to 9689.8)	18153.7 (16488.6 to 20003.7)	5653.1 (5108.5 to 6241.5)	7834.6 (7001 to 8690.2)	-2.69 (-2.82 to -2.55)
Tropical Latin America	37010.9 (33332.8 to 41157.7)	24605.7 (22010.2 to 27545.3)	21933.1 (19097 to 25162.3)	10005.4 (8592.7 to 11600.5)	-3.15 (-3.25 to -3.06)
Western Europe	5145 (4841.7 to 5477.9)	1408.6 (1320.3 to 1508.6)	2792.2 (2610.5 to 2974.5)	683.2 (637.3 to 735.6)	-2.16 (-2.46 to -1.85)
Western Sub-Saharan Africa	69433.8 (67269.1 to 71819.6)	36703.6 (35417.5 to 38048.5)	69894.2 (66893.7 to 72944.4)	15570.9 (14825.2 to 16315.9)	-2.97 (-3.07 to -2.87)

Abbreviation: ASR, age standardized rate; CI, confidence interval; EAPC, estimated annual percentage change; UI, uncertainty interval.

Table S5: The incident cases and age-standardized incidence of Vitamin A deficiency for female in 1990 and 2019, and its temporal trends from 1990 to 2019.

Characteristics	1990		2019		1990–2019
	Incident cases No.×10^4[95% UI]	ASR per 10,0000 No.[95% UI]	Incident cases No.×10^4[95% UI]	ASR per 10,0000 No.[95% UI]	EAPC No.[95% CI]
Global	336074.1 (322087 to 351326.5)	13456.5 (12848.5 to 14141.4)	208933.1 (199953.4 to 218963.7)	5999.1 (5719 to 6307.3)	-2.75 (-2.93 to -2.56)
Socio-demographic index					
High SDI	5565 (5247.8 to 5898.2)	1489.9 (1395.9 to 1591.9)	3308.5 (3083.2 to 3538)	734.8 (677.3 to 795.2)	-2.26 (-2.35 to -2.18)
High-middle SDI	38000.2 (35934.5 to 40264.2)	6636.7 (6280.9 to 7037.2)	17650.3 (16671.7 to 18705)	2698.5 (2539.8 to 2873.9)	-3.17 (-3.31 to -3.02)
Middle SDI	90408.5 (85105.7 to 96406.1)	9883.6 (9321.4 to 10498.8)	37792.7 (35382.8 to 40371.4)	3411.7 (3179.1 to 3649.9)	-3.53 (-3.68 to -3.38)
Low-middle SDI	119998.9 (111953.9 to 128813.1)	19768.3 (18554.5 to 21100.6)	63293.7 (58349.9 to 68425.6)	7147 (6594.1 to 7719.6)	-3.48 (-3.69 to -3.28)
Low SDI	81913.6 (79020.1 to 85042.6)	28510.4 (27582.1 to 29549.7)	86739.9 (83012.8 to 90994.3)	14190.3 (13655.9 to 14821.7)	-2.41 (-2.67 to -2.14)
Region					
Andean Latin America	2203.6 (1992.8 to 2435.4)	11794.1 (10619.7 to 13219)	1761.9 (1575.7 to 1958)	5826.2 (5127 to 6585.2)	-2.41 (-2.56 to -2.27)
Australasia	32.7 (28.5 to 37.4)	299.1 (256.4 to 347.9)	28.6 (24.9 to 32.8)	187.2 (160.7 to 218.1)	-0.99 (-1.29 to -0.69)
Caribbean	1746.1 (1603.9 to 1896.1)	10195.5 (9260.8 to 11213.3)	1373.6 (1220.9 to 1541.3)	6143.7 (5393.5 to 6988.5)	-1.92 (-2.01 to -1.82)
Central Asia	2083.5 (1896.1 to 2283.7)	5991.8 (5383 to 6654)	1575.2 (1432.9 to 1725.8)	3436.5 (3088.3 to 3801.3)	-1.78 (-2.01 to -1.54)
Central Europe	10937.8 (10276.6 to 11605)	17815.8 (16643.3 to 19015.9)	5517.1 (5197.4 to 5863.4)	9559.5 (8967.4 to 10190.8)	-2.21 (-2.27 to -2.14)
Central Latin America	9153.2 (8251.7 to 10066)	11435.8 (10193.7 to 12694.5)	5737.8 (5150.3 to 6364.6)	4715.7 (4182.7 to 5299)	-3.09 (-3.15 to -3.03)
Central Sub-Saharan Africa	8525.8 (7641.4 to 9411.3)	30954.4 (27410.7 to 34679.4)	12057.6 (10566.9 to 13867.2)	18608.1 (16016.3 to 21566.6)	-1.29 (-1.86 to -0.72)
East Asia	40533.5 (33926.4 to 47827.5)	7423.4 (6146.9 to 8816.1)	12108.8 (10138.8 to 14456.7)	2002 (1664.7 to 2413.3)	-4.71 (-5.07 to -4.35)
Eastern Europe	1454.3 (1321.3 to 1606.2)	1205.5 (1085 to 1343)	773.2 (690.5 to 857.3)	677.5 (595.3 to 759.8)	-2.00 (-2.19 to -1.81)
Eastern Sub-Saharan Africa	38958.3 (37393.1 to 40539.7)	40324 (38557.9 to 42134.5)	43191.3 (40743.5 to 45880.4)	20731.4 (19414.7 to 22180.9)	-2.35 (-2.57 to -2.13)
High-income Asia Pacific	1156.3 (1007.9 to 1330.8)	1510.9 (1288.9 to 1767.4)	595.2 (521 to 675.6)	760.4 (650.6 to 881.3)	-2.03 (-2.17 to -1.89)
High-income North America	1473.3 (1263.2 to 1723.8)	1169.9 (982.1 to 1388.5)	1139.3 (958.3 to 1345.9)	716.4 (584.4 to 870.2)	-1.76 (-1.84 to -1.68)
North Africa and Middle East	21831.9 (20774.6 to 22897.5)	12965 (12263.2 to 13659.8)	13019.3 (12091 to 13978.5)	4629.4 (4269.6 to 5018.3)	-3.52 (-3.64 to -3.40)
Oceania	497.7 (444.3 to 562.7)	16367.1 (14428.1 to 18666.3)	673.7 (578.2 to 782.3)	10961.2 (9360.2 to 12903)	-0.96 (-1.24 to -0.68)
South Asia	104837.7 (93097.5 to 117538.6)	19979.2 (17518.4 to 22725.9)	51734.4 (44395.3 to 60153.4)	6030.2 (5078.8 to 7113.7)	-4.08 (-4.37 to -3.78)
Southeast Asia	42020.8 (39060.9 to 45146.3)	18820.8 (17345.5 to 20365.4)	15378.1 (13837.1 to 17260.6)	5149.6 (4597.2 to 5819.6)	-4.15 (-4.28 to -4.03)
Southern Latin America	2099.8 (1839.1 to 2375.1)	8884.7 (7669.6 to 10213.5)	1773.2 (1530.2 to 2037.3)	5562 (4714.8 to 6528.6)	-1.33 (-1.52 to -1.13)
Southern Sub-Saharan Africa	3885.6 (3490.4 to 4314.5)	15333.6 (13664 to 17226.4)	2863.7 (2551.6 to 3215.5)	7736.1 (6803.8 to 8807)	-2.08 (-2.24 to -1.92)
Tropical Latin America	16481.8 (14452.7 to 18681.9)	21728.5 (18780.3 to 24883.5)	10127.5 (8626.7 to 11816)	9082.5 (7675.5 to 10774.2)	-3.00 (-3.06 to -2.94)
Western Europe	2404.1 (2239 to 2578.9)	1301 (1205.6 to 1408.1)	1346.4 (1256.6 to 1452.5)	657.6 (611.9 to 713.6)	-1.92 (-2.23 to -1.60)
Western Sub-Saharan Africa	23756.4 (22633 to 25017.3)	25122 (23773.8 to 26525.1)	26157 (24602 to 27913.5)	11458.8 (10713.9 to 12285)	-2.63 (-2.92 to -2.33)

Abbreviation: ASR, age standardized rate; CI, confidence interval; EAPC, estimated annual percentage change; UI, uncertainty interval.

Table S6: The incident cases and age-standardized incidence of Vitamin A deficiency for male in 1990 and 2019, and its temporal trends from 1990 to 2019.

Characteristics	1990		2019		1990–2019
	Incident cases No.×10^4[95% UI]	ASR per 10,0000 No.[95% UI]	Incident cases No.×10^4[95% UI]	ASR per 10,0000 No.[95% UI]	EAPC No.[95% CI]
Global	541302.2 (509180.6 to 576528.3)	21073.8 (19711.5 to 22557.3)	280729.6 (263694.4 to 300226.4)	7886.2 (7367.7 to 8489.8)	-3.36 (-3.46 to -3.26)
Socio-demographic index					
High SDI	4229.3 (3880.7 to 4591.8)	1188.9 (1081.9 to 1305.9)	1932.5 (1778.5 to 2101)	444.2 (405 to 490.9)	-3.28 (-3.36 to -3.19)
High-middle SDI	49802.9 (44426.3 to 56126.6)	8566.8 (7650.8 to 9577.2)	16445.4 (15061.8 to 18049.2)	2543.4 (2316 to 2808.2)	-4.19 (-4.26 to -4.13)
Middle SDI	150865.7 (137514.2 to 165820.1)	15781.8 (14436.2 to 17265.9)	45208.8 (41051 to 49625.3)	3935.4 (3559.3 to 4327)	-4.60 (-4.67 to -4.54)
Low-middle SDI	204237.8 (187942.1 to 220649.7)	33272.9 (30685 to 35802)	88186 (78783.6 to 98680.2)	9798.7 (8762.9 to 10925.6)	-4.22 (-4.39 to -4.05)
Low SDI	131923.4 (126531.8 to 137039.9)	47234.5 (45411.8 to 49114.2)	128780.2 (122026.5 to 135727.9)	21850.9 (20800.4 to 23005.1)	-2.67 (-2.86 to -2.48)
Region					
Andean Latin America	2523.4 (2152.6 to 2962.4)	13969.8 (11715 to 16551.5)	1778 (1487.9 to 2109.7)	5982.1 (4917.6 to 7161.1)	-3.06 (-3.26 to -2.86)
Australasia	18.5 (15.4 to 22.4)	176.6 (142.8 to 219.8)	15.9 (13.2 to 19.2)	110.3 (89.1 to 138)	-1.20 (-1.48 to -0.92)
Caribbean	2021.8 (1796.6 to 2244.7)	12116.8 (10659.2 to 13592.4)	1401.6 (1202.6 to 1618.3)	6437.1 (5398.4 to 7575.1)	-2.48 (-2.63 to -2.34)
Central Asia	3927.6 (3452.5 to 4437.7)	11717.5 (10127.4 to 13416)	2334.3 (2070.4 to 2649.1)	5097.2 (4453.4 to 5858.4)	-2.87 (-3.08 to -2.65)
Central Europe	6935.7 (6357.8 to 7532)	12734.6 (11590.2 to 13940.9)	2666.2 (2440.2 to 2895.8)	5416.6 (4920.4 to 5941.3)	-3.17 (-3.31 to -3.02)
Central Latin America	13934.6 (12134.3 to 15952.6)	17886.2 (15506.4 to 20729.7)	7805.3 (6639.4 to 9167.7)	6745.2 (5633.2 to 8031.7)	-3.43 (-3.50 to -3.37)
Central Sub-Saharan Africa	15218.2 (13878.4 to 16589.2)	55764.1 (50095.4 to 61429.2)	21682.3 (18814.6 to 24715.3)	33242.3 (28347.2 to 38656.5)	-1.42 (-1.86 to -0.99)
East Asia	85796.6 (68140 to 105989.5)	14751.9 (11542.1 to 18376.7)	14854.7 (11539.3 to 18943)	2354.1 (1782.9 to 3037)	-6.18 (-6.30 to -6.06)
Eastern Europe	948.2 (812.5 to 1100)	930.5 (786.2 to 1093.9)	352.3 (301.3 to 408.3)	376.5 (318.9 to 445.8)	-3.20 (-3.34 to -3.06)
Eastern Sub-Saharan Africa	51174.3 (49247.6 to 53205)	53317.5 (50965.4 to 55740.1)	54846.1 (51452.5 to 58934.2)	26294.4 (24445.6 to 28470.7)	-2.54 (-2.74 to -2.34)
High-income Asia Pacific	923.3 (732.3 to 1160.3)	1245.1 (959.5 to 1582.3)	456 (374.8 to 551.5)	609.8 (482.2 to 763.7)	-2.35 (-2.43 to -2.27)
High-income North America	529.5 (419.2 to 652.4)	451.9 (348.7 to 574.5)	379.2 (294.9 to 495.3)	255.4 (189.1 to 346.7)	-2.47 (-2.85 to -2.10)
North Africa and Middle East	31325.3 (29572.7 to 33224.2)	17769.4 (16671.7 to 18948.1)	17616.4 (16138.5 to 19121.2)	5823.3 (5263.1 to 6382.5)	-3.85 (-3.97 to -3.73)
Oceania	750.2 (659.8 to 848.7)	23171.1 (20155 to 26483.4)	965.4 (804.1 to 1164.7)	14936 (12314 to 18268.9)	-1.04 (-1.36 to -0.71)
South Asia	194457.7 (170473.5 to 219866.8)	33837.4 (29298.2 to 38804.2)	76198.6 (61400.3 to 93009.6)	8290.6 (6606.1 to 10273.1)	-4.79 (-5.06 to -4.52)
Southeast Asia	53973.5 (48558.8 to 59295.9)	24772.8 (22088.2 to 27316.5)	15208.2 (12839.1 to 17794.1)	5200.5 (4354.9 to 6148.6)	-5.06 (-5.22 to -4.90)
Southern Latin America	2904.8 (2452 to 3407.5)	12758.5 (10704.8 to 15239.4)	2391.2 (1955.9 to 2876.4)	7799.4 (6279.5 to 9533.8)	-1.42 (-1.66 to -1.19)
Southern Sub-Saharan Africa	4991.6 (4344.5 to 5647.3)	21116 (18116.9 to 24134.3)	2789.4 (2340 to 3266)	7935.2 (6538.6 to 9451)	-3.23 (-3.36 to -3.09)
Tropical Latin America	20529.1 (17517.4 to 24136)	27517.5 (23159.4 to 32660.6)	11805.6 (9567.2 to 14675)	10945.1 (8589 to 13844)	-3.28 (-3.40 to -3.15)
Western Europe	2741 (2499.5 to 3012.2)	1515.8 (1370.2 to 1677.8)	1445.8 (1294.6 to 1608.9)	708.6 (633 to 796.6)	-2.37 (-2.67 to -2.07)
Western Sub-Saharan Africa	45677.4 (43892.6 to 47665)	48571.7 (46442.6 to 50841.5)	43737.2 (41355.8 to 46335.6)	19857.7 (18670.8 to 21193.1)	-3.15 (-3.20 to -3.10)

Abbreviation: ASR, age standardized rate; CI, confidence interval; EAPC, estimated annual percentage change; UI, uncertainty interval.

Table S7: The incident cases and age-standardized incidence of protein-energy malnutrition for both gender in 1990 and 2019, and its temporal trends from 1990 to 2019.

Characteristics	1990		2019		1990–2019
	Incident cases No.$\times 10^4$[95% UI]	ASR per 10,0000 No.[95% UI]	Incident cases No.$\times 10^4$[95% UI]	ASR per 10,0000 No.[95% UI]	EAPC No.[95% CI]
Global	111389.2 (91268.4 to 136380.2)	1896.6 (1563.7 to 2293.1)	154086 (128445.2 to 183279)	2099.4 (1752.8 to 2487.4)	-0.03 (-0.19 to 0.13)
Socio-demographic index					
High SDI	7100.8 (5811 to 8643.7)	889.7 (729.2 to 1080.6)	10667 (8616.8 to 12940.6)	1042.1 (847 to 1278.1)	0.20 (0.07 to 0.33)
High-middle SDI	11046.3 (9278.3 to 13395.6)	1365.9 (1091.9 to 1696.3)	21676.4 (17616 to 26405.8)	1827.9 (1489.9 to 2234.5)	0.82 (0.67 to 0.97)
Middle SDI	23131.8 (19993.5 to 27238.3)	1839.8 (1491.3 to 2265)	42326.1 (35473.9 to 51000.1)	2153.1 (1778.8 to 2566.2)	0.26 (0.12 to 0.41)
Low-middle SDI	26240.9 (23968.7 to 29344.6)	2600.2 (2170 to 3101)	35288 (30556 to 41970.7)	2560.8 (2142.3 to 3030.9)	-0.61 (-0.79 to -0.43)
Low SDI	12000.6 (11247.4 to 13000.8)	2245.6 (1901.6 to 2655.5)	20010 (18101.4 to 22485.4)	1948.3 (1670.5 to 2280.6)	-0.91 (-1.08 to -0.74)
Region					
Andean Latin America	273.8 (239 to 311.8)	716.1 (630 to 807.1)	374.5 (329.7 to 428.1)	601.5 (529.7 to 687.2)	-1.17 (-1.34 to -1.00)
Australasia	99 (82.3 to 119.4)	535.4 (453.8 to 634.3)	163.2 (135.7 to 195.9)	601 (511 to 709.8)	0.63 (0.43 to 0.83)
Caribbean	389.6 (320.2 to 471.1)	1030.7 (858.4 to 1225.8)	410.2 (344.6 to 487.1)	921.9 (771.8 to 1094.7)	-0.78 (-0.92 to -0.63)
Central Asia	676.4 (570 to 818.5)	783.2 (662.2 to 942.4)	781.6 (652.6 to 938.9)	837.7 (697.3 to 1013.6)	-0.08 (-0.17 to 0.02)
Central Europe	876.2 (683.3 to 1101.9)	865.6 (674.1 to 1105)	933 (742.6 to 1160.4)	1041.8 (812.2 to 1327.1)	0.46 (0.36 to 0.56)
Central Latin America	2273.8 (1906.6 to 2695.4)	1408.4 (1186.4 to 1646.3)	2896 (2400.9 to 3450.3)	1192.7 (992.7 to 1415.5)	-1.02 (-1.27 to -0.77)
Central Sub-Saharan Africa	1384.1 (1065.4 to 1795.8)	1480.5 (1217.2 to 1795.8)	1924.5 (1568.6 to 2363.2)	1131.2 (952.3 to 1335.9)	-1.18 (-1.46 to -0.91)
East Asia	19345.8 (14525.5 to 25587)	1623.2 (1221.1 to 2138)	28603.7 (22474 to 35618.1)	1991.1 (1566.1 to 2492.8)	0.51 (0.34 to 0.68)
Eastern Europe	1574.5 (1285.4 to 1910.6)	860.7 (701 to 1061.8)	1518.9 (1201.9 to 1877)	995.4 (790 to 1249.4)	0.17 (0.03 to 0.31)
Eastern Sub-Saharan Africa	4569.2 (3662.9 to 5775.7)	1628.7 (1387.4 to 1937.3)	6206.6 (5191.5 to 7441.1)	1221.5 (1049.2 to 1424.1)	-1.31 (-1.43 to -1.19)
High-income Asia Pacific	1118.9 (932 to 1353.9)	740.9 (632.2 to 877.8)	1300.7 (1052.8 to 1590.7)	800 (674.3 to 956.2)	-0.19 (-0.37 to 0.00)
High-income North America	1918.1 (1508.8 to 2405.3)	670.8 (529.5 to 843)	3027.2 (2374.9 to 3731.7)	779 (615.2 to 966.1)	-0.08 (-0.28 to 0.11)
North Africa and Middle East	6110.7 (5016.5 to 7500.9)	1334.6 (1115.9 to 1616.6)	9068.2 (7793.2 to 10559.2)	1521 (1301.7 to 1773.7)	0.12 (-0.04 to 0.28)
Oceania	145.5 (118.8 to 182.1)	1778.2 (1483.8 to 2139.8)	267 (214.3 to 331)	1671.1 (1390.8 to 2008.3)	-0.39 (-0.66 to -0.11)
South Asia	46504.9 (38558.2 to 56361.4)	3286.7 (2736.7 to 3931.1)	62125.9 (51085.7 to 74408.9)	3599.7 (2964.1 to 4309.3)	-0.23 (-0.39 to -0.08)
Southeast Asia	12686.6 (10496.6 to 15363.5)	2550.5 (2130 to 3032.6)	16533.1 (13928.3 to 19273.7)	2615.6 (2213.7 to 3056.3)	-0.21 (-0.34 to -0.08)
Southern Latin America	353.1 (298.9 to 417.5)	719.5 (608.2 to 851.2)	599.8 (496 to 722.8)	902.3 (753.1 to 1080.6)	0.27 (0.03 to 0.51)
Southern Sub-Saharan Africa	669.5 (556 to 824.6)	1132 (955.7 to 1364.2)	797 (669.9 to 944.6)	1023.5 (860 to 1208.2)	-0.78 (-0.92 to -0.64)
Tropical Latin America	1198.9 (975.3 to 1474.4)	797.1 (652.3 to 969.9)	1426 (1194.5 to 1699.8)	691.7 (584.4 to 812.9)	-1.30 (-1.54 to -1.05)
Western Europe	4222 (3426.2 to 5241.6)	1116.1 (905.8 to 1383.2)	6054.2 (4900.5 to 7379.3)	1315 (1071.4 to 1622.5)	0.35 (0.21 to 0.49)
Western Sub-Saharan Africa	4998.5 (4027.9 to 6309)	1652.9 (1391.6 to 1966.9)	9074.8 (7836.5 to 10661.4)	1515.6 (1316.8 to 1746.9)	-0.60 (-0.79 to -0.42)

Abbreviation: ASR, age standardized rate; CI, confidence interval; EAPC, estimated annual percentage change; UI, uncertainty interval.

Table S8: The incident cases and age-standardized incidence of protein-energy malnutrition for female in 1990 and 2019, and its temporal trends from 1990 to 2019.

Characteristics	1990		2019		1990–2019
	Incident cases No.×10^4[95% UI]	ASR per 10,0000 No.[95% UI]	Incident cases No.×10^4[95% UI]	ASR per 10,0000 No.[95% UI]	EAPC No.[95% CI]
Global	51625.2 (41920.1 to 63990.1)	1781.6 (1456.6 to 2184.4)	68206 (56809.7 to 80628.8)	1894.6 (1583.6 to 2246.7)	-0.23 (-0.42 to -0.04)
Socio-demographic index					
High SDI	3506.7 (2870.4 to 4268.2)	863.6 (707.1 to 1048.8)	4917.1 (3962.2 to 5973.1)	960.7 (783.4 to 1176.5)	-0.02 (-0.16 to 0.12)
High-middle SDI	4743.7 (3990.9 to 5727.9)	1247.3 (989.3 to 1567)	8152.9 (6637.7 to 9948.6)	1469.8 (1193.9 to 1800.3)	0.34 (0.13 to 0.54)
Middle SDI	10397.9 (9014 to 12214.8)	1769 (1423.8 to 2207.6)	18481.3 (15440.6 to 22404.7)	1960.3 (1622.4 to 2344.9)	0.04 (-0.13 to 0.22)
Low-middle SDI	11343.6 (10385.5 to 12581)	2397.6 (1982.8 to 2915)	15522.4 (13389.2 to 18314.9)	2398.5 (2002.3 to 2834.4)	-0.71 (-0.94 to -0.47)
Low SDI	5368.2 (5055.8 to 5757.2)	2069.9 (1745.6 to 2466.9)	9039.8 (8255.8 to 10079.4)	1797.7 (1544.6 to 2084.8)	-0.94 (-1.12 to -0.76)
Region					
Andean Latin America	144.7 (125.7 to 166.6)	760.4 (664.9 to 867.8)	190.3 (167.2 to 216.1)	608 (534.3 to 689.8)	-1.47 (-1.69 to -1.26)
Australasia	52.4 (42 to 64.4)	555.4 (455.1 to 676.4)	94.9 (76.6 to 117.2)	669 (555.6 to 818.9)	0.46 (0.30 to 0.62)
Caribbean	188.8 (154.2 to 229)	994.9 (824.3 to 1189.6)	203.1 (169.8 to 242)	913.2 (760.4 to 1093.1)	-0.74 (-0.89 to -0.59)
Central Asia	337 (273.7 to 419.5)	788.3 (646.9 to 967.5)	397 (328.2 to 489.1)	868.4 (714.1 to 1074.7)	0.03 (-0.07 to 0.12)
Central Europe	444.5 (342.2 to 565.4)	888.7 (678.8 to 1154.7)	443.9 (347.6 to 558)	1006.5 (763.3 to 1317)	0.16 (0.07 to 0.26)
Central Latin America	1186.8 (979.6 to 1439.3)	1436 (1197.2 to 1699.7)	1398.2 (1150.5 to 1685.3)	1132 (940.5 to 1360.6)	-1.23 (-1.36 to -1.09)
Central Sub-Saharan Africa	825.9 (578 to 1155)	1697 (1288.1 to 2215.4)	1131.4 (858.6 to 1474.8)	1326.8 (1051.5 to 1653.1)	-1.10 (-1.41 to -0.80)
East Asia	9117.5 (6743.2 to 12219.5)	1599.1 (1185.4 to 2143.9)	10388.4 (8169.9 to 13008.8)	1542.5 (1208 to 1949.5)	-0.35 (-0.63 to -0.08)
Eastern Europe	725.9 (591.3 to 886.5)	807.6 (651.2 to 1010.2)	708.1 (553.4 to 882.8)	938.9 (735 to 1191.4)	0.17 (0.01 to 0.33)
Eastern Sub-Saharan Africa	2204.9 (1749.7 to 2802.1)	1492.4 (1251.1 to 1793.3)	3039.5 (2565.6 to 3603.8)	1177.4 (1017.8 to 1368.6)	-1.19 (-1.29 to -1.08)
High-income Asia Pacific	547.1 (455.4 to 661.4)	728.1 (621.8 to 853.8)	593.3 (474.1 to 728)	744.1 (630.9 to 882.5)	-0.41 (-0.58 to -0.25)
High-income North America	904.9 (713 to 1144.9)	617.7 (485.2 to 781.6)	1423.7 (1124.6 to 1767.7)	714.5 (565.6 to 895.1)	-0.11 (-0.31 to 0.09)
North Africa and Middle East	2916.8 (2349.1 to 3649.7)	1297.3 (1077.7 to 1584.9)	4159.2 (3593.1 to 4838.2)	1451.2 (1254.1 to 1686.6)	0.17 (0.01 to 0.32)
Oceania	77.2 (60.6 to 100.9)	1981.4 (1617.7 to 2469.3)	123 (93.3 to 158.9)	1613.2 (1281 to 2017.7)	-0.97 (-1.16 to -0.77)
South Asia	20819.3 (17089.6 to 25736.9)	3027 (2510.2 to 3652.3)	28441.9 (23360.8 to 34043.7)	3427.8 (2828.8 to 4111.8)	-0.31 (-0.55 to -0.08)
Southeast Asia	5836.6 (4740.9 to 7256.7)	2295.7 (1887 to 2792.6)	7601.9 (6425.2 to 8938.3)	2422.2 (2061.9 to 2845.8)	-0.07 (-0.21 to 0.07)
Southern Latin America	161.4 (134.1 to 192.3)	642.9 (534.3 to 764.9)	273.6 (224.5 to 333.6)	807.4 (669 to 979.7)	0.32 (0.05 to 0.58)
Southern Sub-Saharan Africa	309.8 (266.1 to 367.3)	1002.8 (866.5 to 1173.7)	329.5 (283.8 to 386.9)	832.1 (715.7 to 979.6)	-0.87 (-1.01 to -0.73)
Tropical Latin America	626.8 (496.1 to 791.8)	817.7 (650.4 to 1021.4)	714.1 (596 to 850.2)	689.7 (584.2 to 813.4)	-1.22 (-1.46 to -0.98)
Western Europe	2080.8 (1688.3 to 2576.6)	1056.6 (857 to 1317.9)	2872.8 (2315.8 to 3501.8)	1213.8 (989.1 to 1484.8)	0.22 (0.08 to 0.36)
Western Sub-Saharan Africa	2116.1 (1709.4 to 2681.3)	1357.3 (1150.8 to 1619.8)	3678.5 (3279.4 to 4190.9)	1193.6 (1064.3 to 1347.8)	-0.68 (-0.83 to -0.52)

Abbreviation: ASR, age standardized rate; CI, confidence interval; EAPC, estimated annual percentage change; UI, uncertainty interval.

Table S9: The incident cases and age-standardized incidence of protein-energy malnutrition for male in 1990 and 2019, and its temporal trends from 1990 to 2019.

Characteristics	1990		2019		1990–2019
	Incident cases No.$\times 10^4$[95% UI]	ASR per 10,0000 No.[95% UI]	Incident cases No.$\times 10^4$[95% UI]	ASR per 10,0000 No.[95% UI]	EAPC No.[95% CI]
Global	59764 (49351.5 to 72376.6)	2013.2 (1677.5 to 2409.1)	85880 (71398.6 to 102291.9)	2304 (1918.2 to 2735.2)	0.13 (-0.01 to 0.28)
Socio-demographic index					
High SDI	3594.1 (2933 to 4394.4)	917.7 (751.2 to 1111)	5749.9 (4631.7 to 7007.2)	1122.5 (911.5 to 1379.9)	0.39 (0.27 to 0.51)
High-middle SDI	6302.6 (5264 to 7647.6)	1492.1 (1203.8 to 1844.3)	13523.5 (10944 to 16569)	2185.4 (1772.1 to 2668.4)	1.15 (1.03 to 1.28)
Middle SDI	12733.8 (11003.7 to 14989.1)	1912.3 (1568.7 to 2319.6)	23844.8 (19950.5 to 28742.6)	2346.5 (1935.2 to 2794.5)	0.45 (0.32 to 0.58)
Low-middle SDI	14897.3 (13530 to 16729.1)	2794.9 (2356.3 to 3317.7)	19765.6 (17020.2 to 23596.5)	2718.3 (2276.8 to 3219.8)	-0.53 (-0.68 to -0.38)
Low SDI	6632.4 (6153.3 to 7259.5)	2419.9 (2052.4 to 2825.3)	10970.3 (9830.4 to 12522.1)	2098.7 (1779.7 to 2464.2)	-0.89 (-1.04 to -0.73)
Region					
Western Sub-Saharan Africa	2882.4 (2293.7 to 3637)	1943.1 (1618.7 to 2312.5)	5396.2 (4536.5 to 6495.3)	1850.8 (1579.8 to 2155.2)	-0.52 (-0.76 to -0.29)
Western Europe	2141.3 (1739.9 to 2662.8)	1175.8 (954 to 1452.4)	3181.4 (2567.5 to 3882.6)	1416.5 (1156.4 to 1754)	0.46 (0.32 to 0.60)
Tropical Latin America	572.1 (474.3 to 685.1)	782.6 (650.3 to 934.3)	711.9 (595.3 to 848.9)	697.6 (589.1 to 822.7)	-1.37 (-1.64 to -1.09)
Southern Sub-Saharan Africa	359.8 (290 to 460)	1280.2 (1052.4 to 1574.2)	467.5 (382.6 to 561.1)	1237.8 (1018.7 to 1478.7)	-0.71 (-0.92 to -0.50)
Southern Latin America	191.7 (162.1 to 225)	805.1 (680.2 to 946)	326.2 (267.9 to 392)	1006.2 (834.2 to 1197.2)	0.23 (0.01 to 0.45)
Southeast Asia	6850.1 (5763.8 to 8156.3)	2829.6 (2395.4 to 3304.6)	8931.3 (7502.9 to 10423.8)	2820.1 (2376.1 to 3284.7)	-0.35 (-0.48 to -0.21)
South Asia	25685.5 (21478.6 to 30865.4)	3526.9 (2956.5 to 4193.9)	33684 (27653.7 to 40530.4)	3759.6 (3101.9 to 4516)	-0.17 (-0.29 to -0.05)
Oceania	68.3 (57.1 to 83.3)	1586.4 (1359.2 to 1865.3)	144 (121.1 to 174.9)	1723.9 (1466.4 to 2038.2)	0.22 (-0.15 to 0.59)
North Africa and Middle East	3194 (2658 to 3888.4)	1370.1 (1152.2 to 1645)	4908.9 (4198.9 to 5749.9)	1585.9 (1357.4 to 1847.2)	0.08 (-0.09 to 0.24)
High-income North America	1013.2 (796.3 to 1260.2)	729.5 (574 to 906.9)	1603.5 (1252.3 to 1986.4)	844.9 (664.3 to 1050.5)	-0.07 (-0.26 to 0.11)
High-income Asia Pacific	571.8 (473.5 to 698.7)	754.8 (639.4 to 901.8)	707.3 (571.2 to 863.4)	855.7 (715.4 to 1029.6)	0.02 (-0.19 to 0.23)
Eastern Sub-Saharan Africa	2364.3 (1907.2 to 2960.8)	1773.1 (1517 to 2080.3)	3167.1 (2626.4 to 3834.6)	1269.6 (1082.7 to 1490.7)	-1.43 (-1.57 to -1.29)
Eastern Europe	848.5 (693.6 to 1026.2)	921.4 (750.4 to 1123.9)	810.7 (645.3 to 999)	1057 (847 to 1315.5)	0.16 (0.04 to 0.29)
East Asia	10228.3 (7750.1 to 13327.3)	1650.8 (1255.4 to 2151.5)	18215.3 (14296.2 to 22607.4)	2426.7 (1918.6 to 3021)	1.15 (1.02 to 1.27)
Central Sub-Saharan Africa	558.3 (485.3 to 655.1)	1272.1 (1135 to 1440.1)	793 (707 to 900.1)	933.9 (828.4 to 1051.5)	-1.32 (-1.57 to -1.08)
Central Latin America	1087 (925.5 to 1264.4)	1383.3 (1176.1 to 1604.7)	1497.9 (1247.8 to 1771.1)	1262.6 (1055.2 to 1490.8)	-0.80 (-1.24 to -0.36)
Central Europe	431.6 (342.9 to 536.5)	844.8 (669.8 to 1059.4)	489.1 (392 to 602.8)	1079.8 (861.7 to 1346.3)	0.76 (0.64 to 0.88)
Central Asia	339.4 (292.6 to 399.3)	778.4 (672.7 to 914.8)	384.6 (323.3 to 459.4)	808.2 (675.5 to 969.9)	-0.19 (-0.29 to -0.09)
Caribbean	200.8 (166.1 to 242.4)	1068.6 (897.7 to 1266.8)	207.1 (174.3 to 245.1)	932.8 (787.5 to 1104.7)	-0.82 (-0.96 to -0.67)
Australasia	46.6 (40.1 to 55.2)	514.1 (449.7 to 599.4)	68.4 (56.8 to 81.8)	530 (455 to 615.9)	0.84 (0.49 to 1.19)
Andean Latin America	129 (112.3 to 146.1)	670.7 (589.4 to 753.5)	184.3 (160.7 to 213.2)	593.8 (516.9 to 685.5)	-0.84 (-0.96 to -0.71)

Abbreviation: ASR, age standardized rate; CI, confidence interval; EAPC, estimated annual percentage change; UI, uncertainty interval.

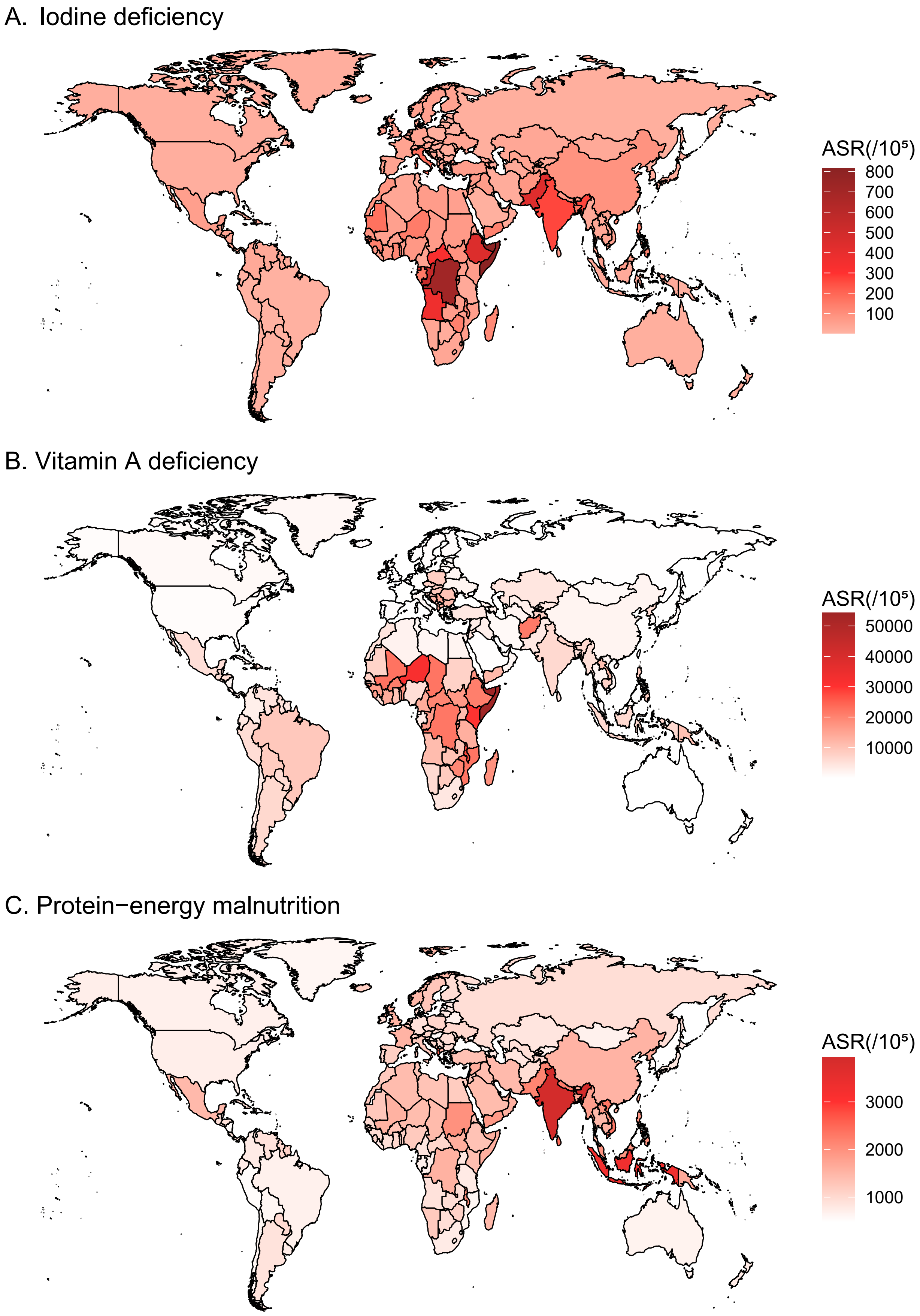

Figure S1: The ASR of iodine deficiency, vitamin A deficiency, and protein-energy malnutrition for female in 204 countries and territories in 2019. (A) iodine deficiency; (B) Vitamin A deficiency; (C) Protein-energy malnutrition

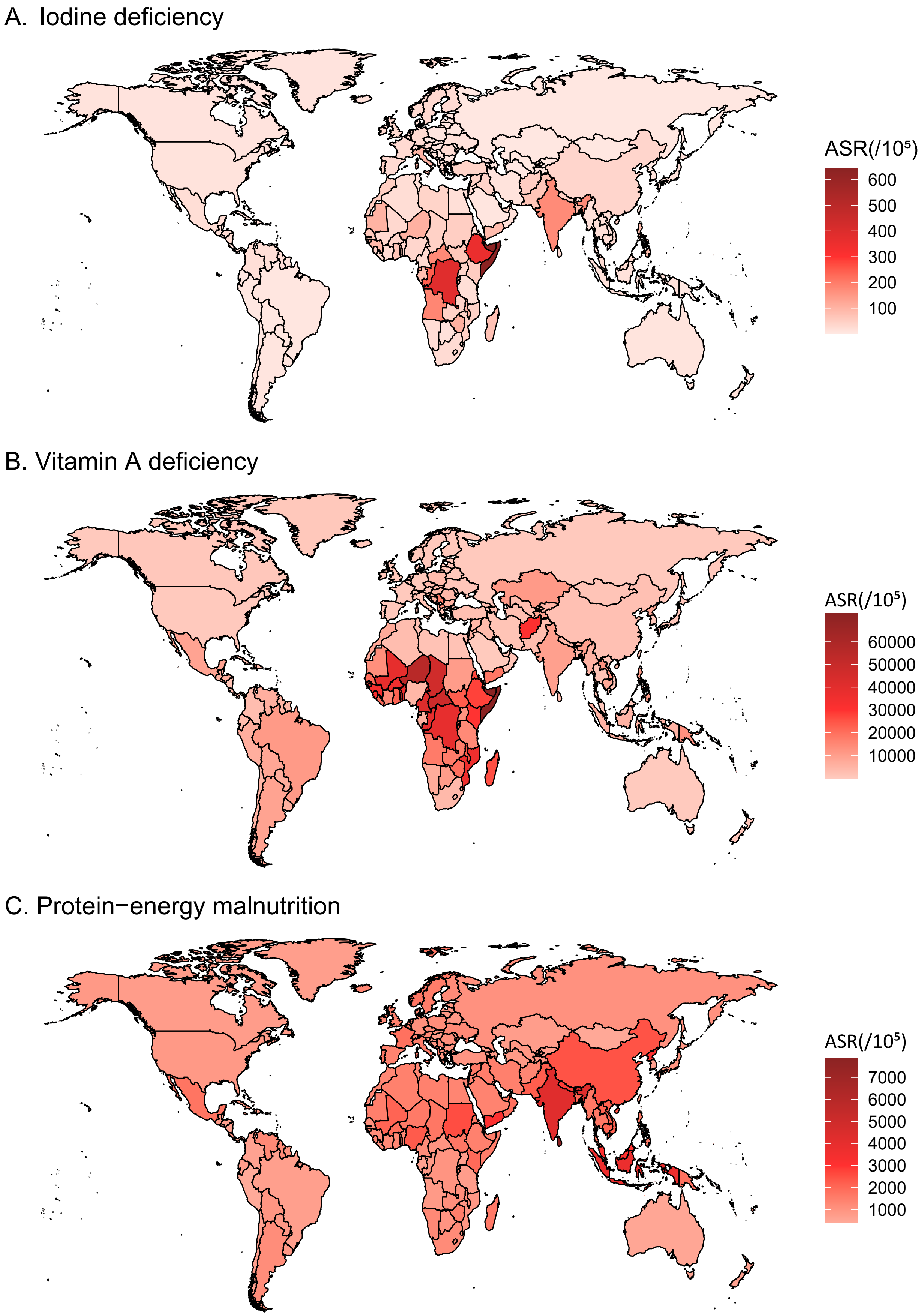

Figure S2: The ASR of iodine deficiency, vitamin A deficiency, and protein-energy malnutrition for mele in 204 countries and territories in 2019. (A) iodine deficiency; (B) Vitamin A deficiency; (C) Protein-energy malnutrition

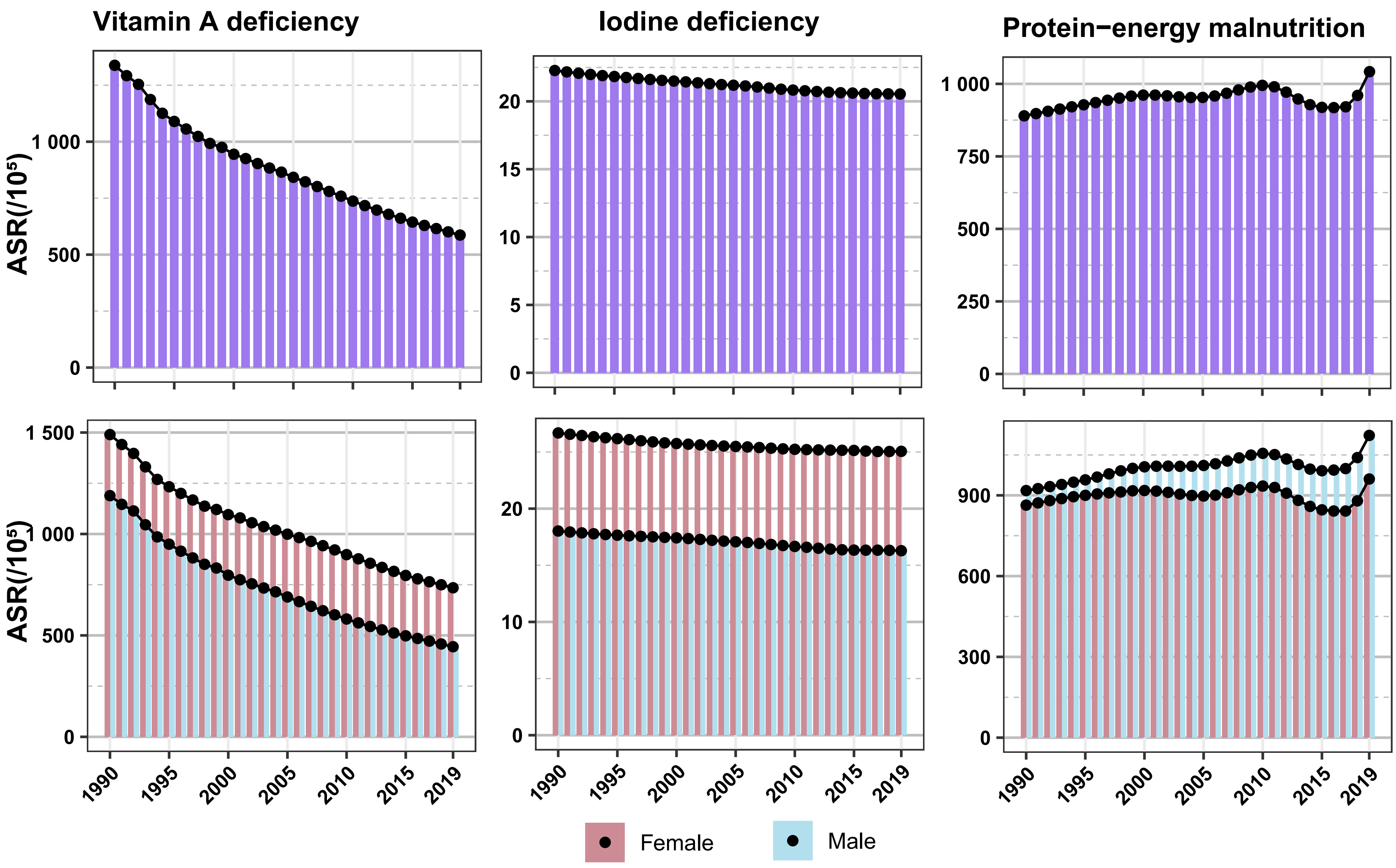

Figure S3: Changes in the burden of iodine deficiency, vitamin A deficiency, and protein-energy malnutrition over time in high SDI regions.

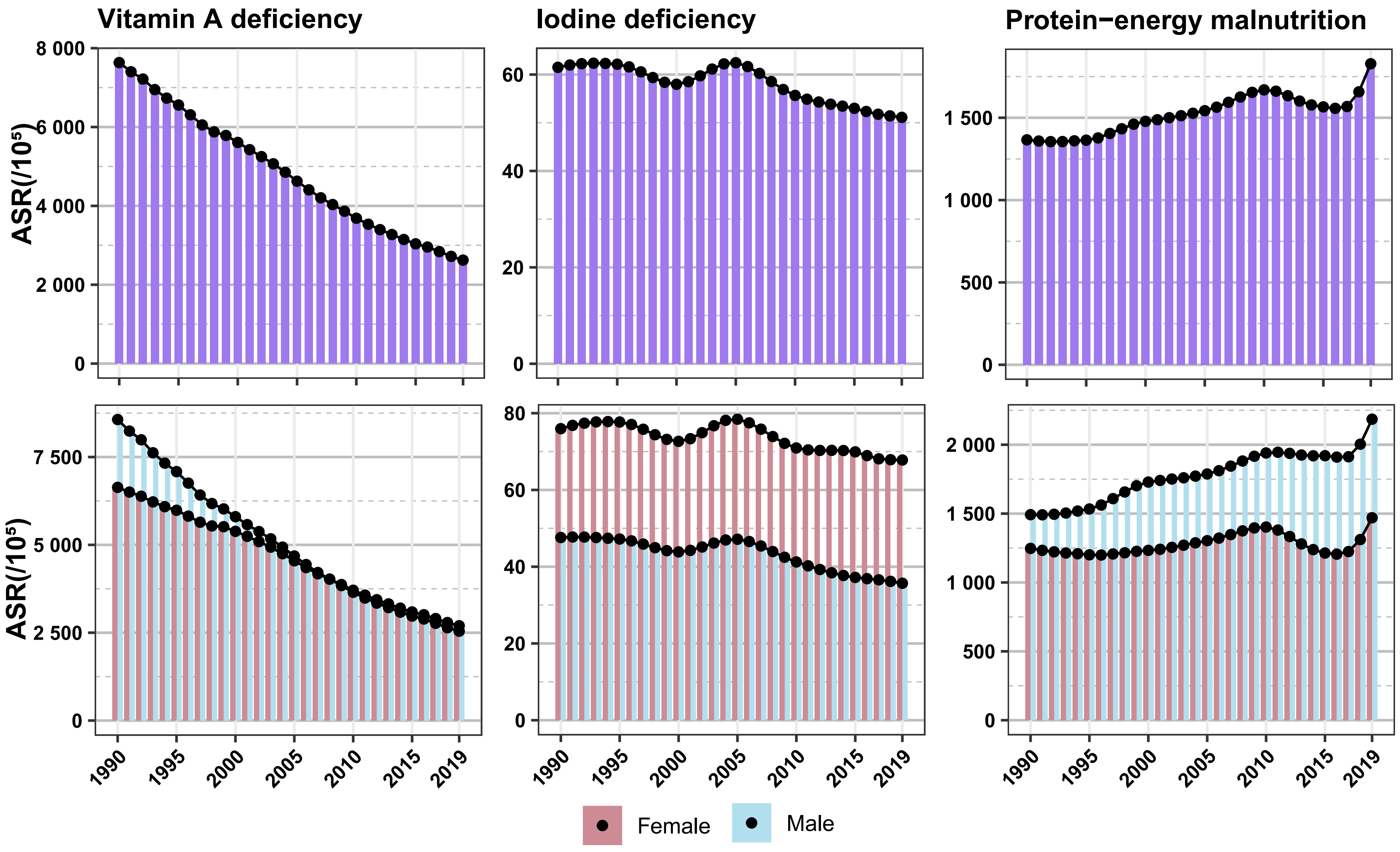

Figure S4: Changes in the burden of iodine deficiency, vitamin A deficiency, and protein-energy malnutrition over time in high-middle SDI regions.

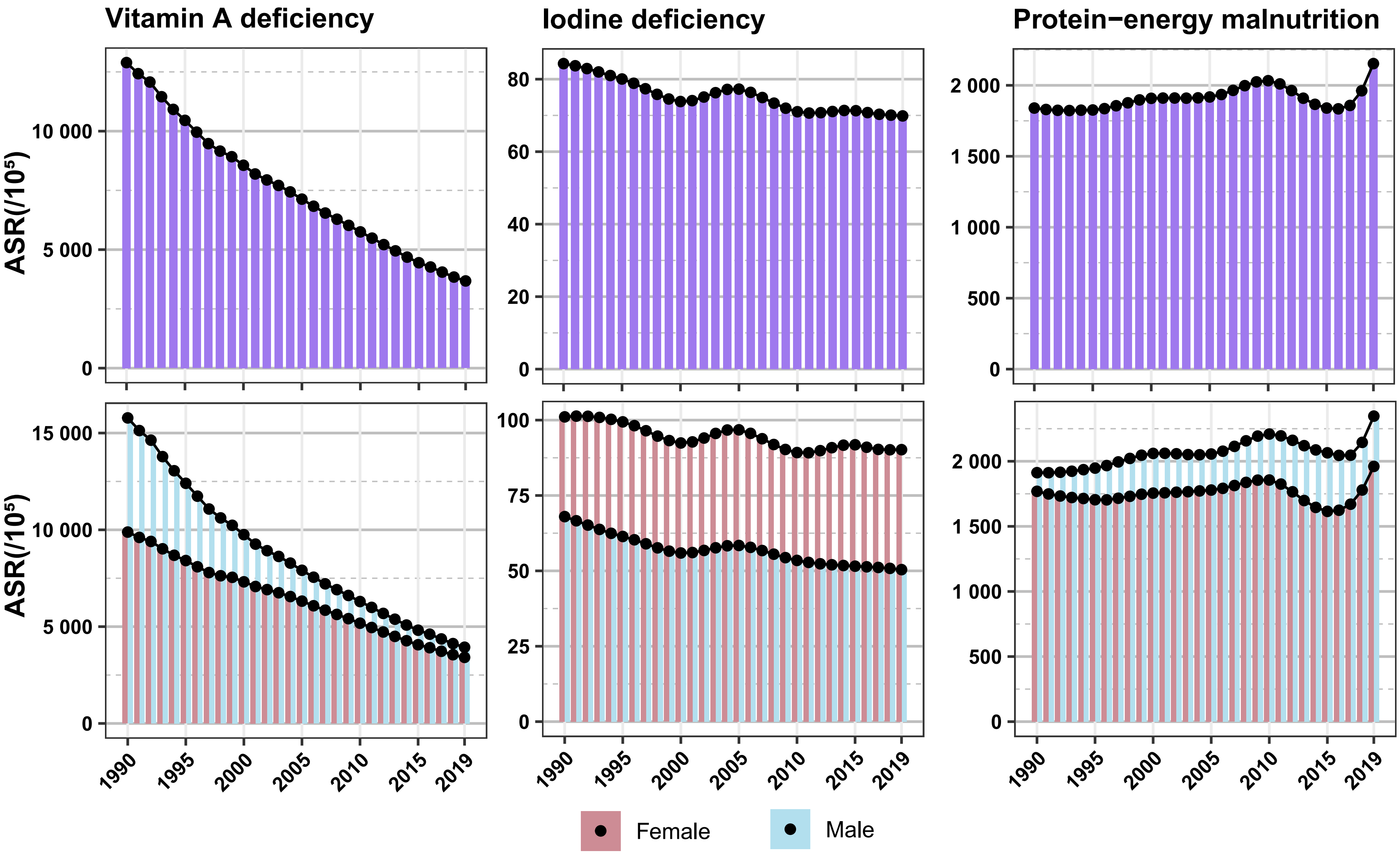

Figure S5: Changes in the burden of iodine deficiency, vitamin A deficiency, and protein-energy malnutrition over time in middle SDI regions.

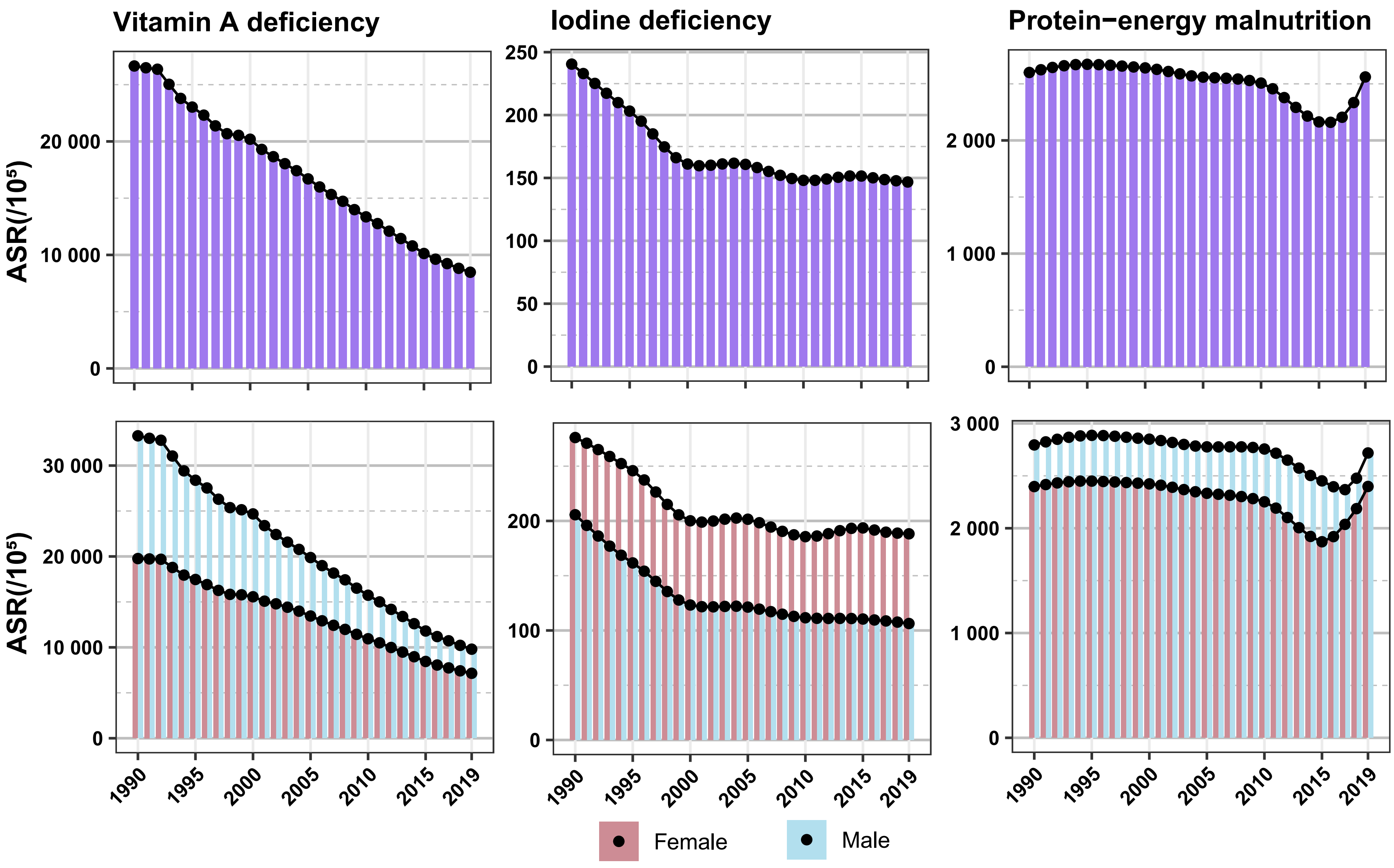

Figure S6: Changes in the burden of iodine deficiency, vitamin A deficiency, and protein-energy malnutrition over time in low-middle SDI regions.

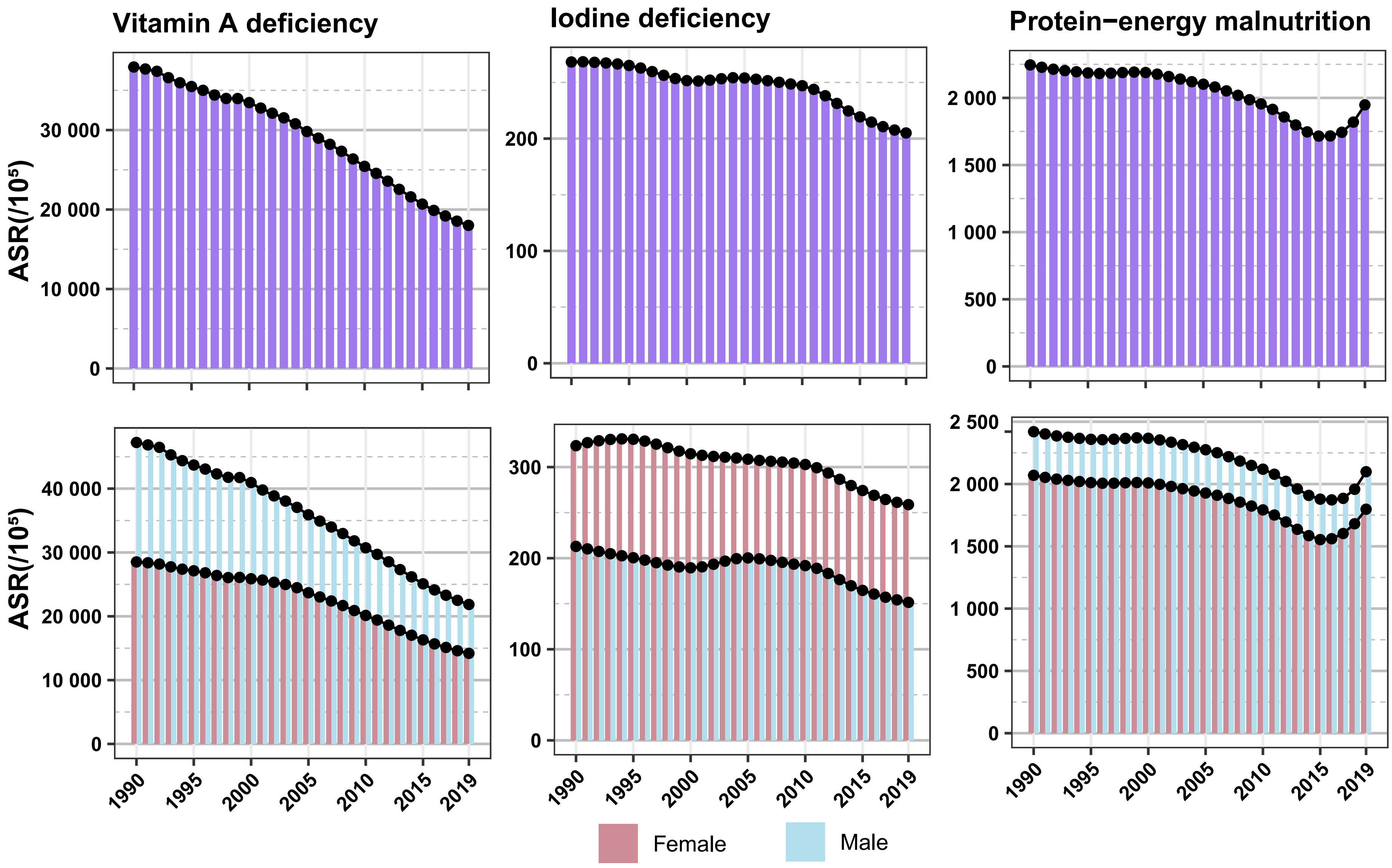

Figure S7: Changes in the burden of iodine deficiency, vitamin A deficiency, and protein-energy malnutrition over time in low SDI regions.

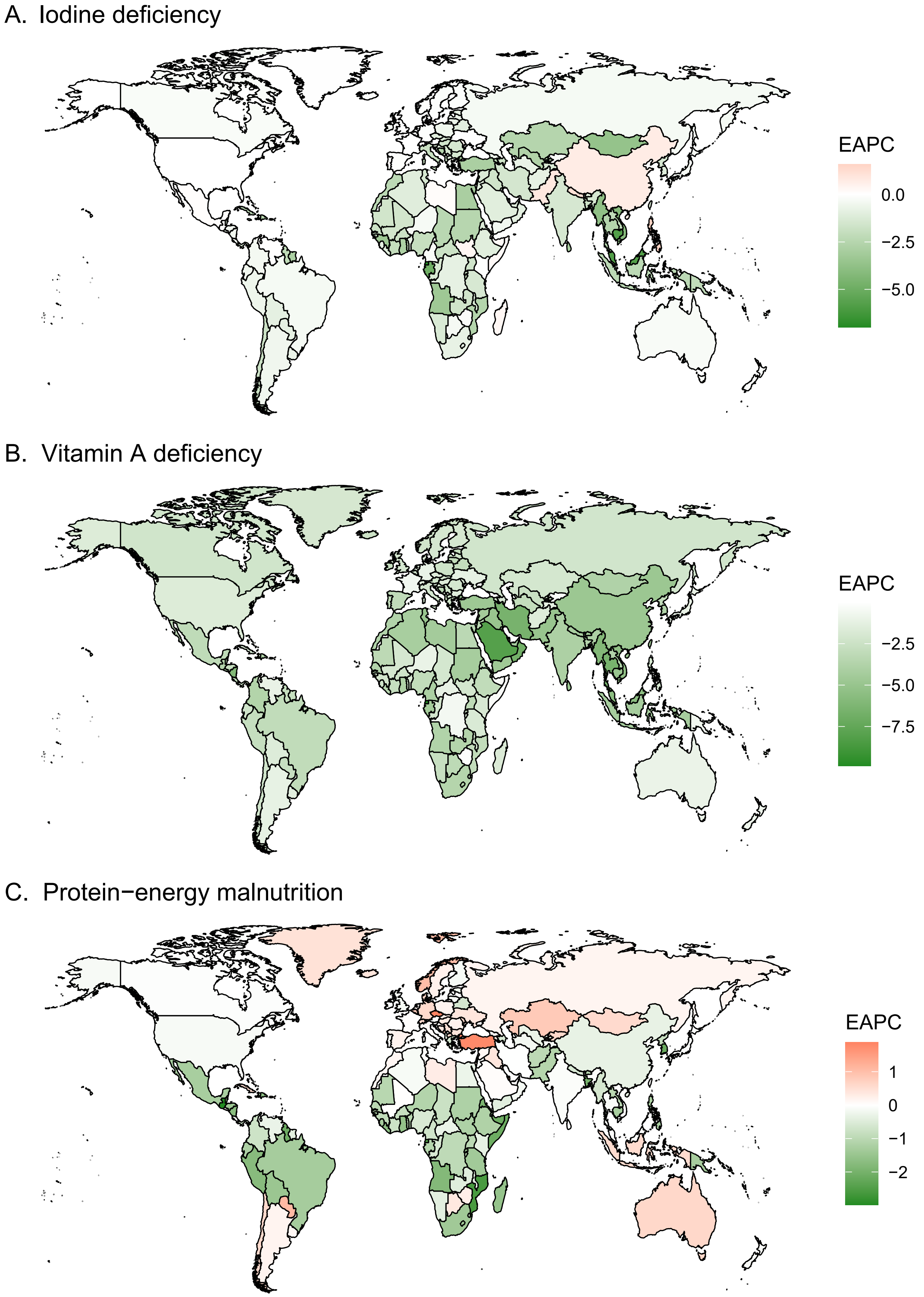

Figure S8: The EAPC of iodine deficiency, vitamin A deficiency, and protein-energy malnutrition for female in 204 countries and territories from 1990 to 2019. (A) iodine deficiency; (B) Vitamin A deficiency; (C) Protein-energy malnutrition

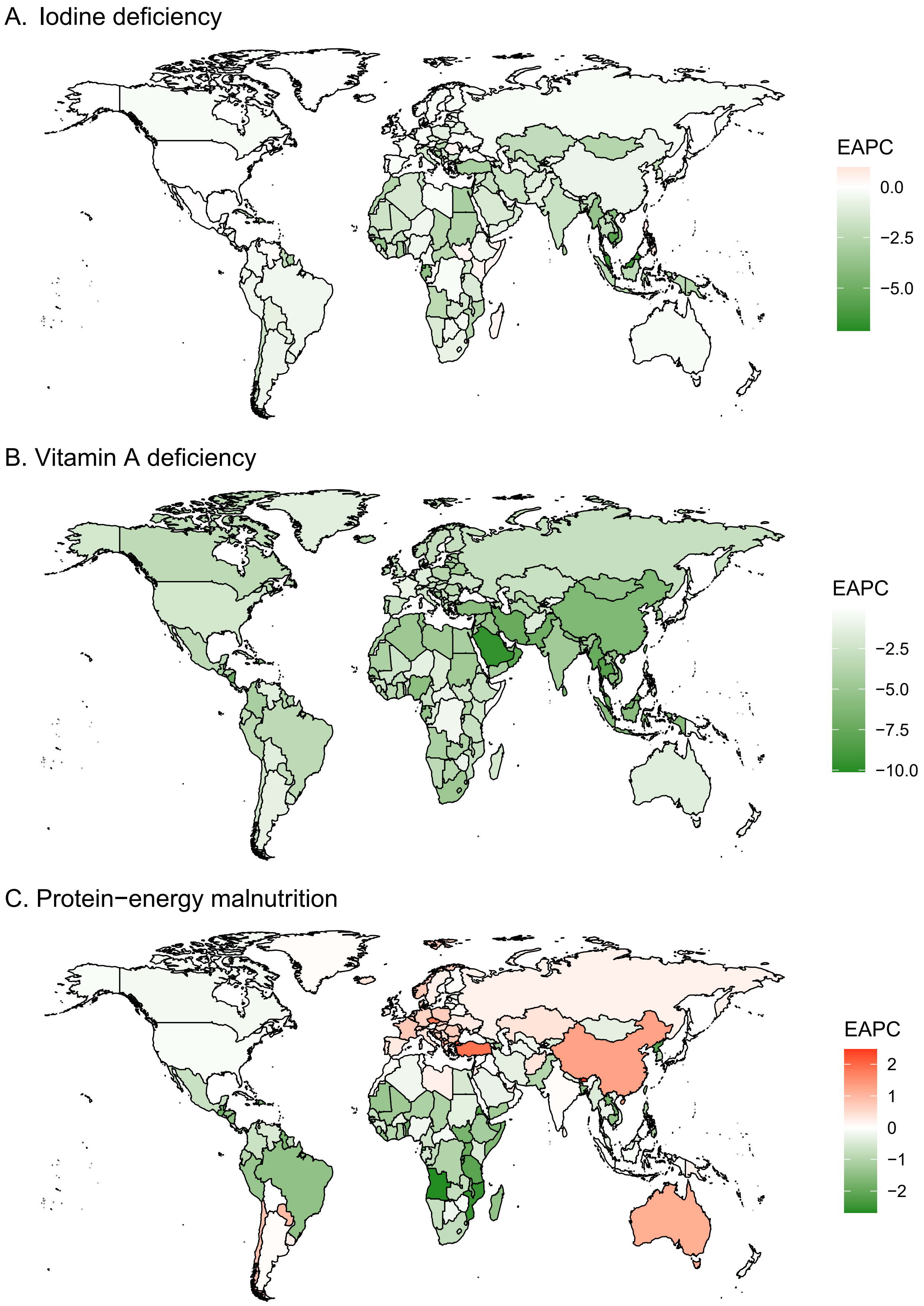

Figure S9: The EAPC of iodine deficiency, vitamin A deficiency, and protein-energy malnutrition for male in 204 countries and territories from 1990 to 2019. (A) iodine deficiency; (B) Vitamin A deficiency; (C) Protein-energy malnutrition

Table S10: The change of iodine deficiency cases between 1990 and 2019 at national level for both gender.

Country	1990		2019		1990–2019
	Incident cases No.×10^4[95% UI]	ASR per 10,0000 No.[95% UI]	Incident cases No.×10^4[95% UI]	ASR per 10,0000 No.[95% UI]	EAPC No.[95% CI]
Afghanistan	9.8 (7.4 to 12.3)	70.9 (55.3 to 87.3)	9.8 (7.4 to 12.3)	70.9 (55.3 to 87.3)	-0.99 (-1.83 to -0.14)
Albania	0.6 (0.5 to 0.8)	17 (13 to 21.4)	0.6 (0.5 to 0.8)	17 (13 to 21.4)	-1.89 (-2.05 to -1.73)
Algeria	11.5 (8.8 to 14.6)	38.4 (30.4 to 47.4)	11.5 (8.8 to 14.6)	38.4 (30.4 to 47.4)	-1.22 (-1.37 to -1.07)
American Samoa	0 (0 to 0)	3.8 (2.8 to 5)	0 (0 to 0)	3.8 (2.8 to 5)	-1.18 (-1.29 to -1.08)
Andorra	0 (0 to 0)	34.7 (27 to 44.7)	0 (0 to 0)	34.7 (27 to 44.7)	0.07 (0.03 to 0.10)
Angola	69.4 (55.5 to 84.8)	503 (405.4 to 608.9)	69.4 (55.5 to 84.8)	503 (405.4 to 608.9)	-2.98 (-3.46 to -2.50)
Antigua and Barbuda	0 (0 to 0)	18.6 (14.4 to 23.5)	0 (0 to 0)	18.6 (14.4 to 23.5)	-2.42 (-2.57 to -2.27)
Argentina	3.3 (2.5 to 4.2)	9.8 (7.4 to 12.5)	3.3 (2.5 to 4.2)	9.8 (7.4 to 12.5)	-0.57 (-0.60 to -0.55)
Armenia	1.9 (1.4 to 2.4)	52.4 (40.6 to 66.6)	1.9 (1.4 to 2.4)	52.4 (40.6 to 66.6)	-2.28 (-2.83 to -1.72)
Australia	2.1 (1.6 to 2.7)	12.8 (10 to 16.2)	2.1 (1.6 to 2.7)	12.8 (10 to 16.2)	-0.24 (-0.25 to -0.23)
Austria	2.8 (2.1 to 3.6)	38.2 (29.8 to 48.6)	2.8 (2.1 to 3.6)	38.2 (29.8 to 48.6)	-0.11 (-0.13 to -0.09)
Azerbaijan	1.2 (0.9 to 1.6)	15.5 (11.7 to 19.8)	1.2 (0.9 to 1.6)	15.5 (11.7 to 19.8)	-1.70 (-2.23 to -1.16)
Bahamas	0 (0 to 0)	12.7 (9.8 to 16.1)	0 (0 to 0)	12.7 (9.8 to 16.1)	-1.66 (-1.78 to -1.53)
Bahrain	0.1 (0.1 to 0.2)	26.7 (21.1 to 33.5)	0.1 (0.1 to 0.2)	26.7 (21.1 to 33.5)	-0.99 (-1.07 to -0.91)
Bangladesh	421 (376.4 to 471.3)	293.7 (260.2 to 329.5)	421 (376.4 to 471.3)	293.7 (260.2 to 329.5)	-1.86 (-2.53 to -1.19)
Barbados	0.1 (0.1 to 0.1)	26.2 (20.2 to 33.5)	0.1 (0.1 to 0.1)	26.2 (20.2 to 33.5)	-0.68 (-0.76 to -0.60)
Belarus	0.9 (0.6 to 1.1)	8.9 (6.6 to 11.5)	0.9 (0.6 to 1.1)	8.9 (6.6 to 11.5)	-0.99 (-1.31 to -0.67)
Belgium	3.4 (2.7 to 4.4)	38 (29.6 to 49)	3.4 (2.7 to 4.4)	38 (29.6 to 49)	-0.19 (-0.20 to -0.18)
Belize	0 (0 to 0)	9.2 (7.1 to 11.7)	0 (0 to 0)	9.2 (7.1 to 11.7)	-1.53 (-1.59 to -1.47)
Benin	3.2 (2.4 to 4.1)	59.2 (45.9 to 75.8)	3.2 (2.4 to 4.1)	59.2 (45.9 to 75.8)	-0.87 (-0.95 to -0.80)
Bermuda	0 (0 to 0)	11.2 (8.6 to 14.5)	0 (0 to 0)	11.2 (8.6 to 14.5)	-2.20 (-2.36 to -2.04)
Bhutan	0.4 (0.3 to 0.5)	47.7 (36.3 to 61)	0.4 (0.3 to 0.5)	47.7 (36.3 to 61)	-0.94 (-1.38 to -0.51)
Bolivia (Plurinational State of)	0.4 (0.4 to 0.6)	6.6 (5.3 to 8.2)	0.4 (0.4 to 0.6)	6.6 (5.3 to 8.2)	-0.88 (-0.95 to -0.82)
Bosnia and Herzegovina	1.6 (1.2 to 1.9)	36.3 (27.9 to 45)	1.6 (1.2 to 1.9)	36.3 (27.9 to 45)	-2.64 (-3.06 to -2.21)
Botswana	0.3 (0.2 to 0.4)	20.8 (16.5 to 25.9)	0.3 (0.2 to 0.4)	20.8 (16.5 to 25.9)	-0.43 (-0.48 to -0.38)
Brazil	11.1 (8.2 to 14.6)	7 (5.3 to 8.9)	11.1 (8.2 to 14.6)	7 (5.3 to 8.9)	-0.43 (-0.46 to -0.39)
Brunei Darussalam	0 (0 to 0.1)	14.7 (11.5 to 18.6)	0 (0 to 0.1)	14.7 (11.5 to 18.6)	-0.23 (-0.26 to -0.20)
Bulgaria	1 (0.7 to 1.2)	12.4 (9.2 to 15.8)	1 (0.7 to 1.2)	12.4 (9.2 to 15.8)	-1.45 (-1.53 to -1.37)
Burkina Faso	8.2 (6.3 to 10.3)	76.9 (60.5 to 96.7)	8.2 (6.3 to 10.3)	76.9 (60.5 to 96.7)	-1.84 (-1.97 to -1.71)
Burundi	12.6 (11.2 to 14)	185.8 (163.9 to 208.1)	12.6 (11.2 to 14)	185.8 (163.9 to 208.1)	-2.07 (-2.21 to -1.93)
Cabo Verde	0.4 (0.3 to 0.5)	98.3 (77 to 122.9)	0.4 (0.3 to 0.5)	98.3 (77 to 122.9)	-1.42 (-1.57 to -1.27)
Cambodia	16.6 (12.7 to 21.3)	143.5 (109.4 to 184.1)	16.6 (12.7 to 21.3)	143.5 (109.4 to 184.1)	-5.83 (-6.33 to -5.32)

Continued on next page

Table S10 – continued from previous page

Country	1990		2019		1990–2019
	Incident cases No.×10^4[95% UI]	ASR per 10,0000 No.[95% UI]	Incident cases No.×10^4[95% UI]	ASR per 10,0000 No.[95% UI]	EAPC No.[95% CI]
Cameroon	4.4 (3.4 to 5.8)	39.2 (30.8 to 50.3)	4.4 (3.4 to 5.8)	39.2 (30.8 to 50.3)	-0.30 (-0.43 to -0.17)
Canada	3.6 (2.8 to 4.5)	13.6 (10.6 to 17.1)	3.6 (2.8 to 4.5)	13.6 (10.6 to 17.1)	-0.22 (-0.23 to -0.20)
Central African Republic	13.1 (11.6 to 14.7)	370.7 (325.3 to 417)	13.1 (11.6 to 14.7)	370.7 (325.3 to 417)	-2.01 (-2.39 to -1.64)
Chad	7.4 (5.8 to 9.3)	102.1 (80.7 to 126.5)	7.4 (5.8 to 9.3)	102.1 (80.7 to 126.5)	-2.42 (-2.56 to -2.27)
Chile	1.8 (1.4 to 2.2)	13.1 (10.1 to 16.1)	1.8 (1.4 to 2.2)	13.1 (10.1 to 16.1)	-1.77 (-1.92 to -1.62)
China	797.5 (609.6 to 1036.4)	60.1 (46.7 to 77)	797.5 (609.6 to 1036.4)	60.1 (46.7 to 77)	0.19 (-0.26 to 0.64)
Colombia	10.1 (7.5 to 13.1)	27.2 (20.7 to 34.8)	10.1 (7.5 to 13.1)	27.2 (20.7 to 34.8)	-0.37 (-0.48 to -0.25)
Comoros	0.1 (0.1 to 0.2)	23.1 (18.1 to 29.2)	0.1 (0.1 to 0.2)	23.1 (18.1 to 29.2)	-0.14 (-0.20 to -0.08)
Congo	18 (14.8 to 21)	532.9 (440.8 to 619.5)	18 (14.8 to 21)	532.9 (440.8 to 619.5)	-0.92 (-1.03 to -0.81)
Cook Islands	0 (0 to 0)	4.3 (3.2 to 5.6)	0 (0 to 0)	4.3 (3.2 to 5.6)	-1.36 (-1.49 to -1.22)
Costa Rica	0.9 (0.7 to 1.1)	25.5 (19.4 to 32.2)	0.9 (0.7 to 1.1)	25.5 (19.4 to 32.2)	-0.40 (-0.46 to -0.33)
Croatia	0.6 (0.5 to 0.8)	13.9 (10.6 to 17.5)	0.6 (0.5 to 0.8)	13.9 (10.6 to 17.5)	-1.16 (-1.28 to -1.04)
Cuba	2.9 (2.2 to 3.7)	26.3 (20.3 to 33.5)	2.9 (2.2 to 3.7)	26.3 (20.3 to 33.5)	-2.15 (-2.69 to -1.62)
Cyprus	0.3 (0.2 to 0.4)	40.1 (31.2 to 51.5)	0.3 (0.2 to 0.4)	40.1 (31.2 to 51.5)	-0.16 (-0.17 to -0.15)
Czechia	0.9 (0.7 to 1.1)	9 (6.8 to 11.6)	0.9 (0.7 to 1.1)	9 (6.8 to 11.6)	-1.05 (-1.17 to -0.92)
Ivoirian	11.3 (8.5 to 14.2)	77.7 (60.3 to 97.6)	11.3 (8.5 to 14.2)	77.7 (60.3 to 97.6)	-1.90 (-2.06 to -1.73)
Democratic People's Republic of Korea	1.2 (0.9 to 1.6)	5.9 (4.5 to 7.6)	1.2 (0.9 to 1.6)	5.9 (4.5 to 7.6)	-1.70 (-1.92 to -1.48)
Democratic Republic of the Congo	360.5 (326.2 to 396.2)	689.8 (622.5 to 765)	360.5 (326.2 to 396.2)	689.8 (622.5 to 765)	-0.64 (-0.94 to -0.34)
Denmark	1.4 (1.1 to 1.8)	29.4 (22.9 to 37.4)	1.4 (1.1 to 1.8)	29.4 (22.9 to 37.4)	-0.20 (-0.23 to -0.17)
Djibouti	3.2 (2.6 to 4)	524.6 (419.3 to 646.9)	3.2 (2.6 to 4)	524.6 (419.3 to 646.9)	-0.32 (-0.70 to 0.07)
Dominica	0 (0 to 0)	28.7 (21.9 to 36.8)	0 (0 to 0)	28.7 (21.9 to 36.8)	-2.51 (-2.70 to -2.33)
Dominican Republic	3 (2.3 to 3.9)	35.7 (27.6 to 45.4)	3 (2.3 to 3.9)	35.7 (27.6 to 45.4)	-2.43 (-2.55 to -2.32)
Ecuador	0.6 (0.4 to 0.8)	5.3 (4 to 6.9)	0.6 (0.4 to 0.8)	5.3 (4 to 6.9)	-0.47 (-0.54 to -0.41)
Egypt	50 (38.1 to 63.9)	74.1 (58 to 92.7)	50 (38.1 to 63.9)	74.1 (58 to 92.7)	-2.97 (-3.21 to -2.73)
El Salvador	2.1 (1.6 to 2.8)	33 (24.7 to 41.1)	2.1 (1.6 to 2.8)	33 (24.7 to 41.1)	-0.78 (-0.86 to -0.69)
Equatorial Guinea	6.7 (5.5 to 7.8)	1071.8 (902.5 to 1226.4)	6.7 (5.5 to 7.8)	1071.8 (902.5 to 1226.4)	-7.18 (-8.02 to -6.33)
Eritrea	1.4 (1 to 1.8)	40.2 (31.6 to 51.6)	1.4 (1 to 1.8)	40.2 (31.6 to 51.6)	-1.12 (-1.32 to -0.93)
Estonia	0.1 (0.1 to 0.1)	6.2 (4.5 to 8.1)	0.1 (0.1 to 0.1)	6.2 (4.5 to 8.1)	-0.82 (-0.90 to -0.74)
Eswatini	0.5 (0.4 to 0.7)	56.5 (44.1 to 71.8)	0.5 (0.4 to 0.7)	56.5 (44.1 to 71.8)	-0.77 (-0.81 to -0.74)
Ethiopia	361.9 (293.5 to 443.7)	578.2 (463.3 to 712.6)	361.9 (293.5 to 443.7)	578.2 (463.3 to 712.6)	-0.81 (-1.30 to -0.32)
Fiji	0 (0 to 0.1)	5.4 (4.1 to 7.1)	0 (0 to 0.1)	5.4 (4.1 to 7.1)	-1.82 (-2.03 to -1.61)
Finland	1.7 (1.4 to 2.2)	38.3 (29.6 to 48.6)	1.7 (1.4 to 2.2)	38.3 (29.6 to 48.6)	-0.18 (-0.20 to -0.15)
France	20.5 (16 to 26.2)	37.9 (29.4 to 48.6)	20.5 (16 to 26.2)	37.9 (29.4 to 48.6)	-0.04 (-0.05 to -0.03)
Gabon	3.8 (3 to 4.7)	307.2 (247.2 to 378.2)	3.8 (3 to 4.7)	307.2 (247.2 to 378.2)	-4.51 (-5.10 to -3.91)
Gambia	2.3 (1.8 to 3)	196.1 (153.8 to 248.3)	2.3 (1.8 to 3)	196.1 (153.8 to 248.3)	-1.44 (-1.76 to -1.12)

Continued on next page

Table S10 – continued from previous page

Country	1990		2019		1990–2019
	Incident cases No.$\times 10^4$[95% UI]	ASR per 10,0000 No.[95% UI]	Incident cases No.$\times 10^4$[95% UI]	ASR per 10,0000 No.[95% UI]	EAPC No.[95% CI]
Georgia	0.9 (0.7 to 1.2)	18.1 (13.6 to 22.9)	0.9 (0.7 to 1.2)	18.1 (13.6 to 22.9)	-0.68 (-0.78 to -0.58)
Germany	27 (21.1 to 34.8)	37.7 (29.5 to 48.4)	27 (21.1 to 34.8)	37.7 (29.5 to 48.4)	-0.14 (-0.17 to -0.11)
Ghana	35.2 (27.8 to 44.5)	198.3 (155.6 to 250.5)	35.2 (27.8 to 44.5)	198.3 (155.6 to 250.5)	-2.82 (-3.03 to -2.60)
Greece	3.9 (3.1 to 5)	41.4 (32 to 52.2)	3.9 (3.1 to 5)	41.4 (32 to 52.2)	-0.36 (-0.44 to -0.29)
Greenland	0 (0 to 0)	12.6 (9.8 to 15.9)	0 (0 to 0)	12.6 (9.8 to 15.9)	-0.03 (-0.06 to 0.00)
Grenada	0 (0 to 0)	29.7 (22.8 to 38)	0 (0 to 0)	29.7 (22.8 to 38)	-3.20 (-3.34 to -3.06)
Guam	0 (0 to 0)	3.8 (2.8 to 5)	0 (0 to 0)	3.8 (2.8 to 5)	-1.07 (-1.23 to -0.92)
Guatemala	2.6 (1.9 to 3.5)	26.8 (20.2 to 34.3)	2.6 (1.9 to 3.5)	26.8 (20.2 to 34.3)	-0.10 (-0.17 to -0.03)
Guinea	21.5 (17 to 26)	269.4 (217.6 to 328.9)	21.5 (17 to 26)	269.4 (217.6 to 328.9)	-3.42 (-3.69 to -3.15)
Guinea-Bissau	3.1 (2.5 to 3.8)	253.2 (206.4 to 310.6)	3.1 (2.5 to 3.8)	253.2 (206.4 to 310.6)	-1.65 (-1.83 to -1.48)
Guyana	0.4 (0.3 to 0.5)	43 (33 to 54.3)	0.4 (0.3 to 0.5)	43 (33 to 54.3)	-2.13 (-2.28 to -1.97)
Haiti	4.9 (3.7 to 6)	62.2 (48.5 to 77.1)	4.9 (3.7 to 6)	62.2 (48.5 to 77.1)	-0.95 (-1.23 to -0.67)
Honduras	2.2 (1.6 to 2.9)	35.4 (26.8 to 45.1)	2.2 (1.6 to 2.9)	35.4 (26.8 to 45.1)	-0.40 (-0.57 to -0.24)
Hungary	1.7 (1.3 to 2)	18 (13.8 to 22.2)	1.7 (1.3 to 2)	18 (13.8 to 22.2)	-2.00 (-2.19 to -1.82)
Iceland	0 (0 to 0.1)	18.2 (14.2 to 23.4)	0 (0 to 0.1)	18.2 (14.2 to 23.4)	-0.15 (-0.21 to -0.09)
India	3785.6 (3031.6 to 4631.6)	363.7 (292.5 to 447.8)	3785.6 (3031.6 to 4631.6)	363.7 (292.5 to 447.8)	-1.64 (-2.06 to -1.22)
Indonesia	77.6 (58.7 to 99.9)	37 (28.5 to 47.3)	77.6 (58.7 to 99.9)	37 (28.5 to 47.3)	-2.32 (-2.42 to -2.22)
Iran (Islamic Republic of)	15.9 (11.8 to 20.4)	23.4 (18.4 to 29)	15.9 (11.8 to 20.4)	23.4 (18.4 to 29)	-1.62 (-1.82 to -1.42)
Iraq	14.5 (11 to 18.1)	65.1 (51.6 to 79.7)	14.5 (11 to 18.1)	65.1 (51.6 to 79.7)	-1.75 (-2.26 to -1.24)
Ireland	1.5 (1.1 to 1.9)	40.2 (31.2 to 51.4)	1.5 (1.1 to 1.9)	40.2 (31.2 to 51.4)	-0.35 (-0.38 to -0.32)
Israel	2 (1.6 to 2.6)	40.1 (31.1 to 51.3)	2 (1.6 to 2.6)	40.1 (31.1 to 51.3)	-0.17 (-0.18 to -0.15)
Italy	100.1 (76 to 128.4)	196 (151.4 to 248.6)	100.1 (76 to 128.4)	196 (151.4 to 248.6)	-1.74 (-2.06 to -1.41)
Jamaica	0.8 (0.6 to 1)	28.5 (21.8 to 36.1)	0.8 (0.6 to 1)	28.5 (21.8 to 36.1)	-1.85 (-1.99 to -1.70)
Japan	20.3 (16 to 25.4)	17.1 (13.4 to 21.4)	20.3 (16 to 25.4)	17.1 (13.4 to 21.4)	-0.38 (-0.40 to -0.36)
Jordan	1.7 (1.3 to 2.2)	37.5 (29.8 to 46.7)	1.7 (1.3 to 2.2)	37.5 (29.8 to 46.7)	-1.23 (-1.34 to -1.11)
Kazakhstan	5.5 (4.2 to 7.1)	31.3 (23.8 to 40.2)	5.5 (4.2 to 7.1)	31.3 (23.8 to 40.2)	-2.51 (-2.87 to -2.16)
Kenya	15.5 (11.8 to 19.9)	57 (44.9 to 71.8)	15.5 (11.8 to 19.9)	57 (44.9 to 71.8)	0.14 (-0.13 to 0.42)
Kiribati	0 (0 to 0)	7.6 (5.8 to 9.8)	0 (0 to 0)	7.6 (5.8 to 9.8)	-2.02 (-2.40 to -1.64)
Kuwait	0.5 (0.4 to 0.6)	25.4 (20 to 31.8)	0.5 (0.4 to 0.6)	25.4 (20 to 31.8)	-0.93 (-1.02 to -0.85)
Kyrgyzstan	0.9 (0.7 to 1.2)	17.9 (13.7 to 23)	0.9 (0.7 to 1.2)	17.9 (13.7 to 23)	-0.31 (-0.34 to -0.27)
Lao People's Democratic Republic	1.7 (1.3 to 2.2)	37.8 (28.9 to 48.8)	1.7 (1.3 to 2.2)	37.8 (28.9 to 48.8)	-2.17 (-2.29 to -2.05)
Latvia	0.1 (0.1 to 0.2)	6 (4.5 to 7.9)	0.1 (0.1 to 0.2)	6 (4.5 to 7.9)	-0.65 (-0.87 to -0.44)
Lebanon	4.1 (3.5 to 4.7)	105.1 (91.4 to 120.6)	4.1 (3.5 to 4.7)	105.1 (91.4 to 120.6)	-2.34 (-2.76 to -1.92)
Lesotho	12.4 (11.4 to 13.5)	517.5 (469.9 to 565.5)	12.4 (11.4 to 13.5)	517.5 (469.9 to 565.5)	-3.97 (-4.76 to -3.17)
Liberia	0.8 (0.6 to 1.1)	39.4 (30.7 to 50.1)	0.8 (0.6 to 1.1)	39.4 (30.7 to 50.1)	-1.09 (-1.49 to -0.70)

Continued on next page

Table S10 – continued from previous page

Country	1990		2019		1990–2019
	Incident cases No.×10^4[95% UI]	ASR per 10,0000 No.[95% UI]	Incident cases No.×10^4[95% UI]	ASR per 10,0000 No.[95% UI]	EAPC No.[95% CI]
Libya	2.3 (1.7 to 2.9)	44 (34.7 to 54.4)	2.3 (1.7 to 2.9)	44 (34.7 to 54.4)	-0.06 (-0.40 to 0.29)
Lithuania	0.2 (0.2 to 0.3)	5.9 (4.4 to 7.7)	0.2 (0.2 to 0.3)	5.9 (4.4 to 7.7)	-0.70 (-0.91 to -0.50)
Luxembourg	0.1 (0.1 to 0.2)	36.3 (28.3 to 46.3)	0.1 (0.1 to 0.2)	36.3 (28.3 to 46.3)	-0.07 (-0.08 to -0.06)
Madagascar	11.8 (8.9 to 15.1)	89.4 (69 to 112.7)	11.8 (8.9 to 15.1)	89.4 (69 to 112.7)	0.32 (0.15 to 0.48)
Malawi	14.4 (11.2 to 18.4)	127.9 (100.3 to 164.9)	14.4 (11.2 to 18.4)	127.9 (100.3 to 164.9)	-1.36 (-1.67 to -1.04)
Malaysia	47.9 (40.8 to 56.4)	232.7 (196.6 to 273.7)	47.9 (40.8 to 56.4)	232.7 (196.6 to 273.7)	-6.05 (-6.37 to -5.73)
Maldives	0.1 (0.1 to 0.1)	43.6 (33.5 to 55.5)	0.1 (0.1 to 0.1)	43.6 (33.5 to 55.5)	-4.69 (-4.81 to -4.57)
Mali	6 (4.6 to 7.8)	64 (50.3 to 81.9)	6 (4.6 to 7.8)	64 (50.3 to 81.9)	-1.57 (-1.67 to -1.48)
Malta	0.2 (0.1 to 0.2)	43.1 (33.4 to 55.2)	0.2 (0.1 to 0.2)	43.1 (33.4 to 55.2)	-0.38 (-0.39 to -0.37)
Marshall Islands	0 (0 to 0)	6.8 (5.2 to 8.8)	0 (0 to 0)	6.8 (5.2 to 8.8)	-2.14 (-2.46 to -1.81)
Mauritania	5.1 (4 to 6.2)	204.9 (161.9 to 255.3)	5.1 (4 to 6.2)	204.9 (161.9 to 255.3)	-1.63 (-1.88 to -1.39)
Mauritius	0.5 (0.4 to 0.6)	40.1 (30.7 to 51.2)	0.5 (0.4 to 0.6)	40.1 (30.7 to 51.2)	-2.53 (-2.59 to -2.46)
Mexico	40.6 (30.6 to 52)	38.4 (29.6 to 48.2)	40.6 (30.6 to 52)	38.4 (29.6 to 48.2)	0.05 (-0.01 to 0.10)
Micronesia (Federated States of)	0 (0 to 0)	6.9 (5.3 to 8.9)	0 (0 to 0)	6.9 (5.3 to 8.9)	-2.14 (-2.42 to -1.86)
Monaco	0 (0 to 0)	33 (25.5 to 42)	0 (0 to 0)	33 (25.5 to 42)	0.11 (0.09 to 0.14)
Mongolia	1 (0.8 to 1.3)	37.6 (29.7 to 44.8)	1 (0.8 to 1.3)	37.6 (29.7 to 44.8)	-3.50 (-3.82 to -3.18)
Montenegro	0.1 (0.1 to 0.1)	14 (10.7 to 17.6)	0.1 (0.1 to 0.1)	14 (10.7 to 17.6)	-1.16 (-1.26 to -1.06)
Morocco	40.5 (31.9 to 51.2)	128.7 (102.6 to 159.5)	40.5 (31.9 to 51.2)	128.7 (102.6 to 159.5)	-2.42 (-2.60 to -2.24)
Mozambique	18.7 (14.6 to 23)	117.3 (92.6 to 147.2)	18.7 (14.6 to 23)	117.3 (92.6 to 147.2)	-2.52 (-2.90 to -2.15)
Myanmar	28.9 (21.9 to 37.4)	62 (47.4 to 80.3)	28.9 (21.9 to 37.4)	62 (47.4 to 80.3)	-3.76 (-3.96 to -3.55)
Namibia	0.7 (0.5 to 0.9)	42.3 (33.2 to 53.5)	0.7 (0.5 to 0.9)	42.3 (33.2 to 53.5)	-1.29 (-1.34 to -1.25)
Nauru	0 (0 to 0)	4.2 (3.1 to 5.6)	0 (0 to 0)	4.2 (3.1 to 5.6)	-0.97 (-1.55 to -0.39)
Nepal	16.9 (12.9 to 21.8)	75.6 (58.3 to 96.3)	16.9 (12.9 to 21.8)	75.6 (58.3 to 96.3)	0.48 (-0.13 to 1.09)
Netherlands	5.3 (4.1 to 6.7)	36.9 (28.7 to 47)	5.3 (4.1 to 6.7)	36.9 (28.7 to 47)	-0.13 (-0.14 to -0.13)
New Zealand	0.5 (0.4 to 0.6)	14.6 (11.5 to 18.3)	0.5 (0.4 to 0.6)	14.6 (11.5 to 18.3)	-0.12 (-0.14 to -0.10)
Nicaragua	1.7 (1.2 to 2.3)	33.5 (25.2 to 42.6)	1.7 (1.2 to 2.3)	33.5 (25.2 to 42.6)	-0.44 (-0.68 to -0.20)
Niger	15.2 (12.4 to 18.1)	145.6 (117.9 to 177.5)	15.2 (12.4 to 18.1)	145.6 (117.9 to 177.5)	-0.41 (-0.56 to -0.25)
Nigeria	70 (54.4 to 87.8)	69.1 (54.9 to 86.2)	70 (54.4 to 87.8)	69.1 (54.9 to 86.2)	-1.56 (-2.03 to -1.09)
Niue	0 (0 to 0)	4.8 (3.6 to 6.3)	0 (0 to 0)	4.8 (3.6 to 6.3)	-1.66 (-1.89 to -1.43)
North Macedonia	0.3 (0.2 to 0.4)	15 (11.2 to 18.8)	0.3 (0.2 to 0.4)	15 (11.2 to 18.8)	-1.24 (-1.33 to -1.15)
Northern Mariana Islands	0 (0 to 0)	3.8 (2.8 to 5)	0 (0 to 0)	3.8 (2.8 to 5)	-0.98 (-1.14 to -0.81)
Norway	1.6 (1.2 to 2)	39.6 (31.1 to 50.2)	1.6 (1.2 to 2)	39.6 (31.1 to 50.2)	-0.13 (-0.15 to -0.12)
Oman	0.6 (0.5 to 0.8)	27.9 (21.9 to 35)	0.6 (0.5 to 0.8)	27.9 (21.9 to 35)	-1.15 (-1.24 to -1.05)
Pakistan	285.4 (226.6 to 354.9)	216.2 (170 to 269.3)	285.4 (226.6 to 354.9)	216.2 (170 to 269.3)	0.64 (0.56 to 0.71)
Palau	0 (0 to 0)	4.5 (3.3 to 5.9)	0 (0 to 0)	4.5 (3.3 to 5.9)	-1.43 (-1.61 to -1.26)

Continued on next page

Table S10 – continued from previous page

Country	1990		2019		1990–2019
	Incident cases No.×10^4[95% UI]	ASR per 10,0000 No.[95% UI]	Incident cases No.×10^4[95% UI]	ASR per 10,0000 No.[95% UI]	EAPC No.[95% CI]
Palestine	0.8 (0.6 to 1)	31.3 (24.7 to 39.2)	0.8 (0.6 to 1)	31.3 (24.7 to 39.2)	-1.16 (-1.22 to -1.10)
Panama	0.5 (0.4 to 0.7)	19.3 (14.6 to 24.8)	0.5 (0.4 to 0.7)	19.3 (14.6 to 24.8)	-1.49 (-1.69 to -1.30)
Papua New Guinea	0.3 (0.2 to 0.4)	7.1 (5.4 to 9.3)	0.3 (0.2 to 0.4)	7.1 (5.4 to 9.3)	-2.29 (-2.65 to -1.93)
Paraguay	0.4 (0.3 to 0.5)	8.6 (6.6 to 11)	0.4 (0.3 to 0.5)	8.6 (6.6 to 11)	-0.90 (-0.96 to -0.85)
Peru	1.2 (0.9 to 1.6)	5.3 (4 to 6.9)	1.2 (0.9 to 1.6)	5.3 (4 to 6.9)	-0.58 (-0.66 to -0.49)
Philippines	54.1 (42.3 to 68.4)	73.7 (58.4 to 92.4)	54.1 (42.3 to 68.4)	73.7 (58.4 to 92.4)	1.32 (0.30 to 2.34)
Poland	4.7 (3.6 to 5.8)	12.6 (9.6 to 15.7)	4.7 (3.6 to 5.8)	12.6 (9.6 to 15.7)	-0.67 (-0.72 to -0.61)
Portugal	5.2 (4.1 to 6.6)	53.6 (41.7 to 66.9)	5.2 (4.1 to 6.6)	53.6 (41.7 to 66.9)	0.09 (0.03 to 0.14)
Puerto Rico	0.5 (0.4 to 0.7)	14.7 (11.4 to 18.9)	0.5 (0.4 to 0.7)	14.7 (11.4 to 18.9)	-2.35 (-2.43 to -2.26)
Qatar	0.1 (0.1 to 0.1)	23.3 (18.3 to 29.1)	0.1 (0.1 to 0.1)	23.3 (18.3 to 29.1)	-1.12 (-1.23 to -1.01)
Republic of Korea	9 (7 to 11.5)	19.1 (15 to 24)	9 (7 to 11.5)	19.1 (15 to 24)	-1.12 (-1.18 to -1.06)
Republic of Moldova	0.2 (0.1 to 0.2)	4.1 (3 to 5.4)	0.2 (0.1 to 0.2)	4.1 (3 to 5.4)	0.40 (-0.18 to 0.98)
Romania	5.3 (4.5 to 6.3)	23.8 (20.2 to 28.3)	5.3 (4.5 to 6.3)	23.8 (20.2 to 28.3)	-0.46 (-0.58 to -0.34)
Russian Federation	12.4 (9.4 to 15.7)	8.8 (6.6 to 11.4)	12.4 (9.4 to 15.7)	8.8 (6.6 to 11.4)	-0.32 (-0.71 to 0.07)
Rwanda	14.9 (11.7 to 17.9)	162.6 (131 to 198)	14.9 (11.7 to 17.9)	162.6 (131 to 198)	-1.85 (-2.21 to -1.49)
Saint Kitts and Nevis	0 (0 to 0)	21.2 (16.4 to 27.1)	0 (0 to 0)	21.2 (16.4 to 27.1)	-2.90 (-2.97 to -2.83)
Saint Lucia	0 (0 to 0)	11 (8.5 to 14.1)	0 (0 to 0)	11 (8.5 to 14.1)	-1.37 (-1.43 to -1.32)
Saint Vincent and the Grenadines	0 (0 to 0.1)	30.7 (23.5 to 39.2)	0 (0 to 0.1)	30.7 (23.5 to 39.2)	-2.91 (-3.06 to -2.76)
Samoa	0 (0 to 0)	3.6 (2.6 to 4.7)	0 (0 to 0)	3.6 (2.6 to 4.7)	-1.10 (-1.15 to -1.05)
San Marino	0 (0 to 0)	36 (27.9 to 46.6)	0 (0 to 0)	36 (27.9 to 46.6)	0.04 (0.02 to 0.06)
Sao Tome and Principe	0.1 (0.1 to 0.1)	51.2 (40.3 to 64.6)	0.1 (0.1 to 0.1)	51.2 (40.3 to 64.6)	-0.90 (-1.11 to -0.69)
Saudi Arabia	2.2 (1.6 to 2.8)	12 (9.3 to 14.9)	2.2 (1.6 to 2.8)	12 (9.3 to 14.9)	-1.22 (-1.33 to -1.10)
Senegal	11.1 (9.2 to 13)	122.9 (102.7 to 145.3)	11.1 (9.2 to 13)	122.9 (102.7 to 145.3)	-1.90 (-2.11 to -1.68)
Serbia	1 (0.8 to 1.3)	11.4 (8.7 to 14.5)	1 (0.8 to 1.3)	11.4 (8.7 to 14.5)	-1.39 (-1.51 to -1.26)
Seychelles	0 (0 to 0)	32.6 (24.9 to 41.8)	0 (0 to 0)	32.6 (24.9 to 41.8)	-2.19 (-2.25 to -2.12)
Sierra Leone	2.9 (2.2 to 3.8)	72.7 (56.8 to 92.1)	2.9 (2.2 to 3.8)	72.7 (56.8 to 92.1)	-0.89 (-1.30 to -0.48)
Singapore	0.5 (0.4 to 0.7)	16.3 (12.6 to 20.6)	0.5 (0.4 to 0.7)	16.3 (12.6 to 20.6)	-0.58 (-0.62 to -0.54)
Slovakia	0.7 (0.6 to 0.9)	14.2 (10.8 to 18)	0.7 (0.6 to 0.9)	14.2 (10.8 to 18)	-1.31 (-1.42 to -1.20)
Slovenia	0.2 (0.2 to 0.3)	13.6 (10.2 to 17.1)	0.2 (0.2 to 0.3)	13.6 (10.2 to 17.1)	-1.17 (-1.29 to -1.04)
Solomon Islands	0 (0 to 0)	4.9 (3.7 to 6.3)	0 (0 to 0)	4.9 (3.7 to 6.3)	-0.84 (-0.99 to -0.69)
Somalia	54.5 (44.8 to 65.3)	656.5 (527.9 to 793.5)	54.5 (44.8 to 65.3)	656.5 (527.9 to 793.5)	0.31 (0.20 to 0.42)
South Africa	15.9 (12.3 to 20.1)	38.8 (30.5 to 48.2)	15.9 (12.3 to 20.1)	38.8 (30.5 to 48.2)	-0.82 (-0.90 to -0.74)
South Sudan	4.8 (3.7 to 6.2)	70.4 (55.3 to 89.4)	4.8 (3.7 to 6.2)	70.4 (55.3 to 89.4)	0.37 (0.23 to 0.52)
Spain	14.6 (11.3 to 18.8)	38.6 (30.1 to 49.6)	14.6 (11.3 to 18.8)	38.6 (30.1 to 49.6)	-0.13 (-0.17 to -0.09)
Sri Lanka	20.5 (17.5 to 24)	104.2 (88.7 to 121.2)	20.5 (17.5 to 24)	104.2 (88.7 to 121.2)	-3.01 (-3.11 to -2.90)

Continued on next page

Table S10 – continued from previous page

Country	1990		2019		1990–2019
	Incident cases No.×10^4[95% UI]	ASR per 10,0000 No.[95% UI]	Incident cases No.×10^4[95% UI]	ASR per 10,0000 No.[95% UI]	EAPC No.[95% CI]
Sudan	23.2 (18.3 to 28.6)	90.9 (73.4 to 110.9)	23.2 (18.3 to 28.6)	90.9 (73.4 to 110.9)	-2.57 (-3.01 to -2.14)
Suriname	0.1 (0.1 to 0.1)	24.3 (18.8 to 30.9)	0.1 (0.1 to 0.1)	24.3 (18.8 to 30.9)	-2.83 (-3.19 to -2.46)
Sweden	3.1 (2.5 to 4)	41.8 (32.8 to 53.6)	3.1 (2.5 to 4)	41.8 (32.8 to 53.6)	-0.18 (-0.21 to -0.16)
Switzerland	1.8 (1.4 to 2.2)	28 (22.2 to 35.5)	1.8 (1.4 to 2.2)	28 (22.2 to 35.5)	-0.08 (-0.12 to -0.04)
Syrian Arab Republic	12.2 (9.2 to 15.4)	72.2 (58 to 89.4)	12.2 (9.2 to 15.4)	72.2 (58 to 89.4)	-1.34 (-1.62 to -1.06)
Taiwan (Province of China)	2 (1.5 to 2.6)	9.1 (7 to 11.7)	2 (1.5 to 2.6)	9.1 (7 to 11.7)	-1.71 (-1.80 to -1.63)
Tajikistan	2.3 (1.7 to 3)	33.1 (25 to 42.8)	2.3 (1.7 to 3)	33.1 (25 to 42.8)	-1.30 (-2.10 to -0.50)
Thailand	39.8 (30.4 to 50.9)	59 (45.8 to 75.3)	39.8 (30.4 to 50.9)	59 (45.8 to 75.3)	-2.44 (-2.75 to -2.12)
Timor-Leste	0.4 (0.3 to 0.5)	45.7 (34.9 to 58.5)	0.4 (0.3 to 0.5)	45.7 (34.9 to 58.5)	-3.08 (-3.33 to -2.82)
Togo	6.4 (5.7 to 7.1)	141.9 (125.6 to 159.4)	6.4 (5.7 to 7.1)	141.9 (125.6 to 159.4)	-2.04 (-2.36 to -1.73)
Tokelau	0 (0 to 0)	6.2 (4.7 to 8.2)	0 (0 to 0)	6.2 (4.7 to 8.2)	-2.44 (-2.71 to -2.18)
Tonga	0 (0 to 0)	6.1 (4.6 to 8)	0 (0 to 0)	6.1 (4.6 to 8)	-2.00 (-2.22 to -1.79)
Trinidad and Tobago	0.5 (0.4 to 0.7)	38.4 (29.4 to 48.9)	0.5 (0.4 to 0.7)	38.4 (29.4 to 48.9)	-3.63 (-4.03 to -3.23)
Tunisia	2.5 (2 to 3.1)	27.3 (21.7 to 33.5)	2.5 (2 to 3.1)	27.3 (21.7 to 33.5)	-1.56 (-1.66 to -1.47)
Turkey	68.6 (53.3 to 85)	97.4 (76.8 to 118.9)	68.6 (53.3 to 85)	97.4 (76.8 to 118.9)	-2.93 (-3.21 to -2.65)
Turkmenistan	0.8 (0.6 to 1.1)	18.7 (14 to 24.6)	0.8 (0.6 to 1.1)	18.7 (14 to 24.6)	-0.80 (-0.89 to -0.71)
Tuvalu	0 (0 to 0)	8 (6.2 to 10.4)	0 (0 to 0)	8 (6.2 to 10.4)	-2.56 (-2.74 to -2.38)
Uganda	13.7 (10.5 to 17.8)	69.1 (54.5 to 86.7)	13.7 (10.5 to 17.8)	69.1 (54.5 to 86.7)	-1.59 (-1.68 to -1.50)
Ukraine	10.6 (8.1 to 13.7)	24 (18.3 to 31.7)	10.6 (8.1 to 13.7)	24 (18.3 to 31.7)	-0.09 (-0.62 to 0.44)
United Arab Emirates	1.4 (1.1 to 1.7)	69.3 (54.3 to 86.4)	1.4 (1.1 to 1.7)	69.3 (54.3 to 86.4)	-1.21 (-1.40 to -1.02)
United Kingdom	21 (16.5 to 26.8)	40.1 (31.4 to 50.9)	21 (16.5 to 26.8)	40.1 (31.4 to 50.9)	-0.09 (-0.12 to -0.05)
United Republic of Tanzania	9.2 (7 to 12)	32.6 (25.6 to 41.3)	9.2 (7 to 12)	32.6 (25.6 to 41.3)	-1.33 (-1.52 to -1.14)
United States of America	33.2 (26.3 to 41.5)	13.7 (10.7 to 17.1)	33.2 (26.3 to 41.5)	13.7 (10.7 to 17.1)	-0.01 (-0.03 to 0.01)
United States Virgin Islands	0 (0 to 0)	15.2 (11.8 to 19.4)	0 (0 to 0)	15.2 (11.8 to 19.4)	-2.75 (-3.00 to -2.49)
Uruguay	0.3 (0.2 to 0.4)	10.4 (7.9 to 13.4)	0.3 (0.2 to 0.4)	10.4 (7.9 to 13.4)	-0.72 (-0.77 to -0.68)
Uzbekistan	12.5 (9.2 to 15.8)	46.7 (34.9 to 58.6)	12.5 (9.2 to 15.8)	46.7 (34.9 to 58.6)	-2.70 (-3.10 to -2.30)
Vanuatu	0.1 (0 to 0.1)	31.4 (24 to 41.6)	0.1 (0 to 0.1)	31.4 (24 to 41.6)	-1.00 (-1.11 to -0.90)
Venezuela (Bolivarian Republic of)	5.1 (3.8 to 6.6)	23.1 (17.6 to 29.5)	5.1 (3.8 to 6.6)	23.1 (17.6 to 29.5)	0.04 (-0.08 to 0.16)
Viet Nam	93 (69.5 to 119.7)	116.5 (87.7 to 149)	93 (69.5 to 119.7)	116.5 (87.7 to 149)	-4.14 (-4.19 to -4.08)
Yemen	19.4 (14.6 to 24.6)	98.9 (78.5 to 121.4)	19.4 (14.6 to 24.6)	98.9 (78.5 to 121.4)	-0.55 (-1.03 to -0.07)
Zambia	6.8 (5.1 to 8.8)	72.8 (57 to 92.8)	6.8 (5.1 to 8.8)	72.8 (57 to 92.8)	-1.56 (-1.88 to -1.23)
Zimbabwe	21.8 (19.8 to 24)	165.9 (149.4 to 183.4)	21.8 (19.8 to 24)	165.9 (149.4 to 183.4)	-0.26 (-0.59 to 0.06)

Abbreviation: ASR, age standardized rate; CI, confidence interval; EAPC, estimated annual percentage change; UI, uncertainty interval.

Table S11: The change of iodine deficiency cases between 1990 and 2019 at national level for female.

Country	1990		2019		1990–2019
	Incident cases No.×10^4[95% UI]	ASR per 10,0000 No.[95% UI]	Incident cases No.×10^4[95% UI]	ASR per 10,0000 No.[95% UI]	EAPC No.[95% CI]
Afghanistan	5.9 (4.4 to 7.4)	83.4 (65.2 to 103.4)	5.9 (4.4 to 7.4)	83.4 (65.2 to 103.4)	-0.85 (-1.69 to 0.00)
Albania	0.4 (0.3 to 0.5)	21.5 (16.4 to 27.2)	0.4 (0.3 to 0.5)	21.5 (16.4 to 27.2)	-1.83 (-1.99 to -1.66)
Algeria	6.8 (5.2 to 8.7)	45.2 (35.5 to 56.5)	6.8 (5.2 to 8.7)	45.2 (35.5 to 56.5)	-1.07 (-1.21 to -0.93)
American Samoa	0 (0 to 0)	4.3 (3.2 to 5.8)	0 (0 to 0)	4.3 (3.2 to 5.8)	-1.00 (-1.08 to -0.91)
Andorra	0 (0 to 0)	43.5 (33.3 to 55.6)	0 (0 to 0)	43.5 (33.3 to 55.6)	0.10 (0.08 to 0.13)
Angola	49.3 (39.5 to 60.1)	713.9 (580.1 to 867.6)	49.3 (39.5 to 60.1)	713.9 (580.1 to 867.6)	-3.31 (-3.80 to -2.82)
Antigua and Barbuda	0 (0 to 0)	21 (16.3 to 26.5)	0 (0 to 0)	21 (16.3 to 26.5)	-2.43 (-2.57 to -2.29)
Argentina	1.8 (1.3 to 2.3)	10.5 (7.9 to 13.4)	1.8 (1.3 to 2.3)	10.5 (7.9 to 13.4)	-0.52 (-0.55 to -0.50)
Armenia	1.8 (1.4 to 2.3)	103.2 (80.3 to 131.7)	1.8 (1.4 to 2.3)	103.2 (80.3 to 131.7)	-2.17 (-2.73 to -1.62)
Australia	1.2 (0.9 to 1.5)	14.5 (11.3 to 18.4)	1.2 (0.9 to 1.5)	14.5 (11.3 to 18.4)	-0.24 (-0.26 to -0.22)
Austria	1.7 (1.3 to 2.2)	47.7 (36.5 to 61)	1.7 (1.3 to 2.2)	47.7 (36.5 to 61)	-0.07 (-0.08 to -0.06)
Azerbaijan	1.1 (0.8 to 1.4)	26.4 (19.7 to 33.7)	1.1 (0.8 to 1.4)	26.4 (19.7 to 33.7)	-1.64 (-2.16 to -1.11)
Bahamas	0 (0 to 0)	14.2 (10.9 to 18.1)	0 (0 to 0)	14.2 (10.9 to 18.1)	-1.68 (-1.82 to -1.55)
Bahrain	0.1 (0.1 to 0.1)	31.8 (25.1 to 40)	0.1 (0.1 to 0.1)	31.8 (25.1 to 40)	-0.79 (-0.85 to -0.73)
Bangladesh	251.8 (225.4 to 282.7)	355.2 (314.8 to 399.2)	251.8 (225.4 to 282.7)	355.2 (314.8 to 399.2)	-1.63 (-2.34 to -0.92)
Barbados	0 (0 to 0)	29.7 (22.6 to 38.5)	0 (0 to 0)	29.7 (22.6 to 38.5)	-0.70 (-0.79 to -0.61)
Belarus	0.6 (0.5 to 0.8)	12.5 (9.3 to 16)	0.6 (0.5 to 0.8)	12.5 (9.3 to 16)	-0.98 (-1.30 to -0.66)
Belgium	2.1 (1.6 to 2.7)	47.3 (36.6 to 62.1)	2.1 (1.6 to 2.7)	47.3 (36.6 to 62.1)	-0.15 (-0.17 to -0.14)
Belize	0 (0 to 0)	10.3 (8 to 13.1)	0 (0 to 0)	10.3 (8 to 13.1)	-1.57 (-1.63 to -1.50)
Benin	2 (1.5 to 2.6)	72.4 (55.9 to 93.1)	2 (1.5 to 2.6)	72.4 (55.9 to 93.1)	-0.83 (-0.91 to -0.74)
Bermuda	0 (0 to 0)	12.6 (9.7 to 16.4)	0 (0 to 0)	12.6 (9.7 to 16.4)	-2.25 (-2.41 to -2.08)
Bhutan	0.3 (0.2 to 0.3)	73.7 (55.1 to 95.8)	0.3 (0.2 to 0.3)	73.7 (55.1 to 95.8)	-1.11 (-1.60 to -0.62)
Bolivia (Plurinational State of)	0.2 (0.2 to 0.3)	7.2 (5.7 to 8.9)	0.2 (0.2 to 0.3)	7.2 (5.7 to 8.9)	-0.88 (-0.94 to -0.81)
Bosnia and Herzegovina	1 (0.8 to 1.3)	49 (37.4 to 61)	1 (0.8 to 1.3)	49 (37.4 to 61)	-2.54 (-2.96 to -2.12)
Botswana	0.2 (0.1 to 0.2)	23.6 (18.6 to 29.5)	0.2 (0.1 to 0.2)	23.6 (18.6 to 29.5)	-0.34 (-0.40 to -0.28)
Brazil	6.2 (4.6 to 8.1)	7.7 (5.9 to 9.8)	6.2 (4.6 to 8.1)	7.7 (5.9 to 9.8)	-0.33 (-0.36 to -0.30)
Brunei Darussalam	0 (0 to 0)	16.6 (13 to 20.9)	0 (0 to 0)	16.6 (13 to 20.9)	-0.20 (-0.23 to -0.18)
Bulgaria	0.7 (0.5 to 0.9)	18.7 (13.9 to 24.1)	0.7 (0.5 to 0.9)	18.7 (13.9 to 24.1)	-1.41 (-1.49 to -1.34)
Burkina Faso	5 (3.8 to 6.4)	90.1 (69.6 to 115)	5 (3.8 to 6.4)	90.1 (69.6 to 115)	-1.79 (-1.91 to -1.66)
Burundi	7.6 (6.7 to 8.5)	218 (192.2 to 246.3)	7.6 (6.7 to 8.5)	218 (192.2 to 246.3)	-2.04 (-2.17 to -1.90)
Cabo Verde	0.3 (0.2 to 0.3)	121 (93.9 to 152.3)	0.3 (0.2 to 0.3)	121 (93.9 to 152.3)	-1.34 (-1.48 to -1.19)
Cambodia	11.6 (8.9 to 14.8)	192.4 (146.1 to 247)	11.6 (8.9 to 14.8)	192.4 (146.1 to 247)	-5.78 (-6.28 to -5.28)
Cameroon	2.8 (2.1 to 3.7)	47.8 (36.9 to 62.3)	2.8 (2.1 to 3.7)	47.8 (36.9 to 62.3)	-0.24 (-0.36 to -0.11)

Continued on next page

Table S11 – continued from previous page

Country	1990		2019		1990–2019
	Incident cases No.×10^4[95% UI]	ASR per 10,0000 No.[95% UI]	Incident cases No.×10^4[95% UI]	ASR per 10,0000 No.[95% UI]	EAPC No.[95% CI]
Canada	2 (1.6 to 2.6)	15.5 (12.1 to 19.5)	2 (1.6 to 2.6)	15.5 (12.1 to 19.5)	-0.21 (-0.22 to -0.20)
Central African Republic	7.8 (6.8 to 8.7)	437.3 (383.8 to 490.6)	7.8 (6.8 to 8.7)	437.3 (383.8 to 490.6)	-1.78 (-2.08 to -1.48)
Chad	4.4 (3.4 to 5.6)	118.8 (92.7 to 150.1)	4.4 (3.4 to 5.6)	118.8 (92.7 to 150.1)	-2.39 (-2.55 to -2.24)
Chile	1 (0.7 to 1.2)	14.2 (10.8 to 17.5)	1 (0.7 to 1.2)	14.2 (10.8 to 17.5)	-1.76 (-1.91 to -1.60)
China	486.1 (369.1 to 629.4)	74.4 (57.3 to 95.4)	486.1 (369.1 to 629.4)	74.4 (57.3 to 95.4)	0.69 (0.32 to 1.06)
Colombia	6.1 (4.5 to 7.9)	32.3 (24.4 to 41.6)	6.1 (4.5 to 7.9)	32.3 (24.4 to 41.6)	-0.38 (-0.49 to -0.26)
Comoros	0.1 (0.1 to 0.1)	27.3 (21.1 to 34.6)	0.1 (0.1 to 0.1)	27.3 (21.1 to 34.6)	-0.15 (-0.20 to -0.10)
Congo	13.7 (11.3 to 15.9)	795.3 (663 to 923.8)	13.7 (11.3 to 15.9)	795.3 (663 to 923.8)	-1.35 (-1.46 to -1.23)
Cook Islands	0 (0 to 0)	4.9 (3.7 to 6.6)	0 (0 to 0)	4.9 (3.7 to 6.6)	-1.20 (-1.32 to -1.08)
Costa Rica	0.5 (0.4 to 0.7)	30.4 (23.1 to 38.5)	0.5 (0.4 to 0.7)	30.4 (23.1 to 38.5)	-0.42 (-0.49 to -0.35)
Croatia	0.4 (0.3 to 0.5)	17.5 (13.4 to 22.2)	0.4 (0.3 to 0.5)	17.5 (13.4 to 22.2)	-1.11 (-1.23 to -0.99)
Cuba	1.6 (1.2 to 2.1)	29.9 (22.9 to 38.2)	1.6 (1.2 to 2.1)	29.9 (22.9 to 38.2)	-2.18 (-2.72 to -1.64)
Cyprus	0.2 (0.1 to 0.2)	50.1 (38.8 to 65.1)	0.2 (0.1 to 0.2)	50.1 (38.8 to 65.1)	-0.12 (-0.13 to -0.10)
Czechia	0.6 (0.5 to 0.8)	13.5 (10.2 to 17.1)	0.6 (0.5 to 0.8)	13.5 (10.2 to 17.1)	-1.03 (-1.16 to -0.89)
Ivoirian	9.1 (6.7 to 11.6)	123.8 (94.5 to 156.7)	9.1 (6.7 to 11.6)	123.8 (94.5 to 156.7)	-2.03 (-2.22 to -1.84)
Democratic People's Republic of Korea	0.8 (0.6 to 1)	6.8 (5.2 to 8.8)	0.8 (0.6 to 1)	6.8 (5.2 to 8.8)	-1.36 (-1.56 to -1.17)
Democratic Republic of the Congo	232.1 (212.3 to 253.9)	892.2 (814.2 to 984.9)	232.1 (212.3 to 253.9)	892.2 (814.2 to 984.9)	-0.85 (-1.14 to -0.56)
Denmark	0.9 (0.7 to 1.1)	38.3 (29.4 to 49.1)	0.9 (0.7 to 1.1)	38.3 (29.4 to 49.1)	-0.15 (-0.21 to -0.10)
Djibouti	1.7 (1.4 to 2.1)	610.9 (483.6 to 751.2)	1.7 (1.4 to 2.1)	610.9 (483.6 to 751.2)	-0.36 (-0.73 to 0.01)
Dominica	0 (0 to 0)	32.7 (24.7 to 41.5)	0 (0 to 0)	32.7 (24.7 to 41.5)	-2.54 (-2.73 to -2.36)
Dominican Republic	1.8 (1.3 to 2.3)	40.4 (31.2 to 51.3)	1.8 (1.3 to 2.3)	40.4 (31.2 to 51.3)	-2.45 (-2.57 to -2.33)
Ecuador	0.3 (0.2 to 0.4)	5.8 (4.4 to 7.4)	0.3 (0.2 to 0.4)	5.8 (4.4 to 7.4)	-0.47 (-0.54 to -0.40)
Egypt	37.6 (28.4 to 48.8)	112.4 (87.2 to 142.7)	37.6 (28.4 to 48.8)	112.4 (87.2 to 142.7)	-2.93 (-3.17 to -2.69)
El Salvador	1.3 (1 to 1.7)	39.3 (29.3 to 49.5)	1.3 (1 to 1.7)	39.3 (29.3 to 49.5)	-0.80 (-0.88 to -0.71)
Equatorial Guinea	4.3 (3.6 to 5)	1387.4 (1193.1 to 1571.3)	4.3 (3.6 to 5)	1387.4 (1193.1 to 1571.3)	-6.99 (-7.80 to -6.17)
Eritrea	0.8 (0.6 to 1)	47.5 (36.5 to 61.9)	0.8 (0.6 to 1)	47.5 (36.5 to 61.9)	-1.18 (-1.37 to -0.99)
Estonia	0.1 (0 to 0.1)	8.6 (6.4 to 11.2)	0.1 (0 to 0.1)	8.6 (6.4 to 11.2)	-0.80 (-0.88 to -0.72)
Eswatini	0.3 (0.3 to 0.4)	65.7 (51.4 to 83.3)	0.3 (0.3 to 0.4)	65.7 (51.4 to 83.3)	-0.73 (-0.77 to -0.69)
Ethiopia	210.8 (170.5 to 256.8)	671.7 (539.6 to 824.1)	210.8 (170.5 to 256.8)	671.7 (539.6 to 824.1)	-1.04 (-1.46 to -0.63)
Fiji	0 (0 to 0)	6.2 (4.6 to 8.1)	0 (0 to 0)	6.2 (4.6 to 8.1)	-1.62 (-1.80 to -1.43)
Finland	1.1 (0.8 to 1.3)	47.7 (36.4 to 60.5)	1.1 (0.8 to 1.3)	47.7 (36.4 to 60.5)	-0.13 (-0.16 to -0.10)
France	12.6 (9.8 to 16.1)	47.1 (36.6 to 60.4)	12.6 (9.8 to 16.1)	47.1 (36.6 to 60.4)	0.01 (0.00 to 0.03)
Gabon	2.7 (2.1 to 3.3)	425.7 (342.4 to 524.4)	2.7 (2.1 to 3.3)	425.7 (342.4 to 524.4)	-4.87 (-5.51 to -4.23)
Gambia	1.5 (1.2 to 1.9)	244.4 (192.6 to 309.9)	1.5 (1.2 to 1.9)	244.4 (192.6 to 309.9)	-1.39 (-1.72 to -1.07)
Georgia	0.8 (0.6 to 1.1)	32.7 (24.6 to 41.5)	0.8 (0.6 to 1.1)	32.7 (24.6 to 41.5)	-0.57 (-0.66 to -0.49)

Continued on next page

Table S11 – continued from previous page

Country	1990		2019		1990–2019
	Incident cases No.$\times 10^4$[95% UI]	ASR per 10,0000 No.[95% UI]	Incident cases No.$\times 10^4$[95% UI]	ASR per 10,0000 No.[95% UI]	EAPC No.[95% CI]
Germany	16.4 (12.7 to 21.1)	47.2 (36.3 to 60.5)	16.4 (12.7 to 21.1)	47.2 (36.3 to 60.5)	-0.09 (-0.11 to -0.07)
Ghana	22.4 (17.6 to 28.4)	247.9 (193.7 to 316.6)	22.4 (17.6 to 28.4)	247.9 (193.7 to 316.6)	-2.79 (-3.02 to -2.56)
Greece	2.5 (1.9 to 3.1)	52.4 (40.4 to 66.2)	2.5 (1.9 to 3.1)	52.4 (40.4 to 66.2)	-0.35 (-0.44 to -0.27)
Greenland	0 (0 to 0)	14.6 (11.2 to 18.4)	0 (0 to 0)	14.6 (11.2 to 18.4)	-0.04 (-0.07 to -0.02)
Grenada	0 (0 to 0)	33.7 (25.7 to 43.6)	0 (0 to 0)	33.7 (25.7 to 43.6)	-3.21 (-3.35 to -3.07)
Guam	0 (0 to 0)	4.3 (3.2 to 5.7)	0 (0 to 0)	4.3 (3.2 to 5.7)	-0.88 (-1.00 to -0.76)
Guatemala	1.6 (1.2 to 2.1)	31.9 (24 to 40.9)	1.6 (1.2 to 2.1)	31.9 (24 to 40.9)	-0.11 (-0.18 to -0.03)
Guinea	12.1 (9.5 to 14.9)	307.9 (245.5 to 380.7)	12.1 (9.5 to 14.9)	307.9 (245.5 to 380.7)	-3.33 (-3.63 to -3.03)
Guinea-Bissau	2.2 (1.7 to 2.7)	342.5 (273.6 to 429)	2.2 (1.7 to 2.7)	342.5 (273.6 to 429)	-1.69 (-1.89 to -1.49)
Guyana	0.2 (0.2 to 0.3)	48.7 (37 to 61.7)	0.2 (0.2 to 0.3)	48.7 (37 to 61.7)	-2.14 (-2.31 to -1.98)
Haiti	2.9 (2.2 to 3.5)	70.8 (54.5 to 87.1)	2.9 (2.2 to 3.5)	70.8 (54.5 to 87.1)	-0.98 (-1.26 to -0.69)
Honduras	1.3 (1 to 1.7)	42.5 (32.2 to 54.3)	1.3 (1 to 1.7)	42.5 (32.2 to 54.3)	-0.42 (-0.59 to -0.25)
Hungary	0.9 (0.7 to 1.1)	19.3 (14.7 to 24.5)	0.9 (0.7 to 1.1)	19.3 (14.7 to 24.5)	-1.56 (-1.68 to -1.44)
Iceland	0 (0 to 0)	22.1 (17.2 to 28.3)	0 (0 to 0)	22.1 (17.2 to 28.3)	-0.09 (-0.15 to -0.03)
India	1969.8 (1570.5 to 2435.5)	398.7 (318.8 to 495.7)	1969.8 (1570.5 to 2435.5)	398.7 (318.8 to 495.7)	-1.42 (-1.81 to -1.04)
Indonesia	52.2 (38.9 to 67.7)	48.7 (37.1 to 62.8)	52.2 (38.9 to 67.7)	48.7 (37.1 to 62.8)	-2.29 (-2.37 to -2.20)
Iran (Islamic Republic of)	9.7 (7.3 to 12.4)	28.9 (22.5 to 35.7)	9.7 (7.3 to 12.4)	28.9 (22.5 to 35.7)	-1.48 (-1.68 to -1.27)
Iraq	8.4 (6.4 to 10.5)	76.7 (60.5 to 94.6)	8.4 (6.4 to 10.5)	76.7 (60.5 to 94.6)	-1.61 (-2.11 to -1.11)
Ireland	0.9 (0.7 to 1.2)	50.1 (38.7 to 64.1)	0.9 (0.7 to 1.2)	50.1 (38.7 to 64.1)	-0.31 (-0.35 to -0.28)
Israel	1.3 (1 to 1.6)	49.8 (38.5 to 63.8)	1.3 (1 to 1.6)	49.8 (38.5 to 63.8)	-0.11 (-0.13 to -0.09)
Italy	64.9 (49.3 to 83.6)	258.5 (199.8 to 326.1)	64.9 (49.3 to 83.6)	258.5 (199.8 to 326.1)	-1.83 (-2.18 to -1.47)
Jamaica	0.4 (0.3 to 0.6)	32.2 (24.7 to 40.4)	0.4 (0.3 to 0.6)	32.2 (24.7 to 40.4)	-1.87 (-2.02 to -1.72)
Japan	11.2 (8.9 to 14.1)	19 (14.9 to 23.8)	11.2 (8.9 to 14.1)	19 (14.9 to 23.8)	-0.34 (-0.37 to -0.32)
Jordan	0.9 (0.7 to 1.2)	41.4 (32.7 to 52)	0.9 (0.7 to 1.2)	41.4 (32.7 to 52)	-1.05 (-1.15 to -0.96)
Kazakhstan	5 (3.8 to 6.5)	57.3 (43.5 to 74)	5 (3.8 to 6.5)	57.3 (43.5 to 74)	-2.55 (-2.92 to -2.19)
Kenya	8.8 (6.7 to 11.3)	63.1 (49.6 to 79.8)	8.8 (6.7 to 11.3)	63.1 (49.6 to 79.8)	-0.03 (-0.32 to 0.26)
Kiribati	0 (0 to 0)	8.7 (6.6 to 11.3)	0 (0 to 0)	8.7 (6.6 to 11.3)	-1.83 (-2.19 to -1.47)
Kuwait	0.3 (0.2 to 0.3)	30.2 (23.6 to 38.1)	0.3 (0.2 to 0.3)	30.2 (23.6 to 38.1)	-0.79 (-0.86 to -0.72)
Kyrgyzstan	0.8 (0.6 to 1.1)	31.9 (24.3 to 41.2)	0.8 (0.6 to 1.1)	31.9 (24.3 to 41.2)	-0.25 (-0.29 to -0.21)
Lao People's Democratic Republic	1.2 (0.9 to 1.5)	49.6 (37.3 to 65)	1.2 (0.9 to 1.5)	49.6 (37.3 to 65)	-2.09 (-2.20 to -1.99)
Latvia	0.1 (0.1 to 0.1)	8.4 (6.2 to 11.1)	0.1 (0.1 to 0.1)	8.4 (6.2 to 11.1)	-0.64 (-0.84 to -0.43)
Lebanon	2.3 (2 to 2.7)	123.9 (107 to 143.7)	2.3 (2 to 2.7)	123.9 (107 to 143.7)	-2.18 (-2.61 to -1.76)
Lesotho	7.1 (6.5 to 7.7)	585.5 (528.7 to 644.5)	7.1 (6.5 to 7.7)	585.5 (528.7 to 644.5)	-3.94 (-4.74 to -3.15)
Liberia	0.5 (0.4 to 0.7)	47.8 (36.9 to 61.1)	0.5 (0.4 to 0.7)	47.8 (36.9 to 61.1)	-1.02 (-1.43 to -0.62)
Libya	1.3 (1 to 1.7)	51.9 (40.9 to 64.3)	1.3 (1 to 1.7)	51.9 (40.9 to 64.3)	0.12 (-0.22 to 0.46)

Continued on next page

Table S11 – continued from previous page

Country	1990		2019		1990–2019
	Incident cases No.×10^4[95% UI]	ASR per 10,0000 No.[95% UI]	Incident cases No.×10^4[95% UI]	ASR per 10,0000 No.[95% UI]	EAPC No.[95% CI]
Lithuania	0.1 (0.1 to 0.2)	8.2 (6.1 to 10.8)	0.1 (0.1 to 0.2)	8.2 (6.1 to 10.8)	-0.68 (-0.88 to -0.49)
Luxembourg	0.1 (0.1 to 0.1)	45.1 (35.1 to 57.2)	0.1 (0.1 to 0.1)	45.1 (35.1 to 57.2)	-0.02 (-0.03 to -0.01)
Madagascar	7.3 (5.5 to 9.3)	107.8 (82.2 to 137.4)	7.3 (5.5 to 9.3)	107.8 (82.2 to 137.4)	0.30 (0.13 to 0.47)
Malawi	8.2 (6.4 to 10.6)	141.9 (111.4 to 185.7)	8.2 (6.4 to 10.6)	141.9 (111.4 to 185.7)	-1.26 (-1.54 to -0.98)
Malaysia	32.2 (27.3 to 37.7)	312.8 (265.9 to 367.9)	32.2 (27.3 to 37.7)	312.8 (265.9 to 367.9)	-5.78 (-6.05 to -5.50)
Maldives	0.1 (0.1 to 0.1)	58.9 (44.4 to 76.4)	0.1 (0.1 to 0.1)	58.9 (44.4 to 76.4)	-4.58 (-4.75 to -4.40)
Mali	3.8 (3 to 4.9)	78.8 (61.9 to 101)	3.8 (3 to 4.9)	78.8 (61.9 to 101)	-1.55 (-1.65 to -1.45)
Malta	0.1 (0.1 to 0.1)	53.7 (41.8 to 68.7)	0.1 (0.1 to 0.1)	53.7 (41.8 to 68.7)	-0.33 (-0.34 to -0.32)
Marshall Islands	0 (0 to 0)	7.8 (5.9 to 10.1)	0 (0 to 0)	7.8 (5.9 to 10.1)	-1.92 (-2.23 to -1.62)
Mauritania	3.1 (2.5 to 3.9)	251.9 (197.6 to 317.5)	3.1 (2.5 to 3.9)	251.9 (197.6 to 317.5)	-1.58 (-1.83 to -1.34)
Mauritius	0.3 (0.2 to 0.4)	53.6 (40.1 to 69.3)	0.3 (0.2 to 0.4)	53.6 (40.1 to 69.3)	-2.52 (-2.57 to -2.46)
Mexico	30.2 (23 to 38.9)	56 (42.9 to 71.1)	30.2 (23 to 38.9)	56 (42.9 to 71.1)	0.07 (0.03 to 0.10)
Micronesia (Federated States of)	0 (0 to 0)	8 (6 to 10.4)	0 (0 to 0)	8 (6 to 10.4)	-1.94 (-2.19 to -1.68)
Monaco	0 (0 to 0)	40.9 (31.4 to 52.6)	0 (0 to 0)	40.9 (31.4 to 52.6)	0.15 (0.13 to 0.17)
Mongolia	0.9 (0.7 to 1.1)	67.1 (53.1 to 80.1)	0.9 (0.7 to 1.1)	67.1 (53.1 to 80.1)	-3.62 (-3.94 to -3.29)
Montenegro	0.1 (0 to 0.1)	17.6 (13.5 to 22.5)	0.1 (0 to 0.1)	17.6 (13.5 to 22.5)	-1.12 (-1.22 to -1.01)
Morocco	24 (18.6 to 30.5)	152.4 (119.1 to 189.6)	24 (18.6 to 30.5)	152.4 (119.1 to 189.6)	-2.29 (-2.46 to -2.12)
Mozambique	11.1 (8.6 to 14)	133.5 (104.4 to 169.1)	11.1 (8.6 to 14)	133.5 (104.4 to 169.1)	-2.56 (-2.92 to -2.19)
Myanmar	19.7 (14.8 to 25.7)	83.4 (62.9 to 108.8)	19.7 (14.8 to 25.7)	83.4 (62.9 to 108.8)	-3.80 (-4.00 to -3.60)
Namibia	0.4 (0.3 to 0.6)	51.5 (40 to 65.6)	0.4 (0.3 to 0.6)	51.5 (40 to 65.6)	-1.29 (-1.35 to -1.23)
Nauru	0 (0 to 0)	4.8 (3.5 to 6.3)	0 (0 to 0)	4.8 (3.5 to 6.3)	-0.76 (-1.32 to -0.20)
Nepal	10 (7.6 to 13.1)	88.6 (68.1 to 115.6)	10 (7.6 to 13.1)	88.6 (68.1 to 115.6)	1.10 (0.28 to 1.94)
Netherlands	3.2 (2.5 to 4.1)	45.9 (35.6 to 58.6)	3.2 (2.5 to 4.1)	45.9 (35.6 to 58.6)	-0.10 (-0.11 to -0.09)
New Zealand	0.3 (0.2 to 0.3)	16.5 (12.9 to 20.7)	0.3 (0.2 to 0.3)	16.5 (12.9 to 20.7)	-0.13 (-0.15 to -0.11)
Nicaragua	1 (0.7 to 1.4)	40 (29.9 to 51.3)	1 (0.7 to 1.4)	40 (29.9 to 51.3)	-0.44 (-0.68 to -0.19)
Niger	8.7 (7.1 to 10.6)	166.3 (133 to 206.2)	8.7 (7.1 to 10.6)	166.3 (133 to 206.2)	-0.31 (-0.48 to -0.14)
Nigeria	51.1 (39.3 to 64.5)	97.4 (76.8 to 121.4)	51.1 (39.3 to 64.5)	97.4 (76.8 to 121.4)	-1.96 (-2.55 to -1.35)
Niue	0 (0 to 0)	5.5 (4.1 to 7.1)	0 (0 to 0)	5.5 (4.1 to 7.1)	-1.46 (-1.67 to -1.25)
North Macedonia	0.2 (0.1 to 0.2)	18.9 (14.3 to 24)	0.2 (0.1 to 0.2)	18.9 (14.3 to 24)	-1.17 (-1.27 to -1.07)
Northern Mariana Islands	0 (0 to 0)	4.4 (3.2 to 5.7)	0 (0 to 0)	4.4 (3.2 to 5.7)	-0.75 (-0.87 to -0.63)
Norway	1 (0.7 to 1.2)	49.8 (38.9 to 63.1)	1 (0.7 to 1.2)	49.8 (38.9 to 63.1)	-0.08 (-0.10 to -0.06)
Oman	0.3 (0.2 to 0.4)	33.4 (26.1 to 41.4)	0.3 (0.2 to 0.4)	33.4 (26.1 to 41.4)	-0.92 (-1.01 to -0.84)
Pakistan	226.3 (179.7 to 281.5)	360.2 (284.3 to 450.1)	226.3 (179.7 to 281.5)	360.2 (284.3 to 450.1)	0.85 (0.76 to 0.94)
Palau	0 (0 to 0)	5.1 (3.7 to 6.8)	0 (0 to 0)	5.1 (3.7 to 6.8)	-1.17 (-1.32 to -1.02)
Palestine	0.4 (0.3 to 0.6)	36.7 (28.9 to 45.8)	0.4 (0.3 to 0.6)	36.7 (28.9 to 45.8)	-1.01 (-1.08 to -0.94)

Continued on next page

Table S11 – continued from previous page

Country	1990		2019		1990–2019
	Incident cases No.$\times 10^4$[95% UI]	ASR per 10,0000 No.[95% UI]	Incident cases No.$\times 10^4$[95% UI]	ASR per 10,0000 No.[95% UI]	EAPC No.[95% CI]
Panama	0.2 (0.2 to 0.3)	17.6 (13.3 to 22.3)	0.2 (0.2 to 0.3)	17.6 (13.3 to 22.3)	-1.32 (-1.48 to -1.15)
Papua New Guinea	0.2 (0.1 to 0.2)	8.2 (6.2 to 10.6)	0.2 (0.1 to 0.2)	8.2 (6.2 to 10.6)	-2.09 (-2.43 to -1.74)
Paraguay	0.2 (0.2 to 0.3)	9.6 (7.3 to 12.2)	0.2 (0.2 to 0.3)	9.6 (7.3 to 12.2)	-0.86 (-0.91 to -0.80)
Peru	0.7 (0.5 to 0.9)	5.8 (4.4 to 7.5)	0.7 (0.5 to 0.9)	5.8 (4.4 to 7.5)	-0.57 (-0.66 to -0.48)
Philippines	31.2 (24.3 to 39.3)	84.8 (67.1 to 106.4)	31.2 (24.3 to 39.3)	84.8 (67.1 to 106.4)	1.58 (0.49 to 2.69)
Poland	2.9 (2.3 to 3.6)	15.7 (12.1 to 19.7)	2.9 (2.3 to 3.6)	15.7 (12.1 to 19.7)	-0.50 (-0.57 to -0.43)
Portugal	3 (2.3 to 3.9)	62.1 (48 to 78.4)	3 (2.3 to 3.9)	62.1 (48 to 78.4)	0.13 (0.07 to 0.19)
Puerto Rico	0.3 (0.2 to 0.4)	16.6 (12.8 to 21)	0.3 (0.2 to 0.4)	16.6 (12.8 to 21)	-2.39 (-2.47 to -2.30)
Qatar	0 (0 to 0.1)	28.2 (22 to 35.2)	0 (0 to 0.1)	28.2 (22 to 35.2)	-0.84 (-0.92 to -0.75)
Republic of Korea	5 (3.9 to 6.4)	21.5 (16.7 to 27.3)	5 (3.9 to 6.4)	21.5 (16.7 to 27.3)	-1.10 (-1.16 to -1.04)
Republic of Moldova	0.1 (0.1 to 0.2)	5.3 (3.9 to 7)	0.1 (0.1 to 0.2)	5.3 (3.9 to 7)	0.44 (-0.13 to 1.01)
Romania	3 (2.5 to 3.6)	27.2 (22.7 to 32.7)	3 (2.5 to 3.6)	27.2 (22.7 to 32.7)	-1.41 (-1.54 to -1.29)
Russian Federation	9.5 (7.3 to 11.9)	13.5 (10.1 to 17.2)	9.5 (7.3 to 11.9)	13.5 (10.1 to 17.2)	-0.33 (-0.72 to 0.07)
Rwanda	8.7 (6.9 to 10.9)	192.7 (152.2 to 241.5)	8.7 (6.9 to 10.9)	192.7 (152.2 to 241.5)	-1.85 (-2.21 to -1.49)
Saint Kitts and Nevis	0 (0 to 0)	24 (18.4 to 30.2)	0 (0 to 0)	24 (18.4 to 30.2)	-2.92 (-2.99 to -2.85)
Saint Lucia	0 (0 to 0)	12.4 (9.5 to 15.9)	0 (0 to 0)	12.4 (9.5 to 15.9)	-1.39 (-1.44 to -1.33)
Saint Vincent and the Grenadines	0 (0 to 0)	34.9 (26.6 to 45)	0 (0 to 0)	34.9 (26.6 to 45)	-2.93 (-3.08 to -2.78)
Samoa	0 (0 to 0)	4.1 (3 to 5.4)	0 (0 to 0)	4.1 (3 to 5.4)	-0.90 (-0.93 to -0.87)
San Marino	0 (0 to 0)	44.7 (34.4 to 58.2)	0 (0 to 0)	44.7 (34.4 to 58.2)	0.07 (0.04 to 0.10)
Sao Tome and Principe	0 (0 to 0.1)	63.2 (49.2 to 80.5)	0 (0 to 0.1)	63.2 (49.2 to 80.5)	-0.86 (-1.07 to -0.65)
Saudi Arabia	1.1 (0.8 to 1.5)	14 (10.8 to 17.5)	1.1 (0.8 to 1.5)	14 (10.8 to 17.5)	-1.04 (-1.13 to -0.94)
Senegal	6.6 (5.5 to 7.8)	141.5 (116.2 to 166.4)	6.6 (5.5 to 7.8)	141.5 (116.2 to 166.4)	-1.78 (-1.97 to -1.58)
Serbia	0.5 (0.4 to 0.7)	11.6 (9 to 14.9)	0.5 (0.4 to 0.7)	11.6 (9 to 14.9)	-1.41 (-1.56 to -1.26)
Seychelles	0 (0 to 0)	43.1 (32.1 to 55.7)	0 (0 to 0)	43.1 (32.1 to 55.7)	-2.04 (-2.09 to -1.99)
Sierra Leone	1.9 (1.4 to 2.4)	89.5 (69.1 to 113.9)	1.9 (1.4 to 2.4)	89.5 (69.1 to 113.9)	-0.81 (-1.22 to -0.39)
Singapore	0.3 (0.2 to 0.4)	18.4 (14.2 to 23.2)	0.3 (0.2 to 0.4)	18.4 (14.2 to 23.2)	-0.56 (-0.60 to -0.53)
Slovakia	0.5 (0.4 to 0.6)	17.9 (13.8 to 22.8)	0.5 (0.4 to 0.6)	17.9 (13.8 to 22.8)	-1.26 (-1.37 to -1.15)
Slovenia	0.2 (0.1 to 0.2)	17.2 (13 to 21.8)	0.2 (0.1 to 0.2)	17.2 (13 to 21.8)	-1.11 (-1.24 to -0.98)
Solomon Islands	0 (0 to 0)	5.6 (4.2 to 7.3)	0 (0 to 0)	5.6 (4.2 to 7.3)	-0.62 (-0.75 to -0.49)
Somalia	29.7 (24.5 to 35.9)	748.4 (605.7 to 916.4)	29.7 (24.5 to 35.9)	748.4 (605.7 to 916.4)	0.22 (0.11 to 0.34)
South Africa	9.7 (7.5 to 12.3)	45.9 (35.8 to 57.1)	9.7 (7.5 to 12.3)	45.9 (35.8 to 57.1)	-0.80 (-0.89 to -0.71)
South Sudan	2.7 (2.1 to 3.5)	83.3 (64.2 to 106.5)	2.7 (2.1 to 3.5)	83.3 (64.2 to 106.5)	0.32 (0.16 to 0.48)
Spain	9 (6.9 to 11.5)	48.1 (37.1 to 61.8)	9 (6.9 to 11.5)	48.1 (37.1 to 61.8)	-0.08 (-0.12 to -0.03)
Sri Lanka	14.7 (12.6 to 17.1)	149.2 (127.3 to 172.3)	14.7 (12.6 to 17.1)	149.2 (127.3 to 172.3)	-2.95 (-3.04 to -2.85)
Sudan	13.1 (10.2 to 16.4)	103.8 (83.8 to 127.8)	13.1 (10.2 to 16.4)	103.8 (83.8 to 127.8)	-2.48 (-2.89 to -2.07)

Continued on next page

Table S11 – continued from previous page

Country	1990		2019		1990–2019
	Incident cases No.×10^4[95% UI]	ASR per 10,0000 No.[95% UI]	Incident cases No.×10^4[95% UI]	ASR per 10,0000 No.[95% UI]	EAPC No.[95% CI]
Suriname	0.1 (0 to 0.1)	27.7 (21.3 to 35)	0.1 (0 to 0.1)	27.7 (21.3 to 35)	-2.85 (-3.22 to -2.47)
Sweden	1.9 (1.5 to 2.4)	52.3 (40.9 to 67.2)	1.9 (1.5 to 2.4)	52.3 (40.9 to 67.2)	-0.13 (-0.15 to -0.10)
Switzerland	1.1 (0.8 to 1.4)	35 (27.7 to 44.9)	1.1 (0.8 to 1.4)	35 (27.7 to 44.9)	-0.01 (-0.05 to 0.02)
Syrian Arab Republic	7.1 (5.4 to 9.1)	85.5 (67.4 to 106.6)	7.1 (5.4 to 9.1)	85.5 (67.4 to 106.6)	-1.23 (-1.50 to -0.96)
Taiwan (Province of China)	1.2 (0.9 to 1.5)	10.8 (8.3 to 14)	1.2 (0.9 to 1.5)	10.8 (8.3 to 14)	-1.42 (-1.48 to -1.37)
Tajikistan	2.1 (1.6 to 2.8)	60.6 (45.4 to 78.5)	2.1 (1.6 to 2.8)	60.6 (45.4 to 78.5)	-1.28 (-2.07 to -0.47)
Thailand	28.5 (21.7 to 36.8)	83.4 (64.2 to 107.2)	28.5 (21.7 to 36.8)	83.4 (64.2 to 107.2)	-2.54 (-2.92 to -2.17)
Timor-Leste	0.3 (0.2 to 0.3)	61.8 (46.2 to 80.3)	0.3 (0.2 to 0.3)	61.8 (46.2 to 80.3)	-3.13 (-3.39 to -2.88)
Togo	3.9 (3.5 to 4.3)	168.2 (148.8 to 189.5)	3.9 (3.5 to 4.3)	168.2 (148.8 to 189.5)	-1.92 (-2.22 to -1.62)
Tokelau	0 (0 to 0)	7.1 (5.4 to 9.4)	0 (0 to 0)	7.1 (5.4 to 9.4)	-2.23 (-2.47 to -1.98)
Tonga	0 (0 to 0)	7 (5.3 to 9.2)	0 (0 to 0)	7 (5.3 to 9.2)	-1.82 (-2.02 to -1.62)
Trinidad and Tobago	0.3 (0.2 to 0.4)	43.7 (33.2 to 55.9)	0.3 (0.2 to 0.4)	43.7 (33.2 to 55.9)	-3.65 (-4.05 to -3.25)
Tunisia	1.3 (1 to 1.6)	27.6 (21.7 to 34.3)	1.3 (1 to 1.6)	27.6 (21.7 to 34.3)	-1.35 (-1.45 to -1.25)
Turkey	40.1 (30.6 to 50.5)	115.5 (90.4 to 142.7)	40.1 (30.6 to 50.5)	115.5 (90.4 to 142.7)	-2.89 (-3.15 to -2.64)
Turkmenistan	0.8 (0.6 to 1)	33.9 (25.3 to 44.9)	0.8 (0.6 to 1)	33.9 (25.3 to 44.9)	-0.73 (-0.82 to -0.63)
Tuvalu	0 (0 to 0)	9.2 (7 to 11.9)	0 (0 to 0)	9.2 (7 to 11.9)	-2.32 (-2.48 to -2.16)
Uganda	8.3 (6.3 to 10.6)	80.5 (63.1 to 102.1)	8.3 (6.3 to 10.6)	80.5 (63.1 to 102.1)	-1.63 (-1.73 to -1.54)
Ukraine	7.5 (5.8 to 9.8)	34.6 (26.4 to 45.9)	7.5 (5.8 to 9.8)	34.6 (26.4 to 45.9)	-0.04 (-0.55 to 0.48)
United Arab Emirates	0.7 (0.5 to 0.9)	82 (64 to 103)	0.7 (0.5 to 0.9)	82 (64 to 103)	-1.03 (-1.22 to -0.84)
United Kingdom	12.9 (10.1 to 16.5)	49.7 (38.7 to 63.1)	12.9 (10.1 to 16.5)	49.7 (38.7 to 63.1)	0.00 (-0.06 to 0.06)
United Republic of Tanzania	5.6 (4.3 to 7.2)	37.7 (29.4 to 48.3)	5.6 (4.3 to 7.2)	37.7 (29.4 to 48.3)	-1.37 (-1.57 to -1.17)
United States of America	19 (15 to 23.7)	15.7 (12.3 to 19.5)	19 (15 to 23.7)	15.7 (12.3 to 19.5)	0.00 (-0.02 to 0.01)
United States Virgin Islands	0 (0 to 0)	17.2 (13.2 to 22)	0 (0 to 0)	17.2 (13.2 to 22)	-2.79 (-3.05 to -2.53)
Uruguay	0.2 (0.1 to 0.2)	11.1 (8.4 to 14.3)	0.2 (0.1 to 0.2)	11.1 (8.4 to 14.3)	-0.69 (-0.73 to -0.64)
Uzbekistan	11.5 (8.5 to 14.5)	85.5 (64.1 to 107.2)	11.5 (8.5 to 14.5)	85.5 (64.1 to 107.2)	-2.69 (-3.10 to -2.29)
Vanuatu	0 (0 to 0)	36.6 (27.9 to 48.2)	0 (0 to 0)	36.6 (27.9 to 48.2)	-0.77 (-0.86 to -0.68)
Venezuela (Bolivarian Republic of)	3 (2.3 to 3.9)	27.5 (21.1 to 35.4)	3 (2.3 to 3.9)	27.5 (21.1 to 35.4)	0.03 (-0.08 to 0.15)
Viet Nam	64.5 (47.9 to 83.1)	157.2 (117.3 to 201.8)	64.5 (47.9 to 83.1)	157.2 (117.3 to 201.8)	-4.10 (-4.15 to -4.05)
Yemen	11.3 (8.6 to 14.3)	117.7 (92.4 to 146)	11.3 (8.6 to 14.3)	117.7 (92.4 to 146)	-0.43 (-0.91 to 0.06)
Zambia	4.1 (3.1 to 5.4)	84.8 (65.6 to 109.6)	4.1 (3.1 to 5.4)	84.8 (65.6 to 109.6)	-1.61 (-1.94 to -1.27)
Zimbabwe	12.2 (10.9 to 13.5)	178.9 (158.6 to 199.4)	12.2 (10.9 to 13.5)	178.9 (158.6 to 199.4)	-0.22 (-0.51 to 0.08)

Abbreviation: ASR, age standardized rate; CI, confidence interval; EAPC, estimated annual percentage change; UI, uncertainty interval.

Table S12: The change of iodine deficiency cases between 1990 and 2019 at national level for male.

Country	1990		2019		1990–2019
	Incident cases No.×10^4[95% UI]	ASR per 10,0000 No.[95% UI]	Incident cases No.×10^4[95% UI]	ASR per 10,0000 No.[95% UI]	EAPC No.[95% CI]
Afghanistan	4 (3 to 5.1)	58.3 (45.5 to 71.7)	4 (3 to 5.1)	58.3 (45.5 to 71.7)	-1.17 (-2.02 to -0.31)
Albania	0.2 (0.2 to 0.3)	12.8 (9.6 to 16.3)	0.2 (0.2 to 0.3)	12.8 (9.6 to 16.3)	-1.96 (-2.13 to -1.80)
Algeria	4.8 (3.6 to 6.1)	31.8 (25 to 39.5)	4.8 (3.6 to 6.1)	31.8 (25 to 39.5)	-1.43 (-1.60 to -1.26)
American Samoa	0 (0 to 0)	3.3 (2.4 to 4.3)	0 (0 to 0)	3.3 (2.4 to 4.3)	-1.47 (-1.61 to -1.33)
Andorra	0 (0 to 0)	26.9 (20.9 to 34.5)	0 (0 to 0)	26.9 (20.9 to 34.5)	-0.02 (-0.06 to 0.02)
Angola	20.1 (15.5 to 25.1)	293.8 (232.5 to 366.6)	20.1 (15.5 to 25.1)	293.8 (232.5 to 366.6)	-2.35 (-2.80 to -1.89)
Antigua and Barbuda	0 (0 to 0)	16.1 (12.4 to 20.7)	0 (0 to 0)	16.1 (12.4 to 20.7)	-2.39 (-2.53 to -2.24)
Argentina	1.5 (1.1 to 2)	9.1 (6.8 to 11.7)	1.5 (1.1 to 2)	9.1 (6.8 to 11.7)	-0.63 (-0.65 to -0.61)
Armenia	0.1 (0 to 0.1)	3.7 (2.7 to 5.1)	0.1 (0 to 0.1)	3.7 (2.7 to 5.1)	-0.59 (-0.69 to -0.49)
Australia	0.9 (0.7 to 1.2)	11.2 (8.7 to 14.4)	0.9 (0.7 to 1.2)	11.2 (8.7 to 14.4)	-0.24 (-0.24 to -0.23)
Austria	1.1 (0.8 to 1.4)	29.1 (22.5 to 37)	1.1 (0.8 to 1.4)	29.1 (22.5 to 37)	-0.18 (-0.22 to -0.14)
Azerbaijan	0.2 (0.1 to 0.3)	4.8 (3.5 to 6.6)	0.2 (0.1 to 0.3)	4.8 (3.5 to 6.6)	-1.36 (-1.91 to -0.80)
Bahamas	0 (0 to 0)	11.1 (8.5 to 14.2)	0 (0 to 0)	11.1 (8.5 to 14.2)	-1.63 (-1.76 to -1.51)
Bahrain	0.1 (0.1 to 0.1)	22.5 (17.7 to 28.3)	0.1 (0.1 to 0.1)	22.5 (17.7 to 28.3)	-1.16 (-1.25 to -1.07)
Bangladesh	169.1 (147.6 to 193.4)	232.9 (202 to 268.5)	169.1 (147.6 to 193.4)	232.9 (202 to 268.5)	-2.33 (-2.93 to -1.72)
Barbados	0 (0 to 0)	22.7 (17.6 to 29.4)	0 (0 to 0)	22.7 (17.6 to 29.4)	-0.65 (-0.73 to -0.57)
Belarus	0.3 (0.2 to 0.3)	5.3 (3.9 to 7)	0.3 (0.2 to 0.3)	5.3 (3.9 to 7)	-0.96 (-1.31 to -0.62)
Belgium	1.3 (1 to 1.7)	29 (22.5 to 37.2)	1.3 (1 to 1.7)	29 (22.5 to 37.2)	-0.27 (-0.29 to -0.24)
Belize	0 (0 to 0)	8.1 (6.2 to 10.4)	0 (0 to 0)	8.1 (6.2 to 10.4)	-1.50 (-1.56 to -1.44)
Benin	1.1 (0.9 to 1.5)	44.5 (34.5 to 56.7)	1.1 (0.9 to 1.5)	44.5 (34.5 to 56.7)	-0.89 (-0.96 to -0.82)
Bermuda	0 (0 to 0)	9.8 (7.5 to 12.7)	0 (0 to 0)	9.8 (7.5 to 12.7)	-2.15 (-2.30 to -1.99)
Bhutan	0.1 (0.1 to 0.1)	25.3 (19.4 to 32.3)	0.1 (0.1 to 0.1)	25.3 (19.4 to 32.3)	-0.67 (-0.95 to -0.38)
Bolivia (Plurinational State of)	0.2 (0.2 to 0.3)	6 (4.8 to 7.6)	0.2 (0.2 to 0.3)	6 (4.8 to 7.6)	-0.88 (-0.94 to -0.82)
Bosnia and Herzegovina	0.5 (0.4 to 0.7)	24.4 (18.4 to 30.9)	0.5 (0.4 to 0.7)	24.4 (18.4 to 30.9)	-2.81 (-3.22 to -2.39)
Botswana	0.1 (0.1 to 0.1)	17.7 (13.9 to 22.1)	0.1 (0.1 to 0.1)	17.7 (13.9 to 22.1)	-0.50 (-0.54 to -0.45)
Brazil	4.9 (3.6 to 6.4)	6.2 (4.7 to 8)	4.9 (3.6 to 6.4)	6.2 (4.7 to 8)	-0.54 (-0.58 to -0.51)
Brunei Darussalam	0 (0 to 0)	13.1 (10.1 to 16.6)	0 (0 to 0)	13.1 (10.1 to 16.6)	-0.26 (-0.29 to -0.23)
Bulgaria	0.2 (0.2 to 0.3)	6.3 (4.5 to 8.1)	0.2 (0.2 to 0.3)	6.3 (4.5 to 8.1)	-1.45 (-1.55 to -1.36)
Burkina Faso	3.2 (2.4 to 4.1)	62 (48.8 to 78.2)	3.2 (2.4 to 4.1)	62 (48.8 to 78.2)	-1.89 (-2.03 to -1.74)
Burundi	5 (4.4 to 5.6)	152.2 (131.6 to 172.5)	5 (4.4 to 5.6)	152.2 (131.6 to 172.5)	-2.13 (-2.29 to -1.98)
Cabo Verde	0.1 (0.1 to 0.2)	74.3 (57.6 to 95.7)	0.1 (0.1 to 0.2)	74.3 (57.6 to 95.7)	-1.43 (-1.59 to -1.27)
Cambodia	5 (3.8 to 6.5)	89.8 (68.5 to 118.9)	5 (3.8 to 6.5)	89.8 (68.5 to 118.9)	-5.72 (-6.24 to -5.19)
Cameroon	1.6 (1.2 to 2.1)	30 (23.4 to 37.8)	1.6 (1.2 to 2.1)	30 (23.4 to 37.8)	-0.35 (-0.47 to -0.23)

Continued on next page

Table S12 – continued from previous page

Country	1990		2019		1990–2019
	Incident cases No.×10^4[95% UI]	ASR per 10,0000 No.[95% UI]	Incident cases No.×10^4[95% UI]	ASR per 10,0000 No.[95% UI]	EAPC No.[95% CI]
Canada	1.5 (1.2 to 2)	11.7 (9.1 to 14.9)	1.5 (1.2 to 2)	11.7 (9.1 to 14.9)	-0.23 (-0.25 to -0.21)
Central African Republic	5.3 (4.4 to 6.3)	303.4 (247.3 to 359.2)	5.3 (4.4 to 6.3)	303.4 (247.3 to 359.2)	-2.43 (-3.06 to -1.80)
Chad	3 (2.3 to 3.8)	84 (66.3 to 105)	3 (2.3 to 3.8)	84 (66.3 to 105)	-2.45 (-2.59 to -2.30)
Chile	0.8 (0.6 to 1)	12 (9.2 to 14.7)	0.8 (0.6 to 1)	12 (9.2 to 14.7)	-1.78 (-1.93 to -1.63)
China	311.4 (235.7 to 409.4)	46.7 (36 to 60.3)	311.4 (235.7 to 409.4)	46.7 (36 to 60.3)	-0.68 (-1.26 to -0.09)
Colombia	4.1 (3 to 5.3)	22 (16.7 to 27.9)	4.1 (3 to 5.3)	22 (16.7 to 27.9)	-0.32 (-0.43 to -0.22)
Comoros	0 (0 to 0.1)	18.9 (14.7 to 23.7)	0 (0 to 0.1)	18.9 (14.7 to 23.7)	-0.09 (-0.16 to -0.02)
Congo	4.3 (3.4 to 5.4)	265.5 (208.4 to 331.7)	4.3 (3.4 to 5.4)	265.5 (208.4 to 331.7)	0.19 (-0.01 to 0.39)
Cook Islands	0 (0 to 0)	3.7 (2.7 to 4.9)	0 (0 to 0)	3.7 (2.7 to 4.9)	-1.68 (-1.85 to -1.51)
Costa Rica	0.4 (0.3 to 0.5)	20.8 (15.7 to 26.3)	0.4 (0.3 to 0.5)	20.8 (15.7 to 26.3)	-0.38 (-0.45 to -0.32)
Croatia	0.2 (0.2 to 0.3)	10.4 (7.7 to 13.5)	0.2 (0.2 to 0.3)	10.4 (7.7 to 13.5)	-1.23 (-1.34 to -1.11)
Cuba	1.3 (1 to 1.7)	22.8 (17.4 to 29.3)	1.3 (1 to 1.7)	22.8 (17.4 to 29.3)	-2.11 (-2.64 to -1.58)
Cyprus	0.1 (0.1 to 0.2)	30.7 (23.6 to 39.9)	0.1 (0.1 to 0.2)	30.7 (23.6 to 39.9)	-0.24 (-0.27 to -0.21)
Czechia	0.2 (0.2 to 0.3)	4.8 (3.4 to 6.4)	0.2 (0.2 to 0.3)	4.8 (3.4 to 6.4)	-1.04 (-1.13 to -0.95)
Ivoirian	2.2 (1.7 to 2.8)	32.2 (25.4 to 40.5)	2.2 (1.7 to 2.8)	32.2 (25.4 to 40.5)	-1.30 (-1.49 to -1.11)
Democratic People's Republic of Korea	0.5 (0.4 to 0.6)	4.9 (3.7 to 6.3)	0.5 (0.4 to 0.6)	4.9 (3.7 to 6.3)	-2.06 (-2.32 to -1.79)
Democratic Republic of the Congo	128.5 (110.1 to 147.6)	488.1 (418.7 to 565)	128.5 (110.1 to 147.6)	488.1 (418.7 to 565)	-0.23 (-0.60 to 0.14)
Denmark	0.5 (0.4 to 0.6)	21 (16.4 to 26.5)	0.5 (0.4 to 0.6)	21 (16.4 to 26.5)	-0.28 (-0.35 to -0.22)
Djibouti	1.5 (1.2 to 1.9)	454.3 (357.9 to 570.4)	1.5 (1.2 to 1.9)	454.3 (357.9 to 570.4)	-0.28 (-0.68 to 0.12)
Dominica	0 (0 to 0)	25 (19.1 to 32.1)	0 (0 to 0)	25 (19.1 to 32.1)	-2.48 (-2.66 to -2.30)
Dominican Republic	1.3 (0.9 to 1.7)	30.9 (23.5 to 39.9)	1.3 (0.9 to 1.7)	30.9 (23.5 to 39.9)	-2.38 (-2.49 to -2.27)
Ecuador	0.3 (0.2 to 0.3)	4.9 (3.6 to 6.4)	0.3 (0.2 to 0.3)	4.9 (3.6 to 6.4)	-0.48 (-0.54 to -0.42)
Egypt	12.5 (9.5 to 16)	38 (29.9 to 48.1)	12.5 (9.5 to 16)	38 (29.9 to 48.1)	-3.04 (-3.32 to -2.76)
El Salvador	0.8 (0.6 to 1.1)	26.6 (20.1 to 34.2)	0.8 (0.6 to 1.1)	26.6 (20.1 to 34.2)	-0.74 (-0.83 to -0.66)
Equatorial Guinea	2.4 (1.8 to 2.9)	760 (613.2 to 899.1)	2.4 (1.8 to 2.9)	760 (613.2 to 899.1)	-7.10 (-8.03 to -6.16)
Eritrea	0.6 (0.4 to 0.7)	33.2 (26.1 to 42.1)	0.6 (0.4 to 0.7)	33.2 (26.1 to 42.1)	-1.05 (-1.24 to -0.85)
Estonia	0 (0 to 0)	3.7 (2.6 to 5.2)	0 (0 to 0)	3.7 (2.6 to 5.2)	-0.78 (-0.88 to -0.68)
Eswatini	0.2 (0.2 to 0.3)	46.2 (36.1 to 58.7)	0.2 (0.2 to 0.3)	46.2 (36.1 to 58.7)	-0.76 (-0.80 to -0.72)
Ethiopia	151.1 (121.2 to 185.5)	481.4 (382.6 to 592.9)	151.1 (121.2 to 185.5)	481.4 (382.6 to 592.9)	-0.48 (-1.11 to 0.14)
Fiji	0 (0 to 0)	4.7 (3.5 to 6.2)	0 (0 to 0)	4.7 (3.5 to 6.2)	-2.09 (-2.33 to -1.85)
Finland	0.7 (0.5 to 0.9)	29.3 (22.6 to 37.8)	0.7 (0.5 to 0.9)	29.3 (22.6 to 37.8)	-0.24 (-0.27 to -0.21)
France	7.9 (6.1 to 10.3)	28.9 (22.2 to 37.4)	7.9 (6.1 to 10.3)	28.9 (22.2 to 37.4)	-0.13 (-0.15 to -0.10)
Gabon	1.1 (0.9 to 1.4)	187.1 (147 to 232.8)	1.1 (0.9 to 1.4)	187.1 (147 to 232.8)	-3.79 (-4.27 to -3.30)
Gambia	0.8 (0.6 to 1.1)	144.3 (111.1 to 183.5)	0.8 (0.6 to 1.1)	144.3 (111.1 to 183.5)	-1.55 (-1.86 to -1.23)
Georgia	0.1 (0.1 to 0.1)	3.6 (2.6 to 4.7)	0.1 (0.1 to 0.1)	3.6 (2.6 to 4.7)	-0.41 (-0.52 to -0.30)

Continued on next page

Table S12 – continued from previous page

Country	1990		2019		1990–2019
	Incident cases No.×10^4[95% UI]	ASR per 10,0000 No.[95% UI]	Incident cases No.×10^4[95% UI]	ASR per 10,0000 No.[95% UI]	EAPC No.[95% CI]
Germany	10.6 (8.1 to 13.6)	28.8 (22.4 to 36.9)	10.6 (8.1 to 13.6)	28.8 (22.4 to 36.9)	-0.20 (-0.24 to -0.16)
Ghana	12.9 (10.1 to 16.4)	146.2 (114.8 to 185.6)	12.9 (10.1 to 16.4)	146.2 (114.8 to 185.6)	-2.87 (-3.08 to -2.67)
Greece	1.5 (1.1 to 1.9)	30.7 (23.5 to 39.5)	1.5 (1.1 to 1.9)	30.7 (23.5 to 39.5)	-0.39 (-0.44 to -0.34)
Greenland	0 (0 to 0)	11 (8.5 to 13.9)	0 (0 to 0)	11 (8.5 to 13.9)	-0.04 (-0.08 to 0.00)
Grenada	0 (0 to 0)	25.8 (19.8 to 33.1)	0 (0 to 0)	25.8 (19.8 to 33.1)	-3.15 (-3.28 to -3.01)
Guam	0 (0 to 0)	3.3 (2.4 to 4.3)	0 (0 to 0)	3.3 (2.4 to 4.3)	-1.35 (-1.52 to -1.17)
Guatemala	1 (0.8 to 1.4)	21.7 (16.4 to 27.5)	1 (0.8 to 1.4)	21.7 (16.4 to 27.5)	-0.07 (-0.14 to 0.00)
Guinea	9.4 (7.2 to 11.8)	225.8 (178.1 to 277.6)	9.4 (7.2 to 11.8)	225.8 (178.1 to 277.6)	-3.64 (-3.86 to -3.41)
Guinea-Bissau	0.9 (0.8 to 1.1)	156.8 (125.5 to 193.7)	0.9 (0.8 to 1.1)	156.8 (125.5 to 193.7)	-1.56 (-1.72 to -1.39)
Guyana	0.2 (0.1 to 0.2)	37.1 (28.6 to 47.1)	0.2 (0.1 to 0.2)	37.1 (28.6 to 47.1)	-2.09 (-2.25 to -1.93)
Haiti	2 (1.5 to 2.5)	53.3 (40.7 to 66.8)	2 (1.5 to 2.5)	53.3 (40.7 to 66.8)	-0.88 (-1.15 to -0.60)
Honduras	0.9 (0.6 to 1.2)	28.5 (21.6 to 36.3)	0.9 (0.6 to 1.2)	28.5 (21.6 to 36.3)	-0.38 (-0.54 to -0.21)
Hungary	0.8 (0.6 to 1)	16.7 (12.5 to 21.2)	0.8 (0.6 to 1)	16.7 (12.5 to 21.2)	-2.53 (-2.81 to -2.26)
Iceland	0 (0 to 0)	14.5 (11.2 to 18.7)	0 (0 to 0)	14.5 (11.2 to 18.7)	-0.23 (-0.32 to -0.13)
India	1815.9 (1431.6 to 2240.6)	330.9 (263.4 to 406.5)	1815.9 (1431.6 to 2240.6)	330.9 (263.4 to 406.5)	-1.91 (-2.39 to -1.43)
Indonesia	25.4 (19.4 to 33)	24.9 (19.4 to 31.9)	25.4 (19.4 to 33)	24.9 (19.4 to 31.9)	-2.29 (-2.42 to -2.17)
Iran (Islamic Republic of)	6.2 (4.6 to 8)	18.2 (14.4 to 22.6)	6.2 (4.6 to 8)	18.2 (14.4 to 22.6)	-1.84 (-2.05 to -1.64)
Iraq	6 (4.5 to 7.7)	54 (42.3 to 66.8)	6 (4.5 to 7.7)	54 (42.3 to 66.8)	-1.95 (-2.46 to -1.43)
Ireland	0.6 (0.4 to 0.7)	30.6 (23.8 to 39.1)	0.6 (0.4 to 0.7)	30.6 (23.8 to 39.1)	-0.44 (-0.45 to -0.42)
Israel	0.8 (0.6 to 1)	30.6 (23.7 to 39)	0.8 (0.6 to 1)	30.6 (23.7 to 39)	-0.25 (-0.27 to -0.22)
Italy	35.1 (26.7 to 45)	135.5 (104.4 to 172.9)	35.1 (26.7 to 45)	135.5 (104.4 to 172.9)	-1.56 (-1.82 to -1.29)
Jamaica	0.3 (0.2 to 0.4)	24.7 (18.8 to 31.8)	0.3 (0.2 to 0.4)	24.7 (18.8 to 31.8)	-1.80 (-1.95 to -1.65)
Japan	9.1 (7.2 to 11.5)	15.2 (12 to 19.1)	9.1 (7.2 to 11.5)	15.2 (12 to 19.1)	-0.42 (-0.44 to -0.40)
Jordan	0.8 (0.6 to 1)	33.9 (27 to 42)	0.8 (0.6 to 1)	33.9 (27 to 42)	-1.40 (-1.56 to -1.24)
Kazakhstan	0.5 (0.3 to 0.6)	5.8 (4.2 to 7.6)	0.5 (0.3 to 0.6)	5.8 (4.2 to 7.6)	-2.13 (-2.44 to -1.81)
Kenya	6.7 (5.1 to 8.6)	50.7 (40.1 to 63.9)	6.7 (5.1 to 8.6)	50.7 (40.1 to 63.9)	0.36 (0.11 to 0.61)
Kiribati	0 (0 to 0)	6.5 (4.9 to 8.4)	0 (0 to 0)	6.5 (4.9 to 8.4)	-2.31 (-2.72 to -1.89)
Kuwait	0.2 (0.2 to 0.3)	21.5 (16.5 to 27)	0.2 (0.2 to 0.3)	21.5 (16.5 to 27)	-1.17 (-1.27 to -1.08)
Kyrgyzstan	0.1 (0.1 to 0.1)	4 (2.9 to 5.4)	0.1 (0.1 to 0.1)	4 (2.9 to 5.4)	-0.56 (-0.62 to -0.50)
Lao People's Democratic Republic	0.5 (0.4 to 0.7)	25.2 (19.5 to 32.1)	0.5 (0.4 to 0.7)	25.2 (19.5 to 32.1)	-2.18 (-2.34 to -2.02)
Latvia	0 (0 to 0.1)	3.7 (2.6 to 4.9)	0 (0 to 0.1)	3.7 (2.6 to 4.9)	-0.63 (-0.87 to -0.40)
Lebanon	1.8 (1.5 to 2)	87.8 (75.3 to 101.7)	1.8 (1.5 to 2)	87.8 (75.3 to 101.7)	-2.57 (-2.99 to -2.15)
Lesotho	5.3 (4.8 to 5.8)	448.2 (401.5 to 499)	5.3 (4.8 to 5.8)	448.2 (401.5 to 499)	-3.96 (-4.76 to -3.15)
Liberia	0.3 (0.2 to 0.4)	30 (23.5 to 38.1)	0.3 (0.2 to 0.4)	30 (23.5 to 38.1)	-1.08 (-1.46 to -0.70)
Libya	0.9 (0.7 to 1.2)	36.6 (28.5 to 45.4)	0.9 (0.7 to 1.2)	36.6 (28.5 to 45.4)	-0.26 (-0.61 to 0.09)

Continued on next page

Table S12 – continued from previous page

Country	1990		2019		1990–2019
	Incident cases No.$\times 10^4$[95% UI]	ASR per 10,0000 No.[95% UI]	Incident cases No.$\times 10^4$[95% UI]	ASR per 10,0000 No.[95% UI]	EAPC No.[95% CI]
Lithuania	0.1 (0 to 0.1)	3.6 (2.6 to 4.9)	0.1 (0 to 0.1)	3.6 (2.6 to 4.9)	-0.69 (-0.92 to -0.47)
Luxembourg	0 (0 to 0.1)	27.8 (21.3 to 36.2)	0 (0 to 0.1)	27.8 (21.3 to 36.2)	-0.14 (-0.17 to -0.11)
Madagascar	4.6 (3.4 to 5.8)	70.6 (53.5 to 90.4)	4.6 (3.4 to 5.8)	70.6 (53.5 to 90.4)	0.37 (0.20 to 0.53)
Malawi	6.2 (4.7 to 8)	113.2 (87.1 to 145.6)	6.2 (4.7 to 8)	113.2 (87.1 to 145.6)	-1.49 (-1.85 to -1.14)
Malaysia	15.8 (12.7 to 19.1)	153.5 (122.9 to 186.6)	15.8 (12.7 to 19.1)	153.5 (122.9 to 186.6)	-6.46 (-6.88 to -6.03)
Maldives	0 (0 to 0)	28.4 (22.1 to 36)	0 (0 to 0)	28.4 (22.1 to 36)	-4.24 (-4.34 to -4.14)
Mali	2.2 (1.7 to 2.9)	48 (37.5 to 61.2)	2.2 (1.7 to 2.9)	48 (37.5 to 61.2)	-1.60 (-1.70 to -1.50)
Malta	0.1 (0 to 0.1)	32.9 (25.3 to 42.3)	0.1 (0 to 0.1)	32.9 (25.3 to 42.3)	-0.45 (-0.47 to -0.43)
Marshall Islands	0 (0 to 0)	5.8 (4.4 to 7.6)	0 (0 to 0)	5.8 (4.4 to 7.6)	-2.41 (-2.77 to -2.06)
Mauritania	2 (1.5 to 2.4)	155.9 (121.9 to 195.3)	2 (1.5 to 2.4)	155.9 (121.9 to 195.3)	-1.75 (-2.00 to -1.50)
Mauritius	0.2 (0.1 to 0.2)	26.9 (20.8 to 34.5)	0.2 (0.1 to 0.2)	26.9 (20.8 to 34.5)	-2.56 (-2.65 to -2.46)
Mexico	10.4 (7.7 to 13.3)	20.8 (16 to 25.9)	10.4 (7.7 to 13.3)	20.8 (16 to 25.9)	0.06 (-0.01 to 0.14)
Micronesia (Federated States of)	0 (0 to 0)	5.9 (4.5 to 7.7)	0 (0 to 0)	5.9 (4.5 to 7.7)	-2.40 (-2.71 to -2.10)
Monaco	0 (0 to 0)	25.3 (19.6 to 32.4)	0 (0 to 0)	25.3 (19.6 to 32.4)	0.02 (-0.02 to 0.07)
Mongolia	0.1 (0.1 to 0.1)	8.6 (6.3 to 11.1)	0.1 (0.1 to 0.1)	8.6 (6.3 to 11.1)	-2.63 (-2.96 to -2.30)
Montenegro	0 (0 to 0)	10.5 (7.8 to 13.5)	0 (0 to 0)	10.5 (7.8 to 13.5)	-1.24 (-1.33 to -1.14)
Morocco	16.5 (12.8 to 21)	105.4 (83.9 to 131.9)	16.5 (12.8 to 21)	105.4 (83.9 to 131.9)	-2.58 (-2.78 to -2.39)
Mozambique	7.6 (5.8 to 9.4)	99 (77.2 to 123.6)	7.6 (5.8 to 9.4)	99 (77.2 to 123.6)	-2.47 (-2.85 to -2.08)
Myanmar	9.2 (7 to 12)	40.4 (31.4 to 52.1)	9.2 (7 to 12)	40.4 (31.4 to 52.1)	-3.73 (-3.96 to -3.50)
Namibia	0.3 (0.2 to 0.3)	32.9 (25.7 to 41.3)	0.3 (0.2 to 0.3)	32.9 (25.7 to 41.3)	-1.29 (-1.32 to -1.26)
Nauru	0 (0 to 0)	3.7 (2.7 to 4.9)	0 (0 to 0)	3.7 (2.7 to 4.9)	-1.28 (-1.89 to -0.67)
Nepal	6.9 (5.2 to 8.8)	62 (48 to 79)	6.9 (5.2 to 8.8)	62 (48 to 79)	-0.40 (-0.75 to -0.05)
Netherlands	2.1 (1.6 to 2.7)	28.1 (21.7 to 35.7)	2.1 (1.6 to 2.7)	28.1 (21.7 to 35.7)	-0.20 (-0.22 to -0.18)
New Zealand	0.2 (0.2 to 0.3)	12.8 (10 to 16.2)	0.2 (0.2 to 0.3)	12.8 (10 to 16.2)	-0.13 (-0.16 to -0.10)
Nicaragua	0.7 (0.5 to 0.9)	27 (20.3 to 34.3)	0.7 (0.5 to 0.9)	27 (20.3 to 34.3)	-0.41 (-0.65 to -0.17)
Niger	6.5 (5.2 to 7.7)	123.1 (99.3 to 149.8)	6.5 (5.2 to 7.7)	123.1 (99.3 to 149.8)	-0.53 (-0.66 to -0.39)
Nigeria	18.8 (14.6 to 24)	39.1 (30.8 to 48.8)	18.8 (14.6 to 24)	39.1 (30.8 to 48.8)	-0.56 (-0.65 to -0.47)
Niue	0 (0 to 0)	4.2 (3.1 to 5.5)	0 (0 to 0)	4.2 (3.1 to 5.5)	-1.94 (-2.20 to -1.69)
North Macedonia	0.1 (0.1 to 0.1)	11.3 (8.3 to 14.6)	0.1 (0.1 to 0.1)	11.3 (8.3 to 14.6)	-1.30 (-1.38 to -1.22)
Northern Mariana Islands	0 (0 to 0)	3.3 (2.4 to 4.4)	0 (0 to 0)	3.3 (2.4 to 4.4)	-1.24 (-1.42 to -1.06)
Norway	0.6 (0.5 to 0.8)	29.9 (23.2 to 38.2)	0.6 (0.5 to 0.8)	29.9 (23.2 to 38.2)	-0.22 (-0.24 to -0.19)
Oman	0.3 (0.2 to 0.4)	23.7 (18.4 to 29.9)	0.3 (0.2 to 0.4)	23.7 (18.4 to 29.9)	-1.31 (-1.41 to -1.20)
Pakistan	59 (46 to 74.4)	86.5 (68.2 to 109.1)	59 (46 to 74.4)	86.5 (68.2 to 109.1)	-0.81 (-0.88 to -0.74)
Palau	0 (0 to 0)	3.9 (2.8 to 5.1)	0 (0 to 0)	3.9 (2.8 to 5.1)	-1.65 (-1.86 to -1.44)
Palestine	0.3 (0.2 to 0.4)	26 (20.4 to 33)	0.3 (0.2 to 0.4)	26 (20.4 to 33)	-1.38 (-1.44 to -1.32)

Continued on next page

Country	1990		2019		1990–2019
	Incident cases No.×10^4[95% UI]	ASR per 10,0000 No.[95% UI]	Incident cases No.×10^4[95% UI]	ASR per 10,0000 No.[95% UI]	EAPC No.[95% CI]
Panama	0.3 (0.2 to 0.4)	21 (15.4 to 28.9)	0.3 (0.2 to 0.4)	21 (15.4 to 28.9)	-1.63 (-1.85 to -1.41)
Papua New Guinea	0.1 (0.1 to 0.2)	6.1 (4.6 to 8)	0.1 (0.1 to 0.2)	6.1 (4.6 to 8)	-2.56 (-2.95 to -2.18)
Paraguay	0.2 (0.1 to 0.2)	7.7 (5.8 to 9.8)	0.2 (0.1 to 0.2)	7.7 (5.8 to 9.8)	-0.95 (-1.01 to -0.89)
Peru	0.6 (0.4 to 0.7)	4.9 (3.6 to 6.3)	0.6 (0.4 to 0.7)	4.9 (3.6 to 6.3)	-0.57 (-0.64 to -0.49)
Philippines	22.9 (17.9 to 29.2)	62.8 (49.6 to 79)	22.9 (17.9 to 29.2)	62.8 (49.6 to 79)	0.96 (0.06 to 1.88)
Poland	1.8 (1.3 to 2.2)	9.5 (7.1 to 12.1)	1.8 (1.3 to 2.2)	9.5 (7.1 to 12.1)	-0.93 (-0.98 to -0.88)
Portugal	2.2 (1.7 to 2.8)	45.1 (35.1 to 57.1)	2.2 (1.7 to 2.8)	45.1 (35.1 to 57.1)	0.03 (-0.02 to 0.08)
Puerto Rico	0.2 (0.2 to 0.3)	12.8 (9.9 to 16.6)	0.2 (0.2 to 0.3)	12.8 (9.9 to 16.6)	-2.29 (-2.37 to -2.21)
Qatar	0.1 (0 to 0.1)	20.1 (15.4 to 25.1)	0.1 (0 to 0.1)	20.1 (15.4 to 25.1)	-1.21 (-1.32 to -1.10)
Republic of Korea	4 (3.1 to 5.2)	16.9 (13.1 to 21.1)	4 (3.1 to 5.2)	16.9 (13.1 to 21.1)	-1.13 (-1.20 to -1.07)
Republic of Moldova	0.1 (0 to 0.1)	2.9 (2.1 to 4)	0.1 (0 to 0.1)	2.9 (2.1 to 4)	0.39 (-0.21 to 1.00)
Romania	2.3 (1.9 to 2.8)	20.6 (17.4 to 24.7)	2.3 (1.9 to 2.8)	20.6 (17.4 to 24.7)	0.39 (0.10 to 0.69)
Russian Federation	2.9 (2.1 to 3.8)	4.2 (3.1 to 5.5)	2.9 (2.1 to 3.8)	4.2 (3.1 to 5.5)	-0.26 (-0.63 to 0.12)
Rwanda	6.1 (4.5 to 7.9)	131.3 (100.8 to 162.3)	6.1 (4.5 to 7.9)	131.3 (100.8 to 162.3)	-1.84 (-2.20 to -1.47)
Saint Kitts and Nevis	0 (0 to 0)	18.5 (14.3 to 24.1)	0 (0 to 0)	18.5 (14.3 to 24.1)	-2.88 (-2.95 to -2.81)
Saint Lucia	0 (0 to 0)	9.6 (7.4 to 12.4)	0 (0 to 0)	9.6 (7.4 to 12.4)	-1.33 (-1.38 to -1.28)
Saint Vincent and the Grenadines	0 (0 to 0)	26.6 (20.4 to 33.8)	0 (0 to 0)	26.6 (20.4 to 33.8)	-2.88 (-3.02 to -2.73)
Samoa	0 (0 to 0)	3.1 (2.3 to 4.1)	0 (0 to 0)	3.1 (2.3 to 4.1)	-1.39 (-1.47 to -1.31)
San Marino	0 (0 to 0)	27.3 (21.2 to 35.4)	0 (0 to 0)	27.3 (21.2 to 35.4)	-0.03 (-0.05 to -0.01)
Sao Tome and Principe	0 (0 to 0)	39 (30.4 to 48.8)	0 (0 to 0)	39 (30.4 to 48.8)	-0.93 (-1.13 to -0.73)
Saudi Arabia	1 (0.8 to 1.3)	10.4 (8 to 13.1)	1 (0.8 to 1.3)	10.4 (8 to 13.1)	-1.37 (-1.50 to -1.24)
Senegal	4.5 (3.7 to 5.3)	102.6 (84.7 to 123.1)	4.5 (3.7 to 5.3)	102.6 (84.7 to 123.1)	-2.01 (-2.25 to -1.76)
Serbia	0.5 (0.4 to 0.6)	11.3 (8.3 to 14.6)	0.5 (0.4 to 0.6)	11.3 (8.3 to 14.6)	-1.41 (-1.52 to -1.29)
Seychelles	0 (0 to 0)	22.2 (17.2 to 28.4)	0 (0 to 0)	22.2 (17.2 to 28.4)	-2.16 (-2.25 to -2.07)
Sierra Leone	1 (0.8 to 1.4)	54.1 (42.1 to 68.9)	1 (0.8 to 1.4)	54.1 (42.1 to 68.9)	-0.90 (-1.28 to -0.51)
Singapore	0.2 (0.2 to 0.3)	14.4 (11.1 to 18.3)	0.2 (0.2 to 0.3)	14.4 (11.1 to 18.3)	-0.59 (-0.63 to -0.56)
Slovakia	0.3 (0.2 to 0.3)	10.6 (7.8 to 13.6)	0.3 (0.2 to 0.3)	10.6 (7.8 to 13.6)	-1.36 (-1.47 to -1.26)
Slovenia	0.1 (0.1 to 0.1)	10.2 (7.5 to 13.2)	0.1 (0.1 to 0.1)	10.2 (7.5 to 13.2)	-1.22 (-1.34 to -1.09)
Solomon Islands	0 (0 to 0)	4.2 (3.1 to 5.6)	0 (0 to 0)	4.2 (3.1 to 5.6)	-1.16 (-1.35 to -0.97)
Somalia	24.8 (19.9 to 30.1)	572.6 (455 to 700.1)	24.8 (19.9 to 30.1)	572.6 (455 to 700.1)	0.40 (0.28 to 0.52)
South Africa	6.2 (4.7 to 7.9)	31.4 (24.6 to 38.9)	6.2 (4.7 to 7.9)	31.4 (24.6 to 38.9)	-0.80 (-0.86 to -0.73)
South Sudan	2.1 (1.6 to 2.7)	58.8 (45.7 to 76.1)	2.1 (1.6 to 2.7)	58.8 (45.7 to 76.1)	0.36 (0.22 to 0.49)
Spain	5.6 (4.3 to 7.3)	29.4 (22.6 to 38.1)	5.6 (4.3 to 7.3)	29.4 (22.6 to 38.1)	-0.20 (-0.22 to -0.18)
Sri Lanka	5.8 (4.6 to 7.3)	59.6 (47.3 to 74.6)	5.8 (4.6 to 7.3)	59.6 (47.3 to 74.6)	-3.25 (-3.44 to -3.06)
Sudan	10.1 (7.8 to 12.5)	78.3 (62 to 95.1)	10.1 (7.8 to 12.5)	78.3 (62 to 95.1)	-2.67 (-3.14 to -2.21)

Continued on next page

Table S12 – continued from previous page

Country	1990		2019		1990–2019
	Incident cases No.×10^4[95% UI]	ASR per 10,0000 No.[95% UI]	Incident cases No.×10^4[95% UI]	ASR per 10,0000 No.[95% UI]	EAPC No.[95% CI]
Suriname	0 (0 to 0.1)	21.1 (16.3 to 26.8)	0 (0 to 0.1)	21.1 (16.3 to 26.8)	-2.80 (-3.16 to -2.44)
Sweden	1.2 (1 to 1.6)	31.8 (24.6 to 41.4)	1.2 (1 to 1.6)	31.8 (24.6 to 41.4)	-0.26 (-0.29 to -0.22)
Switzerland	0.7 (0.5 to 0.9)	21.5 (16.7 to 27)	0.7 (0.5 to 0.9)	21.5 (16.7 to 27)	-0.19 (-0.25 to -0.13)
Syrian Arab Republic	5 (3.7 to 6.4)	59.6 (46.7 to 73)	5 (3.7 to 6.4)	59.6 (46.7 to 73)	-1.56 (-1.83 to -1.29)
Taiwan (Province of China)	0.8 (0.6 to 1.1)	7.4 (5.7 to 9.6)	0.8 (0.6 to 1.1)	7.4 (5.7 to 9.6)	-2.18 (-2.33 to -2.03)
Tajikistan	0.2 (0.1 to 0.3)	6.1 (4.5 to 8)	0.2 (0.1 to 0.3)	6.1 (4.5 to 8)	-0.99 (-1.76 to -0.22)
Thailand	11.3 (8.4 to 14.8)	34.6 (26.4 to 44.5)	11.3 (8.4 to 14.8)	34.6 (26.4 to 44.5)	-2.16 (-2.45 to -1.88)
Timor-Leste	0.1 (0.1 to 0.2)	30.7 (23.6 to 39.3)	0.1 (0.1 to 0.2)	30.7 (23.6 to 39.3)	-3.11 (-3.37 to -2.85)
Togo	2.5 (2.2 to 2.9)	114.1 (98.7 to 131.7)	2.5 (2.2 to 2.9)	114.1 (98.7 to 131.7)	-2.21 (-2.55 to -1.88)
Tokelau	0 (0 to 0)	5.3 (4 to 7)	0 (0 to 0)	5.3 (4 to 7)	-2.67 (-2.97 to -2.38)
Tonga	0 (0 to 0)	5.2 (3.9 to 6.9)	0 (0 to 0)	5.2 (3.9 to 6.9)	-2.30 (-2.55 to -2.04)
Trinidad and Tobago	0.2 (0.2 to 0.3)	33.3 (25.5 to 42.8)	0.2 (0.2 to 0.3)	33.3 (25.5 to 42.8)	-3.60 (-4.00 to -3.20)
Tunisia	1.3 (1 to 1.6)	27 (21.5 to 33.5)	1.3 (1 to 1.6)	27 (21.5 to 33.5)	-1.79 (-1.89 to -1.70)
Turkey	28.5 (22.3 to 35.5)	80.2 (63.7 to 99.2)	28.5 (22.3 to 35.5)	80.2 (63.7 to 99.2)	-2.98 (-3.29 to -2.67)
Turkmenistan	0.1 (0 to 0.1)	3.7 (2.6 to 4.9)	0.1 (0 to 0.1)	3.7 (2.6 to 4.9)	-0.53 (-0.65 to -0.41)
Tuvalu	0 (0 to 0)	6.8 (5.2 to 8.9)	0 (0 to 0)	6.8 (5.2 to 8.9)	-2.78 (-2.98 to -2.57)
Uganda	5.4 (4.1 to 7.1)	56.9 (44.5 to 72.5)	5.4 (4.1 to 7.1)	56.9 (44.5 to 72.5)	-1.52 (-1.61 to -1.42)
Ukraine	3.1 (2.3 to 4)	13.7 (10.2 to 17.9)	3.1 (2.3 to 4)	13.7 (10.2 to 17.9)	-0.16 (-0.73 to 0.41)
United Arab Emirates	0.7 (0.6 to 0.9)	58.9 (46.8 to 72.9)	0.7 (0.6 to 0.9)	58.9 (46.8 to 72.9)	-1.32 (-1.50 to -1.15)
United Kingdom	8.1 (6.3 to 10.4)	30.7 (23.7 to 39.2)	8.1 (6.3 to 10.4)	30.7 (23.7 to 39.2)	-0.20 (-0.21 to -0.20)
United Republic of Tanzania	3.6 (2.7 to 4.8)	27 (20.9 to 34.6)	3.6 (2.7 to 4.8)	27 (20.9 to 34.6)	-1.27 (-1.44 to -1.09)
United States of America	14.2 (11 to 17.9)	11.7 (9.1 to 14.7)	14.2 (11 to 17.9)	11.7 (9.1 to 14.7)	-0.01 (-0.03 to 0.01)
United States Virgin Islands	0 (0 to 0)	13.2 (10.2 to 16.9)	0 (0 to 0)	13.2 (10.2 to 16.9)	-2.69 (-2.94 to -2.44)
Uruguay	0.1 (0.1 to 0.2)	9.6 (7.2 to 12.4)	0.1 (0.1 to 0.2)	9.6 (7.2 to 12.4)	-0.77 (-0.82 to -0.71)
Uzbekistan	1 (0.7 to 1.4)	8.4 (6 to 11)	1 (0.7 to 1.4)	8.4 (6 to 11)	-2.29 (-2.68 to -1.90)
Vanuatu	0 (0 to 0)	26.2 (19.7 to 35.3)	0 (0 to 0)	26.2 (19.7 to 35.3)	-1.40 (-1.56 to -1.25)
Venezuela (Bolivarian Republic of)	2 (1.5 to 2.6)	18.7 (14.2 to 23.8)	2 (1.5 to 2.6)	18.7 (14.2 to 23.8)	0.07 (-0.04 to 0.19)
Viet Nam	28.4 (21.3 to 37.5)	73.8 (55.8 to 96.2)	28.4 (21.3 to 37.5)	73.8 (55.8 to 96.2)	-4.07 (-4.16 to -3.98)
Yemen	8.1 (6 to 10.4)	81.6 (63.8 to 100.7)	8.1 (6 to 10.4)	81.6 (63.8 to 100.7)	-0.77 (-1.25 to -0.29)
Zambia	2.7 (2 to 3.5)	59.8 (46.4 to 76.6)	2.7 (2 to 3.5)	59.8 (46.4 to 76.6)	-1.46 (-1.77 to -1.16)
Zimbabwe	9.6 (8.8 to 10.7)	152.6 (136.4 to 170.6)	9.6 (8.8 to 10.7)	152.6 (136.4 to 170.6)	-0.33 (-0.69 to 0.04)

Abbreviation: ASR, age standardized rate; CI, confidence interval; EAPC, estimated annual percentage change; UI, uncertainty interval.

Table S13: The change of vitamin A deficiency cases between 1990 and 2019 at national level for both gender.

Country	1990		2019		1990–2019
	Incident cases No.×10^4[95% UI]	ASR per 10,0000 No.[95% UI]	Incident cases No.×10^4[95% UI]	ASR per 10,0000 No.[95% UI]	EAPC No.[95% CI]
Afghanistan	4775.3 (4362.4 to 5217.5)	41738.5 (37517.6 to 46253.4)	4775.3 (4362.4 to 5217.5)	41738.5 (37517.6 to 46253.4)	-1.64 (-2.09 to -1.18)
Albania	876.8 (777.6 to 986.2)	27225.1 (23823.9 to 31079.8)	876.8 (777.6 to 986.2)	27225.1 (23823.9 to 31079.8)	-3.49 (-3.74 to -3.23)
Algeria	2732 (2269.7 to 3238.7)	10831.3 (8778.7 to 13077.8)	2732 (2269.7 to 3238.7)	10831.3 (8778.7 to 13077.8)	-4.59 (-4.77 to -4.42)
American Samoa	5.3 (4.4 to 6.3)	11586.4 (9465.9 to 14131.9)	5.3 (4.4 to 6.3)	11586.4 (9465.9 to 14131.9)	-1.87 (-2.12 to -1.62)
Andorra	0.3 (0.3 to 0.4)	641.2 (519.8 to 790.4)	0.3 (0.3 to 0.4)	641.2 (519.8 to 790.4)	-1.62 (-1.82 to -1.42)
Angola	4813.7 (4400.5 to 5213.2)	46810 (42173.8 to 51407.8)	4813.7 (4400.5 to 5213.2)	46810 (42173.8 to 51407.8)	-4.05 (-4.42 to -3.68)
Antigua and Barbuda	2.5 (2.1 to 3)	4345.8 (3579.5 to 5253.2)	2.5 (2.1 to 3)	4345.8 (3579.5 to 5253.2)	-2.83 (-2.95 to -2.71)
Argentina	3777 (3300.6 to 4316.2)	12043.1 (10373.2 to 13995.1)	3777 (3300.6 to 4316.2)	12043.1 (10373.2 to 13995.1)	-1.20 (-1.42 to -0.97)
Armenia	35.7 (30.5 to 41.7)	1027.4 (851.4 to 1217.6)	35.7 (30.5 to 41.7)	1027.4 (851.4 to 1217.6)	-2.77 (-3.19 to -2.35)
Australia	27.8 (23.9 to 32)	134.7 (115 to 157.4)	27.8 (23.9 to 32)	134.7 (115 to 157.4)	-1.19 (-1.55 to -0.82)
Austria	85.5 (72.1 to 102.4)	1237.1 (1010.6 to 1509.2)	85.5 (72.1 to 102.4)	1237.1 (1010.6 to 1509.2)	-2.62 (-2.89 to -2.34)
Azerbaijan	317 (263.1 to 383.1)	4384.9 (3553.6 to 5437)	317 (263.1 to 383.1)	4384.9 (3553.6 to 5437)	-2.22 (-2.89 to -1.55)
Bahamas	6.7 (5.6 to 8)	2697.3 (2218.6 to 3284.8)	6.7 (5.6 to 8)	2697.3 (2218.6 to 3284.8)	-2.73 (-2.94 to -2.51)
Bahrain	24.5 (20.3 to 29.7)	4847 (3969.5 to 5928)	24.5 (20.3 to 29.7)	4847 (3969.5 to 5928)	-3.83 (-3.99 to -3.67)
Bangladesh	20271.8 (17751.5 to 22730.6)	18570.5 (16035.6 to 21140.2)	20271.8 (17751.5 to 22730.6)	18570.5 (16035.6 to 21140.2)	-4.30 (-4.63 to -3.97)
Barbados	8.3 (7 to 9.7)	3516.1 (2937 to 4153.2)	8.3 (7 to 9.7)	3516.1 (2937 to 4153.2)	-1.87 (-1.95 to -1.78)
Belarus	346.8 (296.5 to 401.6)	3435.1 (2895.7 to 4034.4)	346.8 (296.5 to 401.6)	3435.1 (2895.7 to 4034.4)	-3.56 (-3.78 to -3.35)
Belgium	107.4 (90.5 to 128.2)	1212.2 (983.7 to 1497.4)	107.4 (90.5 to 128.2)	1212.2 (983.7 to 1497.4)	-2.52 (-2.80 to -2.24)
Belize	23.1 (19.5 to 27.1)	12520.7 (10368.1 to 14922.1)	23.1 (19.5 to 27.1)	12520.7 (10368.1 to 14922.1)	-3.27 (-3.43 to -3.10)
Benin	2493.8 (2314.5 to 2668.3)	52554.2 (48209.5 to 56473.7)	2493.8 (2314.5 to 2668.3)	52554.2 (48209.5 to 56473.7)	-2.10 (-2.22 to -1.98)
Bermuda	1.4 (1.2 to 1.6)	2489.9 (2084.8 to 2968.6)	1.4 (1.2 to 1.6)	2489.9 (2084.8 to 2968.6)	-2.74 (-2.90 to -2.58)
Bhutan	153.6 (132.7 to 174.6)	24935.2 (21140.6 to 28794.5)	153.6 (132.7 to 174.6)	24935.2 (21140.6 to 28794.5)	-5.02 (-5.07 to -4.97)
Bolivia (Plurinational State of)	881.6 (745.1 to 1022.1)	14311.6 (11955.5 to 16904.7)	881.6 (745.1 to 1022.1)	14311.6 (11955.5 to 16904.7)	-1.60 (-1.83 to -1.37)
Bosnia and Herzegovina	987.1 (868.1 to 1105)	22566 (19612.4 to 25555.6)	987.1 (868.1 to 1105)	22566 (19612.4 to 25555.6)	-3.79 (-4.07 to -3.52)
Botswana	337 (292 to 382)	26955.5 (23021.6 to 30719)	337 (292 to 382)	26955.5 (23021.6 to 30719)	-3.46 (-3.59 to -3.34)
Brazil	36407 (32783.5 to 40573.2)	24855.3 (22230.9 to 27862.5)	36407 (32783.5 to 40573.2)	24855.3 (22230.9 to 27862.5)	-3.15 (-3.25 to -3.06)

Continued on next page

Table S13 – continued from previous page

Country	1990		2019		1990–2019
	Incident cases No.$\times 10^4$[95% UI]	ASR per 10,0000 No.[95% UI]	Incident cases No.$\times 10^4$[95% UI]	ASR per 10,0000 No.[95% UI]	EAPC No.[95% CI]
Brunei Darussalam	6.4 (5.2 to 7.9)	2599.1 (2094.3 to 3299.5)	6.4 (5.2 to 7.9)	2599.1 (2094.3 to 3299.5)	-2.95 (-3.01 to -2.89)
Bulgaria	1164.4 (1016.6 to 1320.1)	14271 (12305.3 to 16448.3)	1164.4 (1016.6 to 1320.1)	14271 (12305.3 to 16448.3)	-1.77 (-2.02 to -1.52)
Burkina Faso	5542.1 (5200.4 to 5852.9)	59092.2 (55188.9 to 62800.7)	5542.1 (5200.4 to 5852.9)	59092.2 (55188.9 to 62800.7)	-2.84 (-2.94 to -2.73)
Burundi	2021.6 (1798.1 to 2251.8)	35621.4 (31235.6 to 40430.6)	2021.6 (1798.1 to 2251.8)	35621.4 (31235.6 to 40430.6)	-1.99 (-2.21 to -1.77)
Cabo Verde	100.1 (87 to 113.4)	29867.7 (25413.1 to 34527.9)	100.1 (87 to 113.4)	29867.7 (25413.1 to 34527.9)	-6.06 (-6.33 to -5.80)
Cambodia	3661.4 (3285.3 to 4068.2)	36548.1 (32378.6 to 41153.5)	3661.4 (3285.3 to 4068.2)	36548.1 (32378.6 to 41153.5)	-5.38 (-5.51 to -5.25)
Cameroon	7124 (6885.4 to 7386.5)	69494.5 (66902.4 to 72335.4)	7124 (6885.4 to 7386.5)	69494.5 (66902.4 to 72335.4)	-3.10 (-3.27 to -2.93)
Canada	441.2 (367.6 to 526)	1921.7 (1552.3 to 2321.5)	441.2 (367.6 to 526)	1921.7 (1552.3 to 2321.5)	-2.63 (-2.87 to -2.39)
Central African Republic	1244.8 (1139.7 to 1346.3)	45887.5 (41619.7 to 50082.8)	1244.8 (1139.7 to 1346.3)	45887.5 (41619.7 to 50082.8)	-1.08 (-1.18 to -0.98)
Chad	3570.3 (3374.9 to 3759.3)	60512.8 (56794.8 to 64216.9)	3570.3 (3374.9 to 3759.3)	60512.8 (56794.8 to 64216.9)	-1.90 (-2.00 to -1.79)
Chile	997.3 (825.2 to 1199.1)	8303.5 (6752.5 to 10076.1)	997.3 (825.2 to 1199.1)	8303.5 (6752.5 to 10076.1)	-2.25 (-2.46 to -2.04)
China	122360.8 (103119.2 to 145575.8)	11238 (9347 to 13367.9)	122360.8 (103119.2 to 145575.8)	11238 (9347 to 13367.9)	-5.72 (-5.92 to -5.53)
Colombia	2750.2 (2331.3 to 3203.7)	8519.5 (7139.5 to 10048.3)	2750.2 (2331.3 to 3203.7)	8519.5 (7139.5 to 10048.3)	-3.98 (-4.09 to -3.88)
Comoros	187.9 (170.1 to 207.6)	39801.5 (35484.9 to 44664.2)	187.9 (170.1 to 207.6)	39801.5 (35484.9 to 44664.2)	-2.59 (-2.75 to -2.44)
Congo	1165.2 (1074.7 to 1249.5)	48889.9 (44674.3 to 52797.7)	1165.2 (1074.7 to 1249.5)	48889.9 (44674.3 to 52797.7)	-1.84 (-2.17 to -1.50)
Cook Islands	1.7 (1.4 to 2)	9689.9 (7875.3 to 11787.3)	1.7 (1.4 to 2)	9689.9 (7875.3 to 11787.3)	-2.88 (-3.06 to -2.70)
Costa Rica	224.8 (191.8 to 265.9)	7560.9 (6330.3 to 9123.1)	224.8 (191.8 to 265.9)	7560.9 (6330.3 to 9123.1)	-3.29 (-3.39 to -3.19)
Croatia	560.4 (488.9 to 635.2)	12052.2 (10446.2 to 13804.1)	560.4 (488.9 to 635.2)	12052.2 (10446.2 to 13804.1)	-1.91 (-2.10 to -1.73)
Cuba	429.1 (361 to 501.6)	4099.6 (3401.6 to 4918.9)	429.1 (361 to 501.6)	4099.6 (3401.6 to 4918.9)	-2.09 (-2.30 to -1.88)
Cyprus	17.3 (14.1 to 21.2)	2447.8 (1939.2 to 3068.3)	17.3 (14.1 to 21.2)	2447.8 (1939.2 to 3068.3)	-4.56 (-5.11 to -4.00)
Czechia	989.5 (854.9 to 1145.5)	10118.4 (8648 to 11876.2)	989.5 (854.9 to 1145.5)	10118.4 (8648 to 11876.2)	-1.88 (-2.05 to -1.71)
Ivoirian	4565.9 (4119.3 to 5016.6)	38364 (34398.7 to 42372)	4565.9 (4119.3 to 5016.6)	38364 (34398.7 to 42372)	-3.18 (-3.32 to -3.04)
Democratic People's Republic of Korea	3373.1 (2870.9 to 3891)	17477.5 (14717.6 to 20450.6)	3373.1 (2870.9 to 3891)	17477.5 (14717.6 to 20450.6)	-3.33 (-3.59 to -3.07)
Democratic Republic of the Congo	16062.7 (14452.3 to 17695.2)	42167.4 (37452.4 to 46894.1)	16062.7 (14452.3 to 17695.2)	42167.4 (37452.4 to 46894.1)	-0.64 (-1.20 to -0.07)
Denmark	38.4 (32.1 to 46)	835.9 (680.5 to 1036.6)	38.4 (32.1 to 46)	835.9 (680.5 to 1036.6)	-2.02 (-2.25 to -1.79)
Djibouti	214.9 (193.3 to 235.7)	43788 (38861.4 to 48563.5)	214.9 (193.3 to 235.7)	43788 (38861.4 to 48563.5)	-3.54 (-3.77 to -3.32)
Dominica	3.8 (3.2 to 4.5)	5493.2 (4464.2 to 6650.1)	3.8 (3.2 to 4.5)	5493.2 (4464.2 to 6650.1)	-3.57 (-3.85 to -3.28)

Continued on next page

Table S13 – continued from previous page

Country	1990		2019		1990–2019
	Incident cases No.×10^4[95% UI]	ASR per 10,0000 No.[95% UI]	Incident cases No.×10^4[95% UI]	ASR per 10,0000 No.[95% UI]	EAPC No.[95% CI]
Dominican Republic	1031.7 (908.4 to 1172.1)	14482.3 (12489 to 16636.5)	1031.7 (908.4 to 1172.1)	14482.3 (12489 to 16636.5)	-4.36 (-4.70 to -4.01)
Ecuador	754.8 (630.4 to 900.7)	8176.9 (6743.2 to 9826.9)	754.8 (630.4 to 900.7)	8176.9 (6743.2 to 9826.9)	-2.72 (-3.04 to -2.40)
Egypt	4262.8 (3690.7 to 4964)	7674.9 (6504.2 to 9108.4)	4262.8 (3690.7 to 4964)	7674.9 (6504.2 to 9108.4)	-3.85 (-4.22 to -3.48)
El Salvador	1071 (931.2 to 1220.9)	20665.2 (17765.9 to 23933.9)	1071 (931.2 to 1220.9)	20665.2 (17765.9 to 23933.9)	-4.66 (-4.99 to -4.33)
Equatorial Guinea	250.2 (234.9 to 265.8)	58996.3 (54861.2 to 63142.3)	250.2 (234.9 to 265.8)	58996.3 (54861.2 to 63142.3)	-9.90 (-10.39 to -9.41)
Eritrea	1569.1 (1434.4 to 1685.1)	51808.3 (46756.8 to 56196)	1569.1 (1434.4 to 1685.1)	51808.3 (46756.8 to 56196)	-2.88 (-2.98 to -2.79)
Estonia	29.1 (24.8 to 33.4)	1921.5 (1594.2 to 2244.6)	29.1 (24.8 to 33.4)	1921.5 (1594.2 to 2244.6)	-3.48 (-3.58 to -3.37)
Eswatini	208 (175.4 to 237)	26597.5 (22241.6 to 30659.1)	208 (175.4 to 237)	26597.5 (22241.6 to 30659.1)	-3.73 (-3.84 to -3.62)
Ethiopia	28888.1 (27068.2 to 30895.9)	55371.9 (51310.2 to 59745.9)	28888.1 (27068.2 to 30895.9)	55371.9 (51310.2 to 59745.9)	-2.90 (-3.26 to -2.53)
Fiji	127 (106.3 to 149.5)	17805.6 (14697 to 21255.4)	127 (106.3 to 149.5)	17805.6 (14697 to 21255.4)	-2.61 (-2.80 to -2.42)
Finland	49.5 (41.4 to 59.8)	1108.3 (890.4 to 1390.4)	49.5 (41.4 to 59.8)	1108.3 (890.4 to 1390.4)	-2.45 (-2.67 to -2.23)
France	139.4 (118.8 to 165.9)	259.6 (214.9 to 316.2)	139.4 (118.8 to 165.9)	259.6 (214.9 to 316.2)	-0.88 (-1.08 to -0.67)
Gabon	207.4 (178.3 to 238.8)	21581.2 (18230.1 to 25152.4)	207.4 (178.3 to 238.8)	21581.2 (18230.1 to 25152.4)	-4.99 (-5.15 to -4.84)
Gambia	522.7 (487.4 to 557.2)	53208.6 (49111.2 to 56989.2)	522.7 (487.4 to 557.2)	53208.6 (49111.2 to 56989.2)	-2.93 (-2.96 to -2.90)
Georgia	143.4 (121.7 to 167.6)	2669.1 (2215.2 to 3174.3)	143.4 (121.7 to 167.6)	2669.1 (2215.2 to 3174.3)	-0.18 (-0.81 to 0.47)
Germany	373 (318 to 436.6)	509.6 (424.6 to 606.8)	373 (318 to 436.6)	509.6 (424.6 to 606.8)	-1.37 (-1.45 to -1.28)
Ghana	6913.4 (6355 to 7456.2)	47086.9 (42907.9 to 51165.7)	6913.4 (6355 to 7456.2)	47086.9 (42907.9 to 51165.7)	-3.71 (-3.90 to -3.52)
Greece	164.2 (136.7 to 198.2)	1791.3 (1445.3 to 2242.8)	164.2 (136.7 to 198.2)	1791.3 (1445.3 to 2242.8)	-2.64 (-3.06 to -2.23)
Greenland	0.7 (0.6 to 0.9)	1363.5 (1110.2 to 1646.1)	0.7 (0.6 to 0.9)	1363.5 (1110.2 to 1646.1)	-1.79 (-2.12 to -1.47)
Grenada	9.8 (8.2 to 11.5)	12004.1 (9864.5 to 14311.2)	9.8 (8.2 to 11.5)	12004.1 (9864.5 to 14311.2)	-3.64 (-4.01 to -3.28)
Guam	7.7 (6.3 to 9.4)	6082.5 (4921.6 to 7508)	7.7 (6.3 to 9.4)	6082.5 (4921.6 to 7508)	-2.60 (-2.80 to -2.40)
Guatemala	1545.6 (1356.3 to 1756.9)	19030.3 (16481.5 to 21961.7)	1545.6 (1356.3 to 1756.9)	19030.3 (16481.5 to 21961.7)	-3.95 (-4.13 to -3.77)
Guinea	3113.3 (2902 to 3326.4)	51725 (47844.9 to 55617.6)	3113.3 (2902 to 3326.4)	51725 (47844.9 to 55617.6)	-2.46 (-2.55 to -2.38)
Guinea-Bissau	567.1 (534.7 to 598.5)	57411.8 (53698.8 to 60960.4)	567.1 (534.7 to 598.5)	57411.8 (53698.8 to 60960.4)	-2.43 (-2.49 to -2.37)
Guyana	99.8 (84.6 to 117.1)	13281.7 (11114.4 to 15793.1)	99.8 (84.6 to 117.1)	13281.7 (11114.4 to 15793.1)	-3.26 (-3.40 to -3.12)
Haiti	1663.7 (1453.6 to 1879.3)	26475.9 (22772.1 to 30289.1)	1663.7 (1453.6 to 1879.3)	26475.9 (22772.1 to 30289.1)	-2.41 (-2.46 to -2.36)
Honduras	687 (589.7 to 791.5)	14344.2 (12092.6 to 16940.6)	687 (589.7 to 791.5)	14344.2 (12092.6 to 16940.6)	-3.06 (-3.27 to -2.85)
Hungary	1144.8 (994.8 to 1293.6)	11607.9 (9937 to 13385.8)	1144.8 (994.8 to 1293.6)	11607.9 (9937 to 13385.8)	-1.90 (-1.97 to -1.82)
Iceland	2.8 (2.3 to 3.5)	1218.2 (969.6 to 1536.4)	2.8 (2.3 to 3.5)	1218.2 (969.6 to 1536.4)	-2.91 (-3.12 to -2.70)
India	250998.2 (222317.5 to 278623.1)	29243.9 (25640.6 to 32812.5)	250998.2 (222317.5 to 278623.1)	29243.9 (25640.6 to 32812.5)	-4.39 (-4.71 to -4.08)
Indonesia	48858.7 (43522.1 to 54658)	27720.4 (24314.2 to 31288.3)	48858.7 (43522.1 to 54658)	27720.4 (24314.2 to 31288.3)	-5.07 (-5.17 to -4.98)
Iran (Islamic Republic of)	6380.2 (5501.5 to 7379.4)	11013.2 (9319.2 to 12973.8)	6380.2 (5501.5 to 7379.4)	11013.2 (9319.2 to 12973.8)	-7.05 (-7.57 to -6.52)

Continued on next page

Table S13 – continued from previous page

Country	1990		2019		1990–2019
	Incident cases No.×10^4[95% UI]	ASR per 10,0000 No.[95% UI]	Incident cases No.×10^4[95% UI]	ASR per 10,0000 No.[95% UI]	EAPC No.[95% CI]
Iraq	2498.6 (2126.1 to 2909.9)	14157.6 (11755.1 to 16890)	2498.6 (2126.1 to 2909.9)	14157.6 (11755.1 to 16890)	-5.16 (-5.48 to -4.84)
Ireland	42.1 (34.7 to 50.8)	1297.5 (1037.5 to 1609.8)	42.1 (34.7 to 50.8)	1297.5 (1037.5 to 1609.8)	-2.93 (-3.30 to -2.55)
Israel	413.1 (349.5 to 496.7)	9766.4 (8128.1 to 11842.8)	413.1 (349.5 to 496.7)	9766.4 (8128.1 to 11842.8)	-2.57 (-2.94 to -2.21)
Italy	1837.4 (1616 to 2117.8)	2924.5 (2492.4 to 3445.7)	1837.4 (1616 to 2117.8)	2924.5 (2492.4 to 3445.7)	-2.20 (-2.59 to -1.81)
Jamaica	145.3 (123.3 to 170.8)	6429.1 (5306.2 to 7675.7)	145.3 (123.3 to 170.8)	6429.1 (5306.2 to 7675.7)	-3.25 (-3.41 to -3.10)
Japan	1114.8 (940.3 to 1335.5)	1020.9 (839.5 to 1260.8)	1114.8 (940.3 to 1335.5)	1020.9 (839.5 to 1260.8)	-1.02 (-1.15 to -0.90)
Jordan	537.7 (458.8 to 622.7)	14577.8 (12277.1 to 17103.8)	537.7 (458.8 to 622.7)	14577.8 (12277.1 to 17103.8)	-3.70 (-4.11 to -3.28)
Kazakhstan	2532.3 (2164.5 to 2923.3)	15992.8 (13361.3 to 18738)	2532.3 (2164.5 to 2923.3)	15992.8 (13361.3 to 18738)	-2.52 (-2.61 to -2.43)
Kenya	12874.3 (11937.9 to 13753)	55426.8 (50875.7 to 59654.1)	12874.3 (11937.9 to 13753)	55426.8 (50875.7 to 59654.1)	-2.02 (-2.20 to -1.85)
Kiribati	21.9 (19.4 to 24.7)	31237.2 (27298.5 to 35627.6)	21.9 (19.4 to 24.7)	31237.2 (27298.5 to 35627.6)	-0.14 (-0.32 to 0.04)
Kuwait	39 (32 to 46.8)	2226.3 (1809.9 to 2713.2)	39 (32 to 46.8)	2226.3 (1809.9 to 2713.2)	-4.28 (-4.51 to -4.04)
Kyrgyzstan	262.9 (217.2 to 315.1)	5983.7 (4827.7 to 7260.4)	262.9 (217.2 to 315.1)	5983.7 (4827.7 to 7260.4)	-0.57 (-0.87 to -0.27)
Lao People's Democratic Republic	1490.1 (1351.1 to 1616.5)	36868.5 (33077.5 to 40334.2)	1490.1 (1351.1 to 1616.5)	36868.5 (33077.5 to 40334.2)	-4.00 (-4.27 to -3.73)
Latvia	45.2 (39 to 52.4)	1764.7 (1498 to 2073.5)	45.2 (39 to 52.4)	1764.7 (1498 to 2073.5)	-2.90 (-3.06 to -2.74)
Lebanon	229.2 (194 to 270.9)	7051.5 (5831.5 to 8495.5)	229.2 (194 to 270.9)	7051.5 (5831.5 to 8495.5)	-4.78 (-5.01 to -4.55)
Lesotho	665.3 (601.5 to 731.4)	38818.6 (34746.4 to 43118.1)	665.3 (601.5 to 731.4)	38818.6 (34746.4 to 43118.1)	-2.98 (-3.12 to -2.83)
Liberia	681.7 (615.6 to 745.3)	35237.8 (31333.3 to 39053.8)	681.7 (615.6 to 745.3)	35237.8 (31333.3 to 39053.8)	-3.69 (-3.99 to -3.39)
Libya	325.7 (269.7 to 384.4)	7697 (6236.3 to 9246.1)	325.7 (269.7 to 384.4)	7697 (6236.3 to 9246.1)	-4.60 (-5.16 to -4.05)
Lithuania	65.1 (55.4 to 76.3)	1834 (1527 to 2195)	65.1 (55.4 to 76.3)	1834 (1527 to 2195)	-3.53 (-3.63 to -3.44)
Luxembourg	2.6 (2.1 to 3.1)	751.1 (613 to 935.9)	2.6 (2.1 to 3.1)	751.1 (613 to 935.9)	-1.95 (-2.16 to -1.75)
Madagascar	4645 (4181.9 to 5142.6)	38273 (33882.5 to 42871.9)	4645 (4181.9 to 5142.6)	38273 (33882.5 to 42871.9)	-1.95 (-2.16 to -1.74)
Malawi	5104.3 (4777.4 to 5447.3)	52642.9 (48715.2 to 56769.4)	5104.3 (4777.4 to 5447.3)	52642.9 (48715.2 to 56769.4)	-2.63 (-2.86 to -2.40)
Malaysia	622.8 (515.5 to 750.7)	3706.2 (3020.7 to 4519.8)	622.8 (515.5 to 750.7)	3706.2 (3020.7 to 4519.8)	-6.23 (-6.79 to -5.67)
Maldives	67.2 (58.5 to 76.5)	31634.5 (27138.8 to 36344.9)	67.2 (58.5 to 76.5)	31634.5 (27138.8 to 36344.9)	-8.07 (-8.54 to -7.60)
Mali	4980.8 (4686.1 to 5260.9)	58646 (54848.2 to 62269.6)	4980.8 (4686.1 to 5260.9)	58646 (54848.2 to 62269.6)	-2.34 (-2.39 to -2.30)
Malta	8.1 (6.6 to 10)	2420.8 (1936.3 to 3079)	8.1 (6.6 to 10)	2420.8 (1936.3 to 3079)	-3.75 (-4.06 to -3.43)
Marshall Islands	21.5 (19.5 to 23.4)	47649.8 (42740.7 to 52278.7)	21.5 (19.5 to 23.4)	47649.8 (42740.7 to 52278.7)	-2.44 (-2.51 to -2.38)
Mauritania	653.3 (575.7 to 727)	32713.4 (28684.8 to 36674.3)	653.3 (575.7 to 727)	32713.4 (28684.8 to 36674.3)	-3.81 (-3.89 to -3.72)
Mauritius	79.7 (66.6 to 94.6)	7952.6 (6550 to 9554.5)	79.7 (66.6 to 94.6)	7952.6 (6550 to 9554.5)	-6.00 (-6.33 to -5.67)
Mexico	14604 (12592.4 to 16686.1)	18098.5 (15498.7 to 20835)	14604 (12592.4 to 16686.1)	18098.5 (15498.7 to 20835)	-3.23 (-3.32 to -3.15)

Continued on next page

Table S13 – continued from previous page

Country	1990		2019		1990–2019
	Incident cases No.×10^4[95% UI]	ASR per 10,0000 No.[95% UI]	Incident cases No.×10^4[95% UI]	ASR per 10,0000 No.[95% UI]	EAPC No.[95% CI]
Micronesia (Federated States of)	53.2 (49.1 to 57.1)	51663.1 (47261.1 to 55869.5)	53.2 (49.1 to 57.1)	51663.1 (47261.1 to 55869.5)	-1.26 (-1.34 to -1.18)
Monaco	0.1 (0.1 to 0.2)	498 (414.3 to 609.6)	0.1 (0.1 to 0.2)	498 (414.3 to 609.6)	-1.47 (-1.69 to -1.24)
Mongolia	112.4 (91.3 to 135.9)	5219.2 (4143.2 to 6446.5)	112.4 (91.3 to 135.9)	5219.2 (4143.2 to 6446.5)	-4.84 (-5.04 to -4.64)
Montenegro	57.4 (49.5 to 66)	9551.5 (8116.6 to 11129.9)	57.4 (49.5 to 66)	9551.5 (8116.6 to 11129.9)	-2.00 (-2.46 to -1.52)
Morocco	5074.3 (4390.6 to 5824.6)	20349.7 (17273.5 to 23794)	5074.3 (4390.6 to 5824.6)	20349.7 (17273.5 to 23794)	-3.96 (-4.18 to -3.74)
Mozambique	8287.3 (7829.9 to 8732.1)	62889.2 (58775 to 66874.1)	8287.3 (7829.9 to 8732.1)	62889.2 (58775 to 66874.1)	-2.90 (-3.04 to -2.76)
Myanmar	12906.2 (11509 to 14399.6)	33564.6 (29583 to 37834.3)	12906.2 (11509 to 14399.6)	33564.6 (29583 to 37834.3)	-6.73 (-6.93 to -6.54)
Namibia	209.7 (177.6 to 244.9)	15549.3 (12925 to 18449.3)	209.7 (177.6 to 244.9)	15549.3 (12925 to 18449.3)	-3.02 (-3.25 to -2.78)
Nauru	1.7 (1.4 to 2)	17234.4 (14349.9 to 20668.1)	1.7 (1.4 to 2)	17234.4 (14349.9 to 20668.1)	-0.96 (-1.66 to -0.26)
Nepal	4310.4 (3843.6 to 4859.7)	22064.4 (19326.4 to 25177.8)	4310.4 (3843.6 to 4859.7)	22064.4 (19326.4 to 25177.8)	-4.84 (-4.98 to -4.69)
Netherlands	121 (101.1 to 144.4)	902.1 (728.8 to 1114.6)	121 (101.1 to 144.4)	902.1 (728.8 to 1114.6)	-1.81 (-2.06 to -1.57)
New Zealand	23.3 (19.8 to 27.5)	741.9 (620.1 to 895.7)	23.3 (19.8 to 27.5)	741.9 (620.1 to 895.7)	-0.73 (-0.98 to -0.48)
Nicaragua	416 (353.7 to 484.5)	10557.4 (8799.7 to 12562.8)	416 (353.7 to 484.5)	10557.4 (8799.7 to 12562.8)	-6.74 (-7.28 to -6.21)
Niger	5052.9 (4794.9 to 5318.8)	63679.1 (60043.3 to 67480.1)	5052.9 (4794.9 to 5318.8)	63679.1 (60043.3 to 67480.1)	-1.22 (-1.29 to -1.14)
Nigeria	15849.7 (13899.1 to 17835.9)	17500.2 (15142.1 to 20062.8)	15849.7 (13899.1 to 17835.9)	17500.2 (15142.1 to 20062.8)	-4.35 (-4.92 to -3.78)
Niue	0.3 (0.2 to 0.3)	13632.1 (11012.6 to 16441.5)	0.3 (0.2 to 0.3)	13632.1 (11012.6 to 16441.5)	-3.03 (-3.18 to -2.88)
North Macedonia	496.7 (439.5 to 556.5)	25539.9 (22249.3 to 29042.2)	496.7 (439.5 to 556.5)	25539.9 (22249.3 to 29042.2)	-2.75 (-2.92 to -2.57)
Northern Mariana Islands	2.4 (2 to 2.8)	5523 (4544.6 to 6759.5)	2.4 (2 to 2.8)	5523 (4544.6 to 6759.5)	-0.38 (-0.64 to -0.13)
Norway	34.4 (28.5 to 41.6)	908.6 (723.7 to 1131.8)	34.4 (28.5 to 41.6)	908.6 (723.7 to 1131.8)	-2.71 (-2.96 to -2.46)
Oman	259.5 (216.4 to 308.7)	13315.5 (10911.8 to 16069.3)	259.5 (216.4 to 308.7)	13315.5 (10911.8 to 16069.3)	-7.54 (-8.10 to -6.98)
Pakistan	23561.3 (20896.1 to 26306.6)	20976.5 (18328.1 to 23917.1)	23561.3 (20896.1 to 26306.6)	20976.5 (18328.1 to 23917.1)	-6.13 (-6.46 to -5.79)
Palau	1.6 (1.3 to 1.9)	11098.7 (9032.5 to 13708.8)	1.6 (1.3 to 1.9)	11098.7 (9032.5 to 13708.8)	-2.27 (-2.44 to -2.10)
Palestine	876.7 (788.8 to 962.9)	42555.1 (37960.6 to 47276.6)	876.7 (788.8 to 962.9)	42555.1 (37960.6 to 47276.6)	-6.82 (-7.24 to -6.41)
Panama	151.5 (129.1 to 177.1)	6425.7 (5339.8 to 7730.7)	151.5 (129.1 to 177.1)	6425.7 (5339.8 to 7730.7)	-2.87 (-3.09 to -2.65)
Papua New Guinea	676.6 (573 to 781.1)	16770.3 (14040 to 19627.3)	676.6 (573 to 781.1)	16770.3 (14040 to 19627.3)	-0.65 (-1.07 to -0.24)
Paraguay	603.9 (514 to 704.8)	15515.9 (12995.9 to 18286.4)	603.9 (514 to 704.8)	15515.9 (12995.9 to 18286.4)	-2.80 (-2.95 to -2.65)
Peru	3090.5 (2704.2 to 3509.3)	14628.8 (12656 to 16754.5)	3090.5 (2704.2 to 3509.3)	14628.8 (12656 to 16754.5)	-3.19 (-3.35 to -3.04)
Philippines	11470.1 (10026.2 to 13033.1)	18913 (16316.2 to 21842.9)	11470.1 (10026.2 to 13033.1)	18913 (16316.2 to 21842.9)	-1.80 (-2.22 to -1.39)
Poland	4907.2 (4312.7 to 5609.6)	13436.3 (11586.1 to 15560.3)	4907.2 (4312.7 to 5609.6)	13436.3 (11586.1 to 15560.3)	-2.82 (-2.86 to -2.78)
Portugal	281.1 (231.9 to 335.4)	3123.2 (2496.6 to 3881.9)	281.1 (231.9 to 335.4)	3123.2 (2496.6 to 3881.9)	-3.36 (-3.73 to -2.98)

Continued on next page

Table S13 – continued from previous page

Country	1990		2019		1990–2019
	Incident cases No.×10^4[95% UI]	ASR per 10,0000 No.[95% UI]	Incident cases No.×10^4[95% UI]	ASR per 10,0000 No.[95% UI]	EAPC No.[95% CI]
Puerto Rico	97.4 (82.8 to 115.2)	2862.5 (2384.6 to 3460.2)	97.4 (82.8 to 115.2)	2862.5 (2384.6 to 3460.2)	-3.20 (-3.29 to -3.10)
Qatar	16.3 (13.2 to 20.2)	3681.3 (2955.7 to 4576.5)	16.3 (13.2 to 20.2)	3681.3 (2955.7 to 4576.5)	-5.24 (-5.47 to -5.02)
Republic of Korea	905.3 (732.7 to 1099.3)	2229.6 (1767.3 to 2746.1)	905.3 (732.7 to 1099.3)	2229.6 (1767.3 to 2746.1)	-4.18 (-4.35 to -4.01)
Republic of Moldova	155.3 (133.1 to 180.2)	3592.5 (3016 to 4221.5)	155.3 (133.1 to 180.2)	3592.5 (3016 to 4221.5)	-2.39 (-2.76 to -2.02)
Romania	3202.4 (2826.1 to 3629.4)	14416.6 (12485.1 to 16617.8)	3202.4 (2826.1 to 3629.4)	14416.6 (12485.1 to 16617.8)	-2.55 (-2.70 to -2.40)
Russian Federation	677.5 (583.1 to 787.3)	430.5 (360.7 to 514)	677.5 (583.1 to 787.3)	430.5 (360.7 to 514)	-2.47 (-2.56 to -2.39)
Rwanda	2457.9 (2202.9 to 2763.3)	33886.5 (29955.1 to 38408.3)	2457.9 (2202.9 to 2763.3)	33886.5 (29955.1 to 38408.3)	-3.12 (-3.49 to -2.76)
Saint Kitts and Nevis	2.1 (1.7 to 2.5)	5395.8 (4355.2 to 6550.9)	2.1 (1.7 to 2.5)	5395.8 (4355.2 to 6550.9)	-3.44 (-3.62 to -3.25)
Saint Lucia	9.8 (8.2 to 11.4)	7373.4 (6092.3 to 8725.5)	9.8 (8.2 to 11.4)	7373.4 (6092.3 to 8725.5)	-2.84 (-3.05 to -2.63)
Saint Vincent and the Grenadines	12.5 (10.6 to 14.6)	11757.7 (9765.7 to 14066.1)	12.5 (10.6 to 14.6)	11757.7 (9765.7 to 14066.1)	-3.69 (-3.89 to -3.49)
Samoa	36.1 (30.9 to 42)	23699.7 (19977 to 27726.2)	36.1 (30.9 to 42)	23699.7 (19977 to 27726.2)	-0.68 (-0.85 to -0.51)
San Marino	0.2 (0.1 to 0.2)	806.1 (654.5 to 1007.4)	0.2 (0.1 to 0.2)	806.1 (654.5 to 1007.4)	-1.84 (-2.18 to -1.50)
Sao Tome and Principe	56.4 (51.8 to 60.5)	47964.8 (43503.5 to 51762.3)	56.4 (51.8 to 60.5)	47964.8 (43503.5 to 51762.3)	-4.56 (-4.65 to -4.48)
Saudi Arabia	630.8 (513.2 to 796.6)	3938.5 (3124 to 5166.3)	630.8 (513.2 to 796.6)	3938.5 (3124 to 5166.3)	-8.89 (-9.74 to -8.02)
Senegal	4153.4 (3964.1 to 4347.8)	55613.7 (52913.8 to 58495.6)	4153.4 (3964.1 to 4347.8)	55613.7 (52913.8 to 58495.6)	-4.34 (-4.79 to -3.89)
Serbia	2680 (2411 to 2954.3)	29608.9 (26346.1 to 32995.9)	2680 (2411 to 2954.3)	29608.9 (26346.1 to 32995.9)	-2.36 (-2.57 to -2.15)
Seychelles	4.8 (3.9 to 5.8)	7291 (5850.6 to 8946.3)	4.8 (3.9 to 5.8)	7291 (5850.6 to 8946.3)	-5.35 (-5.93 to -4.76)
Sierra Leone	1822.2 (1699 to 1934.3)	51178.1 (47341.1 to 54712.2)	1822.2 (1699 to 1934.3)	51178.1 (47341.1 to 54712.2)	-2.63 (-2.78 to -2.49)
Singapore	53.2 (44 to 63.4)	1904.6 (1556 to 2332.8)	53.2 (44 to 63.4)	1904.6 (1556 to 2332.8)	-3.64 (-3.73 to -3.54)
Slovakia	650.2 (566.7 to 739.2)	12906.7 (11039.8 to 14864.1)	650.2 (566.7 to 739.2)	12906.7 (11039.8 to 14864.1)	-2.53 (-2.61 to -2.45)
Slovenia	156.6 (135.5 to 179.2)	8314 (7057.1 to 9661.7)	156.6 (135.5 to 179.2)	8314 (7057.1 to 9661.7)	-1.86 (-1.95 to -1.78)
Solomon Islands	139 (124.5 to 153.1)	42483.4 (37675.5 to 47221.5)	139 (124.5 to 153.1)	42483.4 (37675.5 to 47221.5)	-1.16 (-1.37 to -0.95)
Somalia	4912.2 (4641.5 to 5160.2)	68419.7 (64136.4 to 72391.4)	4912.2 (4641.5 to 5160.2)	68419.7 (64136.4 to 72391.4)	-0.30 (-0.34 to -0.27)
South Africa	4497.3 (3854.4 to 5188)	13497.2 (11455.4 to 15717.2)	4497.3 (3854.4 to 5188)	13497.2 (11455.4 to 15717.2)	-4.30 (-4.42 to -4.19)
South Sudan	2272.3 (2026.8 to 2511.9)	38240 (33691.4 to 42712.1)	2272.3 (2026.8 to 2511.9)	38240 (33691.4 to 42712.1)	-2.35 (-2.50 to -2.19)
Spain	642.6 (544.3 to 750.4)	1827.6 (1507.5 to 2187.5)	642.6 (544.3 to 750.4)	1827.6 (1507.5 to 2187.5)	-2.89 (-3.26 to -2.52)
Sri Lanka	2731.8 (2373.9 to 3127.2)	17121.1 (14723.2 to 19848.2)	2731.8 (2373.9 to 3127.2)	17121.1 (14723.2 to 19848.2)	-5.69 (-5.87 to -5.52)
Sudan	7410.2 (6682.4 to 8221.3)	36394.4 (32214.5 to 40852.6)	7410.2 (6682.4 to 8221.3)	36394.4 (32214.5 to 40852.6)	-4.54 (-4.85 to -4.23)

Continued on next page

Table S13 – continued from previous page

Country	1990		2019		1990–2019
	Incident cases No.×10^4[95% UI]	ASR per 10,0000 No.[95% UI]	Incident cases No.×10^4[95% UI]	ASR per 10,0000 No.[95% UI]	EAPC No.[95% CI]
Suriname	37.3 (31.6 to 43.7)	10037.9 (8321.2 to 11955.4)	37.3 (31.6 to 43.7)	10037.9 (8321.2 to 11955.4)	-3.17 (-3.33 to -3.00)
Sweden	86.4 (72.9 to 103.3)	1137.6 (931.4 to 1419.9)	86.4 (72.9 to 103.3)	1137.6 (931.4 to 1419.9)	-2.52 (-2.88 to -2.16)
Switzerland	32.9 (27.7 to 39.2)	535.5 (435.3 to 658.4)	32.9 (27.7 to 39.2)	535.5 (435.3 to 658.4)	-1.64 (-1.82 to -1.47)
Syrian Arab Republic	1830.5 (1526.8 to 2164)	14119.5 (11651.7 to 17057.5)	1830.5 (1526.8 to 2164)	14119.5 (11651.7 to 17057.5)	-4.62 (-4.85 to -4.39)
Taiwan (Province of China)	596.2 (481.7 to 727.8)	3253.2 (2590 to 4026.6)	596.2 (481.7 to 727.8)	3253.2 (2590 to 4026.6)	-5.51 (-5.75 to -5.26)
Tajikistan	709.2 (608.1 to 822.7)	13136.7 (11104.9 to 15449.2)	709.2 (608.1 to 822.7)	13136.7 (11104.9 to 15449.2)	-1.69 (-2.24 to -1.13)
Thailand	8028.8 (6965 to 9153.5)	15181.1 (13069.4 to 17425.4)	8028.8 (6965 to 9153.5)	15181.1 (13069.4 to 17425.4)	-7.44 (-7.89 to -6.98)
Timor-Leste	273.4 (245.2 to 300.8)	36026.7 (31926.9 to 40171.7)	273.4 (245.2 to 300.8)	36026.7 (31926.9 to 40171.7)	-6.00 (-6.45 to -5.56)
Togo	1668.4 (1539 to 1803.8)	46624.4 (42655.9 to 50759.7)	1668.4 (1539 to 1803.8)	46624.4 (42655.9 to 50759.7)	-2.83 (-2.96 to -2.70)
Tokelau	0.4 (0.3 to 0.4)	24927.5 (20985.3 to 28770.8)	0.4 (0.3 to 0.4)	24927.5 (20985.3 to 28770.8)	-3.02 (-3.20 to -2.84)
Tonga	20.6 (17.6 to 24)	22923.7 (19285.1 to 27024.5)	20.6 (17.6 to 24)	22923.7 (19285.1 to 27024.5)	-1.73 (-1.96 to -1.50)
Trinidad and Tobago	54.8 (45.8 to 65.1)	4752.1 (3899.9 to 5745.8)	54.8 (45.8 to 65.1)	4752.1 (3899.9 to 5745.8)	-3.48 (-3.70 to -3.25)
Tunisia	691.7 (578.2 to 820.7)	8202.7 (6753.4 to 10020.2)	691.7 (578.2 to 820.7)	8202.7 (6753.4 to 10020.2)	-5.23 (-5.50 to -4.95)
Turkey	8301.1 (7435.6 to 9192.9)	13891.2 (12333.7 to 15509.1)	8301.1 (7435.6 to 9192.9)	13891.2 (12333.7 to 15509.1)	-5.20 (-5.35 to -5.06)
Turkmenistan	210.2 (175.8 to 252.5)	5700.1 (4653.3 to 7018.3)	210.2 (175.8 to 252.5)	5700.1 (4653.3 to 7018.3)	-3.44 (-3.77 to -3.10)
Tuvalu	2.5 (2.2 to 2.8)	29113.6 (25597.7 to 33303.1)	2.5 (2.2 to 2.8)	29113.6 (25597.7 to 33303.1)	-1.88 (-2.04 to -1.72)
Uganda	5658.5 (5067.1 to 6280.4)	31699.6 (27839.9 to 35685.8)	5658.5 (5067.1 to 6280.4)	31699.6 (27839.9 to 35685.8)	-3.60 (-3.78 to -3.42)
Ukraine	1083.4 (932.7 to 1254.5)	2138.5 (1807.2 to 2517.9)	1083.4 (932.7 to 1254.5)	2138.5 (1807.2 to 2517.9)	-1.67 (-1.86 to -1.48)
United Arab Emirates	60.3 (49.6 to 72.9)	3233.2 (2620.1 to 3937.2)	60.3 (49.6 to 72.9)	3233.2 (2620.1 to 3937.2)	-4.80 (-5.24 to -4.36)
United Kingdom	661.2 (552.6 to 788.4)	1297.1 (1045.1 to 1600.6)	661.2 (552.6 to 788.4)	1297.1 (1045.1 to 1600.6)	-2.40 (-2.69 to -2.10)
United Republic of Tanzania	7859.9 (7052.8 to 8714.1)	29513.2 (26147.2 to 33084.7)	7859.9 (7052.8 to 8714.1)	29513.2 (26147.2 to 33084.7)	-2.36 (-2.59 to -2.13)
United States of America	1560.8 (1319.6 to 1810.1)	690.9 (574 to 816.6)	1560.8 (1319.6 to 1810.1)	690.9 (574 to 816.6)	-1.80 (-2.05 to -1.55)
United States Virgin Islands	3.3 (2.7 to 3.8)	3211.7 (2669.7 to 3806.3)	3.3 (2.7 to 3.8)	3211.7 (2669.7 to 3806.3)	-4.01 (-4.25 to -3.78)
Uruguay	230.1 (189 to 274.4)	8558.6 (6883.7 to 10362)	230.1 (189 to 274.4)	8558.6 (6883.7 to 10362)	-1.58 (-1.84 to -1.32)
Uzbekistan	1688.1 (1367.9 to 2031.2)	8104 (6441 to 9928.1)	1688.1 (1367.9 to 2031.2)	8104 (6441 to 9928.1)	-3.37 (-3.60 to -3.15)
Vanuatu	59.4 (53.2 to 65.5)	41097 (36427.9 to 45759.3)	59.4 (53.2 to 65.5)	41097 (36427.9 to 45759.3)	-1.18 (-1.33 to -1.03)

Continued on next page

Table S13 – continued from previous page

Country	1990		2019		1990–2019
	Incident cases No.$\times 10^4$[95% UI]	ASR per 10,0000 No.[95% UI]	Incident cases No.$\times 10^4$[95% UI]	ASR per 10,0000 No.[95% UI]	EAPC No.[95% CI]
Venezuela (Bolivarian Republic of)	1637.6 (1403.5 to 1889.1)	8781.6 (7390.3 to 10406)	1637.6 (1403.5 to 1889.1)	8781.6 (7390.3 to 10406)	-1.31 (-1.85 to -0.77)
Viet Nam	5671.5 (4750.4 to 6662.5)	9302.6 (7714.6 to 11115.9)	5671.5 (4750.4 to 6662.5)	9302.6 (7714.6 to 11115.9)	-5.28 (-5.58 to -4.99)
Yemen	6164.8 (5622.5 to 6737.4)	44607.6 (40223.6 to 49336.3)	6164.8 (5622.5 to 6737.4)	44607.6 (40223.6 to 49336.3)	-4.49 (-4.80 to -4.17)
Zambia	3113.4 (2820.8 to 3434.3)	38626.3 (34505.6 to 43029.2)	3113.4 (2820.8 to 3434.3)	38626.3 (34505.6 to 43029.2)	-3.76 (-4.20 to -3.31)
Zimbabwe	2959.8 (2609.9 to 3317.3)	29228.5 (25411 to 33119.8)	2959.8 (2609.9 to 3317.3)	29228.5 (25411 to 33119.8)	-0.61 (-0.96 to -0.26)

Abbreviation: ASR, age standardized rate; CI, confidence interval; EAPC, estimated annual percentage change; UI, uncertainty interval.

Table S14: The change of vitamin A deficiency cases between 1990 and 2019 at national level for female.

Country	1990		2019		1990–2019
	Incident cases No.$\times 10^4$[95% UI]	ASR per 10,0000 No.[95% UI]	Incident cases No.$\times 10^4$[95% UI]	ASR per 10,0000 No.[95% UI]	EAPC No.[95% CI]
Afghanistan	1979.4 (1742.2 to 2245.4)	34238.3 (29444.4 to 39519.2)	1979.4 (1742.2 to 2245.4)	34238.3 (29444.4 to 39519.2)	-1.61 (-2.08 to -1.14)
Albania	468.2 (405.6 to 536)	29227.6 (24936.3 to 33987.2)	468.2 (405.6 to 536)	29227.6 (24936.3 to 33987.2)	-2.77 (-2.98 to -2.56)
Algeria	1021.3 (831.4 to 1250.9)	8237.2 (6553.4 to 10318.3)	1021.3 (831.4 to 1250.9)	8237.2 (6553.4 to 10318.3)	-4.21 (-4.40 to -4.02)
American Samoa	2.1 (1.7 to 2.6)	9350.3 (7366.4 to 11830.2)	2.1 (1.7 to 2.6)	9350.3 (7366.4 to 11830.2)	-2.03 (-2.24 to -1.81)
Andorra	0.2 (0.1 to 0.2)	678 (525.7 to 844.8)	0.2 (0.1 to 0.2)	678 (525.7 to 844.8)	-1.45 (-1.68 to -1.23)
Angola	1752.7 (1532.1 to 1987.9)	34483.9 (29559.1 to 39645.6)	1752.7 (1532.1 to 1987.9)	34483.9 (29559.1 to 39645.6)	-3.78 (-4.17 to -3.37)
Antigua and Barbuda	1.3 (1.1 to 1.6)	4462.8 (3560 to 5475.4)	1.3 (1.1 to 1.6)	4462.8 (3560 to 5475.4)	-2.49 (-2.59 to -2.39)
Argentina	1584.1 (1342 to 1855.1)	9912 (8207.9 to 11796.6)	1584.1 (1342 to 1855.1)	9912 (8207.9 to 11796.6)	-1.15 (-1.35 to -0.94)
Armenia	18.6 (15.4 to 22.2)	1041.7 (836 to 1280.5)	18.6 (15.4 to 22.2)	1041.7 (836 to 1280.5)	-2.14 (-2.54 to -1.75)
Australia	18 (14.7 to 21.5)	172.1 (140 to 206.7)	18 (14.7 to 21.5)	172.1 (140 to 206.7)	-0.99 (-1.38 to -0.60)
Austria	40.6 (33.8 to 49.6)	1148 (928 to 1447.2)	40.6 (33.8 to 49.6)	1148 (928 to 1447.2)	-2.21 (-2.49 to -1.93)
Azerbaijan	133.8 (109.5 to 162.1)	3570.8 (2868.8 to 4398)	133.8 (109.5 to 162.1)	3570.8 (2868.8 to 4398)	-1.74 (-2.27 to -1.21)
Bahamas	3.6 (2.9 to 4.5)	2851.3 (2290 to 3609)	3.6 (2.9 to 4.5)	2851.3 (2290 to 3609)	-2.41 (-2.60 to -2.23)
Bahrain	9.8 (7.9 to 12.2)	4604.5 (3639.4 to 5807.9)	9.8 (7.9 to 12.2)	4604.5 (3639.4 to 5807.9)	-3.50 (-3.65 to -3.35)
Bangladesh	9097.8 (7746.7 to 10666.1)	17083.2 (14253.9 to 20370.8)	9097.8 (7746.7 to 10666.1)	17083.2 (14253.9 to 20370.8)	-3.65 (-3.98 to -3.31)
Barbados	4.5 (3.7 to 5.3)	3627.3 (2913.4 to 4401.1)	4.5 (3.7 to 5.3)	3627.3 (2913.4 to 4401.1)	-1.66 (-1.74 to -1.58)

Continued on next page

Table S14 – continued from previous page

Country	1990		2019		1990–2019
	Incident cases No.×10⁴[95% UI]	ASR per 10,0000 No.[95% UI]	Incident cases No.×10⁴[95% UI]	ASR per 10,0000 No.[95% UI]	EAPC No.[95% CI]
Belarus	203.1 (171.6 to 238.8)	3754.9 (3113.1 to 4502.2)	203.1 (171.6 to 238.8)	3754.9 (3113.1 to 4502.2)	-2.88 (-3.13 to -2.63)
Belgium	51.4 (42.6 to 62.8)	1150 (911.6 to 1448.1)	51.4 (42.6 to 62.8)	1150 (911.6 to 1448.1)	-2.09 (-2.37 to -1.80)
Belize	10.3 (8.5 to 12.5)	11191.7 (9025.1 to 13818.5)	10.3 (8.5 to 12.5)	11191.7 (9025.1 to 13818.5)	-2.92 (-3.07 to -2.78)
Benin	795.1 (676.1 to 916)	32922.6 (27554.1 to 38452.3)	795.1 (676.1 to 916)	32922.6 (27554.1 to 38452.3)	-1.86 (-2.08 to -1.64)
Bermuda	0.8 (0.6 to 0.9)	2728.8 (2208.6 to 3370.3)	0.8 (0.6 to 0.9)	2728.8 (2208.6 to 3370.3)	-2.53 (-2.67 to -2.39)
Bhutan	60.1 (50.5 to 71)	20745.3 (16942.3 to 24642)	60.1 (50.5 to 71)	20745.3 (16942.3 to 24642)	-4.43 (-4.51 to -4.34)
Bolivia (Plurinational State of)	467.6 (388.4 to 555.9)	14792.9 (11999.5 to 17890.7)	467.6 (388.4 to 555.9)	14792.9 (11999.5 to 17890.7)	-1.63 (-1.80 to -1.45)
Bosnia and Herzegovina	596.2 (517.6 to 677.8)	26524.3 (22610 to 30474.3)	596.2 (517.6 to 677.8)	26524.3 (22610 to 30474.3)	-3.20 (-3.43 to -2.97)
Botswana	149.2 (124.8 to 175)	22864.9 (18992.4 to 27325.4)	149.2 (124.8 to 175)	22864.9 (18992.4 to 27325.4)	-3.19 (-3.32 to -3.06)
Brazil	16211.2 (14207.6 to 18422.4)	21935.8 (18931.4 to 25192.5)	16211.2 (14207.6 to 18422.4)	21935.8 (18931.4 to 25192.5)	-3.00 (-3.06 to -2.94)
Brunei Darussalam	3.2 (2.5 to 4)	2757.3 (2149 to 3532.8)	3.2 (2.5 to 4)	2757.3 (2149 to 3532.8)	-2.79 (-2.88 to -2.70)
Bulgaria	755.9 (652.9 to 870.9)	17535.1 (14742.7 to 20377.5)	755.9 (652.9 to 870.9)	17535.1 (14742.7 to 20377.5)	-1.48 (-1.69 to -1.26)
Burkina Faso	1930.4 (1687.6 to 2186.7)	40103.4 (34517.8 to 45943.4)	1930.4 (1687.6 to 2186.7)	40103.4 (34517.8 to 45943.4)	-2.38 (-2.53 to -2.23)
Burundi	848.1 (727.8 to 978)	29299.6 (24641.7 to 34274.3)	848.1 (727.8 to 978)	29299.6 (24641.7 to 34274.3)	-1.83 (-2.06 to -1.59)
Cabo Verde	27.5 (22.5 to 33.2)	15676.5 (12546 to 19162.4)	27.5 (22.5 to 33.2)	15676.5 (12546 to 19162.4)	-4.88 (-4.98 to -4.77)
Cambodia	1646.1 (1422.9 to 1875.5)	31313 (26604 to 36190.9)	1646.1 (1422.9 to 1875.5)	31313 (26604 to 36190.9)	-4.89 (-5.03 to -4.75)
Cameroon	2396.4 (2177.2 to 2628.8)	47299.3 (42634.2 to 52173.2)	2396.4 (2177.2 to 2628.8)	47299.3 (42634.2 to 52173.2)	-4.03 (-4.15 to -3.91)
Canada	256.8 (208.8 to 318.5)	2207 (1755.4 to 2763.4)	256.8 (208.8 to 318.5)	2207 (1755.4 to 2763.4)	-2.26 (-2.49 to -2.02)
Central African Republic	384 (329.8 to 438.8)	27871.6 (23632 to 32457.2)	384 (329.8 to 438.8)	27871.6 (23632 to 32457.2)	-1.16 (-1.37 to -0.95)
Chad	1243.2 (1082.1 to 1398)	41203.8 (35385.7 to 46981.6)	1243.2 (1082.1 to 1398)	41203.8 (35385.7 to 46981.6)	-2.06 (-2.27 to -1.85)
Chile	419.1 (336.6 to 519.5)	6823.2 (5377.2 to 8585.7)	419.1 (336.6 to 519.5)	6823.2 (5377.2 to 8585.7)	-2.22 (-2.40 to -2.04)
China	39048.9 (32446.4 to 46258.4)	7414.7 (6087.8 to 8856.7)	39048.9 (32446.4 to 46258.4)	7414.7 (6087.8 to 8856.7)	-4.79 (-5.15 to -4.42)
Colombia	1254.4 (1025.6 to 1498.4)	7678.9 (6147.4 to 9364.3)	1254.4 (1025.6 to 1498.4)	7678.9 (6147.4 to 9364.3)	-3.70 (-3.81 to -3.60)
Comoros	72.1 (61.9 to 82.8)	30485.7 (25630.5 to 35463.9)	72.1 (61.9 to 82.8)	30485.7 (25630.5 to 35463.9)	-2.75 (-2.89 to -2.61)
Congo	391.6 (337.8 to 449.4)	32449.5 (27536.1 to 37731.1)	391.6 (337.8 to 449.4)	32449.5 (27536.1 to 37731.1)	-2.13 (-2.58 to -1.69)
Cook Islands	0.7 (0.5 to 0.8)	7781.6 (6099.9 to 9855.1)	0.7 (0.5 to 0.8)	7781.6 (6099.9 to 9855.1)	-2.91 (-3.03 to -2.78)
Costa Rica	101.1 (84.2 to 122.5)	6818.9 (5508.1 to 8452)	101.1 (84.2 to 122.5)	6818.9 (5508.1 to 8452)	-3.02 (-3.10 to -2.94)
Croatia	364.5 (309.9 to 424.1)	14795.3 (12424.6 to 17366.9)	364.5 (309.9 to 424.1)	14795.3 (12424.6 to 17366.9)	-1.59 (-1.74 to -1.44)
Cuba	206.9 (171.7 to 249.1)	3942.9 (3208.1 to 4823.3)	206.9 (171.7 to 249.1)	3942.9 (3208.1 to 4823.3)	-1.83 (-2.01 to -1.66)
Cyprus	7.3 (5.8 to 9.2)	2097.4 (1605.5 to 2695.8)	7.3 (5.8 to 9.2)	2097.4 (1605.5 to 2695.8)	-4.00 (-4.50 to -3.50)

Continued on next page

Table S14 – continued from previous page

Country	1990		2019		1990–2019
	Incident cases No.×10^4[95% UI]	ASR per 10,0000 No.[95% UI]	Incident cases No.×10^4[95% UI]	ASR per 10,0000 No.[95% UI]	EAPC No.[95% CI]
Czechia	665.1 (557.2 to 776.5)	12846.2 (10602.1 to 15275.3)	665.1 (557.2 to 776.5)	12846.2 (10602.1 to 15275.3)	-1.59 (-1.75 to -1.44)
Ivoirian	1947.7 (1687.1 to 2196.4)	33314.5 (28639.4 to 37871.5)	1947.7 (1687.1 to 2196.4)	33314.5 (28639.4 to 37871.5)	-2.79 (-2.96 to -2.63)
Democratic People's Republic of Korea	1252.4 (1025.8 to 1498.5)	12344.6 (9938.1 to 14916.6)	1252.4 (1025.8 to 1498.5)	12344.6 (9938.1 to 14916.6)	-2.95 (-3.27 to -2.63)
Democratic Republic of the Congo	5836.2 (4993.6 to 6704.3)	30441.1 (25510 to 35548.4)	5836.2 (4993.6 to 6704.3)	30441.1 (25510 to 35548.4)	-0.56 (-1.21 to 0.10)
Denmark	19 (15.5 to 22.9)	826.7 (653 to 1033.1)	19 (15.5 to 22.9)	826.7 (653 to 1033.1)	-1.71 (-1.95 to -1.46)
Djibouti	72.9 (63 to 83.6)	32300.9 (27481.7 to 37661.2)	72.9 (63 to 83.6)	32300.9 (27481.7 to 37661.2)	-3.19 (-3.41 to -2.98)
Dominica	1.9 (1.6 to 2.3)	5500.8 (4395 to 6817.9)	1.9 (1.6 to 2.3)	5500.8 (4395 to 6817.9)	-3.13 (-3.37 to -2.90)
Dominican Republic	472.2 (398.9 to 549.7)	12983 (10778.2 to 15466.4)	472.2 (398.9 to 549.7)	12983 (10778.2 to 15466.4)	-3.83 (-4.15 to -3.50)
Ecuador	367.6 (297.9 to 442.1)	7875.9 (6307.4 to 9604)	367.6 (297.9 to 442.1)	7875.9 (6307.4 to 9604)	-2.31 (-2.64 to -1.97)
Egypt	1844.4 (1546.6 to 2168.7)	6760.2 (5531.3 to 8102)	1844.4 (1546.6 to 2168.7)	6760.2 (5531.3 to 8102)	-3.29 (-3.66 to -2.93)
El Salvador	449.4 (381.6 to 523.7)	17018.6 (14145.3 to 20118.5)	449.4 (381.6 to 523.7)	17018.6 (14145.3 to 20118.5)	-4.16 (-4.47 to -3.85)
Equatorial Guinea	96.2 (86.3 to 106.8)	43697.5 (38483.2 to 49216.6)	96.2 (86.3 to 106.8)	43697.5 (38483.2 to 49216.6)	-9.88 (-10.32 to -9.43)
Eritrea	626.5 (553.8 to 702.3)	41468.5 (35989.2 to 47047)	626.5 (553.8 to 702.3)	41468.5 (35989.2 to 47047)	-2.93 (-3.02 to -2.84)
Estonia	18.2 (15.1 to 21.6)	2250.4 (1832.9 to 2717.6)	18.2 (15.1 to 21.6)	2250.4 (1832.9 to 2717.6)	-2.86 (-2.94 to -2.77)
Eswatini	86.7 (70.9 to 103.8)	21088 (16993.7 to 25529.2)	86.7 (70.9 to 103.8)	21088 (16993.7 to 25529.2)	-3.28 (-3.42 to -3.14)
Ethiopia	12578.4 (11348.8 to 13824.7)	48784.6 (43552 to 54345)	12578.4 (11348.8 to 13824.7)	48784.6 (43552 to 54345)	-3.00 (-3.36 to -2.63)
Fiji	36.2 (29.5 to 43.8)	10187.1 (8120 to 12611.8)	36.2 (29.5 to 43.8)	10187.1 (8120 to 12611.8)	-3.37 (-3.65 to -3.09)
Finland	24.3 (20.1 to 30.1)	1072.5 (846.1 to 1368.4)	24.3 (20.1 to 30.1)	1072.5 (846.1 to 1368.4)	-2.05 (-2.28 to -1.81)
France	68.4 (56.5 to 83.3)	251 (201.6 to 318.3)	68.4 (56.5 to 83.3)	251 (201.6 to 318.3)	-0.66 (-0.89 to -0.43)
Gabon	65.2 (53.3 to 78.8)	13517.3 (10718.9 to 16684)	65.2 (53.3 to 78.8)	13517.3 (10718.9 to 16684)	-4.44 (-4.62 to -4.26)
Gambia	176 (153.3 to 199.9)	36157.9 (31216.2 to 41572.2)	176 (153.3 to 199.9)	36157.9 (31216.2 to 41572.2)	-2.72 (-2.78 to -2.66)
Georgia	69 (57.3 to 82.7)	2406.7 (1954.9 to 2948.4)	69 (57.3 to 82.7)	2406.7 (1954.9 to 2948.4)	-0.07 (-0.61 to 0.47)
Germany	168.9 (141.4 to 201.2)	451.9 (367.3 to 551.8)	168.9 (141.4 to 201.2)	451.9 (367.3 to 551.8)	-1.05 (-1.15 to -0.96)
Ghana	2214.7 (1901.7 to 2548.9)	30409.9 (25475.5 to 35379.8)	2214.7 (1901.7 to 2548.9)	30409.9 (25475.5 to 35379.8)	-3.85 (-4.14 to -3.56)
Greece	76.2 (62.3 to 93)	1653.4 (1301 to 2102.3)	76.2 (62.3 to 93)	1653.4 (1301 to 2102.3)	-2.35 (-2.73 to -1.96)
Greenland	0.5 (0.4 to 0.7)	2187.2 (1720.4 to 2767.8)	0.5 (0.4 to 0.7)	2187.2 (1720.4 to 2767.8)	-2.01 (-2.20 to -1.83)
Grenada	4.4 (3.6 to 5.3)	10707.1 (8576 to 12888.8)	4.4 (3.6 to 5.3)	10707.1 (8576 to 12888.8)	-3.19 (-3.51 to -2.87)
Guam	3.1 (2.5 to 3.9)	5206.7 (4077.3 to 6661.8)	3.1 (2.5 to 3.9)	5206.7 (4077.3 to 6661.8)	-2.61 (-2.83 to -2.39)

Continued on next page

Table S14 – continued from previous page

Country	1990		2019		1990–2019
	Incident cases No.×10^4[95% UI]	ASR per 10,0000 No.[95% UI]	Incident cases No.×10^4[95% UI]	ASR per 10,0000 No.[95% UI]	EAPC No.[95% CI]
Guatemala	692.5 (588.7 to 806.3)	16951.2 (14072.4 to 20047.8)	692.5 (588.7 to 806.3)	16951.2 (14072.4 to 20047.8)	-3.66 (-3.81 to -3.51)
Guinea	971.7 (832 to 1126.2)	31808.6 (26764.3 to 37419.5)	971.7 (832 to 1126.2)	31808.6 (26764.3 to 37419.5)	-2.20 (-2.28 to -2.12)
Guinea-Bissau	183.5 (160.3 to 207)	36282.6 (31205.1 to 41407.8)	183.5 (160.3 to 207)	36282.6 (31205.1 to 41407.8)	-2.31 (-2.42 to -2.20)
Guyana	43.4 (35.6 to 52.5)	11376.1 (9142.2 to 13995.7)	43.4 (35.6 to 52.5)	11376.1 (9142.2 to 13995.7)	-2.77 (-2.88 to -2.65)
Haiti	753 (647.6 to 871.2)	23315.4 (19643.9 to 27670.2)	753 (647.6 to 871.2)	23315.4 (19643.9 to 27670.2)	-2.14 (-2.19 to -2.10)
Honduras	308.2 (259.2 to 365.5)	12809.9 (10561.2 to 15497.3)	308.2 (259.2 to 365.5)	12809.9 (10561.2 to 15497.3)	-2.72 (-2.90 to -2.54)
Hungary	775.4 (661.8 to 892.8)	14694.4 (12333.7 to 17182.6)	775.4 (661.8 to 892.8)	14694.4 (12333.7 to 17182.6)	-1.62 (-1.68 to -1.55)
Iceland	1.3 (1.1 to 1.7)	1170 (904 to 1501.5)	1.3 (1.1 to 1.7)	1170 (904 to 1501.5)	-2.45 (-2.67 to -2.22)
India	84667.4 (72836 to 97310.3)	20750.9 (17599.6 to 24219.2)	84667.4 (72836 to 97310.3)	20750.9 (17599.6 to 24219.2)	-4.00 (-4.33 to -3.67)
Indonesia	21399.1 (18878.8 to 24044.7)	23824.1 (20599.5 to 27240)	21399.1 (18878.8 to 24044.7)	23824.1 (20599.5 to 27240)	-4.25 (-4.40 to -4.10)
Iran (Islamic Republic of)	2234.3 (1834.1 to 2663)	7967.1 (6439.8 to 9741.4)	2234.3 (1834.1 to 2663)	7967.1 (6439.8 to 9741.4)	-6.53 (-7.03 to -6.04)
Iraq	1003.6 (819.9 to 1211.5)	11628.1 (9231.2 to 14401.4)	1003.6 (819.9 to 1211.5)	11628.1 (9231.2 to 14401.4)	-4.93 (-5.16 to -4.70)
Ireland	21 (16.5 to 26.4)	1300 (984.1 to 1690.1)	21 (16.5 to 26.4)	1300 (984.1 to 1690.1)	-2.63 (-2.99 to -2.26)
Israel	168.5 (135.6 to 207.6)	7938.1 (6237.9 to 10005.9)	168.5 (135.6 to 207.6)	7938.1 (6237.9 to 10005.9)	-2.15 (-2.50 to -1.81)
Italy	830.3 (711.7 to 963.2)	2570.6 (2141.5 to 3073)	830.3 (711.7 to 963.2)	2570.6 (2141.5 to 3073)	-2.02 (-2.38 to -1.65)
Jamaica	74.9 (61.7 to 91.2)	6481.8 (5216.8 to 8076.5)	74.9 (61.7 to 91.2)	6481.8 (5216.8 to 8076.5)	-2.85 (-2.98 to -2.72)
Japan	644.6 (535.8 to 784.6)	1158 (934.7 to 1452)	644.6 (535.8 to 784.6)	1158 (934.7 to 1452)	-1.03 (-1.16 to -0.90)
Jordan	206.1 (169.7 to 247.9)	11782.8 (9580.4 to 14387.1)	206.1 (169.7 to 247.9)	11782.8 (9580.4 to 14387.1)	-3.32 (-3.70 to -2.94)
Kazakhstan	661.7 (553.8 to 784.6)	8148.7 (6644.8 to 9901.9)	661.7 (553.8 to 784.6)	8148.7 (6644.8 to 9901.9)	-2.06 (-2.15 to -1.96)
Kenya	6250.8 (5676.8 to 6845)	53329.3 (47814.4 to 59180.8)	6250.8 (5676.8 to 6845)	53329.3 (47814.4 to 59180.8)	-1.92 (-2.10 to -1.73)
Kiribati	8.6 (7.2 to 10)	24260.9 (20102.4 to 28836.1)	8.6 (7.2 to 10)	24260.9 (20102.4 to 28836.1)	-0.33 (-0.50 to -0.17)
Kuwait	17.1 (13.7 to 21.2)	2288.9 (1799.1 to 2853)	17.1 (13.7 to 21.2)	2288.9 (1799.1 to 2853)	-4.01 (-4.21 to -3.82)
Kyrgyzstan	111.9 (92.3 to 136.5)	4929.9 (3948.7 to 6103.8)	111.9 (92.3 to 136.5)	4929.9 (3948.7 to 6103.8)	-0.28 (-0.58 to 0.03)
Lao People's Democratic Republic	642.9 (568.2 to 720.6)	31265.8 (27334.1 to 35390.1)	642.9 (568.2 to 720.6)	31265.8 (27334.1 to 35390.1)	-3.72 (-3.97 to -3.46)
Latvia	28.9 (24.3 to 34.1)	2102.6 (1738.9 to 2530.9)	28.9 (24.3 to 34.1)	2102.6 (1738.9 to 2530.9)	-2.34 (-2.51 to -2.16)
Lebanon	106 (86.8 to 127.4)	6590.6 (5243.8 to 8079)	106 (86.8 to 127.4)	6590.6 (5243.8 to 8079)	-4.47 (-4.66 to -4.28)
Lesotho	271.4 (234.6 to 309)	30916 (26311.3 to 35746)	271.4 (234.6 to 309)	30916 (26311.3 to 35746)	-2.87 (-3.03 to -2.70)
Liberia	130.1 (105.7 to 158.8)	13365 (10655.5 to 16698.4)	130.1 (105.7 to 158.8)	13365 (10655.5 to 16698.4)	-3.44 (-4.03 to -2.85)
Libya	139.1 (111.6 to 170.9)	6885.1 (5357.4 to 8706.7)	139.1 (111.6 to 170.9)	6885.1 (5357.4 to 8706.7)	-4.40 (-4.93 to -3.87)
Lithuania	41 (34.2 to 49.2)	2176.4 (1772 to 2656.5)	41 (34.2 to 49.2)	2176.4 (1772 to 2656.5)	-2.97 (-3.08 to -2.86)
Luxembourg	1.3 (1.1 to 1.6)	745.3 (595 to 941.8)	1.3 (1.1 to 1.6)	745.3 (595 to 941.8)	-1.60 (-1.82 to -1.39)
Madagascar	1823.1 (1561.2 to 2088.1)	30188.9 (25323.7 to 34994.7)	1823.1 (1561.2 to 2088.1)	30188.9 (25323.7 to 34994.7)	-1.75 (-1.97 to -1.52)

Continued on next page

Table S14 – continued from previous page

Country	1990		2019		1990–2019
	Incident cases No.×10^4[95% UI]	ASR per 10,0000 No.[95% UI]	Incident cases No.×10^4[95% UI]	ASR per 10,0000 No.[95% UI]	EAPC No.[95% CI]
Malawi	2417.5 (2174.3 to 2641.6)	48764.1 (43351.9 to 53927.7)	2417.5 (2174.3 to 2641.6)	48764.1 (43351.9 to 53927.7)	-2.12 (-2.41 to -1.83)
Malaysia	312.8 (250.5 to 386.7)	3752 (2959.2 to 4677.3)	312.8 (250.5 to 386.7)	3752 (2959.2 to 4677.3)	-5.54 (-6.09 to -4.98)
Maldives	28.1 (23.3 to 33)	26707.8 (21861.9 to 31860.8)	28.1 (23.3 to 33)	26707.8 (21861.9 to 31860.8)	-6.93 (-7.43 to -6.44)
Mali	1706.4 (1495.3 to 1917.6)	39935.1 (34331.1 to 45223.8)	1706.4 (1495.3 to 1917.6)	39935.1 (34331.1 to 45223.8)	-2.03 (-2.10 to -1.95)
Malta	3.5 (2.8 to 4.3)	2075.7 (1614.4 to 2610.2)	3.5 (2.8 to 4.3)	2075.7 (1614.4 to 2610.2)	-3.19 (-3.49 to -2.89)
Marshall Islands	8.6 (7.5 to 9.8)	39397.3 (33562.8 to 45111.4)	8.6 (7.5 to 9.8)	39397.3 (33562.8 to 45111.4)	-2.75 (-2.79 to -2.70)
Mauritania	207.8 (174.8 to 243.3)	20922.8 (17403.3 to 24876)	207.8 (174.8 to 243.3)	20922.8 (17403.3 to 24876)	-3.21 (-3.35 to -3.06)
Mauritius	37.6 (30.9 to 45.8)	7437.7 (6006.3 to 9211.5)	37.6 (30.9 to 45.8)	7437.7 (6006.3 to 9211.5)	-5.36 (-5.64 to -5.08)
Mexico	5432.9 (4595 to 6284.9)	13272.5 (11159 to 15531.3)	5432.9 (4595 to 6284.9)	13272.5 (11159 to 15531.3)	-3.11 (-3.20 to -3.03)
Micronesia (Federated States of)	21.1 (18.8 to 23.4)	42417.7 (37228.9 to 47564.3)	21.1 (18.8 to 23.4)	42417.7 (37228.9 to 47564.3)	-1.35 (-1.48 to -1.23)
Monaco	0.1 (0.1 to 0.1)	531.5 (428.9 to 654.5)	0.1 (0.1 to 0.1)	531.5 (428.9 to 654.5)	-1.24 (-1.48 to -1.01)
Mongolia	39 (31.3 to 48)	3603.6 (2837.8 to 4522.9)	39 (31.3 to 48)	3603.6 (2837.8 to 4522.9)	-3.82 (-3.93 to -3.71)
Montenegro	37.4 (32 to 43.8)	12056.4 (10048.2 to 14338)	37.4 (32 to 43.8)	12056.4 (10048.2 to 14338)	-1.59 (-2.00 to -1.18)
Morocco	1807.9 (1541.3 to 2124.1)	14612.9 (12212.2 to 17604.4)	1807.9 (1541.3 to 2124.1)	14612.9 (12212.2 to 17604.4)	-3.72 (-3.94 to -3.50)
Mozambique	3434.3 (3090.7 to 3771.2)	50047.1 (44304.1 to 55459.1)	3434.3 (3090.7 to 3771.2)	50047.1 (44304.1 to 55459.1)	-2.97 (-3.11 to -2.84)
Myanmar	5506.9 (4722.3 to 6384)	28160.1 (23824.3 to 33040.9)	5506.9 (4722.3 to 6384)	28160.1 (23824.3 to 33040.9)	-6.02 (-6.17 to -5.87)
Namibia	90.8 (74.6 to 110.2)	13137.1 (10587.7 to 16210)	90.8 (74.6 to 110.2)	13137.1 (10587.7 to 16210)	-2.70 (-2.99 to -2.41)
Nauru	0.7 (0.5 to 0.8)	13640.6 (10699.2 to 17061.7)	0.7 (0.5 to 0.8)	13640.6 (10699.2 to 17061.7)	-1.24 (-1.84 to -0.63)
Nepal	2190.7 (1913 to 2490.1)	22562.6 (19470.7 to 26275.9)	2190.7 (1913 to 2490.1)	22562.6 (19470.7 to 26275.9)	-4.18 (-4.37 to -4.00)
Netherlands	59.4 (47.6 to 73.5)	885.1 (680.6 to 1124.5)	59.4 (47.6 to 73.5)	885.1 (680.6 to 1124.5)	-1.61 (-1.87 to -1.34)
New Zealand	14.7 (11.9 to 17.9)	919.8 (727.7 to 1146.2)	14.7 (11.9 to 17.9)	919.8 (727.7 to 1146.2)	-0.78 (-1.02 to -0.55)
Nicaragua	176.4 (147 to 209.6)	8746.4 (7147.9 to 10598.2)	176.4 (147 to 209.6)	8746.4 (7147.9 to 10598.2)	-6.05 (-6.52 to -5.58)
Niger	1795.4 (1606.7 to 2006.4)	45012.2 (39697.2 to 50974)	1795.4 (1606.7 to 2006.4)	45012.2 (39697.2 to 50974)	-1.03 (-1.12 to -0.94)
Nigeria	6003.4 (5068 to 6993.7)	13426.2 (11084.2 to 15872.7)	6003.4 (5068 to 6993.7)	13426.2 (11084.2 to 15872.7)	-2.94 (-3.75 to -2.13)
Niue	0.1 (0.1 to 0.1)	10445.6 (8245.1 to 12754.1)	0.1 (0.1 to 0.1)	10445.6 (8245.1 to 12754.1)	-3.00 (-3.13 to -2.87)
North Macedonia	221.4 (191.9 to 254.4)	22674.2 (19325.3 to 26510.2)	221.4 (191.9 to 254.4)	22674.2 (19325.3 to 26510.2)	-2.31 (-2.46 to -2.16)
Northern Mariana Islands	1 (0.8 to 1.2)	4881.6 (3792.2 to 6137.5)	1 (0.8 to 1.2)	4881.6 (3792.2 to 6137.5)	-0.76 (-0.97 to -0.55)
Norway	16.5 (13.5 to 20.4)	874.8 (683.2 to 1131.5)	16.5 (13.5 to 20.4)	874.8 (683.2 to 1131.5)	-2.24 (-2.51 to -1.96)
Oman	92.7 (75 to 113)	11404.6 (9006.8 to 14279)	92.7 (75 to 113)	11404.6 (9006.8 to 14279)	-6.75 (-7.27 to -6.23)
Pakistan	8821.6 (7568.2 to 10160.5)	16585.5 (14100.6 to 19304.4)	8821.6 (7568.2 to 10160.5)	16585.5 (14100.6 to 19304.4)	-4.99 (-5.15 to -4.82)

Continued on next page

Table S14 – continued from previous page

Country	1990		2019		1990–2019
	Incident cases No.×10^4[95% UI]	ASR per 10,0000 No.[95% UI]	Incident cases No.×10^4[95% UI]	ASR per 10,0000 No.[95% UI]	EAPC No.[95% CI]
Palau	0.6 (0.5 to 0.7)	8624.3 (6847.4 to 10721.5)	0.6 (0.5 to 0.7)	8624.3 (6847.4 to 10721.5)	-2.38 (-2.50 to -2.26)
Palestine	291.2 (250.3 to 336.2)	28438.4 (24003 to 33391.9)	291.2 (250.3 to 336.2)	28438.4 (24003 to 33391.9)	-6.01 (-6.37 to -5.65)
Panama	67.5 (56.7 to 81.2)	5800 (4761.2 to 7120.4)	67.5 (56.7 to 81.2)	5800 (4761.2 to 7120.4)	-2.55 (-2.73 to -2.37)
Papua New Guinea	288.7 (237.2 to 349)	14855.1 (12006.2 to 18136.7)	288.7 (237.2 to 349)	14855.1 (12006.2 to 18136.7)	-0.68 (-1.05 to -0.31)
Paraguay	270.6 (222.3 to 329.5)	14054.3 (11263.6 to 17420)	270.6 (222.3 to 329.5)	14054.3 (11263.6 to 17420)	-2.71 (-2.86 to -2.56)
Peru	1368.4 (1186.3 to 1582.5)	12715.6 (10865.8 to 14834.8)	1368.4 (1186.3 to 1582.5)	12715.6 (10865.8 to 14834.8)	-2.80 (-2.95 to -2.66)
Philippines	4962.2 (4247.2 to 5780.2)	16505.5 (13965.3 to 19481)	4962.2 (4247.2 to 5780.2)	16505.5 (13965.3 to 19481)	-1.90 (-2.27 to -1.52)
Poland	3206.3 (2744.3 to 3730.6)	16648.2 (13963.7 to 19686)	3206.3 (2744.3 to 3730.6)	16648.2 (13963.7 to 19686)	-2.39 (-2.43 to -2.35)
Portugal	121.9 (99.4 to 148.5)	2641.2 (2079.3 to 3339)	121.9 (99.4 to 148.5)	2641.2 (2079.3 to 3339)	-2.93 (-3.27 to -2.59)
Puerto Rico	53.7 (43.9 to 64.9)	3046.4 (2454.5 to 3752.5)	53.7 (43.9 to 64.9)	3046.4 (2454.5 to 3752.5)	-2.85 (-2.93 to -2.78)
Qatar	5.5 (4.4 to 6.8)	3736.8 (2928.4 to 4717.9)	5.5 (4.4 to 6.8)	3736.8 (2928.4 to 4717.9)	-4.55 (-4.77 to -4.32)
Republic of Korea	479.4 (383.4 to 581.4)	2365.5 (1857.3 to 2931.3)	479.4 (383.4 to 581.4)	2365.5 (1857.3 to 2931.3)	-3.82 (-4.04 to -3.61)
Republic of Moldova	89.4 (74.5 to 107)	3894.6 (3209.3 to 4725.2)	89.4 (74.5 to 107)	3894.6 (3209.3 to 4725.2)	-1.77 (-2.17 to -1.38)
Romania	2059.7 (1772.1 to 2384.5)	17662.2 (14937.5 to 20827.1)	2059.7 (1772.1 to 2384.5)	17662.2 (14937.5 to 20827.1)	-2.07 (-2.21 to -1.94)
Russian Federation	393.3 (329.6 to 471)	457.9 (378.3 to 559.4)	393.3 (329.6 to 471)	457.9 (378.3 to 559.4)	-2.21 (-2.28 to -2.13)
Rwanda	1042.8 (893.2 to 1201.5)	28193.1 (23687.2 to 32862.8)	1042.8 (893.2 to 1201.5)	28193.1 (23687.2 to 32862.8)	-2.92 (-3.31 to -2.53)
Saint Kitts and Nevis	1 (0.8 to 1.3)	5226.9 (4116.8 to 6609.6)	1 (0.8 to 1.3)	5226.9 (4116.8 to 6609.6)	-2.97 (-3.11 to -2.84)
Saint Lucia	5 (4.1 to 6.1)	7364.9 (5906.4 to 9189.9)	5 (4.1 to 6.1)	7364.9 (5906.4 to 9189.9)	-2.59 (-2.78 to -2.41)
Saint Vincent and the Grenadines	5.6 (4.6 to 6.7)	10446.4 (8353.6 to 12817.1)	5.6 (4.6 to 6.7)	10446.4 (8353.6 to 12817.1)	-3.09 (-3.24 to -2.94)
Samoa	12 (9.8 to 14.5)	16631.8 (13248.1 to 20246.8)	12 (9.8 to 14.5)	16631.8 (13248.1 to 20246.8)	-0.68 (-0.85 to -0.51)
San Marino	0.1 (0.1 to 0.1)	813.6 (642 to 1025.4)	0.1 (0.1 to 0.1)	813.6 (642 to 1025.4)	-1.62 (-1.94 to -1.29)
Sao Tome and Principe	15.4 (13 to 18.1)	26286.2 (21790.1 to 31114.3)	15.4 (13 to 18.1)	26286.2 (21790.1 to 31114.3)	-4.29 (-4.48 to -4.11)
Saudi Arabia	279 (224.5 to 353.2)	3833.3 (3005.3 to 4923.7)	279 (224.5 to 353.2)	3833.3 (3005.3 to 4923.7)	-8.08 (-8.94 to -7.21)
Senegal	992 (843.9 to 1155.7)	26235.5 (21832 to 31278)	992 (843.9 to 1155.7)	26235.5 (21832 to 31278)	-3.72 (-4.02 to -3.41)
Serbia	1265.6 (1120.6 to 1429.5)	26890.9 (23579.2 to 30891.5)	1265.6 (1120.6 to 1429.5)	26890.9 (23579.2 to 30891.5)	-2.31 (-2.48 to -2.15)
Seychelles	2.2 (1.8 to 2.7)	6784.1 (5361.3 to 8439.7)	2.2 (1.8 to 2.7)	6784.1 (5361.3 to 8439.7)	-4.60 (-5.13 to -4.06)
Sierra Leone	556.4 (472.9 to 633.7)	30819.3 (25653.5 to 35577)	556.4 (472.9 to 633.7)	30819.3 (25653.5 to 35577)	-2.26 (-2.50 to -2.02)

Continued on next page

Table S14 – continued from previous page

Country	1990		2019		1990–2019
	Incident cases No.$\times 10^4$[95% UI]	ASR per 10,0000 No.[95% UI]	Incident cases No.$\times 10^4$[95% UI]	ASR per 10,0000 No.[95% UI]	EAPC No.[95% CI]
Singapore	29.1 (23.5 to 35.9)	2087.5 (1645.2 to 2633)	29.1 (23.5 to 35.9)	2087.5 (1645.2 to 2633)	-3.28 (-3.39 to -3.17)
Slovakia	415.2 (355.6 to 484.8)	15627.7 (13159.3 to 18581.9)	415.2 (355.6 to 484.8)	15627.7 (13159.3 to 18581.9)	-2.10 (-2.18 to -2.02)
Slovenia	106.8 (90.4 to 125.4)	10781.5 (8937.8 to 12824.9)	106.8 (90.4 to 125.4)	10781.5 (8937.8 to 12824.9)	-1.52 (-1.58 to -1.46)
Solomon Islands	56.2 (48.4 to 64.6)	35501 (29972.7 to 41420.7)	56.2 (48.4 to 64.6)	35501 (29972.7 to 41420.7)	-1.34 (-1.55 to -1.14)
Somalia	2024.6 (1867.9 to 2190.6)	58377.3 (53054.1 to 63870.7)	2024.6 (1867.9 to 2190.6)	58377.3 (53054.1 to 63870.7)	-0.30 (-0.35 to -0.26)
South Africa	1969.4 (1659.8 to 2335.4)	11369.4 (9427.2 to 13751)	1969.4 (1659.8 to 2335.4)	11369.4 (9427.2 to 13751)	-3.61 (-3.71 to -3.51)
South Sudan	845.2 (722.5 to 979.3)	30398.5 (25448.7 to 35628.1)	845.2 (722.5 to 979.3)	30398.5 (25448.7 to 35628.1)	-2.11 (-2.23 to -1.99)
Spain	350.3 (288.4 to 426.6)	2000.6 (1590.9 to 2475.6)	350.3 (288.4 to 426.6)	2000.6 (1590.9 to 2475.6)	-3.05 (-3.53 to -2.55)
Sri Lanka	1283.6 (1086.1 to 1493.1)	15599.9 (12993.9 to 18434.9)	1283.6 (1086.1 to 1493.1)	15599.9 (12993.9 to 18434.9)	-4.84 (-5.08 to -4.59)
Sudan	2880.5 (2454.5 to 3334.3)	28553.8 (23881.5 to 33475.4)	2880.5 (2454.5 to 3334.3)	28553.8 (23881.5 to 33475.4)	-4.27 (-4.57 to -3.96)
Suriname	16.6 (13.7 to 20.1)	8967.6 (7225.6 to 11001.3)	16.6 (13.7 to 20.1)	8967.6 (7225.6 to 11001.3)	-2.75 (-2.89 to -2.61)
Sweden	40.5 (33.3 to 49.8)	1065.2 (836.2 to 1361.9)	40.5 (33.3 to 49.8)	1065.2 (836.2 to 1361.9)	-2.05 (-2.40 to -1.69)
Switzerland	17.1 (13.8 to 20.7)	558.8 (436.5 to 701.3)	17.1 (13.8 to 20.7)	558.8 (436.5 to 701.3)	-1.36 (-1.56 to -1.15)
Syrian Arab Republic	757.6 (622 to 913.6)	11981.1 (9585.1 to 14780.8)	757.6 (622 to 913.6)	11981.1 (9585.1 to 14780.8)	-4.44 (-4.65 to -4.23)
Taiwan (Province of China)	232.1 (185.1 to 289.2)	2579 (2013 to 3252.9)	232.1 (185.1 to 289.2)	2579 (2013 to 3252.9)	-4.66 (-4.81 to -4.51)
Tajikistan	289.5 (239 to 345.9)	10662.2 (8539.7 to 13111.1)	289.5 (239 to 345.9)	10662.2 (8539.7 to 13111.1)	-1.14 (-1.64 to -0.64)
Thailand	3193.8 (2726.4 to 3755.4)	12140.5 (10247.9 to 14306)	3193.8 (2726.4 to 3755.4)	12140.5 (10247.9 to 14306)	-6.51 (-7.02 to -5.99)
Timor-Leste	120.4 (105.3 to 136)	32476.6 (27829.8 to 37095)	120.4 (105.3 to 136)	32476.6 (27829.8 to 37095)	-5.78 (-6.20 to -5.35)
Togo	462.3 (386.5 to 547.6)	25317.3 (20634.5 to 30493.2)	462.3 (386.5 to 547.6)	25317.3 (20634.5 to 30493.2)	-2.40 (-2.61 to -2.18)
Tokelau	0.1 (0.1 to 0.2)	18737 (15196.9 to 22717.6)	0.1 (0.1 to 0.2)	18737 (15196.9 to 22717.6)	-3.07 (-3.23 to -2.92)
Tonga	7.6 (6.3 to 9.1)	16972.2 (13805.5 to 20708.8)	7.6 (6.3 to 9.1)	16972.2 (13805.5 to 20708.8)	-1.85 (-2.05 to -1.66)
Trinidad and Tobago	27 (21.9 to 33)	4652.3 (3706.8 to 5837.1)	27 (21.9 to 33)	4652.3 (3706.8 to 5837.1)	-3.02 (-3.20 to -2.83)
Tunisia	293.3 (239.4 to 354.1)	7028 (5622 to 8623.8)	293.3 (239.4 to 354.1)	7028 (5622 to 8623.8)	-4.93 (-5.22 to -4.64)
Turkey	4359.6 (3824.2 to 4905.9)	14717.2 (12770.8 to 16631.3)	4359.6 (3824.2 to 4905.9)	14717.2 (12770.8 to 16631.3)	-4.73 (-4.90 to -4.57)
Turkmenistan	86.4 (70.4 to 107.2)	4591.9 (3653.3 to 5794.8)	86.4 (70.4 to 107.2)	4591.9 (3653.3 to 5794.8)	-2.90 (-3.20 to -2.60)
Tuvalu	0.9 (0.8 to 1.1)	21186.2 (17718.9 to 25081.5)	0.9 (0.8 to 1.1)	21186.2 (17718.9 to 25081.5)	-2.03 (-2.18 to -1.89)
Uganda	2413.1 (2115.7 to 2744.7)	27169.8 (23256.2 to 31290.8)	2413.1 (2115.7 to 2744.7)	27169.8 (23256.2 to 31290.8)	-3.19 (-3.36 to -3.03)
Ukraine	680.3 (573.1 to 809.1)	2490 (2047.4 to 3015.5)	680.3 (573.1 to 809.1)	2490 (2047.4 to 3015.5)	-1.20 (-1.43 to -0.97)
United Arab Emirates	27.2 (22.3 to 33)	4084.6 (3326.3 to 5009.6)	27.2 (22.3 to 33)	4084.6 (3326.3 to 5009.6)	-4.22 (-4.57 to -3.87)

Continued on next page

Table S14 – continued from previous page

Country	1990		2019		1990–2019
	Incident cases No.$\times10^4$[95% UI]	ASR per 10,0000 No.[95% UI]	Incident cases No.$\times10^4$[95% UI]	ASR per 10,0000 No.[95% UI]	EAPC No.[95% CI]
United Kingdom	314 (256.1 to 386.6)	1209.6 (953.9 to 1537)	314 (256.1 to 386.6)	1209.6 (953.9 to 1537)	-1.98 (-2.28 to -1.69)
United Republic of Tanzania	3339.5 (2875.7 to 3807.1)	24613.3 (20887 to 28428)	3339.5 (2875.7 to 3807.1)	24613.3 (20887 to 28428)	-1.82 (-2.08 to -1.56)
United States of America	1215.9 (1010.2 to 1444.3)	1058 (865.3 to 1287.6)	1215.9 (1010.2 to 1444.3)	1058 (865.3 to 1287.6)	-1.66 (-1.79 to -1.53)
United States Virgin Islands	1.8 (1.5 to 2.1)	3388.5 (2752.1 to 4165.5)	1.8 (1.5 to 2.1)	3388.5 (2752.1 to 4165.5)	-3.59 (-3.78 to -3.39)
Uruguay	96.5 (78.5 to 116.7)	6951.4 (5547.3 to 8658.6)	96.5 (78.5 to 116.7)	6951.4 (5547.3 to 8658.6)	-1.44 (-1.69 to -1.20)
Uzbekistan	673.6 (540.8 to 837)	6354 (5011 to 8074.3)	673.6 (540.8 to 837)	6354 (5011 to 8074.3)	-2.61 (-2.83 to -2.38)
Vanuatu	21.9 (18.6 to 25.5)	31047.9 (26005.9 to 36485.9)	21.9 (18.6 to 25.5)	31047.9 (26005.9 to 36485.9)	-1.46 (-1.60 to -1.33)
Venezuela (Bolivarian Republic of)	670.7 (564.9 to 792.3)	7133.2 (5914.1 to 8618.7)	670.7 (564.9 to 792.3)	7133.2 (5914.1 to 8618.7)	-1.08 (-1.59 to -0.57)
Viet Nam	2829.2 (2299.1 to 3417.1)	9210 (7360.8 to 11401.7)	2829.2 (2299.1 to 3417.1)	9210 (7360.8 to 11401.7)	-5.34 (-5.57 to -5.11)
Yemen	2461.7 (2161.7 to 2763.9)	36291 (31098.3 to 41353.1)	2461.7 (2161.7 to 2763.9)	36291 (31098.3 to 41353.1)	-4.61 (-4.95 to -4.27)
Zambia	1140.7 (991.3 to 1305.7)	28165.9 (24072.9 to 32600.5)	1140.7 (991.3 to 1305.7)	28165.9 (24072.9 to 32600.5)	-3.66 (-4.10 to -3.21)
Zimbabwe	1318.2 (1118.2 to 1529.9)	25261.1 (21084.8 to 29929.7)	1318.2 (1118.2 to 1529.9)	25261.1 (21084.8 to 29929.7)	-0.14 (-0.50 to 0.22)

Abbreviation: ASR, age standardized rate; CI, confidence interval; EAPC, estimated annual percentage change; UI, uncertainty interval.

Table S15: The change of vitamin A deficiency cases between 1990 and 2019 at national level for male.

Country	1990		2019		1990–2019
	Incident cases No.$\times10^4$[95% UI]	ASR per 10,0000 No.[95% UI]	Incident cases No.$\times10^4$[95% UI]	ASR per 10,0000 No.[95% UI]	EAPC No.[95% CI]
Afghanistan	2795.9 (2450.7 to 3161.7)	49428.1 (42176.5 to 57025.7)	2795.9 (2450.7 to 3161.7)	49428.1 (42176.5 to 57025.7)	-1.68 (-2.12 to -1.23)
Albania	408.6 (329.1 to 494.3)	25351.7 (20067.7 to 31135)	408.6 (329.1 to 494.3)	25351.7 (20067.7 to 31135)	-4.47 (-4.78 to -4.16)
Algeria	1710.7 (1307.5 to 2172)	13353.2 (9906.5 to 17328)	1710.7 (1307.5 to 2172)	13353.2 (9906.5 to 17328)	-4.84 (-5.01 to -4.68)
American Samoa	3.2 (2.4 to 4.2)	13700.6 (10036.2 to 18067.3)	3.2 (2.4 to 4.2)	13700.6 (10036.2 to 18067.3)	-1.75 (-2.03 to -1.47)
Andorra	0.2 (0.1 to 0.2)	608.8 (429.4 to 828.2)	0.2 (0.1 to 0.2)	608.8 (429.4 to 828.2)	-1.80 (-1.99 to -1.62)
Angola	3061 (2721.5 to 3382.4)	58944.2 (51362.7 to 66260.3)	3061 (2721.5 to 3382.4)	58944.2 (51362.7 to 66260.3)	-4.17 (-4.53 to -3.81)

Continued on next page

Table S15 – continued from previous page

Country	1990		2019		1990–2019
	Incident cases No.×10^4[95% UI]	ASR per 10,0000 No.[95% UI]	Incident cases No.×10^4[95% UI]	ASR per 10,0000 No.[95% UI]	EAPC No.[95% CI]
Antigua and Barbuda	1.2 (0.9 to 1.5)	4223.7 (2970.2 to 5636.1)	1.2 (0.9 to 1.5)	4223.7 (2970.2 to 5636.1)	-3.24 (-3.39 to -3.09)
Argentina	2192.8 (1775.7 to 2657.3)	14210.8 (11294 to 17570.7)	2192.8 (1775.7 to 2657.3)	14210.8 (11294 to 17570.7)	-1.23 (-1.48 to -0.99)
Armenia	17.1 (12.9 to 22.1)	1012.9 (749.7 to 1352.4)	17.1 (12.9 to 22.1)	1012.9 (749.7 to 1352.4)	-3.53 (-4.00 to -3.06)
Australia	9.9 (7.6 to 12.5)	98 (74.2 to 126.5)	9.9 (7.6 to 12.5)	98 (74.2 to 126.5)	-1.56 (-1.90 to -1.22)
Austria	44.8 (33.8 to 59.4)	1326.3 (957.3 to 1821.5)	44.8 (33.8 to 59.4)	1326.3 (957.3 to 1821.5)	-2.99 (-3.27 to -2.71)
Azerbaijan	183.1 (135.2 to 242.8)	5212.7 (3714.3 to 7108.9)	183.1 (135.2 to 242.8)	5212.7 (3714.3 to 7108.9)	-2.60 (-3.37 to -1.84)
Bahamas	3.1 (2.3 to 4.1)	2539.9 (1815.8 to 3476.9)	3.1 (2.3 to 4.1)	2539.9 (1815.8 to 3476.9)	-3.13 (-3.38 to -2.88)
Bahrain	14.7 (11.1 to 19.4)	5026.4 (3712.5 to 6762.3)	14.7 (11.1 to 19.4)	5026.4 (3712.5 to 6762.3)	-4.06 (-4.22 to -3.89)
Bangladesh	11174 (9101 to 13453.6)	19991.8 (16217.5 to 24231.5)	11174 (9101 to 13453.6)	19991.8 (16217.5 to 24231.5)	-4.94 (-5.28 to -4.59)
Barbados	3.9 (2.9 to 5)	3400.9 (2477.7 to 4511.1)	3.9 (2.9 to 5)	3400.9 (2477.7 to 4511.1)	-2.11 (-2.21 to -2.02)
Belarus	143.7 (109.8 to 186)	3100.2 (2298.4 to 4091.3)	143.7 (109.8 to 186)	3100.2 (2298.4 to 4091.3)	-4.66 (-4.87 to -4.46)
Belgium	56 (42.3 to 76)	1273.5 (917 to 1801.2)	56 (42.3 to 76)	1273.5 (917 to 1801.2)	-2.94 (-3.21 to -2.67)
Belize	12.9 (9.8 to 16.2)	13819.4 (10312.9 to 17969.7)	12.9 (9.8 to 16.2)	13819.4 (10312.9 to 17969.7)	-3.58 (-3.76 to -3.39)
Benin	1698.7 (1558.6 to 1814.8)	73065.1 (66597 to 78711)	1698.7 (1558.6 to 1814.8)	73065.1 (66597 to 78711)	-2.23 (-2.30 to -2.15)
Bermuda	0.6 (0.5 to 0.8)	2244.7 (1653.2 to 3000.7)	0.6 (0.5 to 0.8)	2244.7 (1653.2 to 3000.7)	-3.02 (-3.21 to -2.84)
Bhutan	93.5 (75.4 to 113.2)	28658.4 (22272.5 to 35262.6)	93.5 (75.4 to 113.2)	28658.4 (22272.5 to 35262.6)	-5.47 (-5.53 to -5.42)
Bolivia (Plurinational State of)	414 (309 to 534)	13823.8 (10173.4 to 18320.3)	414 (309 to 534)	13823.8 (10173.4 to 18320.3)	-1.57 (-1.88 to -1.27)
Bosnia and Herzegovina	390.9 (308.8 to 481.5)	18736.1 (14458.6 to 23338.4)	390.9 (308.8 to 481.5)	18736.1 (14458.6 to 23338.4)	-4.79 (-5.12 to -4.47)
Botswana	187.9 (152 to 227.1)	31389.1 (24588.6 to 38477.9)	187.9 (152 to 227.1)	31389.1 (24588.6 to 38477.9)	-3.71 (-3.85 to -3.57)
Brazil	20195.8 (17187.8 to 23771.5)	27812.5 (23377.4 to 33081.9)	20195.8 (17187.8 to 23771.5)	27812.5 (23377.4 to 33081.9)	-3.28 (-3.41 to -3.14)
Brunei Darussalam	3.2 (2.2 to 4.5)	2458.1 (1650.2 to 3559.4)	3.2 (2.2 to 4.5)	2458.1 (1650.2 to 3559.4)	-3.11 (-3.18 to -3.04)
Bulgaria	408.5 (319.7 to 524.1)	11013.6 (8355.3 to 14458.3)	408.5 (319.7 to 524.1)	11013.6 (8355.3 to 14458.3)	-2.26 (-2.57 to -1.94)
Burkina Faso	3611.7 (3373.1 to 3801.8)	79343.9 (73273.5 to 83855.4)	3611.7 (3373.1 to 3801.8)	79343.9 (73273.5 to 83855.4)	-3.12 (-3.25 to -2.99)
Burundi	1173.4 (1001 to 1351.8)	42238.8 (35493.1 to 49754.6)	1173.4 (1001 to 1351.8)	42238.8 (35493.1 to 49754.6)	-2.13 (-2.34 to -1.91)
Cabo Verde	72.6 (61.3 to 84)	45179.3 (37240.6 to 53137.9)	72.6 (61.3 to 84)	45179.3 (37240.6 to 53137.9)	-6.72 (-7.06 to -6.38)
Cambodia	2015.4 (1697.8 to 2341.1)	42166.5 (34937.5 to 49784)	2015.4 (1697.8 to 2341.1)	42166.5 (34937.5 to 49784)	-5.81 (-5.94 to -5.69)
Cameroon	4727.6 (4620.8 to 4817.3)	92312.4 (89901.8 to 94293.4)	4727.6 (4620.8 to 4817.3)	92312.4 (89901.8 to 94293.4)	-2.74 (-2.94 to -2.53)
Canada	184.4 (130.6 to 255.2)	1638.2 (1107 to 2305.5)	184.4 (130.6 to 255.2)	1638.2 (1107 to 2305.5)	-3.18 (-3.43 to -2.93)
Central African Republic	860.8 (777.6 to 941.1)	64335.5 (57373.2 to 71002.8)	860.8 (777.6 to 941.1)	64335.5 (57373.2 to 71002.8)	-1.03 (-1.09 to -0.97)

Continued on next page

Table S15 – continued from previous page

Country	1990		2019		1990–2019
	Incident cases No.×10^4[95% UI]	ASR per 10,0000 No.[95% UI]	Incident cases No.×10^4[95% UI]	ASR per 10,0000 No.[95% UI]	EAPC No.[95% CI]
Chad	2327.1 (2182.5 to 2439)	80950.4 (75176.5 to 85135.6)	2327.1 (2182.5 to 2439)	80950.4 (75176.5 to 85135.6)	-1.85 (-1.90 to -1.79)
Chile	578.2 (430.8 to 760.6)	9808.2 (7177.9 to 13068.4)	578.2 (430.8 to 760.6)	9808.2 (7177.9 to 13068.4)	-2.28 (-2.51 to -2.04)
China	83311.9 (65672.2 to 103746.9)	14803.4 (11480.2 to 18541)	83311.9 (65672.2 to 103746.9)	14803.4 (11480.2 to 18541)	-6.31 (-6.43 to -6.18)
Colombia	1495.8 (1158.2 to 1868.5)	9377.4 (7001.9 to 12055)	1495.8 (1158.2 to 1868.5)	9377.4 (7001.9 to 12055)	-4.24 (-4.34 to -4.13)
Comoros	115.8 (100.5 to 133.1)	49250.3 (42040.6 to 57858.3)	115.8 (100.5 to 133.1)	49250.3 (42040.6 to 57858.3)	-2.52 (-2.69 to -2.35)
Congo	773.7 (698.1 to 843.1)	65899.7 (58816.4 to 72361.4)	773.7 (698.1 to 843.1)	65899.7 (58816.4 to 72361.4)	-1.72 (-2.00 to -1.44)
Cook Islands	1 (0.8 to 1.3)	11459.6 (8369.5 to 15193.4)	1 (0.8 to 1.3)	11459.6 (8369.5 to 15193.4)	-2.79 (-3.00 to -2.58)
Costa Rica	123.7 (95.9 to 155.5)	8296.4 (6262.4 to 10721.7)	123.7 (95.9 to 155.5)	8296.4 (6262.4 to 10721.7)	-3.52 (-3.63 to -3.40)
Croatia	195.9 (152.5 to 248.3)	9308.6 (7066.1 to 12071.1)	195.9 (152.5 to 248.3)	9308.6 (7066.1 to 12071.1)	-2.46 (-2.69 to -2.22)
Cuba	222.3 (167.3 to 282.4)	4252.9 (3122.7 to 5567.7)	222.3 (167.3 to 282.4)	4252.9 (3122.7 to 5567.7)	-2.34 (-2.58 to -2.09)
Cyprus	10 (7.3 to 13.5)	2785.4 (1958.9 to 3916.8)	10 (7.3 to 13.5)	2785.4 (1958.9 to 3916.8)	-5.01 (-5.61 to -4.41)
Czechia	324.5 (248.7 to 417.8)	7385.7 (5539.1 to 9701.7)	324.5 (248.7 to 417.8)	7385.7 (5539.1 to 9701.7)	-2.38 (-2.58 to -2.19)
Ivoirian	2618.2 (2271.3 to 2972.1)	43231.1 (36934.1 to 49626.3)	2618.2 (2271.3 to 2972.1)	43231.1 (36934.1 to 49626.3)	-3.51 (-3.64 to -3.38)
Democratic People's Republic of Korea	2120.6 (1671.9 to 2573.5)	23015.7 (17993.6 to 28349.4)	2120.6 (1671.9 to 2573.5)	23015.7 (17993.6 to 28349.4)	-3.65 (-3.87 to -3.42)
Democratic Republic of the Congo	10226.5 (8891.2 to 11546.6)	54104.6 (46316.4 to 62192.1)	10226.5 (8891.2 to 11546.6)	54104.6 (46316.4 to 62192.1)	-0.70 (-1.21 to -0.19)
Denmark	19.4 (14.5 to 25.7)	844.9 (599.1 to 1186.9)	19.4 (14.5 to 25.7)	844.9 (599.1 to 1186.9)	-2.33 (-2.55 to -2.11)
Djibouti	142 (122.6 to 159.9)	53549.1 (45576.9 to 61173.6)	142 (122.6 to 159.9)	53549.1 (45576.9 to 61173.6)	-3.72 (-3.95 to -3.49)
Dominica	1.9 (1.4 to 2.5)	5486 (3956.4 to 7433.9)	1.9 (1.4 to 2.5)	5486 (3956.4 to 7433.9)	-4.04 (-4.37 to -3.70)
Dominican Republic	559.5 (457.3 to 683)	16043.9 (12793.9 to 20017.9)	559.5 (457.3 to 683)	16043.9 (12793.9 to 20017.9)	-4.85 (-5.22 to -4.48)
Ecuador	387.2 (291.5 to 510.7)	8480.1 (6197.9 to 11500.9)	387.2 (291.5 to 510.7)	8480.1 (6197.9 to 11500.9)	-3.16 (-3.46 to -2.85)
Egypt	2418.4 (1923.3 to 3069.8)	8543.1 (6503 to 11119.3)	2418.4 (1923.3 to 3069.8)	8543.1 (6503 to 11119.3)	-4.33 (-4.71 to -3.96)
El Salvador	621.5 (502.2 to 753.6)	24444.1 (19286.6 to 30437)	621.5 (502.2 to 753.6)	24444.1 (19286.6 to 30437)	-5.04 (-5.39 to -4.70)
Equatorial Guinea	154.1 (142.4 to 163.9)	75282.2 (68989.8 to 80605.1)	154.1 (142.4 to 163.9)	75282.2 (68989.8 to 80605.1)	-10.09 (-10.62 to -9.56)
Eritrea	942.6 (840.7 to 1034.3)	61974.5 (54259.8 to 68996.3)	942.6 (840.7 to 1034.3)	61974.5 (54259.8 to 68996.3)	-2.86 (-2.96 to -2.75)
Estonia	10.9 (8.3 to 14)	1577.6 (1147.8 to 2100.9)	10.9 (8.3 to 14)	1577.6 (1147.8 to 2100.9)	-4.58 (-4.79 to -4.37)
Eswatini	121.3 (95.6 to 145.8)	32637.4 (24976.2 to 39928.7)	121.3 (95.6 to 145.8)	32637.4 (24976.2 to 39928.7)	-4.11 (-4.22 to -4.00)
Ethiopia	16309.6 (14813.1 to 17954.3)	61911 (55354.7 to 68899.2)	16309.6 (14813.1 to 17954.3)	61911 (55354.7 to 68899.2)	-2.82 (-3.19 to -2.45)
Fiji	90.8 (71.1 to 112.7)	25165.6 (19352.8 to 31721.9)	90.8 (71.1 to 112.7)	25165.6 (19352.8 to 31721.9)	-2.35 (-2.54 to -2.16)

Continued on next page

Table S15 – continued from previous page

Country	1990		2019		1990–2019
	Incident cases No.×10^4[95% UI]	ASR per 10,0000 No.[95% UI]	Incident cases No.×10^4[95% UI]	ASR per 10,0000 No.[95% UI]	EAPC No.[95% CI]
Finland	25.1 (18.6 to 34.2)	1143.7 (805.1 to 1600.5)	25.1 (18.6 to 34.2)	1143.7 (805.1 to 1600.5)	-2.84 (-3.05 to -2.63)
France	70.9 (54.9 to 93.4)	268.1 (198 to 370.5)	70.9 (54.9 to 93.4)	268.1 (198 to 370.5)	-1.08 (-1.26 to -0.91)
Gabon	142.2 (116 to 169.9)	29691.2 (23627.7 to 36038.1)	142.2 (116 to 169.9)	29691.2 (23627.7 to 36038.1)	-5.24 (-5.38 to -5.09)
Gambia	346.6 (318.2 to 372.5)	70475.2 (63565.6 to 76074.4)	346.6 (318.2 to 372.5)	70475.2 (63565.6 to 76074.4)	-3.03 (-3.09 to -2.97)
Georgia	74.5 (57.5 to 96.7)	2944 (2199.4 to 3933)	74.5 (57.5 to 96.7)	2944 (2199.4 to 3933)	-0.28 (-1.00 to 0.45)
Germany	204.1 (160.3 to 257.2)	566.6 (426.6 to 731.9)	204.1 (160.3 to 257.2)	566.6 (426.6 to 731.9)	-1.64 (-1.72 to -1.56)
Ghana	4698.6 (4228.8 to 5128.5)	63968.9 (57140.6 to 70407.4)	4698.6 (4228.8 to 5128.5)	63968.9 (57140.6 to 70407.4)	-3.61 (-3.75 to -3.46)
Greece	88.1 (65.1 to 121.1)	1928.8 (1361.3 to 2825.2)	88.1 (65.1 to 121.1)	1928.8 (1361.3 to 2825.2)	-2.91 (-3.35 to -2.47)
Greenland	0.2 (0.1 to 0.3)	664.8 (457.8 to 1020.8)	0.2 (0.1 to 0.3)	664.8 (457.8 to 1020.8)	-1.41 (-2.04 to -0.77)
Grenada	5.3 (4 to 6.9)	13298.3 (9644.7 to 17719.1)	5.3 (4 to 6.9)	13298.3 (9644.7 to 17719.1)	-4.06 (-4.46 to -3.66)
Guam	4.6 (3.4 to 6.2)	6847.9 (5001.9 to 9297.4)	4.6 (3.4 to 6.2)	6847.9 (5001.9 to 9297.4)	-2.57 (-2.76 to -2.39)
Guatemala	853.1 (704.5 to 1032.1)	21144.1 (16824.4 to 26093.8)	853.1 (704.5 to 1032.1)	21144.1 (16824.4 to 26093.8)	-4.21 (-4.41 to -4.00)
Guinea	2141.6 (1968.3 to 2292.1)	72519.1 (65878.6 to 78189.2)	2141.6 (1968.3 to 2292.1)	72519.1 (65878.6 to 78189.2)	-2.56 (-2.71 to -2.42)
Guinea-Bissau	383.6 (361.2 to 402.8)	79800.1 (74726.9 to 84102.2)	383.6 (361.2 to 402.8)	79800.1 (74726.9 to 84102.2)	-2.50 (-2.56 to -2.43)
Guyana	56.4 (43.2 to 72.6)	15226.6 (11536.2 to 20004.3)	56.4 (43.2 to 72.6)	15226.6 (11536.2 to 20004.3)	-3.69 (-3.85 to -3.52)
Haiti	910.6 (746.1 to 1089.5)	29824.4 (23840.6 to 36411.1)	910.6 (746.1 to 1089.5)	29824.4 (23840.6 to 36411.1)	-2.64 (-2.70 to -2.57)
Honduras	378.8 (302.4 to 473.6)	15889.2 (12210.3 to 20441.1)	378.8 (302.4 to 473.6)	15889.2 (12210.3 to 20441.1)	-3.36 (-3.59 to -3.12)
Hungary	369.4 (284.4 to 465.6)	8461.4 (6345.6 to 11021.9)	369.4 (284.4 to 465.6)	8461.4 (6345.6 to 11021.9)	-2.43 (-2.54 to -2.32)
Iceland	1.5 (1.1 to 2)	1264.9 (854.6 to 1791.2)	1.5 (1.1 to 2)	1264.9 (854.6 to 1791.2)	-3.36 (-3.56 to -3.15)
India	166330.8 (142277.2 to 190990.2)	37068.6 (31099.1 to 43379.8)	166330.8 (142277.2 to 190990.2)	37068.6 (31099.1 to 43379.8)	-4.60 (-4.91 to -4.30)
Indonesia	27459.6 (22496.3 to 32447.3)	31606.1 (25449.7 to 37730.5)	27459.6 (22496.3 to 32447.3)	31606.1 (25449.7 to 37730.5)	-5.84 (-5.98 to -5.71)
Iran (Islamic Republic of)	4145.9 (3384 to 5057)	13937 (11066.2 to 17510.5)	4145.9 (3384 to 5057)	13937 (11066.2 to 17510.5)	-7.37 (-7.91 to -6.83)
Iraq	1495.1 (1178.3 to 1872.9)	16576.1 (12383 to 21363.5)	1495.1 (1178.3 to 1872.9)	16576.1 (12383 to 21363.5)	-5.31 (-5.69 to -4.92)
Ireland	21.1 (15.4 to 29.4)	1295 (899.3 to 1875.4)	21.1 (15.4 to 29.4)	1295 (899.3 to 1875.4)	-3.24 (-3.63 to -2.85)
Israel	244.6 (185.4 to 314.9)	11589.8 (8529.1 to 15358.9)	244.6 (185.4 to 314.9)	11589.8 (8529.1 to 15358.9)	-2.89 (-3.27 to -2.51)
Italy	1007.1 (826.2 to 1225.3)	3280.9 (2584.8 to 4125.3)	1007.1 (826.2 to 1225.3)	3280.9 (2584.8 to 4125.3)	-2.36 (-2.77 to -1.94)
Jamaica	70.4 (53.2 to 93.6)	6375 (4696.6 to 8741.8)	70.4 (53.2 to 93.6)	6375 (4696.6 to 8741.8)	-3.72 (-3.90 to -3.54)
Japan	470.2 (342.5 to 650.9)	884.8 (620.5 to 1271.4)	470.2 (342.5 to 650.9)	884.8 (620.5 to 1271.4)	-1.01 (-1.16 to -0.86)
Jordan	331.6 (263.6 to 409.1)	17119.2 (13376.5 to 21735.4)	331.6 (263.6 to 409.1)	17119.2 (13376.5 to 21735.4)	-3.97 (-4.41 to -3.52)
Kazakhstan	1870.6 (1525.3 to 2239.4)	23967.8 (19236.2 to 29246.3)	1870.6 (1525.3 to 2239.4)	23967.8 (19236.2 to 29246.3)	-2.69 (-2.80 to -2.58)
Kenya	6623.5 (5895.5 to 7333.2)	57553.9 (50385.2 to 64320.6)	6623.5 (5895.5 to 7333.2)	57553.9 (50385.2 to 64320.6)	-2.13 (-2.29 to -1.97)
Kiribati	13.4 (11.3 to 15.5)	38226.5 (31927.7 to 45208.2)	13.4 (11.3 to 15.5)	38226.5 (31927.7 to 45208.2)	-0.02 (-0.21 to 0.17)
Kuwait	21.8 (16.3 to 29.2)	2178 (1589.4 to 2932.2)	21.8 (16.3 to 29.2)	2178 (1589.4 to 2932.2)	-4.54 (-4.80 to -4.27)
Kyrgyzstan	151 (112.4 to 198.8)	7048.5 (5119.9 to 9502)	151 (112.4 to 198.8)	7048.5 (5119.9 to 9502)	-0.79 (-1.09 to -0.50)

Continued on next page

Country	1990		2019		1990–2019
	Incident cases No.×10^4[95% UI]	ASR per 10,0000 No.[95% UI]	Incident cases No.×10^4[95% UI]	ASR per 10,0000 No.[95% UI]	EAPC No.[95% CI]
Lao People's Democratic Republic	847.2 (738.8 to 948.8)	42642.3 (36674.9 to 48220.3)	847.2 (738.8 to 948.8)	42642.3 (36674.9 to 48220.3)	-4.24 (-4.52 to -3.95)
Latvia	16.3 (12.3 to 21.2)	1407.7 (1032.7 to 1881.1)	16.3 (12.3 to 21.2)	1407.7 (1032.7 to 1881.1)	-3.99 (-4.19 to -3.79)
Lebanon	123.3 (95 to 160.1)	7498 (5570 to 10083.2)	123.3 (95 to 160.1)	7498 (5570 to 10083.2)	-5.06 (-5.33 to -4.79)
Lesotho	393.9 (337.5 to 446.1)	46959.8 (39672 to 53678.2)	393.9 (337.5 to 446.1)	46959.8 (39672 to 53678.2)	-3.07 (-3.20 to -2.94)
Liberia	551.7 (489.5 to 610.2)	58162.6 (50628.9 to 65209.7)	551.7 (489.5 to 610.2)	58162.6 (50628.9 to 65209.7)	-3.83 (-4.06 to -3.61)
Libya	186.6 (139.4 to 239)	8448.3 (6181 to 11022.4)	186.6 (139.4 to 239)	8448.3 (6181 to 11022.4)	-4.78 (-5.35 to -4.20)
Lithuania	24.1 (18.1 to 31.2)	1476.7 (1080.1 to 1971)	24.1 (18.1 to 31.2)	1476.7 (1080.1 to 1971)	-4.65 (-4.80 to -4.50)
Luxembourg	1.3 (0.9 to 1.7)	756.8 (530.9 to 1062.8)	1.3 (0.9 to 1.7)	756.8 (530.9 to 1062.8)	-2.31 (-2.52 to -2.09)
Madagascar	2821.8 (2428.5 to 3224.2)	46406.9 (39048 to 53664.2)	2821.8 (2428.5 to 3224.2)	46406.9 (39048 to 53664.2)	-2.10 (-2.30 to -1.89)
Malawi	2686.8 (2424.4 to 2948.6)	56664.7 (50247.6 to 63073.4)	2686.8 (2424.4 to 2948.6)	56664.7 (50247.6 to 63073.4)	-3.15 (-3.34 to -2.96)
Malaysia	310 (225.1 to 423.5)	3661.4 (2630.5 to 5096)	310 (225.1 to 423.5)	3661.4 (2630.5 to 5096)	-7.04 (-7.60 to -6.48)
Maldives	39.1 (32 to 46.4)	36399.1 (29229.8 to 43590.6)	39.1 (32 to 46.4)	36399.1 (29229.8 to 43590.6)	-9.01 (-9.46 to -8.55)
Mali	3274.4 (3083.8 to 3467.6)	77986.4 (72785.7 to 82858.3)	3274.4 (3083.8 to 3467.6)	77986.4 (72785.7 to 82858.3)	-2.54 (-2.62 to -2.46)
Malta	4.6 (3.3 to 6.3)	2762.2 (1894.1 to 3949.9)	4.6 (3.3 to 6.3)	2762.2 (1894.1 to 3949.9)	-4.20 (-4.54 to -3.87)
Marshall Islands	12.9 (11.2 to 14.4)	55509.1 (47082.1 to 62925.3)	12.9 (11.2 to 14.4)	55509.1 (47082.1 to 62925.3)	-2.25 (-2.33 to -2.17)
Mauritania	445.5 (381.4 to 508.8)	44648.4 (37802.7 to 51638.6)	445.5 (381.4 to 508.8)	44648.4 (37802.7 to 51638.6)	-4.10 (-4.17 to -4.02)
Mauritius	42.1 (31.4 to 56.6)	8459.6 (6184.7 to 11601.3)	42.1 (31.4 to 56.6)	8459.6 (6184.7 to 11601.3)	-6.70 (-7.06 to -6.33)
Mexico	9171.1 (7465.9 to 11091.2)	23049.5 (18452.8 to 28200.5)	9171.1 (7465.9 to 11091.2)	23049.5 (18452.8 to 28200.5)	-3.31 (-3.40 to -3.22)
Micronesia (Federated States of)	32.1 (28.9 to 35.3)	60386.7 (53464.5 to 67237.1)	32.1 (28.9 to 35.3)	60386.7 (53464.5 to 67237.1)	-1.20 (-1.26 to -1.15)
Monaco	0.1 (0 to 0.1)	463.5 (338.9 to 638.1)	0.1 (0 to 0.1)	463.5 (338.9 to 638.1)	-1.73 (-1.94 to -1.52)
Mongolia	73.4 (55.2 to 95.8)	6820.7 (4973.8 to 9025.6)	73.4 (55.2 to 95.8)	6820.7 (4973.8 to 9025.6)	-5.53 (-5.78 to -5.28)
Montenegro	20 (15.1 to 25.4)	7113.2 (5193 to 9232.8)	20 (15.1 to 25.4)	7113.2 (5193 to 9232.8)	-2.73 (-3.31 to -2.15)
Morocco	3266.4 (2690.3 to 3955.7)	26110.9 (20746 to 32408.3)	3266.4 (2690.3 to 3955.7)	26110.9 (20746 to 32408.3)	-4.11 (-4.35 to -3.88)
Mozambique	4853 (4523.8 to 5139.6)	76868.4 (70468.9 to 82264.5)	4853 (4523.8 to 5139.6)	76868.4 (70468.9 to 82264.5)	-2.85 (-3.00 to -2.71)
Myanmar	7399.3 (6267.9 to 8632.8)	39010.5 (32566 to 46307.7)	7399.3 (6267.9 to 8632.8)	39010.5 (32566 to 46307.7)	-7.34 (-7.57 to -7.10)
Namibia	119 (92.6 to 148.9)	18052 (13654.5 to 22983.2)	119 (92.6 to 148.9)	18052 (13654.5 to 22983.2)	-3.28 (-3.48 to -3.07)
Nauru	1 (0.8 to 1.3)	20662.1 (15532.9 to 26623.7)	1 (0.8 to 1.3)	20662.1 (15532.9 to 26623.7)	-0.79 (-1.54 to -0.03)
Nepal	2119.7 (1751.6 to 2538.9)	21567.5 (17139.3 to 26359.5)	2119.7 (1751.6 to 2538.9)	21567.5 (17139.3 to 26359.5)	-5.77 (-5.91 to -5.63)
Netherlands	61.7 (45.8 to 81.7)	918.7 (642.1 to 1257.4)	61.7 (45.8 to 81.7)	918.7 (642.1 to 1257.4)	-2.01 (-2.23 to -1.79)
New Zealand	8.7 (6.5 to 11.5)	564.9 (409 to 772.3)	8.7 (6.5 to 11.5)	564.9 (409 to 772.3)	-0.68 (-0.96 to -0.39)

Continued on next page

Table S15 – continued from previous page

Country	1990		2019		1990–2019
	Incident cases No.×10^4[95% UI]	ASR per 10,0000 No.[95% UI]	Incident cases No.×10^4[95% UI]	ASR per 10,0000 No.[95% UI]	EAPC No.[95% CI]
Nicaragua	239.6 (186.4 to 302.8)	12417.1 (9383.7 to 15967.2)	239.6 (186.4 to 302.8)	12417.1 (9383.7 to 15967.2)	-7.35 (-7.93 to -6.76)
Niger	3257.4 (3082 to 3405.4)	82826.3 (77714.7 to 86821.4)	3257.4 (3082 to 3405.4)	82826.3 (77714.7 to 86821.4)	-1.33 (-1.43 to -1.22)
Nigeria	9846.3 (8182.3 to 11643.6)	21655 (17544.9 to 26158.6)	9846.3 (8182.3 to 11643.6)	21655 (17544.9 to 26158.6)	-5.69 (-6.10 to -5.27)
Niue	0.2 (0.1 to 0.2)	16646.5 (12309 to 21494.1)	0.2 (0.1 to 0.2)	16646.5 (12309 to 21494.1)	-3.04 (-3.20 to -2.87)
North Macedonia	275.2 (224.6 to 325.1)	28315.4 (22697.3 to 33883.9)	275.2 (224.6 to 325.1)	28315.4 (22697.3 to 33883.9)	-3.12 (-3.32 to -2.91)
Northern Mariana Islands	1.4 (1 to 1.8)	6127.4 (4461.4 to 8327.7)	1.4 (1 to 1.8)	6127.4 (4461.4 to 8327.7)	-0.14 (-0.40 to 0.12)
Norway	17.8 (13.2 to 23.7)	941.5 (662.1 to 1322.4)	17.8 (13.2 to 23.7)	941.5 (662.1 to 1322.4)	-3.16 (-3.39 to -2.93)
Oman	166.8 (129.7 to 212.4)	14705.5 (11127 to 19113.3)	166.8 (129.7 to 212.4)	14705.5 (11127 to 19113.3)	-8.05 (-8.64 to -7.46)
Pakistan	14739.7 (12449.3 to 17381.5)	24988.5 (20630.8 to 30097.7)	14739.7 (12449.3 to 17381.5)	24988.5 (20630.8 to 30097.7)	-7.10 (-7.55 to -6.65)
Palau	1 (0.7 to 1.3)	13418.2 (9811.3 to 17645.1)	1 (0.7 to 1.3)	13418.2 (9811.3 to 17645.1)	-2.28 (-2.48 to -2.08)
Palestine	585.6 (509 to 653.7)	56223.7 (48253.1 to 63366.8)	585.6 (509 to 653.7)	56223.7 (48253.1 to 63366.8)	-7.30 (-7.76 to -6.84)
Panama	84 (65.9 to 106.4)	7035.4 (5331.1 to 9237.4)	84 (65.9 to 106.4)	7035.4 (5331.1 to 9237.4)	-3.15 (-3.41 to -2.89)
Papua New Guinea	387.9 (300 to 477.8)	18540.1 (14089 to 23251.4)	387.9 (300 to 477.8)	18540.1 (14089 to 23251.4)	-0.63 (-1.08 to -0.18)
Paraguay	333.3 (259.4 to 424.2)	16948.8 (12875.4 to 22128)	333.3 (259.4 to 424.2)	16948.8 (12875.4 to 22128)	-2.87 (-3.03 to -2.72)
Peru	1722.1 (1394.7 to 2113.5)	16554.1 (13166.1 to 20574.8)	1722.1 (1394.7 to 2113.5)	16554.1 (13166.1 to 20574.8)	-3.52 (-3.70 to -3.34)
Philippines	6507.9 (5284.9 to 7993.8)	21274.2 (16910.4 to 26809.4)	6507.9 (5284.9 to 7993.8)	21274.2 (16910.4 to 26809.4)	-1.75 (-2.19 to -1.30)
Poland	1700.8 (1310.4 to 2176)	10216.1 (7677.7 to 13388.9)	1700.8 (1310.4 to 2176)	10216.1 (7677.7 to 13388.9)	-3.63 (-3.70 to -3.56)
Portugal	159.2 (120.2 to 210.6)	3616.9 (2606.4 to 4996.8)	159.2 (120.2 to 210.6)	3616.9 (2606.4 to 4996.8)	-3.71 (-4.11 to -3.29)
Puerto Rico	43.7 (32.6 to 57.5)	2671 (1915.5 to 3606.9)	43.7 (32.6 to 57.5)	2671 (1915.5 to 3606.9)	-3.66 (-3.77 to -3.54)
Qatar	10.8 (8 to 14.5)	3652.5 (2634.3 to 4934.1)	10.8 (8 to 14.5)	3652.5 (2634.3 to 4934.1)	-5.48 (-5.72 to -5.23)
Republic of Korea	425.8 (305.5 to 582.4)	2098.6 (1457.8 to 2941.9)	425.8 (305.5 to 582.4)	2098.6 (1457.8 to 2941.9)	-4.57 (-4.70 to -4.44)
Republic of Moldova	66 (50 to 84.4)	3275.8 (2433.1 to 4286.9)	66 (50 to 84.4)	3275.8 (2433.1 to 4286.9)	-3.28 (-3.64 to -2.93)
Romania	1142.7 (869.8 to 1433.8)	11183.9 (8220.4 to 14353.9)	1142.7 (869.8 to 1433.8)	11183.9 (8220.4 to 14353.9)	-3.42 (-3.62 to -3.22)
Russian Federation	284.2 (217.2 to 364.3)	401.9 (299.5 to 535.2)	284.2 (217.2 to 364.3)	401.9 (299.5 to 535.2)	-2.83 (-2.94 to -2.72)
Rwanda	1415 (1192 to 1646.8)	39823.6 (32917.6 to 47302.5)	1415 (1192 to 1646.8)	39823.6 (32917.6 to 47302.5)	-3.28 (-3.64 to -2.92)
Saint Kitts and Nevis	1.1 (0.8 to 1.4)	5566.6 (3978 to 7435)	1.1 (0.8 to 1.4)	5566.6 (3978 to 7435)	-3.94 (-4.16 to -3.71)
Saint Lucia	4.8 (3.6 to 6.2)	7382.3 (5352.2 to 9784.2)	4.8 (3.6 to 6.2)	7382.3 (5352.2 to 9784.2)	-3.10 (-3.33 to -2.87)

Continued on next page

Table S15 – continued from previous page

Country	1990		2019		1990–2019
	Incident cases No.×10^4[95% UI]	ASR per 10,0000 No.[95% UI]	Incident cases No.×10^4[95% UI]	ASR per 10,0000 No.[95% UI]	EAPC No.[95% CI]
Saint Vincent and the Grenadines	6.9 (5.3 to 8.8)	13051 (9758.5 to 17014)	6.9 (5.3 to 8.8)	13051 (9758.5 to 17014)	-4.23 (-4.47 to -3.99)
Samoa	24.1 (19.5 to 29.2)	30021.4 (23895.6 to 36996.9)	24.1 (19.5 to 29.2)	30021.4 (23895.6 to 36996.9)	-0.65 (-0.82 to -0.48)
San Marino	0.1 (0.1 to 0.1)	798.6 (554.5 to 1150.1)	0.1 (0.1 to 0.1)	798.6 (554.5 to 1150.1)	-2.08 (-2.45 to -1.72)
Sao Tome and Principe	41 (37.3 to 44.2)	69854.6 (62632.2 to 75835.7)	41 (37.3 to 44.2)	69854.6 (62632.2 to 75835.7)	-4.69 (-4.77 to -4.62)
Saudi Arabia	351.8 (250.4 to 508.6)	4023.3 (2745.3 to 5993.5)	351.8 (250.4 to 508.6)	4023.3 (2745.3 to 5993.5)	-9.63 (-10.49 to -8.76)
Senegal	3161.5 (3037.3 to 3261.6)	86673 (83014.3 to 89461.5)	3161.5 (3037.3 to 3261.6)	86673 (83014.3 to 89461.5)	-4.65 (-5.16 to -4.14)
Serbia	1414.4 (1193.5 to 1627.3)	32375.3 (26883.3 to 37883)	1414.4 (1193.5 to 1627.3)	32375.3 (26883.3 to 37883)	-2.42 (-2.67 to -2.17)
Seychelles	2.5 (1.9 to 3.4)	7787.7 (5570.6 to 10566.7)	2.5 (1.9 to 3.4)	7787.7 (5570.6 to 10566.7)	-6.10 (-6.73 to -5.48)
Sierra Leone	1265.8 (1171.5 to 1349.5)	72679.8 (66493.8 to 77770.4)	1265.8 (1171.5 to 1349.5)	72679.8 (66493.8 to 77770.4)	-2.87 (-2.97 to -2.76)
Singapore	24.1 (17.9 to 32.7)	1727.6 (1237 to 2416.5)	24.1 (17.9 to 32.7)	1727.6 (1237 to 2416.5)	-4.03 (-4.14 to -3.92)
Slovakia	235 (176.7 to 299.1)	10178.2 (7388.3 to 13183.1)	235 (176.7 to 299.1)	10178.2 (7388.3 to 13183.1)	-3.27 (-3.36 to -3.17)
Slovenia	49.8 (37.4 to 64)	5852.7 (4262.5 to 7694.6)	49.8 (37.4 to 64)	5852.7 (4262.5 to 7694.6)	-2.46 (-2.59 to -2.33)
Solomon Islands	82.8 (71.4 to 94.5)	49032.9 (41576.5 to 56363.5)	82.8 (71.4 to 94.5)	49032.9 (41576.5 to 56363.5)	-1.02 (-1.23 to -0.82)
Somalia	2887.6 (2681.8 to 3063.4)	77680 (71182.2 to 83125.7)	2887.6 (2681.8 to 3063.4)	77680 (71182.2 to 83125.7)	-0.29 (-0.33 to -0.26)
South Africa	2527.9 (2037.5 to 3101.3)	15736.3 (12358.1 to 19481.7)	2527.9 (2037.5 to 3101.3)	15736.3 (12358.1 to 19481.7)	-4.91 (-5.05 to -4.78)
South Sudan	1427.1 (1227.9 to 1641.8)	45271 (38257.9 to 52830.4)	1427.1 (1227.9 to 1641.8)	45271 (38257.9 to 52830.4)	-2.45 (-2.63 to -2.27)
Spain	292.3 (220.2 to 381)	1655.6 (1217 to 2266.2)	292.3 (220.2 to 381)	1655.6 (1217 to 2266.2)	-2.73 (-2.98 to -2.48)
Sri Lanka	1448.3 (1177.5 to 1786.8)	18624.8 (14789.3 to 23155)	1448.3 (1177.5 to 1786.8)	18624.8 (14789.3 to 23155)	-6.75 (-6.97 to -6.53)
Sudan	4529.7 (3914.8 to 5198.1)	44110.3 (37387.6 to 51490.5)	4529.7 (3914.8 to 5198.1)	44110.3 (37387.6 to 51490.5)	-4.72 (-5.03 to -4.41)
Suriname	20.7 (15.9 to 26.6)	11082.5 (8281.5 to 14453.4)	20.7 (15.9 to 26.6)	11082.5 (8281.5 to 14453.4)	-3.52 (-3.71 to -3.34)
Sweden	45.9 (34.3 to 62.3)	1208.2 (848.1 to 1706.3)	45.9 (34.3 to 62.3)	1208.2 (848.1 to 1706.3)	-2.96 (-3.32 to -2.60)
Switzerland	15.7 (11.6 to 20.8)	512.7 (359 to 718.5)	15.7 (11.6 to 20.8)	512.7 (359 to 718.5)	-1.95 (-2.10 to -1.80)
Syrian Arab Republic	1072.9 (805.7 to 1384.4)	16166.1 (11762.9 to 21422.7)	1072.9 (805.7 to 1384.4)	16166.1 (11762.9 to 21422.7)	-4.71 (-4.96 to -4.46)
Taiwan (Province of China)	364.1 (263.7 to 482)	3888 (2705.5 to 5249.3)	364.1 (263.7 to 482)	3888 (2705.5 to 5249.3)	-6.23 (-6.55 to -5.90)
Tajikistan	419.7 (332.1 to 521.4)	15601.3 (11953.6 to 19832.1)	419.7 (332.1 to 521.4)	15601.3 (11953.6 to 19832.1)	-2.10 (-2.70 to -1.49)
Thailand	4835 (3911.4 to 5895.2)	18233.3 (14637.7 to 22298.7)	4835 (3911.4 to 5895.2)	18233.3 (14637.7 to 22298.7)	-8.16 (-8.57 to -7.74)
Timor-Leste	153 (129.1 to 176.9)	39341.5 (32483.5 to 46144.9)	153 (129.1 to 176.9)	39341.5 (32483.5 to 46144.9)	-6.18 (-6.65 to -5.72)
Togo	1206 (1089.7 to 1311.9)	68855.7 (61409.4 to 75378.7)	1206 (1089.7 to 1311.9)	68855.7 (61409.4 to 75378.7)	-3.03 (-3.15 to -2.91)
Tokelau	0.2 (0.2 to 0.3)	31175.9 (24304.2 to 38413.9)	0.2 (0.2 to 0.3)	31175.9 (24304.2 to 38413.9)	-3.05 (-3.25 to -2.86)

Continued on next page

Table S15 – continued from previous page

Country	1990		2019		1990–2019
	Incident cases No.×10^4[95% UI]	ASR per 10,0000 No.[95% UI]	Incident cases No.×10^4[95% UI]	ASR per 10,0000 No.[95% UI]	EAPC No.[95% CI]
Tonga	13 (10.4 to 16)	28713.8 (22362.8 to 35937.7)	13 (10.4 to 16)	28713.8 (22362.8 to 35937.7)	-1.64 (-1.88 to -1.40)
Trinidad and Tobago	27.9 (20.9 to 36.5)	4850.7 (3523.7 to 6573.2)	27.9 (20.9 to 36.5)	4850.7 (3523.7 to 6573.2)	-3.95 (-4.21 to -3.68)
Tunisia	398.5 (303.3 to 519.8)	9346.8 (6846.4 to 12622.9)	398.5 (303.3 to 519.8)	9346.8 (6846.4 to 12622.9)	-5.46 (-5.71 to -5.20)
Turkey	3941.5 (3278.9 to 4695.1)	13096.3 (10642.1 to 15757.1)	3941.5 (3278.9 to 4695.1)	13096.3 (10642.1 to 15757.1)	-5.81 (-5.94 to -5.68)
Turkmenistan	123.8 (95 to 160.9)	6813.3 (5028.1 to 9072.6)	123.8 (95 to 160.9)	6813.3 (5028.1 to 9072.6)	-3.86 (-4.22 to -3.50)
Tuvalu	1.6 (1.3 to 1.8)	37420.9 (30902.5 to 44648.2)	1.6 (1.3 to 1.8)	37420.9 (30902.5 to 44648.2)	-1.92 (-2.09 to -1.75)
Uganda	3245.4 (2744.8 to 3750.4)	36376.9 (29951 to 43106.9)	3245.4 (2744.8 to 3750.4)	36376.9 (29951 to 43106.9)	-3.94 (-4.15 to -3.73)
Ukraine	403.1 (307.3 to 525.6)	1764.6 (1310.8 to 2378.2)	403.1 (307.3 to 525.6)	1764.6 (1310.8 to 2378.2)	-2.53 (-2.69 to -2.37)
United Arab Emirates	33.2 (24.6 to 44.9)	2745.9 (2010.9 to 3754.7)	33.2 (24.6 to 44.9)	2745.9 (2010.9 to 3754.7)	-4.89 (-5.39 to -4.38)
United Kingdom	347.1 (259.2 to 462.2)	1385.1 (979.9 to 1917)	347.1 (259.2 to 462.2)	1385.1 (979.9 to 1917)	-2.78 (-3.07 to -2.49)
United Republic of Tanzania	4520.4 (3848.1 to 5198.3)	34681.2 (28862.9 to 40840.6)	4520.4 (3848.1 to 5198.3)	34681.2 (28862.9 to 40840.6)	-2.81 (-3.02 to -2.60)
United States of America	344.9 (252.6 to 457.1)	323.1 (224.7 to 451.5)	344.9 (252.6 to 457.1)	323.1 (224.7 to 451.5)	-2.16 (-2.74 to -1.57)
United States Virgin Islands	1.5 (1.1 to 1.9)	3025.8 (2216.3 to 3983.3)	1.5 (1.1 to 1.9)	3025.8 (2216.3 to 3983.3)	-4.62 (-4.90 to -4.33)
Uruguay	133.5 (99.8 to 172.1)	10195.1 (7413.3 to 13429.2)	133.5 (99.8 to 172.1)	10195.1 (7413.3 to 13429.2)	-1.67 (-1.95 to -1.39)
Uzbekistan	1014.5 (740.9 to 1329.1)	9855.4 (7005.2 to 13101.7)	1014.5 (740.9 to 1329.1)	9855.4 (7005.2 to 13101.7)	-3.96 (-4.19 to -3.72)
Vanuatu	37.6 (32.2 to 42.4)	50650.5 (42673 to 57812.8)	37.6 (32.2 to 42.4)	50650.5 (42673 to 57812.8)	-1.00 (-1.15 to -0.85)
Venezuela (Bolivarian Republic of)	966.9 (777.8 to 1203.7)	10427.7 (8120.9 to 13228.8)	966.9 (777.8 to 1203.7)	10427.7 (8120.9 to 13228.8)	-1.49 (-2.04 to -0.93)
Viet Nam	2842.3 (2093.3 to 3686.2)	9398.5 (6758.7 to 12445.6)	2842.3 (2093.3 to 3686.2)	9398.5 (6758.7 to 12445.6)	-5.22 (-5.60 to -4.84)
Yemen	3703.1 (3239.2 to 4187.4)	52553.7 (45017 to 60370.7)	3703.1 (3239.2 to 4187.4)	52553.7 (45017 to 60370.7)	-4.39 (-4.69 to -4.09)
Zambia	1972.7 (1710 to 2233.8)	49533.8 (42148.7 to 57088)	1972.7 (1710 to 2233.8)	49533.8 (42148.7 to 57088)	-3.83 (-4.27 to -3.39)
Zimbabwe	1641.6 (1353 to 1943.3)	33361.1 (26968 to 40343.1)	1641.6 (1353 to 1943.3)	33361.1 (26968 to 40343.1)	-1.05 (-1.44 to -0.65)

Abbreviation: ASR, age standardized rate; CI, confidence interval; EAPC, estimated annual percentage change; UI, uncertainty interval.

Table S16: The change of protein-energy malnutrition cases between 1990 and 2019 at national level for both gender.

Country	1990		2019		1990–2019
	Incident cases No.×10^4[95% UI]	ASR per 10,0000 No.[95% UI]	Incident cases No.×10^4[95% UI]	ASR per 10,0000 No.[95% UI]	EAPC No.[95% CI]
Afghanistan	205.5 (160.6 to 264.1)	1073.2 (874.6 to 1313.8)	205.5 (160.6 to 264.1)	1073.2 (874.6 to 1313.8)	-0.26 (-0.44 to -0.08)
Albania	67.6 (50.1 to 88.2)	1775.2 (1340.8 to 2283.7)	67.6 (50.1 to 88.2)	1775.2 (1340.8 to 2283.7)	0.23 (0.08 to 0.39)
Algeria	418.7 (352.1 to 501.1)	1338.4 (1129.3 to 1581.3)	418.7 (352.1 to 501.1)	1338.4 (1129.3 to 1581.3)	-0.15 (-0.36 to 0.07)
American Samoa	0.8 (0.7 to 0.9)	1438.9 (1264.2 to 1630.2)	0.8 (0.7 to 0.9)	1438.9 (1264.2 to 1630.2)	-0.26 (-0.39 to -0.14)
Andorra	0.5 (0.4 to 0.6)	959.4 (763.4 to 1205.9)	0.5 (0.4 to 0.6)	959.4 (763.4 to 1205.9)	0.26 (0.09 to 0.44)
Angola	246.9 (192 to 332.6)	1518.3 (1274.8 to 1870.5)	246.9 (192 to 332.6)	1518.3 (1274.8 to 1870.5)	-2.25 (-2.41 to -2.09)
Antigua and Barbuda	0.7 (0.6 to 0.8)	1129 (978.3 to 1307.9)	0.7 (0.6 to 0.8)	1129 (978.3 to 1307.9)	-0.52 (-0.61 to -0.44)
Argentina	271.3 (227.8 to 322.7)	823.7 (690.3 to 979.3)	271.3 (227.8 to 322.7)	823.7 (690.3 to 979.3)	0.11 (-0.11 to 0.32)
Armenia	23.5 (18.9 to 29.2)	665.8 (538.4 to 823.3)	23.5 (18.9 to 29.2)	665.8 (538.4 to 823.3)	0.16 (0.08 to 0.25)
Australia	78 (64.7 to 94.6)	510.9 (432.6 to 610)	78 (64.7 to 94.6)	510.9 (432.6 to 610)	0.84 (0.60 to 1.07)
Austria	79 (60.5 to 101.8)	1044.1 (812.2 to 1314)	79 (60.5 to 101.8)	1044.1 (812.2 to 1314)	-0.02 (-0.17 to 0.12)
Azerbaijan	75.6 (56.8 to 102)	870.7 (662.1 to 1159.4)	75.6 (56.8 to 102)	870.7 (662.1 to 1159.4)	-0.89 (-1.09 to -0.68)
Bahamas	5 (4.1 to 6.1)	2043.3 (1674.5 to 2470.9)	5 (4.1 to 6.1)	2043.3 (1674.5 to 2470.9)	-0.36 (-0.69 to -0.03)
Bahrain	7.9 (6.9 to 9.1)	1552.5 (1354.2 to 1765.1)	7.9 (6.9 to 9.1)	1552.5 (1354.2 to 1765.1)	0.15 (0.01 to 0.30)
Bangladesh	4030.4 (3532.8 to 4571.8)	2641.2 (2339.9 to 2971.9)	4030.4 (3532.8 to 4571.8)	2641.2 (2339.9 to 2971.9)	-1.70 (-2.08 to -1.32)
Barbados	2.2 (1.9 to 2.6)	930.3 (800.5 to 1075)	2.2 (1.9 to 2.6)	930.3 (800.5 to 1075)	-0.16 (-0.34 to 0.02)
Belarus	63.2 (49.4 to 79.8)	763.7 (575.3 to 994.2)	63.2 (49.4 to 79.8)	763.7 (575.3 to 994.2)	-0.23 (-0.33 to -0.14)
Belgium	112.3 (90.8 to 138.3)	1096.5 (888.5 to 1342.4)	112.3 (90.8 to 138.3)	1096.5 (888.5 to 1342.4)	0.77 (0.58 to 0.95)
Belize	2 (1.6 to 2.5)	967 (801.2 to 1150.4)	2 (1.6 to 2.5)	967 (801.2 to 1150.4)	-1.55 (-1.70 to -1.40)
Benin	135.7 (111.7 to 169.6)	1699 (1446.3 to 2041.9)	135.7 (111.7 to 169.6)	1699 (1446.3 to 2041.9)	-1.45 (-1.58 to -1.33)
Bermuda	0.5 (0.4 to 0.6)	939.4 (777 to 1140.2)	0.5 (0.4 to 0.6)	939.4 (777 to 1140.2)	0.36 (0.18 to 0.55)
Bhutan	15.3 (12.1 to 19.3)	1881.9 (1540.3 to 2304.8)	15.3 (12.1 to 19.3)	1881.9 (1540.3 to 2304.8)	1.14 (0.82 to 1.46)
Bolivia (Plurinational State of)	60.3 (52.9 to 69.3)	830.9 (732 to 935.5)	60.3 (52.9 to 69.3)	830.9 (732 to 935.5)	-0.68 (-0.83 to -0.52)
Bosnia and Herzegovina	32.4 (24.4 to 42.3)	823.4 (619.5 to 1103.3)	32.4 (24.4 to 42.3)	823.4 (619.5 to 1103.3)	0.50 (0.42 to 0.58)
Botswana	15.4 (13.2 to 17.9)	985.6 (859.7 to 1126.2)	15.4 (13.2 to 17.9)	985.6 (859.7 to 1126.2)	0.07 (-0.13 to 0.28)
Brazil	1168.3 (950.9 to 1438.3)	799.3 (651.4 to 974.5)	1168.3 (950.9 to 1438.3)	799.3 (651.4 to 974.5)	-1.38 (-1.63 to -1.12)

Continued on next page

Table S16 – continued from previous page

Country	1990		2019		1990–2019
	Incident cases No.$\times10^4$[95% UI]	ASR per 10,0000 No.[95% UI]	Incident cases No.$\times10^4$[95% UI]	ASR per 10,0000 No.[95% UI]	EAPC No.[95% CI]
Brunei Darussalam	2.3 (2.1 to 2.6)	824.3 (726.7 to 942)	2.3 (2.1 to 2.6)	824.3 (726.7 to 942)	0.07 (0.00 to 0.13)
Bulgaria	52.6 (40.3 to 67.8)	800.1 (610.4 to 1042.6)	52.6 (40.3 to 67.8)	800.1 (610.4 to 1042.6)	0.57 (0.46 to 0.69)
Burkina Faso	289.7 (250.4 to 346)	1782 (1582.3 to 2058.6)	289.7 (250.4 to 346)	1782 (1582.3 to 2058.6)	-0.52 (-0.92 to -0.12)
Burundi	149.2 (122.7 to 185)	1719.9 (1478.4 to 2031.6)	149.2 (122.7 to 185)	1719.9 (1478.4 to 2031.6)	-1.53 (-1.63 to -1.43)
Cabo Verde	3.4 (2.8 to 4.2)	798.6 (678.9 to 943.4)	3.4 (2.8 to 4.2)	798.6 (678.9 to 943.4)	-0.28 (-0.51 to -0.04)
Cambodia	359.3 (269 to 486.5)	2497.2 (2021.6 to 3119.1)	359.3 (269 to 486.5)	2497.2 (2021.6 to 3119.1)	-1.34 (-1.52 to -1.15)
Cameroon	162.5 (132.9 to 200.6)	1056.9 (895.3 to 1236.9)	162.5 (132.9 to 200.6)	1056.9 (895.3 to 1236.9)	-0.67 (-0.84 to -0.50)
Canada	190.5 (155.5 to 230.8)	684.1 (559 to 827.7)	190.5 (155.5 to 230.8)	684.1 (559 to 827.7)	-0.10 (-0.28 to 0.09)
Central African Republic	51.1 (40.7 to 65)	1158.2 (970.4 to 1399.6)	51.1 (40.7 to 65)	1158.2 (970.4 to 1399.6)	-0.22 (-0.35 to -0.09)
Chad	220.9 (185.9 to 269.6)	2013.8 (1761.8 to 2354.9)	220.9 (185.9 to 269.6)	2013.8 (1761.8 to 2354.9)	-0.91 (-1.02 to -0.80)
Chile	61.3 (51.9 to 72.1)	476.7 (401.1 to 559.6)	61.3 (51.9 to 72.1)	476.7 (401.1 to 559.6)	0.69 (0.25 to 1.13)
China	18519.8 (13881.7 to 24503.5)	1613.5 (1208.9 to 2130.8)	18519.8 (13881.7 to 24503.5)	1613.5 (1208.9 to 2130.8)	0.61 (0.44 to 0.79)
Colombia	235.9 (205 to 266.6)	715.7 (618.4 to 807.6)	235.9 (205 to 266.6)	715.7 (618.4 to 807.6)	-0.73 (-0.95 to -0.51)
Comoros	10.2 (8.9 to 11.7)	1590.1 (1408.4 to 1777.9)	10.2 (8.9 to 11.7)	1590.1 (1408.4 to 1777.9)	-1.19 (-1.29 to -1.10)
Congo	30.4 (23.8 to 38.7)	894.1 (741.8 to 1073.1)	30.4 (23.8 to 38.7)	894.1 (741.8 to 1073.1)	-0.69 (-0.85 to -0.52)
Cook Islands	0.3 (0.2 to 0.3)	1412.8 (1233.9 to 1603.8)	0.3 (0.2 to 0.3)	1412.8 (1233.9 to 1603.8)	0.06 (-0.06 to 0.17)
Costa Rica	23.4 (19.4 to 28.3)	761.1 (626.6 to 928.5)	23.4 (19.4 to 28.3)	761.1 (626.6 to 928.5)	0.00 (-0.26 to 0.26)
Croatia	24.4 (19.2 to 30.8)	635.2 (509.3 to 792.4)	24.4 (19.2 to 30.8)	635.2 (509.3 to 792.4)	0.43 (0.33 to 0.54)
Cuba	71.5 (58.5 to 88.9)	698.7 (574.9 to 855.1)	71.5 (58.5 to 88.9)	698.7 (574.9 to 855.1)	0.36 (0.11 to 0.62)
Cyprus	8.3 (6.8 to 10.3)	1082.9 (883.3 to 1330.5)	8.3 (6.8 to 10.3)	1082.9 (883.3 to 1330.5)	0.64 (0.54 to 0.73)
Czechia	54.4 (42.2 to 69.6)	668.2 (515 to 872.9)	54.4 (42.2 to 69.6)	668.2 (515 to 872.9)	1.83 (1.56 to 2.11)
Ivoirian	231.5 (191.9 to 281)	1215 (1042.1 to 1411.8)	231.5 (191.9 to 281)	1215 (1042.1 to 1411.8)	-0.87 (-1.00 to -0.75)
Democratic People's Republic of Korea	616.4 (466.4 to 825.5)	2285 (1799.1 to 2931.1)	616.4 (466.4 to 825.5)	2285 (1799.1 to 2931.1)	-1.72 (-2.30 to -1.13)
Democratic Republic of the Congo	1036 (781.6 to 1357.4)	1547.5 (1248.7 to 1899.2)	1036 (781.6 to 1357.4)	1547.5 (1248.7 to 1899.2)	-0.95 (-1.28 to -0.63)
Denmark	49.3 (38 to 63.1)	989.6 (776.7 to 1252.7)	49.3 (38 to 63.1)	989.6 (776.7 to 1252.7)	0.58 (0.29 to 0.86)
Djibouti	16.4 (14.1 to 19.4)	2227.5 (1970.2 to 2547.8)	16.4 (14.1 to 19.4)	2227.5 (1970.2 to 2547.8)	-0.94 (-1.05 to -0.82)
Dominica	0.8 (0.7 to 1)	1092.5 (933.4 to 1280.4)	0.8 (0.7 to 1)	1092.5 (933.4 to 1280.4)	-0.43 (-0.56 to -0.29)

Continued on next page

Table S16 – continued from previous page

Country	1990		2019		1990–2019
	Incident cases No.×10^4[95% UI]	ASR per 10,0000 No.[95% UI]	Incident cases No.×10^4[95% UI]	ASR per 10,0000 No.[95% UI]	EAPC No.[95% CI]
Dominican Republic	69.4 (55.4 to 85.7)	825 (685.6 to 967)	69.4 (55.4 to 85.7)	825 (685.6 to 967)	-1.61 (-1.74 to -1.48)
Ecuador	81 (72.2 to 90.2)	823.9 (732.5 to 922.4)	81 (72.2 to 90.2)	823.9 (732.5 to 922.4)	-1.07 (-1.27 to -0.87)
Egypt	837.3 (684.1 to 1025.6)	1162.6 (966.7 to 1405.8)	837.3 (684.1 to 1025.6)	1162.6 (966.7 to 1405.8)	-0.42 (-0.63 to -0.20)
El Salvador	45.5 (38.9 to 53.2)	860.4 (749.8 to 986.3)	45.5 (38.9 to 53.2)	860.4 (749.8 to 986.3)	-0.15 (-0.31 to 0.00)
Equatorial Guinea	9.2 (8.3 to 10.5)	1151.5 (1054.5 to 1272.4)	9.2 (8.3 to 10.5)	1151.5 (1054.5 to 1272.4)	-2.50 (-2.92 to -2.07)
Eritrea	110.9 (90.9 to 139.8)	2522.4 (2149.9 to 3034.1)	110.9 (90.9 to 139.8)	2522.4 (2149.9 to 3034.1)	-2.02 (-2.23 to -1.82)
Estonia	11.7 (9.7 to 14)	913.6 (748.3 to 1120.1)	11.7 (9.7 to 14)	913.6 (748.3 to 1120.1)	-0.29 (-0.55 to -0.02)
Eswatini	8.9 (6.6 to 11.7)	890.7 (717.3 to 1090.4)	8.9 (6.6 to 11.7)	890.7 (717.3 to 1090.4)	-0.71 (-0.83 to -0.59)
Ethiopia	1378.3 (1102.2 to 1766.9)	1821.6 (1531.4 to 2175.1)	1378.3 (1102.2 to 1766.9)	1821.6 (1531.4 to 2175.1)	-1.17 (-1.42 to -0.92)
Fiji	10.2 (9.1 to 11.4)	1318 (1167.1 to 1475.8)	10.2 (9.1 to 11.4)	1318 (1167.1 to 1475.8)	-0.38 (-0.55 to -0.21)
Finland	47.7 (36.4 to 60.7)	997.1 (775.6 to 1248.6)	47.7 (36.4 to 60.7)	997.1 (775.6 to 1248.6)	-0.05 (-0.19 to 0.09)
France	917.8 (771.2 to 1076.7)	1483.5 (1251.2 to 1741.5)	917.8 (771.2 to 1076.7)	1483.5 (1251.2 to 1741.5)	0.38 (0.22 to 0.53)
Gabon	10.6 (8.5 to 13.4)	802.1 (671.3 to 954.3)	10.6 (8.5 to 13.4)	802.1 (671.3 to 954.3)	-0.83 (-0.98 to -0.69)
Gambia	28.4 (23.5 to 34.7)	1860.4 (1595.7 to 2185.8)	28.4 (23.5 to 34.7)	1860.4 (1595.7 to 2185.8)	-0.84 (-1.06 to -0.62)
Georgia	26 (19.7 to 34)	552.8 (418.5 to 730.5)	26 (19.7 to 34)	552.8 (418.5 to 730.5)	-0.24 (-0.49 to 0.01)
Germany	642.2 (490.9 to 837.2)	806.6 (615.2 to 1063.7)	642.2 (490.9 to 837.2)	806.6 (615.2 to 1063.7)	0.61 (0.47 to 0.75)
Ghana	320.8 (272.3 to 382.5)	1502.9 (1300.1 to 1738.3)	320.8 (272.3 to 382.5)	1502.9 (1300.1 to 1738.3)	-1.30 (-1.48 to -1.11)
Greece	55 (42.6 to 71.1)	598.5 (479.1 to 758.9)	55 (42.6 to 71.1)	598.5 (479.1 to 758.9)	-0.04 (-0.20 to 0.11)
Greenland	0.3 (0.3 to 0.4)	583.8 (487.1 to 699.2)	0.3 (0.3 to 0.4)	583.8 (487.1 to 699.2)	0.22 (0.05 to 0.40)
Grenada	0.9 (0.7 to 1)	1036.8 (881.2 to 1200.6)	0.9 (0.7 to 1)	1036.8 (881.2 to 1200.6)	-0.25 (-0.37 to -0.13)
Guam	1.9 (1.7 to 2.3)	1383.5 (1194.5 to 1599.6)	1.9 (1.7 to 2.3)	1383.5 (1194.5 to 1599.6)	-0.68 (-0.89 to -0.47)
Guatemala	134.9 (121.8 to 150.2)	1670.1 (1490.9 to 1872.4)	134.9 (121.8 to 150.2)	1670.1 (1490.9 to 1872.4)	-2.51 (-2.66 to -2.36)
Guinea	135.2 (114 to 163.6)	1247.9 (1086.4 to 1447.5)	135.2 (114 to 163.6)	1247.9 (1086.4 to 1447.5)	-0.66 (-0.82 to -0.50)
Guinea-Bissau	21.9 (18.2 to 27.2)	1393.3 (1203.3 to 1651.2)	21.9 (18.2 to 27.2)	1393.3 (1203.3 to 1651.2)	-1.29 (-1.42 to -1.15)
Guyana	14.2 (12.2 to 16.6)	1695.5 (1475.8 to 1929.2)	14.2 (12.2 to 16.6)	1695.5 (1475.8 to 1929.2)	-1.92 (-2.08 to -1.76)
Haiti	124.2 (90.7 to 167.8)	1309 (1015.3 to 1669.9)	124.2 (90.7 to 167.8)	1309 (1015.3 to 1669.9)	-1.20 (-1.34 to -1.06)
Honduras	49.8 (42.7 to 57.5)	971.8 (846 to 1108)	49.8 (42.7 to 57.5)	971.8 (846 to 1108)	-1.38 (-1.56 to -1.20)
Hungary	43 (34.8 to 53.5)	540.9 (452.2 to 650.1)	43 (34.8 to 53.5)	540.9 (452.2 to 650.1)	0.41 (0.33 to 0.50)
Iceland	2.6 (2.1 to 3.3)	1038.5 (825.3 to 1285.1)	2.6 (2.1 to 3.3)	1038.5 (825.3 to 1285.1)	0.39 (0.28 to 0.50)
India	37667.2 (31021.8 to 46006.9)	3520.6 (2910.4 to 4257.3)	37667.2 (31021.8 to 46006.9)	3520.6 (2910.4 to 4257.3)	-0.01 (-0.16 to 0.14)
Indonesia	5957.7 (4786.4 to 7396.9)	3113.6 (2535.6 to 3800.5)	5957.7 (4786.4 to 7396.9)	3113.6 (2535.6 to 3800.5)	0.21 (0.06 to 0.36)
Iran (Islamic Republic of)	944.3 (754.3 to 1179.7)	1305 (1047.3 to 1615.8)	944.3 (754.3 to 1179.7)	1305 (1047.3 to 1615.8)	-0.18 (-0.38 to 0.03)

Continued on next page

Table S16 – continued from previous page

Country	1990		2019		1990–2019
	Incident cases No.×10^4[95% UI]	ASR per 10,0000 No.[95% UI]	Incident cases No.×10^4[95% UI]	ASR per 10,0000 No.[95% UI]	EAPC No.[95% CI]
Iraq	336.6 (283 to 401.4)	1238.8 (1055.6 to 1457)	336.6 (283 to 401.4)	1238.8 (1055.6 to 1457)	0.19 (0.01 to 0.36)
Ireland	32.6 (25.7 to 42)	943.8 (748.3 to 1204.7)	32.6 (25.7 to 42)	943.8 (748.3 to 1204.7)	0.12 (-0.01 to 0.25)
Israel	47.6 (38 to 59.4)	962.2 (769.7 to 1195.7)	47.6 (38 to 59.4)	962.2 (769.7 to 1195.7)	0.52 (0.36 to 0.68)
Italy	613.5 (478.2 to 799.5)	1202.3 (938.8 to 1534.9)	613.5 (478.2 to 799.5)	1202.3 (938.8 to 1534.9)	0.24 (0.01 to 0.46)
Jamaica	28.7 (24.8 to 33)	1214.4 (1042.1 to 1396.7)	28.7 (24.8 to 33)	1214.4 (1042.1 to 1396.7)	-1.90 (-2.08 to -1.72)
Japan	774.3 (627.9 to 965.2)	774.5 (656.3 to 924.1)	774.3 (627.9 to 965.2)	774.5 (656.3 to 924.1)	0.00 (-0.20 to 0.20)
Jordan	53.3 (45.5 to 62.4)	1137.6 (968.2 to 1326.1)	53.3 (45.5 to 62.4)	1137.6 (968.2 to 1326.1)	0.12 (0.01 to 0.23)
Kazakhstan	109.6 (90.9 to 133.1)	646.3 (534.5 to 783.7)	109.6 (90.9 to 133.1)	646.3 (534.5 to 783.7)	0.64 (0.55 to 0.73)
Kenya	538.3 (401.4 to 745.9)	1737.7 (1390.9 to 2211)	538.3 (401.4 to 745.9)	1737.7 (1390.9 to 2211)	-0.43 (-0.62 to -0.25)
Kiribati	1.7 (1.5 to 2)	1904.1 (1680 to 2143.6)	1.7 (1.5 to 2)	1904.1 (1680 to 2143.6)	-1.02 (-1.10 to -0.94)
Kuwait	18.5 (14.3 to 23.7)	991.3 (763.3 to 1274.6)	18.5 (14.3 to 23.7)	991.3 (763.3 to 1274.6)	0.44 (0.30 to 0.57)
Kyrgyzstan	29.5 (23.3 to 37.9)	549.3 (433.2 to 697.8)	29.5 (23.3 to 37.9)	549.3 (433.2 to 697.8)	0.05 (-0.23 to 0.34)
Lao People's Democratic Republic	162.4 (114 to 220.2)	2930.8 (2280.5 to 3681.9)	162.4 (114 to 220.2)	2930.8 (2280.5 to 3681.9)	-1.41 (-1.57 to -1.25)
Latvia	17 (13.9 to 21)	807.8 (650.6 to 1010.2)	17 (13.9 to 21)	807.8 (650.6 to 1010.2)	-0.03 (-0.13 to 0.07)
Lebanon	45.7 (39.4 to 53.1)	1112.9 (957.2 to 1286.5)	45.7 (39.4 to 53.1)	1112.9 (957.2 to 1286.5)	0.27 (0.11 to 0.44)
Lesotho	35.7 (30.7 to 41.6)	1659.9 (1443.1 to 1915.8)	35.7 (30.7 to 41.6)	1659.9 (1443.1 to 1915.8)	-1.46 (-1.73 to -1.19)
Liberia	35.1 (27.5 to 45.5)	1245.7 (1033.5 to 1510.5)	35.1 (27.5 to 45.5)	1245.7 (1033.5 to 1510.5)	-1.55 (-1.73 to -1.37)
Libya	64.9 (55.3 to 78)	1155.1 (977.8 to 1374.3)	64.9 (55.3 to 78)	1155.1 (977.8 to 1374.3)	0.25 (0.08 to 0.42)
Lithuania	24.6 (19.9 to 30.3)	805.3 (645.3 to 1000.5)	24.6 (19.9 to 30.3)	805.3 (645.3 to 1000.5)	-0.12 (-0.23 to -0.02)
Luxembourg	4.5 (3.7 to 5.5)	1138.4 (940.1 to 1374.2)	4.5 (3.7 to 5.5)	1138.4 (940.1 to 1374.2)	0.45 (0.35 to 0.56)
Madagascar	393.5 (324.6 to 489)	2344.9 (2031.8 to 2753.2)	393.5 (324.6 to 489)	2344.9 (2031.8 to 2753.2)	-1.50 (-1.58 to -1.42)
Malawi	258.4 (175.1 to 372)	1668.6 (1264.7 to 2196.5)	258.4 (175.1 to 372)	1668.6 (1264.7 to 2196.5)	-0.86 (-1.09 to -0.64)
Malaysia	448.4 (394.2 to 509.6)	2339.7 (2050.4 to 2667.7)	448.4 (394.2 to 509.6)	2339.7 (2050.4 to 2667.7)	-0.20 (-0.43 to 0.03)
Maldives	12.3 (9.8 to 15.1)	4292.4 (3565.3 to 5068.5)	12.3 (9.8 to 15.1)	4292.4 (3565.3 to 5068.5)	-0.17 (-0.39 to 0.05)
Mali	292.8 (254.5 to 344.9)	2020.9 (1788.8 to 2294.4)	292.8 (254.5 to 344.9)	2020.9 (1788.8 to 2294.4)	-0.75 (-0.90 to -0.60)
Malta	3.5 (2.8 to 4.5)	987.7 (791.2 to 1242.2)	3.5 (2.8 to 4.5)	987.7 (791.2 to 1242.2)	0.14 (0.00 to 0.29)
Marshall Islands	0.7 (0.6 to 0.8)	1280.8 (1102.3 to 1453.2)	0.7 (0.6 to 0.8)	1280.8 (1102.3 to 1453.2)	-0.62 (-0.75 to -0.50)
Mauritania	51 (43.5 to 61.1)	1685.6 (1475.1 to 1947)	51 (43.5 to 61.1)	1685.6 (1475.1 to 1947)	-1.20 (-1.38 to -1.01)
Mauritius	24.4 (21.6 to 27.7)	2313.7 (2058.5 to 2619.1)	24.4 (21.6 to 27.7)	2313.7 (2058.5 to 2619.1)	-0.64 (-0.80 to -0.49)
Mexico	1517.1 (1231.1 to 1847.9)	1875.8 (1538.7 to 2257.9)	1517.1 (1231.1 to 1847.9)	1875.8 (1538.7 to 2257.9)	-1.00 (-1.32 to -0.67)

Continued on next page

Table S16 – continued from previous page

Country	1990		2019		1990–2019
	Incident cases No.×10⁴[95% UI]	ASR per 10,0000 No.[95% UI]	Incident cases No.×10⁴[95% UI]	ASR per 10,0000 No.[95% UI]	EAPC No.[95% CI]
Micronesia (Federated States of)	1.5 (1.3 to 1.8)	1212.1 (1052.5 to 1394.1)	1.5 (1.3 to 1.8)	1212.1 (1052.5 to 1394.1)	-0.54 (-0.69 to -0.40)
Monaco	0.3 (0.2 to 0.4)	976.1 (782.3 to 1205)	0.3 (0.2 to 0.4)	976.1 (782.3 to 1205)	0.06 (-0.07 to 0.20)
Mongolia	14.5 (12.1 to 17.5)	484.5 (402.4 to 588.6)	14.5 (12.1 to 17.5)	484.5 (402.4 to 588.6)	0.16 (-0.04 to 0.37)
Montenegro	5.1 (4 to 6.5)	908.6 (703.3 to 1176.7)	5.1 (4 to 6.5)	908.6 (703.3 to 1176.7)	1.05 (0.89 to 1.20)
Morocco	318.4 (267.3 to 379.6)	1024.4 (872.1 to 1207.3)	318.4 (267.3 to 379.6)	1024.4 (872.1 to 1207.3)	0.10 (-0.12 to 0.31)
Mozambique	259.4 (222.1 to 310.2)	1301.3 (1146.9 to 1510.4)	259.4 (222.1 to 310.2)	1301.3 (1146.9 to 1510.4)	-2.55 (-2.76 to -2.34)
Myanmar	923.7 (769.9 to 1114.2)	1931 (1654.1 to 2242.6)	923.7 (769.9 to 1114.2)	1931 (1654.1 to 2242.6)	-0.53 (-0.72 to -0.35)
Namibia	26.1 (21.8 to 31.4)	1464.1 (1257.1 to 1695.5)	26.1 (21.8 to 31.4)	1464.1 (1257.1 to 1695.5)	-0.59 (-0.86 to -0.31)
Nauru	0.2 (0.1 to 0.2)	1297.9 (1128.4 to 1486.5)	0.2 (0.1 to 0.2)	1297.9 (1128.4 to 1486.5)	-0.32 (-0.45 to -0.20)
Nepal	725.8 (582.2 to 926.7)	2865 (2427.1 to 3410.4)	725.8 (582.2 to 926.7)	2865 (2427.1 to 3410.4)	-0.45 (-0.54 to -0.35)
Netherlands	158.4 (127.9 to 197.2)	1052.4 (857.4 to 1309)	158.4 (127.9 to 197.2)	1052.4 (857.4 to 1309)	0.32 (0.11 to 0.53)
New Zealand	21 (17.4 to 25.3)	650.1 (546.1 to 776.9)	21 (17.4 to 25.3)	650.1 (546.1 to 776.9)	-0.10 (-0.25 to 0.04)
Nicaragua	39.1 (34.3 to 45.2)	809.6 (712.1 to 923.5)	39.1 (34.3 to 45.2)	809.6 (712.1 to 923.5)	-1.60 (-1.85 to -1.35)
Niger	291.5 (252.8 to 350.1)	1838.5 (1635.6 to 2121.8)	291.5 (252.8 to 350.1)	1838.5 (1635.6 to 2121.8)	-1.10 (-1.24 to -0.96)
Nigeria	2456.8 (1843.3 to 3271.8)	1777.7 (1423.4 to 2212.8)	2456.8 (1843.3 to 3271.8)	1777.7 (1423.4 to 2212.8)	-0.37 (-0.65 to -0.10)
Niue	0 (0 to 0)	1426.8 (1245.8 to 1611.1)	0 (0 to 0)	1426.8 (1245.8 to 1611.1)	-0.02 (-0.12 to 0.08)
North Macedonia	17.9 (13.8 to 23.2)	965.4 (732.7 to 1254.3)	17.9 (13.8 to 23.2)	965.4 (732.7 to 1254.3)	0.79 (0.64 to 0.93)
Northern Mariana Islands	0.6 (0.5 to 0.7)	1354.5 (1192.9 to 1532.5)	0.6 (0.5 to 0.7)	1354.5 (1192.9 to 1532.5)	0.21 (0.05 to 0.37)
Norway	45.8 (36.1 to 58.4)	1140.6 (897.9 to 1452.1)	45.8 (36.1 to 58.4)	1140.6 (897.9 to 1452.1)	0.87 (0.74 to 1.00)
Oman	40.1 (35 to 46.6)	1553.4 (1345.8 to 1796.9)	40.1 (35 to 46.6)	1553.4 (1345.8 to 1796.9)	-0.08 (-0.21 to 0.05)
Pakistan	4066.2 (3266.3 to 5105.9)	2452.2 (2024.7 to 2951.1)	4066.2 (3266.3 to 5105.9)	2452.2 (2024.7 to 2951.1)	-1.03 (-1.24 to -0.83)
Palau	0.2 (0.2 to 0.2)	1274.2 (1111.2 to 1455.5)	0.2 (0.2 to 0.2)	1274.2 (1111.2 to 1455.5)	-0.08 (-0.24 to 0.07)
Palestine	27.9 (24.2 to 32.3)	959.9 (830.1 to 1109.3)	27.9 (24.2 to 32.3)	959.9 (830.1 to 1109.3)	0.14 (-0.03 to 0.30)
Panama	18.2 (16 to 20.6)	722.1 (628.7 to 822.4)	18.2 (16 to 20.6)	722.1 (628.7 to 822.4)	-0.91 (-1.16 to -0.67)
Papua New Guinea	107.6 (85.6 to 137.7)	1956 (1608.1 to 2409.1)	107.6 (85.6 to 137.7)	1956 (1608.1 to 2409.1)	-0.56 (-0.87 to -0.25)
Paraguay	30.6 (25.4 to 36.5)	766.7 (651.6 to 889.1)	30.6 (25.4 to 36.5)	766.7 (651.6 to 889.1)	1.01 (0.86 to 1.17)
Peru	132.5 (112.3 to 156)	627.4 (536.7 to 725)	132.5 (112.3 to 156)	627.4 (536.7 to 725)	-1.56 (-1.75 to -1.38)
Philippines	1623.2 (1237.2 to 2120.9)	2174 (1721.6 to 2720.8)	1623.2 (1237.2 to 2120.9)	2174 (1721.6 to 2720.8)	-1.10 (-1.35 to -0.86)
Poland	294.6 (226.3 to 374.5)	907.8 (697.6 to 1176)	294.6 (226.3 to 374.5)	907.8 (697.6 to 1176)	0.44 (0.23 to 0.64)
Portugal	125.3 (101 to 153.4)	1278 (1051.8 to 1553.8)	125.3 (101 to 153.4)	1278 (1051.8 to 1553.8)	0.17 (0.03 to 0.32)

Continued on next page

Table S16 – continued from previous page

Country	1990		2019		1990–2019
	Incident cases No.×10^4[95% UI]	ASR per 10,0000 No.[95% UI]	Incident cases No.×10^4[95% UI]	ASR per 10,0000 No.[95% UI]	EAPC No.[95% CI]
Puerto Rico	36.2 (29.7 to 43.9)	1050 (860.9 to 1285.8)	36.2 (29.7 to 43.9)	1050 (860.9 to 1285.8)	0.51 (0.34 to 0.67)
Qatar	5.5 (4.5 to 6.6)	1143.3 (943.4 to 1380.2)	5.5 (4.5 to 6.6)	1143.3 (943.4 to 1380.2)	0.54 (0.43 to 0.65)
Republic of Korea	320.2 (267.9 to 377.6)	794.6 (670.7 to 925.3)	320.2 (267.9 to 377.6)	794.6 (670.7 to 925.3)	-1.55 (-1.84 to -1.26)
Republic of Moldova	29.5 (23.4 to 37.7)	712.5 (558 to 928)	29.5 (23.4 to 37.7)	712.5 (558 to 928)	0.58 (0.49 to 0.67)
Romania	164.5 (129.5 to 207.2)	859.7 (666.7 to 1095.5)	164.5 (129.5 to 207.2)	859.7 (666.7 to 1095.5)	0.46 (0.32 to 0.60)
Russian Federation	1026.4 (830.2 to 1245.3)	834.2 (672.4 to 1026.6)	1026.4 (830.2 to 1245.3)	834.2 (672.4 to 1026.6)	0.17 (0.02 to 0.32)
Rwanda	127 (107.6 to 153.2)	1302.5 (1141.4 to 1496)	127 (107.6 to 153.2)	1302.5 (1141.4 to 1496)	-1.76 (-1.99 to -1.52)
Saint Kitts and Nevis	0.4 (0.4 to 0.5)	1112.3 (952.1 to 1293.6)	0.4 (0.4 to 0.5)	1112.3 (952.1 to 1293.6)	-0.23 (-0.34 to -0.13)
Saint Lucia	1.4 (1.2 to 1.7)	1045.6 (902.3 to 1209.6)	1.4 (1.2 to 1.7)	1045.6 (902.3 to 1209.6)	-0.67 (-0.80 to -0.53)
Saint Vincent and the Grenadines	1.5 (1.2 to 1.7)	1378.4 (1169.3 to 1623)	1.5 (1.2 to 1.7)	1378.4 (1169.3 to 1623)	-0.97 (-1.10 to -0.84)
Samoa	1.5 (1.2 to 1.7)	1006.8 (848.5 to 1178.2)	1.5 (1.2 to 1.7)	1006.8 (848.5 to 1178.2)	-0.08 (-0.23 to 0.06)
San Marino	0.2 (0.2 to 0.3)	1006.5 (809.2 to 1245.4)	0.2 (0.2 to 0.3)	1006.5 (809.2 to 1245.4)	0.13 (-0.01 to 0.27)
Sao Tome and Principe	2.1 (1.8 to 2.6)	1273.5 (1082.1 to 1504.7)	2.1 (1.8 to 2.6)	1273.5 (1082.1 to 1504.7)	-1.34 (-1.54 to -1.14)
Saudi Arabia	258.1 (215 to 309.5)	1340.3 (1129.7 to 1579.6)	258.1 (215 to 309.5)	1340.3 (1129.7 to 1579.6)	-0.16 (-0.28 to -0.03)
Senegal	150 (129.6 to 176.6)	1280.6 (1124.9 to 1461.1)	150 (129.6 to 176.6)	1280.6 (1124.9 to 1461.1)	-0.84 (-0.91 to -0.76)
Serbia	70.7 (55.9 to 89.9)	910.3 (710.4 to 1165.6)	70.7 (55.9 to 89.9)	910.3 (710.4 to 1165.6)	0.48 (0.38 to 0.58)
Seychelles	1.1 (0.9 to 1.2)	1514.3 (1321.3 to 1705.4)	1.1 (0.9 to 1.2)	1514.3 (1321.3 to 1705.4)	0.11 (0.03 to 0.18)
Sierra Leone	90.3 (71.1 to 116.5)	1591.3 (1317.8 to 1947.3)	90.3 (71.1 to 116.5)	1591.3 (1317.8 to 1947.3)	-1.20 (-1.31 to -1.08)
Singapore	22.1 (18.9 to 26)	869.3 (761 to 998.9)	22.1 (18.9 to 26)	869.3 (761 to 998.9)	-0.04 (-0.13 to 0.05)
Slovakia	36.2 (28.2 to 46.5)	795.5 (616.1 to 1038.9)	36.2 (28.2 to 46.5)	795.5 (616.1 to 1038.9)	0.47 (0.41 to 0.53)
Slovenia	12.8 (9.8 to 16.2)	826.3 (627.2 to 1081.4)	12.8 (9.8 to 16.2)	826.3 (627.2 to 1081.4)	0.30 (0.24 to 0.37)
Solomon Islands	6.7 (5.3 to 8.6)	1497.1 (1246.1 to 1788.3)	6.7 (5.3 to 8.6)	1497.1 (1246.1 to 1788.3)	-0.83 (-0.95 to -0.71)
Somalia	244.5 (203.2 to 298.6)	2318 (2003.5 to 2703.8)	244.5 (203.2 to 298.6)	2318 (2003.5 to 2703.8)	-1.89 (-2.13 to -1.64)
South Africa	421 (340.2 to 532.9)	1046.1 (857.2 to 1310.6)	421 (340.2 to 532.9)	1046.1 (857.2 to 1310.6)	-1.04 (-1.26 to -0.83)
South Sudan	182 (154.3 to 220.1)	2302.6 (2010.2 to 2655.9)	182 (154.3 to 220.1)	2302.6 (2010.2 to 2655.9)	-1.52 (-1.70 to -1.34)
Spain	433.8 (340.9 to 550.2)	1141 (906.8 to 1445)	433.8 (340.9 to 550.2)	1141 (906.8 to 1445)	0.25 (0.10 to 0.40)
Sri Lanka	675.8 (585.4 to 772)	4078.1 (3519.8 to 4654.5)	675.8 (585.4 to 772)	4078.1 (3519.8 to 4654.5)	0.08 (-0.30 to 0.46)
Sudan	841.5 (645.8 to 1104.5)	2397.3 (1943.4 to 2970.4)	841.5 (645.8 to 1104.5)	2397.3 (1943.4 to 2970.4)	-0.75 (-1.16 to -0.33)

Continued on next page

Table S16 – continued from previous page

Country	1990		2019		1990–2019
	Incident cases No.$\times 10^4$[95% UI]	ASR per 10,0000 No.[95% UI]	Incident cases No.$\times 10^4$[95% UI]	ASR per 10,0000 No.[95% UI]	EAPC No.[95% CI]
Suriname	4.4 (3.7 to 5.3)	1130.2 (955.8 to 1336.6)	4.4 (3.7 to 5.3)	1130.2 (955.8 to 1336.6)	-0.64 (-0.78 to -0.50)
Sweden	95.2 (75.3 to 119.4)	1148.8 (910.3 to 1442.1)	95.2 (75.3 to 119.4)	1148.8 (910.3 to 1442.1)	0.23 (0.02 to 0.43)
Switzerland	66 (50.7 to 84.7)	983.5 (768.9 to 1261.9)	66 (50.7 to 84.7)	983.5 (768.9 to 1261.9)	0.60 (0.49 to 0.71)
Syrian Arab Republic	285.3 (248 to 327.1)	1426.1 (1228.5 to 1633.2)	285.3 (248 to 327.1)	1426.1 (1228.5 to 1633.2)	0.06 (-0.12 to 0.24)
Taiwan (Province of China)	209.5 (174.8 to 249.3)	1123 (934.6 to 1344.7)	209.5 (174.8 to 249.3)	1123 (934.6 to 1344.7)	-0.22 (-0.44 to 0.01)
Tajikistan	78.9 (67.7 to 94.5)	920.4 (783.8 to 1098)	78.9 (67.7 to 94.5)	920.4 (783.8 to 1098)	-0.09 (-0.19 to 0.02)
Thailand	835.9 (732 to 952.8)	1596.4 (1400.1 to 1813)	835.9 (732 to 952.8)	1596.4 (1400.1 to 1813)	-0.19 (-0.27 to -0.11)
Timor-Leste	42.2 (31.7 to 56.4)	3751 (3052 to 4643)	42.2 (31.7 to 56.4)	3751 (3052 to 4643)	-1.30 (-1.44 to -1.16)
Togo	78.7 (63 to 100.3)	1413.8 (1185 to 1696.8)	78.7 (63 to 100.3)	1413.8 (1185 to 1696.8)	-0.91 (-1.06 to -0.75)
Tokelau	0 (0 to 0)	1330.6 (1163.2 to 1516.2)	0 (0 to 0)	1330.6 (1163.2 to 1516.2)	-0.12 (-0.21 to -0.02)
Tonga	1.3 (1.1 to 1.4)	1300.1 (1137.2 to 1474.2)	1.3 (1.1 to 1.4)	1300.1 (1137.2 to 1474.2)	-0.31 (-0.45 to -0.16)
Trinidad and Tobago	11.6 (10.1 to 13.3)	1014.8 (879.9 to 1166.8)	11.6 (10.1 to 13.3)	1014.8 (879.9 to 1166.8)	-0.71 (-0.86 to -0.57)
Tunisia	116 (95.4 to 141.9)	1196.3 (988.7 to 1450.4)	116 (95.4 to 141.9)	1196.3 (988.7 to 1450.4)	0.05 (-0.08 to 0.18)
Turkey	513.7 (426.9 to 625.5)	744.5 (622.5 to 894.9)	513.7 (426.9 to 625.5)	744.5 (622.5 to 894.9)	1.83 (1.55 to 2.10)
Turkmenistan	44.5 (36.2 to 57.1)	812.4 (664.6 to 1027.4)	44.5 (36.2 to 57.1)	812.4 (664.6 to 1027.4)	-0.20 (-0.31 to -0.08)
Tuvalu	0.2 (0.1 to 0.2)	1414.3 (1198.2 to 1671.9)	0.2 (0.1 to 0.2)	1414.3 (1198.2 to 1671.9)	-0.67 (-0.78 to -0.57)
Uganda	308.5 (238.6 to 397.4)	1089.3 (900.4 to 1309.4)	308.5 (238.6 to 397.4)	1089.3 (900.4 to 1309.4)	-1.45 (-1.55 to -1.36)
Ukraine	401.9 (333.4 to 487.6)	985.8 (816.7 to 1204.5)	401.9 (333.4 to 487.6)	985.8 (816.7 to 1204.5)	0.33 (0.17 to 0.49)
United Arab Emirates	29.2 (25.6 to 33.8)	1402.2 (1226 to 1615.5)	29.2 (25.6 to 33.8)	1402.2 (1226 to 1615.5)	0.26 (0.15 to 0.37)
United Kingdom	677.1 (531.4 to 867.5)	1259.2 (988.9 to 1601.3)	677.1 (531.4 to 867.5)	1259.2 (988.9 to 1601.3)	-0.08 (-0.38 to 0.23)
United Republic of Tanzania	448.4 (368.8 to 552.1)	1124.4 (971.6 to 1309.8)	448.4 (368.8 to 552.1)	1124.4 (971.6 to 1309.8)	-1.74 (-1.88 to -1.60)
United States of America	1727.3 (1348 to 2170.6)	669.7 (524.9 to 844.3)	1727.3 (1348 to 2170.6)	669.7 (524.9 to 844.3)	-0.08 (-0.28 to 0.11)
United States Virgin Islands	1 (0.9 to 1.2)	994.5 (855.6 to 1139.4)	1 (0.9 to 1.2)	994.5 (855.6 to 1139.4)	-0.23 (-0.36 to -0.10)
Uruguay	20.4 (17.2 to 24.3)	663.2 (561.6 to 788.3)	20.4 (17.2 to 24.3)	663.2 (561.6 to 788.3)	0.19 (0.00 to 0.39)
Uzbekistan	274.3 (235.6 to 317.8)	893.3 (766.7 to 1033.3)	274.3 (235.6 to 317.8)	893.3 (766.7 to 1033.3)	-0.25 (-0.36 to -0.15)
Vanuatu	2.2 (1.9 to 2.7)	1213.6 (1047.4 to 1407.5)	2.2 (1.9 to 2.7)	1213.6 (1047.4 to 1407.5)	-0.28 (-0.40 to -0.15)

Continued on next page

Table S16 – continued from previous page

Country	1990		2019		1990–2019
	Incident cases No.×10^4[95% UI]	ASR per 10,0000 No.[95% UI]	Incident cases No.×10^4[95% UI]	ASR per 10,0000 No.[95% UI]	EAPC No.[95% CI]
Venezuela (Bolivarian Republic of)	209.9 (180.4 to 242.9)	1028.7 (880.3 to 1198.3)	209.9 (180.4 to 242.9)	1028.7 (880.3 to 1198.3)	-0.41 (-0.68 to -0.14)
Viet Nam	1603.3 (1395.2 to 1849.1)	2085.1 (1816 to 2422.9)	1603.3 (1395.2 to 1849.1)	2085.1 (1816 to 2422.9)	-0.13 (-0.31 to 0.06)
Yemen	738.2 (557.2 to 990.6)	3342.5 (2646.6 to 4185.5)	738.2 (557.2 to 990.6)	3342.5 (2646.6 to 4185.5)	-0.96 (-1.07 to -0.85)
Zambia	140.9 (105.2 to 187.1)	1184.5 (971.3 to 1447.8)	140.9 (105.2 to 187.1)	1184.5 (971.3 to 1447.8)	-0.65 (-0.79 to -0.51)
Zimbabwe	162.5 (133.8 to 196.7)	1415.7 (1208.8 to 1637.4)	162.5 (133.8 to 196.7)	1415.7 (1208.8 to 1637.4)	0.02 (-0.13 to 0.17)

Abbreviation: ASR, age standardized rate; CI, confidence interval; EAPC, estimated annual percentage change; UI, uncertainty interval.

Table S17: The change of protein-energy malnutrition cases between 1990 and 2019 at national level for female.

Country	1990		2019		1990–2019
	Incident cases No.×10^4[95% UI]	ASR per 10,0000 No.[95% UI]	Incident cases No.×10^4[95% UI]	ASR per 10,0000 No.[95% UI]	EAPC No.[95% CI]
Afghanistan	102.8 (79.2 to 133.6)	1097.1 (886.4 to 1360.3)	102.8 (79.2 to 133.6)	1097.1 (886.4 to 1360.3)	-0.97 (-1.16 to -0.77)
Albania	41.4 (29.6 to 55.6)	2248.9 (1634.7 to 2986.8)	41.4 (29.6 to 55.6)	2248.9 (1634.7 to 2986.8)	-0.34 (-0.67 to -0.01)
Algeria	199.1 (162.3 to 242.7)	1315.6 (1084.5 to 1574)	199.1 (162.3 to 242.7)	1315.6 (1084.5 to 1574)	-0.11 (-0.35 to 0.12)
American Samoa	0.4 (0.3 to 0.5)	1614.2 (1396.7 to 1847.6)	0.4 (0.3 to 0.5)	1614.2 (1396.7 to 1847.6)	-0.35 (-0.44 to -0.25)
Andorra	0.2 (0.2 to 0.3)	860.9 (668.3 to 1092.5)	0.2 (0.2 to 0.3)	860.9 (668.3 to 1092.5)	0.20 (0.04 to 0.37)
Angola	123.8 (87.2 to 185.8)	1426.6 (1102.9 to 1948)	123.8 (87.2 to 185.8)	1426.6 (1102.9 to 1948)	-1.79 (-2.01 to -1.58)
Antigua and Barbuda	0.3 (0.3 to 0.4)	1090.5 (932.2 to 1295.1)	0.3 (0.3 to 0.4)	1090.5 (932.2 to 1295.1)	-0.47 (-0.61 to -0.33)
Argentina	120.5 (98.7 to 145.6)	715.8 (587.3 to 865.5)	120.5 (98.7 to 145.6)	715.8 (587.3 to 865.5)	0.17 (-0.08 to 0.42)
Armenia	12.4 (9.9 to 15.5)	708.4 (563.2 to 889.3)	12.4 (9.9 to 15.5)	708.4 (563.2 to 889.3)	0.30 (0.19 to 0.41)
Australia	40.9 (32.5 to 50.9)	525.7 (428.4 to 641.6)	40.9 (32.5 to 50.9)	525.7 (428.4 to 641.6)	0.61 (0.45 to 0.77)
Austria	37.4 (28.2 to 48.8)	937.1 (717.5 to 1202.6)	37.4 (28.2 to 48.8)	937.1 (717.5 to 1202.6)	-0.01 (-0.16 to 0.14)
Azerbaijan	33.4 (25.5 to 43.4)	792.1 (607.5 to 1015.8)	33.4 (25.5 to 43.4)	792.1 (607.5 to 1015.8)	-0.55 (-0.73 to -0.38)
Bahamas	2.2 (1.7 to 2.7)	1722.2 (1401 to 2139.2)	2.2 (1.7 to 2.7)	1722.2 (1401 to 2139.2)	-0.18 (-0.52 to 0.16)
Bahrain	3.4 (2.9 to 3.9)	1493.4 (1304.1 to 1700.4)	3.4 (2.9 to 3.9)	1493.4 (1304.1 to 1700.4)	0.11 (-0.03 to 0.24)
Bangladesh	1828.1 (1599 to 2082.9)	2405.2 (2138.9 to 2704.8)	1828.1 (1599 to 2082.9)	2405.2 (2138.9 to 2704.8)	-1.98 (-2.44 to -1.52)
Barbados	1.1 (1 to 1.3)	916.7 (784.7 to 1066.3)	1.1 (1 to 1.3)	916.7 (784.7 to 1066.3)	-0.15 (-0.34 to 0.04)

Continued on next page

Table S17 – continued from previous page

Country	1990		2019		1990–2019
	Incident cases No.×10^4[95% UI]	ASR per 10,0000 No.[95% UI]	Incident cases No.×10^4[95% UI]	ASR per 10,0000 No.[95% UI]	EAPC No.[95% CI]
Belarus	30.1 (22.3 to 39.3)	750.2 (531.8 to 1008)	30.1 (22.3 to 39.3)	750.2 (531.8 to 1008)	-0.61 (-0.73 to -0.48)
Belgium	57.8 (46.8 to 71.2)	1058 (868.5 to 1301.2)	57.8 (46.8 to 71.2)	1058 (868.5 to 1301.2)	0.79 (0.59 to 0.99)
Belize	1.1 (0.8 to 1.5)	1112.8 (879.4 to 1372.6)	1.1 (0.8 to 1.5)	1112.8 (879.4 to 1372.6)	-1.58 (-1.74 to -1.42)
Benin	61.6 (51.1 to 77.1)	1492.2 (1281.8 to 1779.9)	61.6 (51.1 to 77.1)	1492.2 (1281.8 to 1779.9)	-1.01 (-1.13 to -0.90)
Bermuda	0.3 (0.2 to 0.3)	943.3 (779.5 to 1155.8)	0.3 (0.2 to 0.3)	943.3 (779.5 to 1155.8)	0.57 (0.37 to 0.77)
Bhutan	7.6 (5.5 to 10.4)	1921.2 (1475 to 2501.5)	7.6 (5.5 to 10.4)	1921.2 (1475 to 2501.5)	-1.06 (-1.27 to -0.85)
Bolivia (Plurinational State of)	30.9 (27 to 36)	836.8 (735.3 to 955.5)	30.9 (27 to 36)	836.8 (735.3 to 955.5)	-1.47 (-1.60 to -1.35)
Bosnia and Herzegovina	16.4 (12.3 to 21.8)	852.4 (619.8 to 1165.2)	16.4 (12.3 to 21.8)	852.4 (619.8 to 1165.2)	0.38 (0.28 to 0.48)
Botswana	7.5 (6.3 to 9)	942.9 (805.3 to 1109.6)	7.5 (6.3 to 9)	942.9 (805.3 to 1109.6)	0.35 (0.07 to 0.63)
Brazil	610.7 (482 to 774)	820.2 (651.6 to 1029.9)	610.7 (482 to 774)	820.2 (651.6 to 1029.9)	-1.30 (-1.55 to -1.05)
Brunei Darussalam	1.4 (1.2 to 1.5)	1001.6 (896.3 to 1119.8)	1.4 (1.2 to 1.5)	1001.6 (896.3 to 1119.8)	-0.24 (-0.33 to -0.15)
Bulgaria	26.8 (20.1 to 34.8)	825.2 (614.2 to 1100.3)	26.8 (20.1 to 34.8)	825.2 (614.2 to 1100.3)	0.42 (0.34 to 0.51)
Burkina Faso	126.5 (109.9 to 149.5)	1551.3 (1375.8 to 1784.6)	126.5 (109.9 to 149.5)	1551.3 (1375.8 to 1784.6)	-0.82 (-1.21 to -0.43)
Burundi	69.2 (57.4 to 85.7)	1522.1 (1297.2 to 1796.8)	69.2 (57.4 to 85.7)	1522.1 (1297.2 to 1796.8)	-0.97 (-1.11 to -0.83)
Cabo Verde	1.7 (1.4 to 2.1)	755.7 (625.3 to 905.8)	1.7 (1.4 to 2.1)	755.7 (625.3 to 905.8)	-0.46 (-0.81 to -0.10)
Cambodia	160.7 (117.5 to 220.9)	2155.1 (1696.7 to 2756.8)	160.7 (117.5 to 220.9)	2155.1 (1696.7 to 2756.8)	-1.26 (-1.51 to -1.01)
Cameroon	73.5 (59 to 91.8)	942.8 (794.5 to 1129.2)	73.5 (59 to 91.8)	942.8 (794.5 to 1129.2)	-0.69 (-0.83 to -0.55)
Canada	92.7 (73.7 to 114.5)	651.8 (522.2 to 800.7)	92.7 (73.7 to 114.5)	651.8 (522.2 to 800.7)	-0.04 (-0.27 to 0.18)
Central African Republic	27.8 (20.5 to 38)	1220.9 (964.3 to 1560.8)	27.8 (20.5 to 38)	1220.9 (964.3 to 1560.8)	-0.30 (-0.41 to -0.19)
Chad	104.7 (87.3 to 131.4)	1857.6 (1601.2 to 2217.7)	104.7 (87.3 to 131.4)	1857.6 (1601.2 to 2217.7)	-0.77 (-0.95 to -0.58)
Chile	31 (25.7 to 37.1)	471.9 (389.8 to 565)	31 (25.7 to 37.1)	471.9 (389.8 to 565)	0.58 (0.17 to 0.99)
China	8733.6 (6439.3 to 11759.6)	1593.3 (1175.8 to 2147.9)	8733.6 (6439.3 to 11759.6)	1593.3 (1175.8 to 2147.9)	-0.34 (-0.61 to -0.06)
Colombia	130.7 (111.8 to 152)	788.9 (674.4 to 910.4)	130.7 (111.8 to 152)	788.9 (674.4 to 910.4)	-0.73 (-0.97 to -0.48)
Comoros	5.4 (4.3 to 6.6)	1600.6 (1348.3 to 1880.5)	5.4 (4.3 to 6.6)	1600.6 (1348.3 to 1880.5)	-0.94 (-1.06 to -0.82)
Congo	14.9 (11.2 to 19.9)	849.1 (681.3 to 1059.4)	14.9 (11.2 to 19.9)	849.1 (681.3 to 1059.4)	-0.57 (-0.77 to -0.37)
Cook Islands	0.1 (0.1 to 0.2)	1525.2 (1307.8 to 1764.2)	0.1 (0.1 to 0.2)	1525.2 (1307.8 to 1764.2)	0.13 (0.01 to 0.24)
Costa Rica	12.2 (10.1 to 14.9)	768.8 (625.2 to 948.6)	12.2 (10.1 to 14.9)	768.8 (625.2 to 948.6)	-0.01 (-0.23 to 0.21)
Croatia	12 (9.3 to 15.4)	625.7 (492.9 to 793.8)	12 (9.3 to 15.4)	625.7 (492.9 to 793.8)	0.06 (-0.05 to 0.16)
Cuba	35 (28.3 to 44.3)	697.1 (565.8 to 872.8)	35 (28.3 to 44.3)	697.1 (565.8 to 872.8)	0.54 (0.28 to 0.79)
Cyprus	4.2 (3.4 to 5.2)	1084.9 (874.6 to 1334.2)	4.2 (3.4 to 5.2)	1084.9 (874.6 to 1334.2)	0.36 (0.30 to 0.43)

Continued on next page

Table S17 – continued from previous page

Country	1990		2019		1990–2019
	Incident cases No.×10^4[95% UI]	ASR per 10,0000 No.[95% UI]	Incident cases No.×10^4[95% UI]	ASR per 10,0000 No.[95% UI]	EAPC No.[95% CI]
Czechia	24.3 (18.6 to 31.3)	598.1 (455.8 to 779.6)	24.3 (18.6 to 31.3)	598.1 (455.8 to 779.6)	1.88 (1.54 to 2.22)
Ivoirian	103 (83.6 to 129.7)	1066.2 (893.8 to 1269.3)	103 (83.6 to 129.7)	1066.2 (893.8 to 1269.3)	-0.75 (-0.84 to -0.67)
Democratic People's Republic of Korea	232.1 (170.5 to 313.9)	1685 (1300.6 to 2192.3)	232.1 (170.5 to 313.9)	1685 (1300.6 to 2192.3)	-0.81 (-1.08 to -0.54)
Democratic Republic of the Congo	647.9 (442.7 to 926.7)	1875.7 (1386.6 to 2505.1)	647.9 (442.7 to 926.7)	1875.7 (1386.6 to 2505.1)	-0.95 (-1.29 to -0.60)
Denmark	22.9 (17.5 to 29.9)	891.2 (687.5 to 1147)	22.9 (17.5 to 29.9)	891.2 (687.5 to 1147)	0.60 (0.25 to 0.94)
Djibouti	7.9 (6.8 to 9.3)	2245 (1980.4 to 2564.4)	7.9 (6.8 to 9.3)	2245 (1980.4 to 2564.4)	-1.24 (-1.34 to -1.14)
Dominica	0.4 (0.3 to 0.5)	1079.8 (909.4 to 1276.8)	0.4 (0.3 to 0.5)	1079.8 (909.4 to 1276.8)	-0.37 (-0.52 to -0.22)
Dominican Republic	31.7 (25.4 to 38.7)	768.5 (641.4 to 910.9)	31.7 (25.4 to 38.7)	768.5 (641.4 to 910.9)	-1.65 (-1.81 to -1.50)
Ecuador	41.2 (36.3 to 46.9)	859.5 (753 to 982.5)	41.2 (36.3 to 46.9)	859.5 (753 to 982.5)	-1.20 (-1.43 to -0.96)
Egypt	397.6 (318.5 to 501.2)	1134.2 (931.1 to 1396.3)	397.6 (318.5 to 501.2)	1134.2 (931.1 to 1396.3)	-0.27 (-0.46 to -0.07)
El Salvador	25.5 (21.1 to 30.5)	928.5 (793.2 to 1082.5)	25.5 (21.1 to 30.5)	928.5 (793.2 to 1082.5)	-0.07 (-0.22 to 0.09)
Equatorial Guinea	5.6 (5 to 6.4)	1369.4 (1244.4 to 1528.5)	5.6 (5 to 6.4)	1369.4 (1244.4 to 1528.5)	-2.55 (-3.09 to -2.01)
Eritrea	47.6 (39.6 to 59.5)	2065.6 (1761.5 to 2469.5)	47.6 (39.6 to 59.5)	2065.6 (1761.5 to 2469.5)	-1.92 (-2.04 to -1.80)
Estonia	5.7 (4.7 to 7)	901.7 (715.2 to 1134.1)	5.7 (4.7 to 7)	901.7 (715.2 to 1134.1)	-0.24 (-0.54 to 0.06)
Eswatini	4 (2.9 to 5.4)	753.7 (596.7 to 948.6)	4 (2.9 to 5.4)	753.7 (596.7 to 948.6)	-0.93 (-1.01 to -0.85)
Ethiopia	664 (516.5 to 865.7)	1662 (1378.8 to 2025.7)	664 (516.5 to 865.7)	1662 (1378.8 to 2025.7)	-1.16 (-1.33 to -0.99)
Fiji	4.9 (4.3 to 5.5)	1324.6 (1150.4 to 1497.4)	4.9 (4.3 to 5.5)	1324.6 (1150.4 to 1497.4)	-0.36 (-0.51 to -0.22)
Finland	22.4 (16.9 to 28.9)	902 (690.1 to 1153.3)	22.4 (16.9 to 28.9)	902 (690.1 to 1153.3)	-0.13 (-0.30 to 0.03)
France	498.2 (415.7 to 589.2)	1503.8 (1254.9 to 1777.1)	498.2 (415.7 to 589.2)	1503.8 (1254.9 to 1777.1)	0.02 (-0.10 to 0.13)
Gabon	5.9 (4.4 to 7.9)	870.6 (702.9 to 1096.5)	5.9 (4.4 to 7.9)	870.6 (702.9 to 1096.5)	-1.08 (-1.26 to -0.90)
Gambia	11.5 (9.8 to 13.8)	1444.1 (1263.2 to 1675.8)	11.5 (9.8 to 13.8)	1444.1 (1263.2 to 1675.8)	-1.19 (-1.36 to -1.02)
Georgia	12.7 (9.3 to 16.9)	540.8 (395.4 to 734.2)	12.7 (9.3 to 16.9)	540.8 (395.4 to 734.2)	0.05 (-0.15 to 0.25)
Germany	305.4 (226.8 to 399.7)	745.1 (554.7 to 981.7)	305.4 (226.8 to 399.7)	745.1 (554.7 to 981.7)	0.58 (0.41 to 0.76)
Ghana	149.5 (123 to 187.8)	1370.9 (1159.5 to 1659.5)	149.5 (123 to 187.8)	1370.9 (1159.5 to 1659.5)	-1.57 (-1.81 to -1.34)
Greece	22.5 (17.2 to 28.9)	478.6 (372.6 to 608.5)	22.5 (17.2 to 28.9)	478.6 (372.6 to 608.5)	-0.60 (-0.80 to -0.39)
Greenland	0.1 (0.1 to 0.1)	495.6 (414.1 to 599.9)	0.1 (0.1 to 0.1)	495.6 (414.1 to 599.9)	0.46 (0.08 to 0.84)
Grenada	0.4 (0.3 to 0.5)	873.5 (729.6 to 1039.7)	0.4 (0.3 to 0.5)	873.5 (729.6 to 1039.7)	-0.24 (-0.41 to -0.08)
Guam	1 (0.8 to 1.1)	1456.3 (1225.4 to 1709.3)	1 (0.8 to 1.1)	1456.3 (1225.4 to 1709.3)	-0.72 (-0.96 to -0.49)

Continued on next page

Table S17 – continued from previous page

Country	1990		2019		1990–2019
	Incident cases No.×10^4[95% UI]	ASR per 10,0000 No.[95% UI]	Incident cases No.×10^4[95% UI]	ASR per 10,0000 No.[95% UI]	EAPC No.[95% CI]
Guatemala	78.6 (68.3 to 91.3)	2049 (1782 to 2347.6)	78.6 (68.3 to 91.3)	2049 (1782 to 2347.6)	-2.98 (-3.16 to -2.79)
Guinea	72.2 (62 to 87)	1290 (1136.4 to 1500.8)	72.2 (62 to 87)	1290 (1136.4 to 1500.8)	-0.90 (-1.00 to -0.80)
Guinea-Bissau	9.8 (8.3 to 12.2)	1188.1 (1036.4 to 1413.4)	9.8 (8.3 to 12.2)	1188.1 (1036.4 to 1413.4)	-1.15 (-1.28 to -1.01)
Guyana	6.7 (5.7 to 7.9)	1567.8 (1356.9 to 1811.8)	6.7 (5.7 to 7.9)	1567.8 (1356.9 to 1811.8)	-2.10 (-2.30 to -1.89)
Haiti	59.9 (41.9 to 83.2)	1204.9 (894.2 to 1606.6)	59.9 (41.9 to 83.2)	1204.9 (894.2 to 1606.6)	-1.04 (-1.18 to -0.90)
Honduras	28.8 (23.9 to 34.8)	1092.5 (928.8 to 1274.6)	28.8 (23.9 to 34.8)	1092.5 (928.8 to 1274.6)	-1.42 (-1.61 to -1.22)
Hungary	19.4 (15.6 to 24.4)	488.8 (402.7 to 594.7)	19.4 (15.6 to 24.4)	488.8 (402.7 to 594.7)	0.32 (0.24 to 0.39)
Iceland	1.2 (1 to 1.5)	963.2 (778.2 to 1200.7)	1.2 (1 to 1.5)	963.2 (778.2 to 1200.7)	0.39 (0.30 to 0.48)
India	16661.8 (13532.9 to 20770.2)	3213.4 (2634.1 to 3929.4)	16661.8 (13532.9 to 20770.2)	3213.4 (2634.1 to 3929.4)	-0.06 (-0.29 to 0.18)
Indonesia	2569.8 (2057.6 to 3230)	2647.4 (2134.2 to 3254.1)	2569.8 (2057.6 to 3230)	2647.4 (2134.2 to 3254.1)	0.55 (0.41 to 0.70)
Iran (Islamic Republic of)	461.9 (359.2 to 590.2)	1282.9 (1013.2 to 1622.3)	461.9 (359.2 to 590.2)	1282.9 (1013.2 to 1622.3)	-0.01 (-0.23 to 0.21)
Iraq	153.5 (127.6 to 185.1)	1149.1 (966.1 to 1366.4)	153.5 (127.6 to 185.1)	1149.1 (966.1 to 1366.4)	0.39 (0.18 to 0.60)
Ireland	14.8 (11.4 to 19.2)	856.3 (669 to 1099.3)	14.8 (11.4 to 19.2)	856.3 (669 to 1099.3)	-0.01 (-0.14 to 0.12)
Israel	21.8 (16.9 to 27.4)	874.3 (679.7 to 1098.1)	21.8 (16.9 to 27.4)	874.3 (679.7 to 1098.1)	0.58 (0.39 to 0.78)
Italy	285.9 (219.5 to 372.8)	1098.8 (850.3 to 1415.9)	285.9 (219.5 to 372.8)	1098.8 (850.3 to 1415.9)	0.09 (-0.12 to 0.30)
Jamaica	13.7 (11.7 to 15.9)	1144.4 (977.9 to 1328.3)	13.7 (11.7 to 15.9)	1144.4 (977.9 to 1328.3)	-2.11 (-2.34 to -1.88)
Japan	363.3 (294.6 to 451.7)	737.2 (626.8 to 881.2)	363.3 (294.6 to 451.7)	737.2 (626.8 to 881.2)	-0.07 (-0.25 to 0.12)
Jordan	25.7 (21.3 to 30.3)	1164.9 (981.1 to 1364.4)	25.7 (21.3 to 30.3)	1164.9 (981.1 to 1364.4)	-0.07 (-0.18 to 0.04)
Kazakhstan	57.9 (46.9 to 72.6)	679.6 (547.9 to 854.7)	57.9 (46.9 to 72.6)	679.6 (547.9 to 854.7)	0.82 (0.70 to 0.95)
Kenya	222.9 (162.6 to 319.7)	1330.5 (1047.8 to 1756.6)	222.9 (162.6 to 319.7)	1330.5 (1047.8 to 1756.6)	-0.36 (-0.54 to -0.17)
Kiribati	0.9 (0.8 to 1.1)	2020.6 (1764.6 to 2337.4)	0.9 (0.8 to 1.1)	2020.6 (1764.6 to 2337.4)	-1.07 (-1.16 to -0.99)
Kuwait	7.7 (6.1 to 9.6)	912.9 (720.7 to 1148.1)	7.7 (6.1 to 9.6)	912.9 (720.7 to 1148.1)	0.50 (0.32 to 0.68)
Kyrgyzstan	14.6 (11.2 to 18.9)	533.4 (406.4 to 691.8)	14.6 (11.2 to 18.9)	533.4 (406.4 to 691.8)	0.60 (0.39 to 0.80)
Lao People's Democratic Republic	75.1 (52.2 to 105.9)	2608.8 (1982.7 to 3414.7)	75.1 (52.2 to 105.9)	2608.8 (1982.7 to 3414.7)	-1.00 (-1.21 to -0.79)
Latvia	8 (6.6 to 10)	767.7 (618.2 to 975.2)	8 (6.6 to 10)	767.7 (618.2 to 975.2)	-0.05 (-0.13 to 0.04)
Lebanon	22.7 (18.9 to 26.7)	1128.5 (948.3 to 1326.1)	22.7 (18.9 to 26.7)	1128.5 (948.3 to 1326.1)	0.34 (0.16 to 0.53)
Lesotho	17.2 (14.8 to 20.2)	1522.8 (1318 to 1752.5)	17.2 (14.8 to 20.2)	1522.8 (1318 to 1752.5)	-1.92 (-2.40 to -1.45)
Liberia	16 (12.4 to 21.3)	1134.8 (930.5 to 1418.8)	16 (12.4 to 21.3)	1134.8 (930.5 to 1418.8)	-1.66 (-1.83 to -1.49)
Libya	29.8 (24.9 to 36)	1090.7 (918.1 to 1312.5)	29.8 (24.9 to 36)	1090.7 (918.1 to 1312.5)	0.29 (0.11 to 0.46)
Lithuania	11.6 (9.2 to 14.8)	776.7 (619.1 to 1000.9)	11.6 (9.2 to 14.8)	776.7 (619.1 to 1000.9)	-0.12 (-0.21 to -0.03)
Luxembourg	2.1 (1.7 to 2.6)	1019.4 (832 to 1250.8)	2.1 (1.7 to 2.6)	1019.4 (832 to 1250.8)	0.32 (0.18 to 0.46)
Madagascar	192.2 (164.5 to 230.2)	2199 (1926.6 to 2525.9)	192.2 (164.5 to 230.2)	2199 (1926.6 to 2525.9)	-1.55 (-1.64 to -1.46)

Continued on next page

Table S17 – continued from previous page

Country	1990 Incident cases No.×10^4[95% UI]	1990 ASR per 10,0000 No.[95% UI]	2019 Incident cases No.×10^4[95% UI]	2019 ASR per 10,0000 No.[95% UI]	1990–2019 EAPC No.[95% CI]
Malawi	158.6 (103.5 to 230.2)	1961.5 (1439.6 to 2615.9)	158.6 (103.5 to 230.2)	1961.5 (1439.6 to 2615.9)	-0.36 (-0.65 to -0.08)
Malaysia	172 (152.7 to 194.7)	1781.8 (1568.9 to 2022.4)	172 (152.7 to 194.7)	1781.8 (1568.9 to 2022.4)	-0.05 (-0.22 to 0.12)
Maldives	5 (3.9 to 6.3)	3421.6 (2819.4 to 4204.5)	5 (3.9 to 6.3)	3421.6 (2819.4 to 4204.5)	-0.90 (-1.16 to -0.65)
Mali	118.2 (101.8 to 139.6)	1516.6 (1335.5 to 1746)	118.2 (101.8 to 139.6)	1516.6 (1335.5 to 1746)	0.02 (-0.18 to 0.23)
Malta	1.6 (1.2 to 2.1)	875.4 (689 to 1126.1)	1.6 (1.2 to 2.1)	875.4 (689 to 1126.1)	0.06 (-0.08 to 0.20)
Marshall Islands	0.3 (0.3 to 0.4)	1258.3 (1078 to 1447.4)	0.3 (0.3 to 0.4)	1258.3 (1078 to 1447.4)	-0.82 (-0.94 to -0.69)
Mauritania	22.8 (18.8 to 28.3)	1411.8 (1205.8 to 1684)	22.8 (18.8 to 28.3)	1411.8 (1205.8 to 1684)	-1.04 (-1.42 to -0.66)
Mauritius	11.6 (10.2 to 13.3)	2189.8 (1929.5 to 2496.2)	11.6 (10.2 to 13.3)	2189.8 (1929.5 to 2496.2)	-0.73 (-0.91 to -0.55)
Mexico	789.8 (623.9 to 998.2)	1897.1 (1534.5 to 2304.4)	789.8 (623.9 to 998.2)	1897.1 (1534.5 to 2304.4)	-1.27 (-1.38 to -1.15)
Micronesia (Federated States of)	0.7 (0.6 to 0.9)	1255.6 (1073.3 to 1464.1)	0.7 (0.6 to 0.9)	1255.6 (1073.3 to 1464.1)	-0.57 (-0.67 to -0.46)
Monaco	0.1 (0.1 to 0.2)	875.7 (698.8 to 1108.6)	0.1 (0.1 to 0.2)	875.7 (698.8 to 1108.6)	0.01 (-0.16 to 0.18)
Mongolia	8.6 (7 to 10.6)	579.5 (466.6 to 714.8)	8.6 (7 to 10.6)	579.5 (466.6 to 714.8)	0.57 (0.44 to 0.70)
Montenegro	2.8 (2.1 to 3.7)	1032.8 (760.2 to 1386.3)	2.8 (2.1 to 3.7)	1032.8 (760.2 to 1386.3)	0.94 (0.77 to 1.12)
Morocco	152.6 (127.8 to 184.8)	987.2 (828.5 to 1179.7)	152.6 (127.8 to 184.8)	987.2 (828.5 to 1179.7)	0.14 (-0.08 to 0.36)
Mozambique	114.8 (99.4 to 135)	1098.6 (975.5 to 1253.3)	114.8 (99.4 to 135)	1098.6 (975.5 to 1253.3)	-2.59 (-2.84 to -2.34)
Myanmar	489 (382.1 to 622.3)	2008.8 (1636.9 to 2444)	489 (382.1 to 622.3)	2008.8 (1636.9 to 2444)	-0.62 (-0.77 to -0.48)
Namibia	13.1 (10.6 to 16.2)	1421.8 (1192.9 to 1703.9)	13.1 (10.6 to 16.2)	1421.8 (1192.9 to 1703.9)	-0.42 (-0.74 to -0.10)
Nauru	0.1 (0.1 to 0.1)	1320 (1127.9 to 1540.1)	0.1 (0.1 to 0.1)	1320 (1127.9 to 1540.1)	-0.36 (-0.48 to -0.24)
Nepal	336.6 (277.8 to 425.3)	2600.3 (2220 to 3120.9)	336.6 (277.8 to 425.3)	2600.3 (2220 to 3120.9)	-0.55 (-0.74 to -0.36)
Netherlands	72.1 (57.6 to 91.9)	935.3 (752.8 to 1187.7)	72.1 (57.6 to 91.9)	935.3 (752.8 to 1187.7)	0.14 (-0.16 to 0.45)
New Zealand	11.4 (9.2 to 14.3)	695.7 (569.1 to 863.2)	11.4 (9.2 to 14.3)	695.7 (569.1 to 863.2)	-0.09 (-0.28 to 0.10)
Nicaragua	20.2 (18.3 to 23.3)	766.2 (687.8 to 872.9)	20.2 (18.3 to 23.3)	766.2 (687.8 to 872.9)	-1.73 (-1.99 to -1.47)
Niger	135.4 (117.2 to 161.8)	1717.6 (1522.4 to 1985.7)	135.4 (117.2 to 161.8)	1717.6 (1522.4 to 1985.7)	-1.11 (-1.29 to -0.94)
Nigeria	959.6 (730.7 to 1294.5)	1345.7 (1089.9 to 1685.2)	959.6 (730.7 to 1294.5)	1345.7 (1089.9 to 1685.2)	-0.56 (-0.86 to -0.26)
Niue	0 (0 to 0)	1455.6 (1244 to 1661.3)	0 (0 to 0)	1455.6 (1244 to 1661.3)	-0.01 (-0.12 to 0.11)
North Macedonia	11.3 (8.3 to 14.9)	1253.8 (892.4 to 1687.6)	11.3 (8.3 to 14.9)	1253.8 (892.4 to 1687.6)	0.59 (0.39 to 0.80)
Northern Mariana Islands	0.3 (0.3 to 0.3)	1450.1 (1256.4 to 1647.9)	0.3 (0.3 to 0.3)	1450.1 (1256.4 to 1647.9)	0.27 (0.11 to 0.42)
Norway	21 (16.5 to 26.8)	1037.8 (808.9 to 1329.7)	21 (16.5 to 26.8)	1037.8 (808.9 to 1329.7)	1.03 (0.91 to 1.14)
Oman	17.3 (15.3 to 20.1)	1436.1 (1251.4 to 1654.9)	17.3 (15.3 to 20.1)	1436.1 (1251.4 to 1654.9)	-0.14 (-0.30 to 0.02)
Pakistan	1985.3 (1587.4 to 2528.7)	2525.9 (2068.2 to 3057.1)	1985.3 (1587.4 to 2528.7)	2525.9 (2068.2 to 3057.1)	-1.09 (-1.28 to -0.91)

Continued on next page

Table S17 – continued from previous page

Country	1990		2019		1990–2019
	Incident cases No.×10^4[95% UI]	ASR per 10,0000 No.[95% UI]	Incident cases No.×10^4[95% UI]	ASR per 10,0000 No.[95% UI]	EAPC No.[95% CI]
Palau	0.1 (0.1 to 0.1)	1326.6 (1140.5 to 1544.2)	0.1 (0.1 to 0.1)	1326.6 (1140.5 to 1544.2)	-0.10 (-0.30 to 0.11)
Palestine	15.3 (12.7 to 18.4)	1084.1 (915.9 to 1277.6)	15.3 (12.7 to 18.4)	1084.1 (915.9 to 1277.6)	0.23 (0.05 to 0.40)
Panama	10.7 (8.9 to 12.5)	874 (728.1 to 1019.9)	10.7 (8.9 to 12.5)	874 (728.1 to 1019.9)	-0.73 (-1.07 to -0.40)
Papua New Guinea	58.1 (44.2 to 78.4)	2227.3 (1778.9 to 2843)	58.1 (44.2 to 78.4)	2227.3 (1778.9 to 2843)	-1.28 (-1.49 to -1.07)
Paraguay	16 (13.1 to 19.7)	792.9 (660.8 to 929.2)	16 (13.1 to 19.7)	792.9 (660.8 to 929.2)	1.08 (0.93 to 1.23)
Peru	72.6 (60.9 to 87.1)	686 (577.9 to 800.3)	72.6 (60.9 to 87.1)	686 (577.9 to 800.3)	-1.74 (-2.01 to -1.48)
Philippines	938.5 (676.2 to 1273.5)	2511.8 (1924.7 to 3243.4)	938.5 (676.2 to 1273.5)	2511.8 (1924.7 to 3243.4)	-1.21 (-1.53 to -0.88)
Poland	150.4 (113.6 to 195)	933.8 (703.3 to 1223.9)	150.4 (113.6 to 195)	933.8 (703.3 to 1223.9)	0.18 (-0.03 to 0.38)
Portugal	53.7 (43.3 to 66.1)	1020.4 (832 to 1238)	53.7 (43.3 to 66.1)	1020.4 (832 to 1238)	-0.06 (-0.17 to 0.04)
Puerto Rico	19.8 (15.9 to 25.3)	1138.9 (900 to 1474.3)	19.8 (15.9 to 25.3)	1138.9 (900 to 1474.3)	0.56 (0.40 to 0.72)
Qatar	2.2 (1.8 to 2.8)	1150.9 (938.3 to 1429.4)	2.2 (1.8 to 2.8)	1150.9 (938.3 to 1429.4)	1.38 (1.16 to 1.59)
Republic of Korea	171.4 (144.6 to 200.9)	841.1 (715.1 to 977.2)	171.4 (144.6 to 200.9)	841.1 (715.1 to 977.2)	-2.18 (-2.57 to -1.79)
Republic of Moldova	14.4 (11.3 to 18.8)	704.2 (544.9 to 945)	14.4 (11.3 to 18.8)	704.2 (544.9 to 945)	0.58 (0.50 to 0.65)
Romania	81.2 (61.8 to 106)	861.2 (651.6 to 1145.7)	81.2 (61.8 to 106)	861.2 (651.6 to 1145.7)	0.21 (0.06 to 0.35)
Russian Federation	478.5 (384.1 to 588.1)	792.9 (634.9 to 999.8)	478.5 (384.1 to 588.1)	792.9 (634.9 to 999.8)	0.16 (-0.01 to 0.33)
Rwanda	64.8 (52.5 to 82.4)	1252.3 (1059 to 1496.4)	64.8 (52.5 to 82.4)	1252.3 (1059 to 1496.4)	-1.39 (-1.68 to -1.10)
Saint Kitts and Nevis	0.2 (0.2 to 0.3)	1166.7 (990.2 to 1377.1)	0.2 (0.2 to 0.3)	1166.7 (990.2 to 1377.1)	-0.34 (-0.42 to -0.25)
Saint Lucia	0.7 (0.6 to 0.8)	968.9 (817.3 to 1146.3)	0.7 (0.6 to 0.8)	968.9 (817.3 to 1146.3)	-0.72 (-0.86 to -0.58)
Saint Vincent and the Grenadines	0.6 (0.5 to 0.8)	1150.1 (948 to 1382.9)	0.6 (0.5 to 0.8)	1150.1 (948 to 1382.9)	-0.60 (-0.75 to -0.45)
Samoa	0.7 (0.6 to 0.9)	1072.3 (884.3 to 1256.4)	0.7 (0.6 to 0.9)	1072.3 (884.3 to 1256.4)	-0.16 (-0.35 to 0.03)
San Marino	0.1 (0.1 to 0.1)	935.6 (750.1 to 1163.4)	0.1 (0.1 to 0.1)	935.6 (750.1 to 1163.4)	0.03 (-0.12 to 0.19)
Sao Tome and Principe	0.9 (0.8 to 1.1)	1100.6 (934.5 to 1297.7)	0.9 (0.8 to 1.1)	1100.6 (934.5 to 1297.7)	-1.42 (-1.65 to -1.19)
Saudi Arabia	117.1 (95.6 to 142.1)	1280.6 (1069.6 to 1517)	117.1 (95.6 to 142.1)	1280.6 (1069.6 to 1517)	0.05 (-0.08 to 0.19)
Senegal	73.2 (62.6 to 87.3)	1210.3 (1055.6 to 1404.7)	73.2 (62.6 to 87.3)	1210.3 (1055.6 to 1404.7)	-1.00 (-1.17 to -0.84)
Serbia	33.6 (26.2 to 42.9)	843.2 (651.7 to 1096.3)	33.6 (26.2 to 42.9)	843.2 (651.7 to 1096.3)	0.48 (0.39 to 0.58)
Seychelles	0.6 (0.6 to 0.8)	1816 (1540.8 to 2100.2)	0.6 (0.6 to 0.8)	1816 (1540.8 to 2100.2)	0.25 (0.13 to 0.36)
Sierra Leone	41.2 (32.2 to 54.4)	1436.9 (1170.9 to 1784.5)	41.2 (32.2 to 54.4)	1436.9 (1170.9 to 1784.5)	-1.29 (-1.40 to -1.19)

Continued on next page

Table S17 – continued from previous page

Country	1990 Incident cases No.×10^4[95% UI]	1990 ASR per 10,0000 No.[95% UI]	2019 Incident cases No.×10^4[95% UI]	2019 ASR per 10,0000 No.[95% UI]	1990–2019 EAPC No.[95% CI]
Singapore	11 (9.4 to 12.8)	901.7 (792.5 to 1029)	11 (9.4 to 12.8)	901.7 (792.5 to 1029)	-0.17 (-0.24 to -0.10)
Slovakia	18.4 (14.1 to 24)	813 (618.8 to 1090.1)	18.4 (14.1 to 24)	813 (618.8 to 1090.1)	0.28 (0.21 to 0.35)
Slovenia	6.6 (5 to 8.5)	859.8 (638.5 to 1144.9)	6.6 (5 to 8.5)	859.8 (638.5 to 1144.9)	0.08 (0.00 to 0.16)
Solomon Islands	3.4 (2.6 to 4.4)	1576 (1295.4 to 1928.7)	3.4 (2.6 to 4.4)	1576 (1295.4 to 1928.7)	-1.05 (-1.19 to -0.90)
Somalia	129 (107.5 to 158.8)	2468.1 (2122.2 to 2889.8)	129 (107.5 to 158.8)	2468.1 (2122.2 to 2889.8)	-2.12 (-2.38 to -1.86)
South Africa	191.9 (164 to 229.9)	920.3 (785.7 to 1087.5)	191.9 (164 to 229.9)	920.3 (785.7 to 1087.5)	-1.38 (-1.64 to -1.13)
South Sudan	86.5 (72.1 to 107.9)	2086.7 (1794.6 to 2483)	86.5 (72.1 to 107.9)	2086.7 (1794.6 to 2483)	-1.18 (-1.32 to -1.03)
Spain	206.5 (160 to 267.1)	1045.8 (825.3 to 1347)	206.5 (160 to 267.1)	1045.8 (825.3 to 1347)	0.18 (0.03 to 0.33)
Sri Lanka	189.6 (168.8 to 213.9)	2259.3 (2002.7 to 2563.9)	189.6 (168.8 to 213.9)	2259.3 (2002.7 to 2563.9)	-0.02 (-0.15 to 0.12)
Sudan	423.4 (303.9 to 559.7)	2452.1 (1908.6 to 3139.4)	423.4 (303.9 to 559.7)	2452.1 (1908.6 to 3139.4)	-1.18 (-1.60 to -0.76)
Suriname	2.2 (1.8 to 2.6)	1118.1 (922.5 to 1349.2)	2.2 (1.8 to 2.6)	1118.1 (922.5 to 1349.2)	-0.41 (-0.58 to -0.23)
Sweden	43.1 (33.8 to 54.7)	1036.5 (814.4 to 1322.1)	43.1 (33.8 to 54.7)	1036.5 (814.4 to 1322.1)	0.22 (0.01 to 0.43)
Switzerland	30.8 (23.6 to 40.5)	887.4 (684.6 to 1150.5)	30.8 (23.6 to 40.5)	887.4 (684.6 to 1150.5)	0.57 (0.46 to 0.68)
Syrian Arab Republic	139.7 (119.1 to 164.5)	1426.7 (1215.8 to 1671.2)	139.7 (119.1 to 164.5)	1426.7 (1215.8 to 1671.2)	0.13 (-0.09 to 0.35)
Taiwan (Province of China)	151.7 (124.4 to 183.6)	1690.5 (1361.8 to 2050.3)	151.7 (124.4 to 183.6)	1690.5 (1361.8 to 2050.3)	0.07 (-0.16 to 0.31)
Tajikistan	40.1 (33.8 to 49.1)	946.9 (798.2 to 1156.6)	40.1 (33.8 to 49.1)	946.9 (798.2 to 1156.6)	-0.13 (-0.30 to 0.04)
Thailand	380.1 (331.3 to 434.6)	1441.5 (1256.5 to 1645.2)	380.1 (331.3 to 434.6)	1441.5 (1256.5 to 1645.2)	-0.09 (-0.22 to 0.04)
Timor-Leste	19.9 (14.6 to 27)	3329.9 (2638.3 to 4197.4)	19.9 (14.6 to 27)	3329.9 (2638.3 to 4197.4)	-1.43 (-1.61 to -1.26)
Togo	34.5 (27.6 to 43.9)	1234.8 (1031.7 to 1490.2)	34.5 (27.6 to 43.9)	1234.8 (1031.7 to 1490.2)	-1.00 (-1.14 to -0.86)
Tokelau	0 (0 to 0)	1385.1 (1199.2 to 1587.1)	0 (0 to 0)	1385.1 (1199.2 to 1587.1)	-0.19 (-0.29 to -0.09)
Tonga	0.7 (0.6 to 0.8)	1376.3 (1191.7 to 1584.5)	0.7 (0.6 to 0.8)	1376.3 (1191.7 to 1584.5)	-0.36 (-0.53 to -0.19)
Trinidad and Tobago	5.6 (4.9 to 6.5)	990 (853.4 to 1154.2)	5.6 (4.9 to 6.5)	990 (853.4 to 1154.2)	-0.85 (-1.01 to -0.69)
Tunisia	56.8 (45.2 to 72.1)	1203.9 (972.7 to 1481.4)	56.8 (45.2 to 72.1)	1203.9 (972.7 to 1481.4)	0.07 (-0.10 to 0.23)
Turkey	297.7 (249.4 to 364.1)	880.4 (745.1 to 1072.9)	297.7 (249.4 to 364.1)	880.4 (745.1 to 1072.9)	1.78 (1.54 to 2.02)
Turkmenistan	22.4 (17.6 to 29.4)	826 (652.4 to 1068.6)	22.4 (17.6 to 29.4)	826 (652.4 to 1068.6)	-0.17 (-0.32 to -0.02)
Tuvalu	0.1 (0.1 to 0.1)	1407.4 (1159.8 to 1705.5)	0.1 (0.1 to 0.1)	1407.4 (1159.8 to 1705.5)	-0.63 (-0.74 to -0.52)
Uganda	156 (115.2 to 211.5)	1038.9 (828.4 to 1296.8)	156 (115.2 to 211.5)	1038.9 (828.4 to 1296.8)	-0.99 (-1.16 to -0.82)
Ukraine	177.6 (145.4 to 214)	878.9 (723.2 to 1070.9)	177.6 (145.4 to 214)	878.9 (723.2 to 1070.9)	0.39 (0.19 to 0.59)
United Arab Emirates	12.4 (10.9 to 14.2)	1294.7 (1127.9 to 1504.8)	12.4 (10.9 to 14.2)	1294.7 (1127.9 to 1504.8)	0.02 (-0.12 to 0.17)

Continued on next page

Table S17 – continued from previous page

Country	1990		2019		1990–2019
	Incident cases No.×10^4[95% UI]	ASR per 10,0000 No.[95% UI]	Incident cases No.×10^4[95% UI]	ASR per 10,0000 No.[95% UI]	EAPC No.[95% CI]
United Kingdom	353.2 (276.4 to 454.6)	1283.3 (1000.6 to 1637.5)	353.2 (276.4 to 454.6)	1283.3 (1000.6 to 1637.5)	-0.14 (-0.46 to 0.18)
United Republic of Tanzania	221.1 (180.2 to 275.6)	1078.6 (922.9 to 1277)	221.1 (180.2 to 275.6)	1078.6 (922.9 to 1277)	-1.36 (-1.51 to -1.20)
United States of America	812.1 (633.8 to 1034.6)	614.6 (476.8 to 787.4)	812.1 (633.8 to 1034.6)	614.6 (476.8 to 787.4)	-0.12 (-0.33 to 0.08)
United States Virgin Islands	0.5 (0.5 to 0.6)	1000.9 (866 to 1149.1)	0.5 (0.5 to 0.6)	1000.9 (866 to 1149.1)	-0.11 (-0.23 to 0.02)
Uruguay	9.9 (8.2 to 12)	633.6 (525.5 to 765.9)	9.9 (8.2 to 12)	633.6 (525.5 to 765.9)	0.13 (-0.06 to 0.32)
Uzbekistan	134.9 (108.1 to 166.5)	889.2 (723.4 to 1090.7)	134.9 (108.1 to 166.5)	889.2 (723.4 to 1090.7)	-0.31 (-0.44 to -0.17)
Vanuatu	1.1 (0.9 to 1.4)	1301.3 (1098.6 to 1548.9)	1.1 (0.9 to 1.4)	1301.3 (1098.6 to 1548.9)	-0.49 (-0.63 to -0.36)
Venezuela (Bolivarian Republic of)	90.2 (77.7 to 105.6)	834.3 (716.5 to 973.3)	90.2 (77.7 to 105.6)	834.3 (716.5 to 973.3)	-0.30 (-0.54 to -0.05)
Viet Nam	816.9 (685.6 to 990.3)	2055.7 (1715.7 to 2487.3)	816.9 (685.6 to 990.3)	2055.7 (1715.7 to 2487.3)	-0.02 (-0.26 to 0.22)
Yemen	276.2 (192.3 to 393.5)	2348 (1783.7 to 3104.9)	276.2 (192.3 to 393.5)	2348 (1783.7 to 3104.9)	-0.42 (-0.59 to -0.25)
Zambia	63.3 (47.5 to 84.8)	1038 (841.6 to 1286.7)	63.3 (47.5 to 84.8)	1038 (841.6 to 1286.7)	-0.46 (-0.60 to -0.33)
Zimbabwe	76.1 (63.3 to 92.4)	1258.6 (1080.6 to 1462.6)	76.1 (63.3 to 92.4)	1258.6 (1080.6 to 1462.6)	0.26 (0.01 to 0.51)

Abbreviation: ASR, age standardized rate; CI, confidence interval; EAPC, estimated annual percentage change; UI, uncertainty interval.

Table S18: The change of protein-energy malnutrition cases between 1990 and 2019 at national level for male.

Country	1990		2019		1990–2019
	Incident cases No.×10^4[95% UI]	ASR per 10,0000 No.[95% UI]	Incident cases No.×10^4[95% UI]	ASR per 10,0000 No.[95% UI]	EAPC No.[95% CI]
Afghanistan	102.7 (79.1 to 131)	1048.8 (850.2 to 1281.4)	102.7 (79.1 to 131)	1048.8 (850.2 to 1281.4)	0.37 (0.17 to 0.58)
Albania	26.1 (19.5 to 34)	1332.9 (1012.6 to 1714.6)	26.1 (19.5 to 34)	1332.9 (1012.6 to 1714.6)	1.11 (0.81 to 1.42)
Algeria	219.6 (186.7 to 262.7)	1360.3 (1151 to 1617.1)	219.6 (186.7 to 262.7)	1360.3 (1151 to 1617.1)	-0.18 (-0.38 to 0.02)
American Samoa	0.4 (0.3 to 0.4)	1269.8 (1111.4 to 1449.6)	0.4 (0.3 to 0.4)	1269.8 (1111.4 to 1449.6)	-0.21 (-0.38 to -0.04)
Andorra	0.3 (0.2 to 0.4)	1048.1 (836.4 to 1322.6)	0.3 (0.2 to 0.4)	1048.1 (836.4 to 1322.6)	0.33 (0.15 to 0.51)
Angola	123.1 (103.2 to 150)	1613 (1416.9 to 1868.4)	123.1 (103.2 to 150)	1613 (1416.9 to 1868.4)	-2.68 (-2.84 to -2.52)

Continued on next page

Table S18 – continued from previous page

Country	1990		2019		1990–2019
	Incident cases No.×10^4[95% UI]	ASR per 10,0000 No.[95% UI]	Incident cases No.×10^4[95% UI]	ASR per 10,0000 No.[95% UI]	EAPC No.[95% CI]
Antigua and Barbuda	0.3 (0.3 to 0.4)	1175.7 (1011.8 to 1375.3)	0.3 (0.3 to 0.4)	1175.7 (1011.8 to 1375.3)	-0.60 (-0.68 to -0.52)
Argentina	150.8 (126.5 to 179.9)	943.9 (789.6 to 1121.8)	150.8 (126.5 to 179.9)	943.9 (789.6 to 1121.8)	0.06 (-0.13 to 0.25)
Armenia	11.1 (8.7 to 14.1)	615.1 (484.9 to 779.1)	11.1 (8.7 to 14.1)	615.1 (484.9 to 779.1)	0.11 (-0.08 to 0.29)
Australia	37.1 (31.7 to 44.3)	495.3 (432.2 to 580.6)	37.1 (31.7 to 44.3)	495.3 (432.2 to 580.6)	1.14 (0.68 to 1.59)
Austria	41.6 (31.7 to 53.7)	1153.5 (892 to 1468.5)	41.6 (31.7 to 53.7)	1153.5 (892 to 1468.5)	-0.06 (-0.20 to 0.09)
Azerbaijan	42.1 (30.1 to 58.5)	941 (685.1 to 1298.8)	42.1 (30.1 to 58.5)	941 (685.1 to 1298.8)	-1.18 (-1.41 to -0.94)
Bahamas	2.9 (2.3 to 3.5)	2403.3 (1941.6 to 2906)	2.9 (2.3 to 3.5)	2403.3 (1941.6 to 2906)	-0.53 (-0.84 to -0.21)
Bahrain	4.6 (3.9 to 5.3)	1598.7 (1376.2 to 1841.7)	4.6 (3.9 to 5.3)	1598.7 (1376.2 to 1841.7)	0.17 (0.01 to 0.33)
Bangladesh	2202.3 (1911.2 to 2543.5)	2856.2 (2493.2 to 3260.9)	2202.3 (1911.2 to 2543.5)	2856.2 (2493.2 to 3260.9)	-1.46 (-1.83 to -1.09)
Barbados	1.1 (0.9 to 1.3)	948.6 (811.6 to 1103.1)	1.1 (0.9 to 1.3)	948.6 (811.6 to 1103.1)	-0.17 (-0.35 to 0.00)
Belarus	33.2 (26.5 to 41.8)	784.3 (616.7 to 1000)	33.2 (26.5 to 41.8)	784.3 (616.7 to 1000)	0.08 (-0.03 to 0.20)
Belgium	54.5 (42.9 to 68.2)	1128.5 (900.7 to 1402.2)	54.5 (42.9 to 68.2)	1128.5 (900.7 to 1402.2)	0.76 (0.57 to 0.94)
Belize	0.9 (0.7 to 1)	826.1 (700.7 to 958.4)	0.9 (0.7 to 1)	826.1 (700.7 to 958.4)	-1.53 (-1.69 to -1.37)
Benin	74.1 (60.2 to 95.3)	1924.8 (1613.8 to 2326.5)	74.1 (60.2 to 95.3)	1924.8 (1613.8 to 2326.5)	-1.85 (-2.01 to -1.68)
Bermuda	0.3 (0.2 to 0.3)	936.4 (771.8 to 1136.7)	0.3 (0.2 to 0.3)	936.4 (771.8 to 1136.7)	0.16 (-0.01 to 0.32)
Bhutan	7.7 (6.5 to 9.2)	1843.2 (1569.5 to 2148.2)	7.7 (6.5 to 9.2)	1843.2 (1569.5 to 2148.2)	2.47 (2.11 to 2.82)
Bolivia (Plurinational State of)	29.4 (25.5 to 34.4)	826.9 (721.7 to 944.2)	29.4 (25.5 to 34.4)	826.9 (721.7 to 944.2)	-0.01 (-0.20 to 0.18)
Bosnia and Herzegovina	16 (12.4 to 20.5)	798.7 (609 to 1024.9)	16 (12.4 to 20.5)	798.7 (609 to 1024.9)	0.63 (0.55 to 0.72)
Botswana	7.9 (6.8 to 9.1)	1033.2 (898 to 1184.9)	7.9 (6.8 to 9.1)	1033.2 (898 to 1184.9)	-0.21 (-0.42 to 0.01)
Brazil	557.6 (461 to 669.1)	784.7 (650.3 to 938.5)	557.6 (461 to 669.1)	784.7 (650.3 to 938.5)	-1.44 (-1.73 to -1.15)
Brunei Darussalam	0.9 (0.8 to 1.1)	658.4 (549.5 to 803.5)	0.9 (0.8 to 1.1)	658.4 (549.5 to 803.5)	0.53 (0.46 to 0.59)
Bulgaria	25.8 (20 to 33.2)	776.4 (601.8 to 997.4)	25.8 (20 to 33.2)	776.4 (601.8 to 997.4)	0.75 (0.60 to 0.89)
Burkina Faso	163.2 (139.9 to 197.9)	2023 (1775.8 to 2361.9)	163.2 (139.9 to 197.9)	2023 (1775.8 to 2361.9)	-0.30 (-0.75 to 0.15)
Burundi	80 (65.1 to 100)	1939 (1660.5 to 2279.7)	80 (65.1 to 100)	1939 (1660.5 to 2279.7)	-2.06 (-2.32 to -1.81)
Cabo Verde	1.7 (1.4 to 2.1)	856.4 (717.1 to 1019.2)	1.7 (1.4 to 2.1)	856.4 (717.1 to 1019.2)	-0.13 (-0.26 to 0.00)
Cambodia	198.6 (146.3 to 267)	2900.3 (2350.1 to 3584.3)	198.6 (146.3 to 267)	2900.3 (2350.1 to 3584.3)	-1.44 (-1.62 to -1.26)
Cameroon	88.9 (72.9 to 110.5)	1174.5 (989.1 to 1393.9)	88.9 (72.9 to 110.5)	1174.5 (989.1 to 1393.9)	-0.66 (-0.86 to -0.45)
Canada	97.8 (79.3 to 121.2)	718.8 (582.2 to 893)	97.8 (79.3 to 121.2)	718.8 (582.2 to 893)	-0.15 (-0.30 to 0.00)
Central African Republic	23.2 (19.7 to 28)	1101.1 (961.5 to 1271.5)	23.2 (19.7 to 28)	1101.1 (961.5 to 1271.5)	-0.12 (-0.40 to 0.17)

Continued on next page

Table S18 – continued from previous page

Country	1990		2019		1990–2019
	Incident cases No.×10^4[95% UI]	ASR per 10,0000 No.[95% UI]	Incident cases No.×10^4[95% UI]	ASR per 10,0000 No.[95% UI]	EAPC No.[95% CI]
Chad	116.2 (98.5 to 141.2)	2185.2 (1908.6 to 2544.6)	116.2 (98.5 to 141.2)	2185.2 (1908.6 to 2544.6)	-1.07 (-1.18 to -0.97)
Chile	30.3 (25.5 to 35.9)	481.5 (402.5 to 571.4)	30.3 (25.5 to 35.9)	481.5 (402.5 to 571.4)	0.81 (0.33 to 1.29)
China	9786.2 (7385.5 to 12816.8)	1638.1 (1241.4 to 2145.3)	9786.2 (7385.5 to 12816.8)	1638.1 (1241.4 to 2145.3)	1.30 (1.17 to 1.43)
Colombia	105.2 (91.9 to 118.9)	641.5 (551.9 to 733)	105.2 (91.9 to 118.9)	641.5 (551.9 to 733)	-0.70 (-0.89 to -0.51)
Comoros	4.9 (4.4 to 5.3)	1588 (1444 to 1737.5)	4.9 (4.4 to 5.3)	1588 (1444 to 1737.5)	-1.49 (-1.58 to -1.39)
Congo	15.5 (12.6 to 19.1)	945.9 (800.5 to 1115.7)	15.5 (12.6 to 19.1)	945.9 (800.5 to 1115.7)	-0.81 (-0.98 to -0.63)
Cook Islands	0.1 (0.1 to 0.2)	1308.8 (1136.4 to 1498.7)	0.1 (0.1 to 0.2)	1308.8 (1136.4 to 1498.7)	-0.08 (-0.21 to 0.05)
Costa Rica	11.2 (9.2 to 13.7)	758.2 (611.9 to 933.3)	11.2 (9.2 to 13.7)	758.2 (611.9 to 933.3)	0.05 (-0.27 to 0.37)
Croatia	12.4 (9.9 to 15.5)	645.7 (520.6 to 794.3)	12.4 (9.9 to 15.5)	645.7 (520.6 to 794.3)	0.79 (0.59 to 1.00)
Cuba	36.5 (29.5 to 45.2)	701.9 (568.7 to 863.7)	36.5 (29.5 to 45.2)	701.9 (568.7 to 863.7)	0.20 (-0.06 to 0.45)
Cyprus	4.1 (3.3 to 5.1)	1075.5 (877.4 to 1326.1)	4.1 (3.3 to 5.1)	1075.5 (877.4 to 1326.1)	0.91 (0.78 to 1.04)
Czechia	30.1 (23.5 to 38.6)	739.1 (568.1 to 958.5)	30.1 (23.5 to 38.6)	739.1 (568.1 to 958.5)	1.79 (1.56 to 2.02)
Ivoirian	128.4 (107.3 to 154.5)	1354 (1166.5 to 1570.3)	128.4 (107.3 to 154.5)	1354 (1166.5 to 1570.3)	-0.96 (-1.12 to -0.80)
Democratic People's Republic of Korea	384.3 (292.3 to 517.4)	2949.7 (2363.3 to 3762)	384.3 (292.3 to 517.4)	2949.7 (2363.3 to 3762)	-2.21 (-2.98 to -1.44)
Democratic Republic of the Congo	388.1 (337.6 to 451.7)	1225.1 (1093.8 to 1391.4)	388.1 (337.6 to 451.7)	1225.1 (1093.8 to 1391.4)	-1.00 (-1.30 to -0.69)
Denmark	26.4 (20 to 33.8)	1086.3 (849.1 to 1382.1)	26.4 (20 to 33.8)	1086.3 (849.1 to 1382.1)	0.55 (0.32 to 0.78)
Djibouti	8.4 (7.1 to 10.2)	2211.6 (1929.7 to 2580.8)	8.4 (7.1 to 10.2)	2211.6 (1929.7 to 2580.8)	-0.66 (-0.89 to -0.43)
Dominica	0.4 (0.3 to 0.5)	1108 (939.3 to 1302.5)	0.4 (0.3 to 0.5)	1108 (939.3 to 1302.5)	-0.49 (-0.62 to -0.36)
Dominican Republic	37.7 (29.4 to 48.4)	880.5 (723.1 to 1073.5)	37.7 (29.4 to 48.4)	880.5 (723.1 to 1073.5)	-1.57 (-1.72 to -1.43)
Ecuador	39.8 (35.3 to 44.8)	786.5 (695.1 to 889.6)	39.8 (35.3 to 44.8)	786.5 (695.1 to 889.6)	-0.95 (-1.16 to -0.74)
Egypt	439.7 (360.1 to 537.8)	1188.8 (977.9 to 1441.3)	439.7 (360.1 to 537.8)	1188.8 (977.9 to 1441.3)	-0.53 (-0.78 to -0.28)
El Salvador	19.9 (17.2 to 23.1)	792.9 (682.6 to 905.7)	19.9 (17.2 to 23.1)	792.9 (682.6 to 905.7)	-0.30 (-0.46 to -0.13)
Equatorial Guinea	3.7 (3.3 to 4.2)	938.1 (850.7 to 1045.8)	3.7 (3.3 to 4.2)	938.1 (850.7 to 1045.8)	-2.41 (-2.77 to -2.04)
Eritrea	63.3 (51 to 81.3)	3039.4 (2561 to 3682.6)	63.3 (51 to 81.3)	3039.4 (2561 to 3682.6)	-2.16 (-2.48 to -1.85)
Estonia	6 (4.9 to 7.2)	932.5 (764.6 to 1128.7)	6 (4.9 to 7.2)	932.5 (764.6 to 1128.7)	-0.35 (-0.59 to -0.10)
Eswatini	4.9 (3.7 to 6.6)	1054.7 (849.5 to 1295.1)	4.9 (3.7 to 6.6)	1054.7 (849.5 to 1295.1)	-0.57 (-0.73 to -0.41)
Ethiopia	714.3 (584.7 to 897)	1985.3 (1684.1 to 2345.3)	714.3 (584.7 to 897)	1985.3 (1684.1 to 2345.3)	-1.18 (-1.52 to -0.85)
Fiji	5.3 (4.7 to 5.9)	1307.8 (1154.4 to 1475.9)	5.3 (4.7 to 5.9)	1307.8 (1154.4 to 1475.9)	-0.39 (-0.58 to -0.20)

Continued on next page

Table S18 – continued from previous page

Country	1990		2019		1990–2019
	Incident cases No.×10^4[95% UI]	ASR per 10,0000 No.[95% UI]	Incident cases No.×10^4[95% UI]	ASR per 10,0000 No.[95% UI]	EAPC No.[95% CI]
Finland	25.3 (19.2 to 32.3)	1092.9 (844.6 to 1380.5)	25.3 (19.2 to 32.3)	1092.9 (844.6 to 1380.5)	0.02 (-0.10 to 0.15)
France	419.5 (347.7 to 501.4)	1453.8 (1209 to 1729)	419.5 (347.7 to 501.4)	1453.8 (1209 to 1729)	0.75 (0.54 to 0.96)
Gabon	4.7 (4 to 5.6)	734.5 (634.5 to 843.6)	4.7 (4 to 5.6)	734.5 (634.5 to 843.6)	-0.56 (-0.70 to -0.42)
Gambia	16.9 (13.7 to 21.1)	2261.7 (1883.4 to 2698.3)	16.9 (13.7 to 21.1)	2261.7 (1883.4 to 2698.3)	-0.59 (-0.87 to -0.31)
Georgia	13.3 (10.3 to 17)	565.1 (436.8 to 728.1)	13.3 (10.3 to 17)	565.1 (436.8 to 728.1)	-0.55 (-0.87 to -0.23)
Germany	336.8 (260.1 to 437.3)	874.9 (668.8 to 1131)	336.8 (260.1 to 437.3)	874.9 (668.8 to 1131)	0.61 (0.50 to 0.73)
Ghana	171.3 (148.4 to 201.3)	1641.5 (1432 to 1888)	171.3 (148.4 to 201.3)	1641.5 (1432 to 1888)	-1.03 (-1.18 to -0.87)
Greece	32.5 (24.6 to 43)	715.7 (562.7 to 925.8)	32.5 (24.6 to 43)	715.7 (562.7 to 925.8)	0.31 (0.17 to 0.45)
Greenland	0.2 (0.2 to 0.2)	662.3 (537.5 to 804.9)	0.2 (0.2 to 0.2)	662.3 (537.5 to 804.9)	0.06 (-0.18 to 0.31)
Grenada	0.5 (0.4 to 0.6)	1224.9 (1040.6 to 1432)	0.5 (0.4 to 0.6)	1224.9 (1040.6 to 1432)	-0.30 (-0.42 to -0.18)
Guam	1 (0.8 to 1.1)	1318.4 (1131.5 to 1537)	1 (0.8 to 1.1)	1318.4 (1131.5 to 1537)	-0.66 (-0.85 to -0.46)
Guatemala	56.3 (52.4 to 60.9)	1281.2 (1159 to 1413.6)	56.3 (52.4 to 60.9)	1281.2 (1159 to 1413.6)	-1.89 (-2.05 to -1.72)
Guinea	63 (51.4 to 78.9)	1214.2 (1042.1 to 1427.3)	63 (51.4 to 78.9)	1214.2 (1042.1 to 1427.3)	-0.43 (-0.72 to -0.14)
Guinea-Bissau	12.1 (9.9 to 15.1)	1617 (1378.1 to 1911.6)	12.1 (9.9 to 15.1)	1617 (1378.1 to 1911.6)	-1.40 (-1.55 to -1.25)
Guyana	7.6 (6.5 to 8.9)	1828.3 (1579.6 to 2095.1)	7.6 (6.5 to 8.9)	1828.3 (1579.6 to 2095.1)	-1.76 (-1.91 to -1.60)
Haiti	64.3 (47.5 to 85.1)	1424.3 (1134.5 to 1793.1)	64.3 (47.5 to 85.1)	1424.3 (1134.5 to 1793.1)	-1.37 (-1.53 to -1.20)
Honduras	21 (18.4 to 23.7)	852.3 (740.2 to 979.4)	21 (18.4 to 23.7)	852.3 (740.2 to 979.4)	-1.38 (-1.54 to -1.21)
Hungary	23.6 (19 to 28.8)	593.2 (495.4 to 707.2)	23.6 (19 to 28.8)	593.2 (495.4 to 707.2)	0.49 (0.39 to 0.58)
Iceland	1.4 (1.1 to 1.8)	1111.2 (873.3 to 1393.7)	1.4 (1.1 to 1.8)	1111.2 (873.3 to 1393.7)	0.38 (0.24 to 0.52)
India	21005.4 (17494.7 to 25353.6)	3803.7 (3165.2 to 4546.4)	21005.4 (17494.7 to 25353.6)	3803.7 (3165.2 to 4546.4)	0.03 (-0.09 to 0.15)
Indonesia	3387.9 (2722.8 to 4177.7)	3595.8 (2930.7 to 4334.8)	3387.9 (2722.8 to 4177.7)	3595.8 (2930.7 to 4334.8)	-0.08 (-0.24 to 0.08)
Iran (Islamic Republic of)	482.4 (390.7 to 593.3)	1326.6 (1077 to 1604.6)	482.4 (390.7 to 593.3)	1326.6 (1077 to 1604.6)	-0.33 (-0.54 to -0.12)
Iraq	183 (151.9 to 220.6)	1325.2 (1117.4 to 1566.8)	183 (151.9 to 220.6)	1325.2 (1117.4 to 1566.8)	0.01 (-0.15 to 0.17)
Ireland	17.7 (13.8 to 23.2)	1033.1 (813.5 to 1326.3)	17.7 (13.8 to 23.2)	1033.1 (813.5 to 1326.3)	0.22 (0.06 to 0.38)
Israel	25.8 (20.4 to 31.9)	1049.8 (837.3 to 1298.6)	25.8 (20.4 to 31.9)	1049.8 (837.3 to 1298.6)	0.46 (0.32 to 0.60)
Italy	327.6 (255 to 426)	1306.6 (1027.8 to 1657.5)	327.6 (255 to 426)	1306.6 (1027.8 to 1657.5)	0.35 (0.11 to 0.60)
Jamaica	15 (12.9 to 17.5)	1287.6 (1106 to 1496.5)	15 (12.9 to 17.5)	1287.6 (1106 to 1496.5)	-1.72 (-1.85 to -1.58)
Japan	411 (333.2 to 510.2)	813.5 (684.2 to 977.8)	411 (333.2 to 510.2)	813.5 (684.2 to 977.8)	0.05 (-0.17 to 0.28)
Jordan	27.7 (23.8 to 32.7)	1111.4 (946.5 to 1316.1)	27.7 (23.8 to 32.7)	1111.4 (946.5 to 1316.1)	0.31 (0.19 to 0.43)
Kazakhstan	51.7 (43.4 to 61.6)	616.8 (516.4 to 736.2)	51.7 (43.4 to 61.6)	616.8 (516.4 to 736.2)	0.40 (0.32 to 0.49)
Kenya	315.4 (238.1 to 423.1)	2153.6 (1733.1 to 2682.7)	315.4 (238.1 to 423.1)	2153.6 (1733.1 to 2682.7)	-0.48 (-0.70 to -0.27)
Kiribati	0.8 (0.7 to 1)	1774.2 (1564.7 to 2014.5)	0.8 (0.7 to 1)	1774.2 (1564.7 to 2014.5)	-0.97 (-1.07 to -0.88)
Kuwait	10.8 (8.2 to 14.1)	1060.7 (800.7 to 1391.2)	10.8 (8.2 to 14.1)	1060.7 (800.7 to 1391.2)	0.38 (0.26 to 0.49)
Kyrgyzstan	14.9 (11.7 to 19)	571.9 (453.3 to 719.3)	14.9 (11.7 to 19)	571.9 (453.3 to 719.3)	-0.46 (-0.86 to -0.06)

Continued on next page

Country	1990		2019		1990–2019
	Incident cases No.×10^4[95% UI]	ASR per 10,0000 No.[95% UI]	Incident cases No.×10^4[95% UI]	ASR per 10,0000 No.[95% UI]	EAPC No.[95% CI]
Lao People's Democratic Republic	87.3 (60.8 to 117.3)	3284.5 (2558.6 to 4068.5)	87.3 (60.8 to 117.3)	3284.5 (2558.6 to 4068.5)	-1.77 (-1.92 to -1.63)
Latvia	9 (7.2 to 11.1)	851 (673.7 to 1056.2)	9 (7.2 to 11.1)	851 (673.7 to 1056.2)	-0.01 (-0.14 to 0.12)
Lebanon	23.1 (19.9 to 26.7)	1095.6 (935.6 to 1281.3)	23.1 (19.9 to 26.7)	1095.6 (935.6 to 1281.3)	0.20 (0.04 to 0.37)
Lesotho	18.5 (15.7 to 22)	1826.1 (1569.3 to 2108.4)	18.5 (15.7 to 22)	1826.1 (1569.3 to 2108.4)	-1.14 (-1.33 to -0.95)
Liberia	19.1 (14.8 to 24.8)	1355.6 (1111.2 to 1658.3)	19.1 (14.8 to 24.8)	1355.6 (1111.2 to 1658.3)	-1.48 (-1.69 to -1.28)
Libya	35.1 (29.9 to 41.8)	1216.7 (1030.3 to 1436.8)	35.1 (29.9 to 41.8)	1216.7 (1030.3 to 1436.8)	0.22 (0.06 to 0.38)
Lithuania	13 (10.4 to 16.3)	840.1 (667.6 to 1052.4)	13 (10.4 to 16.3)	840.1 (667.6 to 1052.4)	-0.13 (-0.26 to 0.00)
Luxembourg	2.4 (2 to 2.9)	1262.8 (1031.2 to 1520.9)	2.4 (2 to 2.9)	1262.8 (1031.2 to 1520.9)	0.54 (0.45 to 0.63)
Madagascar	201.4 (157.7 to 264.5)	2491.8 (2097.3 to 3016)	201.4 (157.7 to 264.5)	2491.8 (2097.3 to 3016)	-1.42 (-1.56 to -1.28)
Malawi	99.8 (69.3 to 145.3)	1378.2 (1058.7 to 1814.9)	99.8 (69.3 to 145.3)	1378.2 (1058.7 to 1814.9)	-1.68 (-1.89 to -1.47)
Malaysia	276.4 (239.1 to 319.6)	2892 (2477.7 to 3377.1)	276.4 (239.1 to 319.6)	2892 (2477.7 to 3377.1)	-0.33 (-0.61 to -0.05)
Maldives	7.3 (5.8 to 8.9)	5064.8 (4153.8 to 5984.7)	7.3 (5.8 to 8.9)	5064.8 (4153.8 to 5984.7)	0.10 (-0.10 to 0.29)
Mali	174.5 (151.7 to 204.6)	2542.9 (2228.1 to 2929.5)	174.5 (151.7 to 204.6)	2542.9 (2228.1 to 2929.5)	-1.27 (-1.51 to -1.03)
Malta	2 (1.5 to 2.5)	1104.4 (879.6 to 1394.5)	2 (1.5 to 2.5)	1104.4 (879.6 to 1394.5)	0.19 (0.05 to 0.34)
Marshall Islands	0.4 (0.3 to 0.4)	1300.3 (1124.5 to 1490.9)	0.4 (0.3 to 0.4)	1300.3 (1124.5 to 1490.9)	-0.44 (-0.58 to -0.30)
Mauritania	28.2 (24.5 to 33.1)	1976.9 (1739.8 to 2249.2)	28.2 (24.5 to 33.1)	1976.9 (1739.8 to 2249.2)	-1.35 (-1.51 to -1.20)
Mauritius	12.8 (11.3 to 14.6)	2455.1 (2168.2 to 2798)	12.8 (11.3 to 14.6)	2455.1 (2168.2 to 2798)	-0.58 (-0.71 to -0.45)
Mexico	727.3 (602.1 to 876.2)	1858.2 (1550.3 to 2194.1)	727.3 (602.1 to 876.2)	1858.2 (1550.3 to 2194.1)	-0.73 (-1.35 to -0.10)
Micronesia (Federated States of)	0.8 (0.6 to 0.9)	1168.5 (1012.4 to 1341)	0.8 (0.6 to 0.9)	1168.5 (1012.4 to 1341)	-0.54 (-0.75 to -0.32)
Monaco	0.2 (0.1 to 0.2)	1075.5 (858.1 to 1342.7)	0.2 (0.1 to 0.2)	1075.5 (858.1 to 1342.7)	0.11 (0.00 to 0.23)
Mongolia	5.9 (4.9 to 7.2)	391.2 (322.7 to 476.7)	5.9 (4.9 to 7.2)	391.2 (322.7 to 476.7)	-0.34 (-0.71 to 0.02)
Montenegro	2.3 (1.9 to 2.8)	797.7 (644.4 to 985)	2.3 (1.9 to 2.8)	797.7 (644.4 to 985)	1.19 (1.05 to 1.33)
Morocco	165.8 (138.6 to 197.5)	1061.8 (899.2 to 1249.1)	165.8 (138.6 to 197.5)	1061.8 (899.2 to 1249.1)	0.06 (-0.15 to 0.27)
Mozambique	144.6 (121.9 to 178.6)	1524.3 (1325.1 to 1799.5)	144.6 (121.9 to 178.6)	1524.3 (1325.1 to 1799.5)	-2.52 (-2.74 to -2.30)
Myanmar	434.7 (369.2 to 506)	1860.7 (1616.7 to 2123.9)	434.7 (369.2 to 506)	1860.7 (1616.7 to 2123.9)	-0.46 (-0.71 to -0.21)
Namibia	13 (11 to 15.6)	1515.1 (1297.9 to 1759.8)	13 (11 to 15.6)	1515.1 (1297.9 to 1759.8)	-0.76 (-1.01 to -0.51)
Nauru	0.1 (0.1 to 0.1)	1278.3 (1114.4 to 1471.6)	0.1 (0.1 to 0.1)	1278.3 (1114.4 to 1471.6)	-0.31 (-0.48 to -0.14)
Nepal	389.2 (299.3 to 502.8)	3125.9 (2577.3 to 3758)	389.2 (299.3 to 502.8)	3125.9 (2577.3 to 3758)	-0.31 (-0.37 to -0.25)
Netherlands	86.3 (69.3 to 108.7)	1174.5 (955.9 to 1462.7)	86.3 (69.3 to 108.7)	1174.5 (955.9 to 1462.7)	0.45 (0.31 to 0.60)
New Zealand	9.6 (8.1 to 11.4)	601 (513 to 706.5)	9.6 (8.1 to 11.4)	601 (513 to 706.5)	-0.13 (-0.23 to -0.04)

Continued on next page

Table S18 – continued from previous page

Country	1990		2019		1990–2019
	Incident cases No.×10^4[95% UI]	ASR per 10,0000 No.[95% UI]	Incident cases No.×10^4[95% UI]	ASR per 10,0000 No.[95% UI]	EAPC No.[95% CI]
Nicaragua	18.9 (15.8 to 23)	866.5 (737.4 to 1019.6)	18.9 (15.8 to 23)	866.5 (737.4 to 1019.6)	-1.51 (-1.77 to -1.25)
Niger	156.1 (133.7 to 188.6)	1957.3 (1717.8 to 2283.7)	156.1 (133.7 to 188.6)	1957.3 (1717.8 to 2283.7)	-1.08 (-1.21 to -0.95)
Nigeria	1497.2 (1109.9 to 1994.8)	2190 (1740.7 to 2707.6)	1497.2 (1109.9 to 1994.8)	2190 (1740.7 to 2707.6)	-0.19 (-0.53 to 0.15)
Niue	0 (0 to 0)	1379.5 (1209 to 1568.1)	0 (0 to 0)	1379.5 (1209 to 1568.1)	-0.01 (-0.11 to 0.09)
North Macedonia	6.6 (5.2 to 8.4)	690.6 (547.9 to 874)	6.6 (5.2 to 8.4)	690.6 (547.9 to 874)	1.10 (1.02 to 1.19)
Northern Mariana Islands	0.3 (0.3 to 0.4)	1295.8 (1135.2 to 1493.6)	0.3 (0.3 to 0.4)	1295.8 (1135.2 to 1493.6)	0.07 (-0.12 to 0.25)
Norway	24.8 (19.5 to 31.7)	1242 (977.5 to 1574.8)	24.8 (19.5 to 31.7)	1242 (977.5 to 1574.8)	0.74 (0.58 to 0.89)
Oman	22.8 (19.5 to 26.8)	1645.1 (1397.2 to 1931.5)	22.8 (19.5 to 26.8)	1645.1 (1397.2 to 1931.5)	-0.08 (-0.22 to 0.06)
Pakistan	2080.9 (1693.5 to 2608.8)	2388.3 (1986.3 to 2887.9)	2080.9 (1693.5 to 2608.8)	2388.3 (1986.3 to 2887.9)	-0.99 (-1.23 to -0.74)
Palau	0.1 (0.1 to 0.1)	1219.7 (1058.5 to 1395)	0.1 (0.1 to 0.1)	1219.7 (1058.5 to 1395)	-0.05 (-0.16 to 0.07)
Palestine	12.6 (11.1 to 14.2)	832 (725.4 to 959)	12.6 (11.1 to 14.2)	832 (725.4 to 959)	0.05 (-0.13 to 0.22)
Panama	7.5 (6.8 to 8.4)	574.2 (516.4 to 640.9)	7.5 (6.8 to 8.4)	574.2 (516.4 to 640.9)	-1.23 (-1.40 to -1.07)
Papua New Guinea	49.5 (40.9 to 61.4)	1702 (1436.4 to 2043.2)	49.5 (40.9 to 61.4)	1702 (1436.4 to 2043.2)	0.20 (-0.22 to 0.63)
Paraguay	14.6 (12 to 17.5)	742.7 (626.1 to 875.1)	14.6 (12 to 17.5)	742.7 (626.1 to 875.1)	0.94 (0.77 to 1.11)
Peru	59.9 (50.3 to 69.9)	568 (482 to 656.1)	59.9 (50.3 to 69.9)	568 (482 to 656.1)	-1.36 (-1.49 to -1.22)
Philippines	684.8 (553 to 856.8)	1851.8 (1526.8 to 2233)	684.8 (553 to 856.8)	1851.8 (1526.8 to 2233)	-1.02 (-1.18 to -0.86)
Poland	144.3 (112.5 to 181.8)	883.5 (687.8 to 1131.4)	144.3 (112.5 to 181.8)	883.5 (687.8 to 1131.4)	0.70 (0.50 to 0.91)
Portugal	71.6 (56.8 to 89.2)	1540.6 (1232.2 to 1904.5)	71.6 (56.8 to 89.2)	1540.6 (1232.2 to 1904.5)	0.36 (0.16 to 0.56)
Puerto Rico	16.3 (13.6 to 19.2)	964.9 (808.2 to 1133.7)	16.3 (13.6 to 19.2)	964.9 (808.2 to 1133.7)	0.44 (0.26 to 0.62)
Qatar	3.2 (2.6 to 3.9)	1143.2 (948.2 to 1374.4)	3.2 (2.6 to 3.9)	1143.2 (948.2 to 1374.4)	0.19 (0.10 to 0.27)
Republic of Korea	148.7 (120.2 to 177.5)	741.9 (604.5 to 883.3)	148.7 (120.2 to 177.5)	741.9 (604.5 to 883.3)	-0.86 (-1.06 to -0.66)
Republic of Moldova	15.2 (12.1 to 18.9)	727.4 (575 to 916.2)	15.2 (12.1 to 18.9)	727.4 (575 to 916.2)	0.58 (0.45 to 0.70)
Romania	83.3 (67 to 102)	858.6 (688 to 1062.7)	83.3 (67 to 102)	858.6 (688 to 1062.7)	0.69 (0.55 to 0.83)
Russian Federation	547.9 (442.9 to 663)	884.9 (717.8 to 1074.3)	547.9 (442.9 to 663)	884.9 (717.8 to 1074.3)	0.18 (0.04 to 0.31)
Rwanda	62.2 (53.9 to 72.2)	1368.5 (1192.5 to 1551)	62.2 (53.9 to 72.2)	1368.5 (1192.5 to 1551)	-2.18 (-2.41 to -1.94)
Saint Kitts and Nevis	0.2 (0.2 to 0.2)	1055.3 (900 to 1231)	0.2 (0.2 to 0.2)	1055.3 (900 to 1231)	-0.12 (-0.26 to 0.01)
Saint Lucia	0.8 (0.6 to 0.9)	1131 (973.6 to 1309.5)	0.8 (0.6 to 0.9)	1131 (973.6 to 1309.5)	-0.64 (-0.78 to -0.50)

Continued on next page

Table S18 – continued from previous page

Country	1990		2019		1990–2019
	Incident cases No.×10^4[95% UI]	ASR per 10,0000 No.[95% UI]	Incident cases No.×10^4[95% UI]	ASR per 10,0000 No.[95% UI]	EAPC No.[95% CI]
Saint Vincent and the Grenadines	0.8 (0.7 to 1)	1624.4 (1379.8 to 1911.6)	0.8 (0.7 to 1)	1624.4 (1379.8 to 1911.6)	-1.30 (-1.44 to -1.15)
Samoa	0.7 (0.6 to 0.9)	941 (784.2 to 1134.1)	0.7 (0.6 to 0.9)	941 (784.2 to 1134.1)	0.01 (-0.12 to 0.13)
San Marino	0.1 (0.1 to 0.2)	1077.2 (857.2 to 1346.2)	0.1 (0.1 to 0.2)	1077.2 (857.2 to 1346.2)	0.23 (0.09 to 0.36)
Sao Tome and Principe	1.2 (1 to 1.5)	1450.6 (1220 to 1722.4)	1.2 (1 to 1.5)	1450.6 (1220 to 1722.4)	-1.29 (-1.47 to -1.11)
Saudi Arabia	140.9 (117.5 to 168.2)	1383.9 (1163.3 to 1638.5)	140.9 (117.5 to 168.2)	1383.9 (1163.3 to 1638.5)	-0.29 (-0.41 to -0.16)
Senegal	76.8 (66.4 to 90.5)	1355.8 (1179.8 to 1562.2)	76.8 (66.4 to 90.5)	1355.8 (1179.8 to 1562.2)	-0.72 (-0.85 to -0.59)
Serbia	37.2 (28.9 to 47.5)	982.1 (770 to 1259.9)	37.2 (28.9 to 47.5)	982.1 (770 to 1259.9)	0.44 (0.33 to 0.55)
Seychelles	0.4 (0.4 to 0.5)	1198.4 (1052.6 to 1359.6)	0.4 (0.4 to 0.5)	1198.4 (1052.6 to 1359.6)	0.02 (-0.05 to 0.09)
Sierra Leone	49.1 (38.6 to 63.2)	1747.2 (1448.9 to 2144.1)	49.1 (38.6 to 63.2)	1747.2 (1448.9 to 2144.1)	-1.13 (-1.28 to -0.98)
Singapore	11.1 (9.2 to 13.5)	838.9 (716.4 to 989)	11.1 (9.2 to 13.5)	838.9 (716.4 to 989)	0.05 (-0.07 to 0.17)
Slovakia	17.9 (14.1 to 22.5)	781.3 (612.9 to 996.3)	17.9 (14.1 to 22.5)	781.3 (612.9 to 996.3)	0.66 (0.59 to 0.73)
Slovenia	6.1 (4.8 to 7.7)	795 (617.8 to 1015.5)	6.1 (4.8 to 7.7)	795 (617.8 to 1015.5)	0.52 (0.46 to 0.59)
Solomon Islands	3.3 (2.7 to 4.3)	1426.1 (1193.2 to 1716.2)	3.3 (2.7 to 4.3)	1426.1 (1193.2 to 1716.2)	-0.63 (-0.75 to -0.51)
Somalia	115.5 (94.6 to 143)	2183.6 (1876.5 to 2564.6)	115.5 (94.6 to 143)	2183.6 (1876.5 to 2564.6)	-1.66 (-1.96 to -1.36)
South Africa	229 (176.6 to 306.1)	1191.1 (940.9 to 1528.9)	229 (176.6 to 306.1)	1191.1 (940.9 to 1528.9)	-0.83 (-1.10 to -0.56)
South Sudan	95.4 (81.7 to 112.8)	2474.5 (2150.4 to 2828.8)	95.4 (81.7 to 112.8)	2474.5 (2150.4 to 2828.8)	-1.77 (-2.01 to -1.52)
Spain	227.3 (179.3 to 290.1)	1239.3 (991.7 to 1563.8)	227.3 (179.3 to 290.1)	1239.3 (991.7 to 1563.8)	0.30 (0.14 to 0.45)
Sri Lanka	486.2 (412.3 to 566.7)	5876.1 (4963.3 to 6809.5)	486.2 (412.3 to 566.7)	5876.1 (4963.3 to 6809.5)	0.20 (-0.28 to 0.68)
Sudan	418.2 (331.2 to 544.3)	2352.2 (1970.7 to 2890.9)	418.2 (331.2 to 544.3)	2352.2 (1970.7 to 2890.9)	-0.37 (-0.79 to 0.06)
Suriname	2.2 (1.8 to 2.7)	1143.2 (960.6 to 1371.2)	2.2 (1.8 to 2.7)	1143.2 (960.6 to 1371.2)	-0.87 (-1.01 to -0.73)
Sweden	52.1 (41 to 66.5)	1261.9 (1001 to 1592.1)	52.1 (41 to 66.5)	1261.9 (1001 to 1592.1)	0.22 (0.02 to 0.43)
Switzerland	35.3 (27.2 to 46.1)	1078.7 (840.8 to 1392.5)	35.3 (27.2 to 46.1)	1078.7 (840.8 to 1392.5)	0.62 (0.52 to 0.73)
Syrian Arab Republic	145.7 (125.5 to 167.8)	1426.1 (1224.6 to 1646.2)	145.7 (125.5 to 167.8)	1426.1 (1224.6 to 1646.2)	0.00 (-0.15 to 0.16)
Taiwan (Province of China)	57.8 (49.2 to 67.5)	594.6 (510.1 to 689)	57.8 (49.2 to 67.5)	594.6 (510.1 to 689)	-1.26 (-1.49 to -1.02)
Tajikistan	38.8 (33.3 to 45.7)	895.2 (761.9 to 1056.8)	38.8 (33.3 to 45.7)	895.2 (761.9 to 1056.8)	-0.05 (-0.15 to 0.05)
Thailand	455.9 (394.2 to 522.2)	1762.7 (1520.9 to 2023.2)	455.9 (394.2 to 522.2)	1762.7 (1520.9 to 2023.2)	-0.29 (-0.35 to -0.23)
Timor-Leste	22.3 (16.8 to 29.7)	4153.2 (3375.7 to 5079.4)	22.3 (16.8 to 29.7)	4153.2 (3375.7 to 5079.4)	-1.19 (-1.32 to -1.06)
Togo	44.2 (34.9 to 57.1)	1600.3 (1327.4 to 1946.9)	44.2 (34.9 to 57.1)	1600.3 (1327.4 to 1946.9)	-0.82 (-1.02 to -0.61)
Tokelau	0 (0 to 0)	1268.8 (1113.2 to 1469.8)	0 (0 to 0)	1268.8 (1113.2 to 1469.8)	0.00 (-0.12 to 0.12)

Continued on next page

Table S18 – continued from previous page

Country	1990		2019		1990–2019
	Incident cases No.$\times 10^4$[95% UI]	ASR per 10,0000 No.[95% UI]	Incident cases No.$\times 10^4$[95% UI]	ASR per 10,0000 No.[95% UI]	EAPC No.[95% CI]
Tonga	0.6 (0.5 to 0.7)	1217.4 (1057.6 to 1403.5)	0.6 (0.5 to 0.7)	1217.4 (1057.6 to 1403.5)	-0.26 (-0.40 to -0.13)
Trinidad and Tobago	6 (5.2 to 6.9)	1041 (895.8 to 1211.9)	6 (5.2 to 6.9)	1041 (895.8 to 1211.9)	-0.59 (-0.73 to -0.44)
Tunisia	59.2 (49.4 to 71.4)	1190 (997 to 1426.6)	59.2 (49.4 to 71.4)	1190 (997 to 1426.6)	0.03 (-0.09 to 0.15)
Turkey	216 (173 to 268.4)	612.5 (489.3 to 751.3)	216 (173 to 268.4)	612.5 (489.3 to 751.3)	1.89 (1.56 to 2.22)
Turkmenistan	22.1 (18.4 to 27.8)	799.9 (665.4 to 988.6)	22.1 (18.4 to 27.8)	799.9 (665.4 to 988.6)	-0.22 (-0.34 to -0.10)
Tuvalu	0.1 (0.1 to 0.1)	1412.1 (1204.2 to 1667.8)	0.1 (0.1 to 0.1)	1412.1 (1204.2 to 1667.8)	-0.71 (-0.84 to -0.59)
Uganda	152.5 (120.9 to 199.4)	1145.2 (944.4 to 1392)	152.5 (120.9 to 199.4)	1145.2 (944.4 to 1392)	-1.94 (-2.07 to -1.81)
Ukraine	224.3 (185.7 to 272.7)	1096.6 (909.4 to 1327.4)	224.3 (185.7 to 272.7)	1096.6 (909.4 to 1327.4)	0.28 (0.14 to 0.41)
United Arab Emirates	16.9 (14.5 to 19.9)	1444.6 (1241.3 to 1702.7)	16.9 (14.5 to 19.9)	1444.6 (1241.3 to 1702.7)	0.34 (0.22 to 0.46)
United Kingdom	323.9 (255.5 to 415.4)	1234 (977 to 1559.9)	323.9 (255.5 to 415.4)	1234 (977 to 1559.9)	-0.01 (-0.30 to 0.29)
United Republic of Tanzania	227.3 (185.5 to 279.5)	1175.3 (1010.7 to 1368.6)	227.3 (185.5 to 279.5)	1175.3 (1010.7 to 1368.6)	-2.13 (-2.29 to -1.97)
United States of America	915.2 (715.6 to 1141.7)	730.8 (571.5 to 910.5)	915.2 (715.6 to 1141.7)	730.8 (571.5 to 910.5)	-0.07 (-0.26 to 0.12)
United States Virgin Islands	0.5 (0.4 to 0.6)	984.6 (839.4 to 1158.7)	0.5 (0.4 to 0.6)	984.6 (839.4 to 1158.7)	-0.35 (-0.49 to -0.21)
Uruguay	10.5 (8.8 to 12.5)	698.8 (586.8 to 829.9)	10.5 (8.8 to 12.5)	698.8 (586.8 to 829.9)	0.25 (0.05 to 0.45)
Uzbekistan	139.4 (124.1 to 155.9)	897.8 (796.4 to 1009.1)	139.4 (124.1 to 155.9)	897.8 (796.4 to 1009.1)	-0.19 (-0.29 to -0.09)
Vanuatu	1.1 (0.9 to 1.3)	1134.1 (974.5 to 1308.4)	1.1 (0.9 to 1.3)	1134.1 (974.5 to 1308.4)	-0.07 (-0.19 to 0.06)
Venezuela (Bolivarian Republic of)	119.7 (102.2 to 140.5)	1235.8 (1043.2 to 1448.4)	119.7 (102.2 to 140.5)	1235.8 (1043.2 to 1448.4)	-0.50 (-0.79 to -0.21)
Viet Nam	786.4 (686.6 to 908.3)	2159.5 (1877.9 to 2491.9)	786.4 (686.6 to 908.3)	2159.5 (1877.9 to 2491.9)	-0.25 (-0.41 to -0.09)
Yemen	461.9 (357.6 to 601.6)	4325 (3471.3 to 5378.3)	461.9 (357.6 to 601.6)	4325 (3471.3 to 5378.3)	-1.25 (-1.39 to -1.11)
Zambia	77.6 (57.8 to 103.8)	1330.2 (1093.2 to 1634)	77.6 (57.8 to 103.8)	1330.2 (1093.2 to 1634)	-0.78 (-0.93 to -0.63)
Zimbabwe	86.4 (68.8 to 107.3)	1585.8 (1338 to 1866.2)	86.4 (68.8 to 107.3)	1585.8 (1338 to 1866.2)	-0.10 (-0.28 to 0.09)

Abbreviation: ASR, age standardized rate; CI, confidence interval; EAPC, estimated annual percentage change; UI, uncertainty interval.
